# New insights into the germline genes and CDR3 repertoire of the TCRβ chain in *Chiroptera*

**DOI:** 10.3389/fimmu.2023.1147859

**Published:** 2023-03-27

**Authors:** Hao Zhou, Jun Li, Dewei Zhou, Yingjie Wu, Xingliang Wang, Jiang Zhou, Qingqing Ma, Xinsheng Yao, Long Ma

**Affiliations:** ^1^ Department of Immunology, Center of Immunomolecular Engineering, Innovation & Practice Base for Graduate Students Education, Zunyi Medical University, Zunyi, China; ^2^ Department of Genome Informatics, Research Institute for Microbial Diseases, Osaka University, Suita, Japan; ^3^ School of Life Sciences, Guizhou Normal University, Guiyang, China

**Keywords:** bat, TR loci annotation, high-throughput sequencing, TCRβ chain repertoire, germline genes

## Abstract

**Introduction:**

Bats are recognized as natural reservoirs for many viruses, and their unique immune system enables them to coexist with these viruses without frequently exhibiting disease symptoms. However, the current understanding of the bat adaptive immune system is limited due to the lack of a database or tool capable of processing T-cell receptor (TCR) sequences for bats.

**Methods:**

We performed germline gene annotation in three bat species using homologous genes and RSSs (Recombinational Signal Sequences) scanning method. Then we used the conserved C gene to construct the TCRβ chain receptor library of the Intermediate Horseshoe Bat. Bats' TCRβ data will be analyzed using MiXCR and constructed reference library.

**Results:**

Regarding the annotation results, we found that the Pale Spear-nosed Bat has 37 members in the TRBV12 family, which is more than the total number of TRBV genes in the Greater Horseshoe Bat. The average number of unique TCRβ chain receptor sequences in each Intermediate Horseshoe Bat sample reached 24,904.

**Discussion:**

The distinct variations in the distribution of TRBV genes among the three types of bats could have a direct impact on the diversity of the TCR repertoire, as evidenced by the presence of conserved amino acids that indicate the T-cell recognition of antigens in bats is MHC-restricted. The bats’ TCRβ repertoire is formed through the rearrangement of the V-D-J-C genes, with D-J/V-D deletions and insertions resulting in high diversity.

## 1 Introduction

Bats belong to the order Chiroptera, are the second largest order of mammals in the world (1). Despite carrying numerous virulent zoonotic viruses, bats do not often show serious clinical symptoms (2–5). This has led to increased interest in the differences between the bat immune system and those of other mammals.

Studies have identified several differences between the innate immune systems of bats and those of humans or mice. For instance, in humans or mice, activation of pattern recognition receptors (PRRs) by RNA viruses, danger signals, or intracellular double-stranded DNA triggers the activation of NLR-family pyrin domain containing 3 (NLRP3) or Interleukin-1β (IL-1β), resulting in inflammation. In contrast, bats inhibit the transcriptional initiation of NLRP3, leading to reduced functionality of interferon-inducible proteins AIM2 and IFI-16 (6–9), This, in turn, leads to lower caspase-1 activity and IL-1β cleavage, resulting in overall reduced inflammation. A recent study also found that deletion of the 358th serine site of the bat STING protein inhibits interferon secretion (10). Moreover, Pavlovich et al. reported a “high amplification and non-classical distribution of genes” at MHC-I loci in bats, suggesting that the combination of these abundant non-classically distributed MHC-I type genes with highly expressed NKG2, which contain inhibitory interaction motifs, increases the activation threshold of NK cells and reduces their response (11, 12).

The lack of suitable reagents and models for cellular biology studies, the challenge of isolating viruses, and the diverse nature of bat species (belonging to 2 orders, 21 families, and over 1350 species) hinder the understanding of the adaptive immune system of bats (13). Next Generation Sequencing (NGS) technology has facilitated high-throughput sequencing (HTS) for genome analysis of various species. The Bat1k Project and Vertebrate Genome Project (VGP) have produced high-quality bat genomic data, which has improved our knowledge of the bat immune system, particularly their response to viral infections (14, 15). However, the annotation of T/B cell receptor germline genes in bats remains limited. The TCR/BCR repertoire comprises all functional T or B cells in an individual’s circulatory system, each with its own antigen-specific receptor. Deep sequencing of the repertoire is a commonly used method for studying the adaptive immune system, assessing an individual’s health status, developing antibodies, and detecting and treating targeted diseases (16). Various tools, such as IMGT/HighV-QUEST, TRUST, MiXCR, have been used to process TCR/BCR sequences (17–19). Our previous work involved annotating *R. ferrumequinum* TRB loci, indicating that the bat’s adaptive immune response is similar to that of humans and mice, and that its TCR is rearranged from germline genes with high diversity (20). In this study, we expanded the annotation to include the TRB loci of P. discolor and *P. pipistrellus*, and compared the TRB loci of all three bat species. The TRBC Exon1 sequences of various bats were aligned, and primers were developed to amplify the TCR β-chain CDR3 repertoire of *R. affinis* using the 5’ Rapid Amplification of cDNA Ends (5’RACE) technique. Our study provides a crucial theoretical foundation, a novel research method, and a comparable database for studying genetic evolution and adaptive immune response in Chiroptera.

## 2 Materials and methods

### 2.1 TRB locus location and annotation

**Figure 1:**
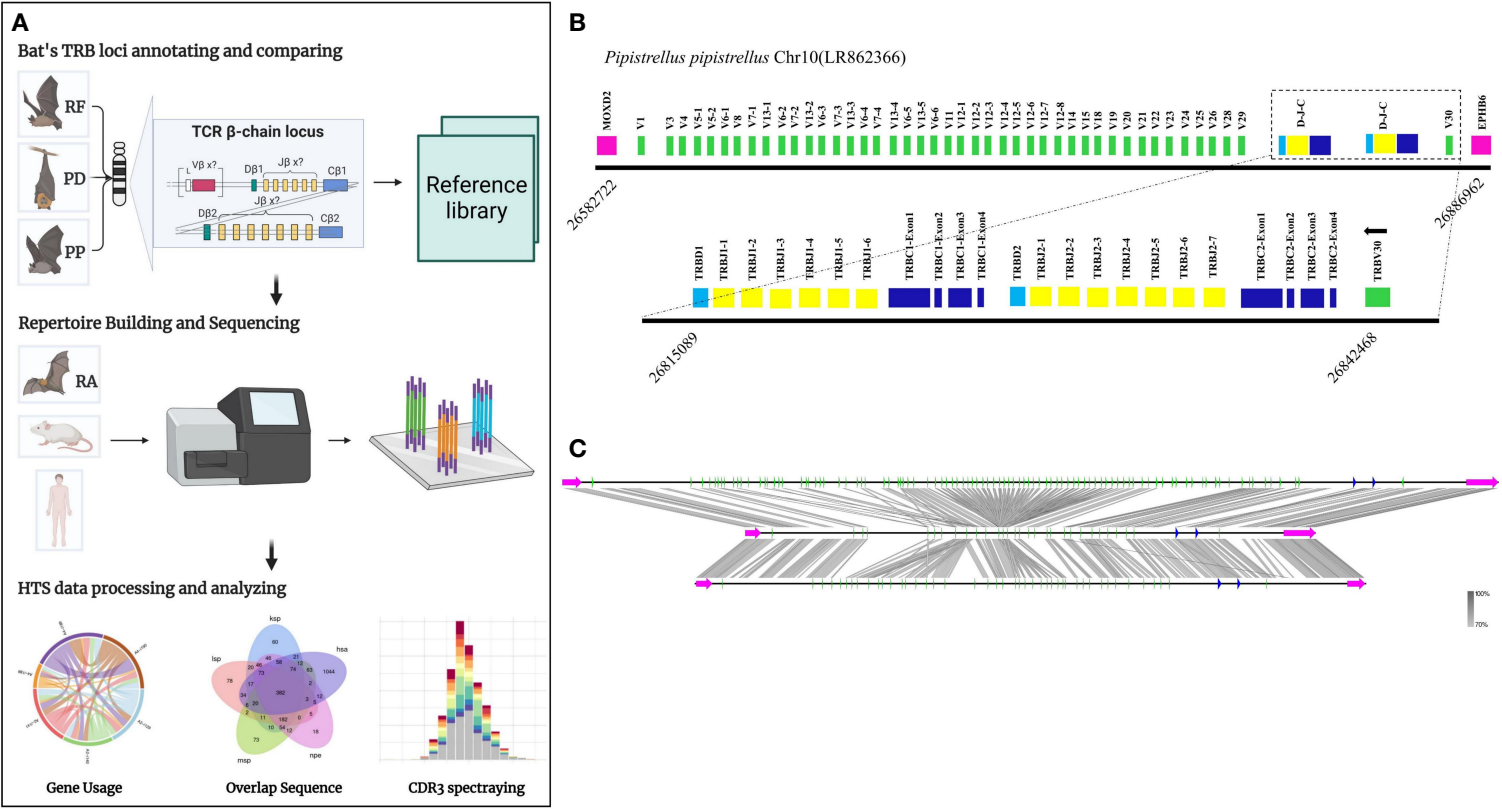
The TRB loci annotation and correlation analysis of three bat species. **(A)** Overview of this study design. **(B)** P. pipistrellus TRB loci on Chromosome 10, with colored boxes representing each annotated gene, including 44 TRBV genes, 2 TRBD genes, 13 TRBJ genes, and 2 TRBC genes. **(C)** A genome homology comparison map of the TRB loci for the three annotated bat species.

**Figure 1A** presents the experimental flow of the study, which involved identifying the TRB loci in the genome by detecting two genes, MOXD2 and EPHB6, located at the boundary of the TRB loci. Two gene annotation methods were used to annotate the regions between the two genes, including IMGT-LIGMotif (21) and 12/23 RSS(Recombination Signal Sequence) scanning (22). For the IMGT-LIGMotif method, representative animals from three different subjects, namely human(primate), mouse(rodents), and pig(artiodactyla), were selected, and TRBV genes of all known species in IMGT were obtained to ensure that no information was missed due to evolutionary divergence. The mapping of these genes to the TRB loci of the three bat species was performed using Geneious Prime (Version 2022.2.1). The 12/23 RSS scanning method involved screening all RSS motifs within the three bat TRB loci and searching for V/D/J genes upstream and downstream of the RSS. The annotation result, including the number of genes and locus composition information, is summarized in **Table 1**. Homology of the TRB loci among the three annotated bats was compared using Easyfig (Version 2.2.5), with a threshold of 70% identity (**Figure 1C**). Following the IMGT guidelines (23), the key components of each germline gene within the locus were labeled after annotation with Geneious Prime, and the annotation file was exported in GenBank format (**Supplementary Data 1** and **Supplement Figure 1**).

**Table 1:** Number of germline genes retrived by homologous genes and RSSs method.

Species and Chromosome accession	Phyllostomus discolor Chr12(NC_040912.2)	Rhinolophus ferrumequinum Chr26(NC_046309)	Pipistrellus Pipistrellus Chr10(LR862366.1)
12/23RSS	96V、 2D、 13J	30V、 2D、 14J	43V、 2D、 13J
IMGT/LIGMotif (Homo Sapiens)	99V、 8J	30V、 2D、 12J	44V、 9J
IMGT/LIGMotif (Mus musculus)	92V、 10J	30V、 2D、 13J	44V、 9J
IMGT/LIGMotif (Sus scrofa)	97V、 10J	30V、 2D、 14J	44V、 9J
Geneious Prime Mapping	64V、 2D、	30V、 14J	34V、 13J
Total Number of Germline Genes	100TRBV、 2TRBD、 13TRBJ、 2TRBC	30TRBV、 2TRBD、 15TRBJ、 2TRBC	45TRBV、 2TRBD、 13TRBJ、 2TRBC
Organization of TRB loci	Vβ(99)-Dβ(1)-Jβ(6)-Cβ(1)-Dβ(1)-Jβ(7)-Cβ(1)- Vβ(1)	Vβ(30)-Dβ(1)-Jβ(6)-Cβ(1)-Dβ(1)-Jβ(9)-Cβ(1)- Vβ(1)	Vβ(44)-Dβ(1)-Jβ(6)-Cβ(1)-Dβ(1)-Jβ(7)-Cβ(1)-Vβ(1)

### 2.2 Naming of germline genes and clustering of TRBV families

**Figure 2:**
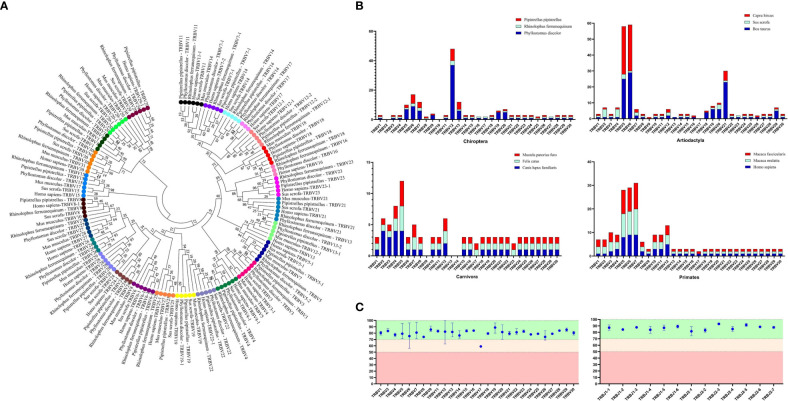
Analysis of TRBV gene homology in different bat species, as well as in human, mice, and pigs. **(A)** An NJ-phylogenetic tree was constructed from functional TRBV gene nucleotide sequences, with each color representing one family and bootstrap is 1000. **(B)** A comparison of TRBV gene family distribution in three mammalian orders (Carnivora, Artiodactyla, and Primates) with the three bat species is shown, with data from https://www.imgt.org/IMGTrepertoire/. **(C)** A comparison of the nucleotide sequence indentity of TRBV and TRBJ genes is presented for the nature annotated bat species.

The germline gene names were assigned following the guidelines provided by IMGT. In order to classify V genes into families, a threshold of 75% nucleotide identity was set. To verify familial homology, a phylogenetic tree of TRBV nucleotide sequences was constructed using the Neighbor-Joining method in MEGA7 (version 7.0.26). The TRBV genes from human, mouse, and pig were retrieved from IMGT-GeneDB (https://www.imgt.org/genedb/), and only functional genes and ORFs were selected, with one gene per family. **Supplementary Table 1** lists the accession numbers used in the analysis. The distribution of TRBV gene families in twelve mammalian species belonging to four different subjects (Artiodactyla, Carnivores, and Primates) was compared to understand the evolutionary direction of the V gene (**Figure 2A**). The D, J, and C genes were named based on their position within the cluster they belong to in the locus.

### 2.3 Establishment of reference library

To establish a reference library, we utilized Geneious Prime (Clustal Omega) to align the amino acid sequences of the germline V and J genes. Priority was given to aligning three conserved sites, 23CYS, 41TRP, and 104CYS, based on their known structure and specific positions in mammalian TRBV (23). Structural domains were classified based on alignment results, with V genes including FR1 (1–26), CDR1 (27–38), FR2 (39–55), CDR2 (56–65), FR3 (66-104), and CDR3 (105-117), and J genes including FR4 (phenylalanine to the end of the FGNG motif sequence). Functional descriptions of germline genes were based on IMGT, incorporating stop codons, RSS, conserved amino acids, and splice sites. **Supplementary Table 2** provides information on pseudogenes and ORFs. The germline gene nucleic sequences and structural domain information were exported as reference library files identifiable by MiXCR, enabling processing of bat TRB data.

### 2.4 Sample collection

Bat samples were collected with the assistance of Zhou’s group in Xishui County, Zunyi City, Guizhou Province. Muscle tissues were used to extract genetic information by amplifying the Cytb gene with designed primers (**Supplementary Table 5**), and PCR products were sequenced to determine bats’ genotypes based on E-value and Percent Identity. Although we did not find any annotated bat species based on BLAST results, we selected three *R. affinis* as our experimental model due to their expected high homology with the target species, *R. ferrumequinum.* Spleen tissues from three 5-month-old BALB/c mice were used for RNA extraction and TCRβ chain library construction. Moreover, we collected molecular cells from 5 ml of peripheral blood from three individuals at the Affiliated Hospital of Zunyi Medical University. The study was approved by the Animal Protection and Ethics Committee of Zunyi Medical University, with work involving humans approved under permit number (2021)1-022 and the bat and mouse project approved under permit number (2018)2-261.

### 2.5 TCR-β chain repertoire sequencing and analysis

To construct and amplify the TCR-β library for *R. affinis*, we collected and aligned fifteen known bats’ TRBC Exon1 nucleotide sequences(accession number in **Supplementary Table 1**), designed primers in conserved regions, and used the 5’RACE method to prepare repertoires of *R. affinis*. Library construction and sequencing were performed by ImmuQuad Company in Hangzhou, China. We used samples from humans and mice for species repertoire comparison. Spleen tissue was used for TCR-β repertoire construction in mice using the 5’RACE method, while peripheral blood mononuclear cells (PBMC) were collected from three individuals for humans, and the multiplex PCR method was used for TCR-β repertoire construction.

We analyzed the sequencing results of bats, humans, and mice using the MiXCR software (Version 3.0.13). MiXCR was used to assemble and present the sequencing results of bats based on the library constructed from annotated genes. The output data of the nine samples are shown in **Table 2** and **Supplementary Table 6**. To ensure the quality of the repertoire, we examined the sequences of bat1 sample, which contained 25 TRBV genes and 14 TRBJ genes (**Figure 4E**). The evolutionary relationship between the sequenced samples and the three annotated bats was determined by sequence attribution, Cytb, and TRBC gene alignment. To assess the homogeneity and heterogeneity of TCR-β repertoires among the three species, we used Productive-Clonetype sequences for subsequent analyses to exclude the effects of repertoire construction and sequencing, as well as physiological health status. Our analyses included: (1) statistics on repertoire overlap index among the three species; (2) usage of TRBV gene and TRBJ gene, V-J gene pairing, and tracking of conserved motifs; and (3) evaluation of CDR3 length and amino acid usage, as well as nucleotide deletion and insertion.

**Table 2:** MiXCR outputted sequence statistics from a total of nine samples, including bats, humans, and mice.

Sample ID	Total sequence counts	Total sequence clonetype	Total productive sequence counts	Productive sequence clonetype
Bat1	2557414	3498	2381182	3234
Bat2	4949687	25443	4199657	22971
Bat3	5303900	45771	4912794	41950
Human1	3844196	76589	3432143	76588
Human2	3872836	75533	3469357	75530
Human3	3931149	75037	3538969	75034
Mus1	17381732	1334514	7776887	1077810
Mus2	14093768	1233928	7604800	1073215
Mus3	15174528	1150536	9456533	1147763

### 2.6 Statistical analysis

The figures were draw with GraphPad Prism (Version 8.0.2), R package “ggplot2”, and R package “Immunarch”. Data analysis was performed by R studio (v3.3.3) and GraphPad Prism. P-values were calculated with the aid of the t test. P<0.05 was considered statistically significant.

## 3 Results

### 3.1 TR loci annotation and Reference directory establishment

**Table 1** records the summary of TRB loci using homologous genes and RSS methods. The TRB location in *P. discolor* was found on chromosome 10(NC_040912: 85517683-85943353), *R. ferrumequinum* on chromosome 26(NC_046309: 7911840-8171343), and *P. pipstrellus* on chromosome10(LR862366.1: 26582722-26886962). The TRB loci in all three species followed the classical mammalian structure, but with differences in length distribution and orientation as shown in **Figure 1B** and **Supplementary Figure 1**.

We identified 100 TRBV genes in *P. discolor* (including 2 pseudogenes), 30 in *R. ferrumequinum*, and 45 in *P. pipistrellus*. A phylogenetic tree was constructed using the nucleotide sequences of TRBV genes, which showed that all genes could be homologous to each other or to those found in pigs and humans. Functional V gene families did not cluster separately in bats (**Figure 2A**). The main differences in the number of germline genes were due to internal duplications and deletions of the V gene family. The evolutionary direction of Chiroptera and Artiodactyla was more similar, as shown in **Figure 2B**. Most of the TRBV and TRBJ gene families of the three bat species had more than 70% identity, except for the TRBV17 family (**Figure 2C**). We identified a specific TRBV12 family of *P. discolor* containing 37 members, which was close to almost all TRBV genes of *P. pipistrellus* and even exceeded the total TRBV genes of *R. ferrumequinum*.

**Figure 3:**
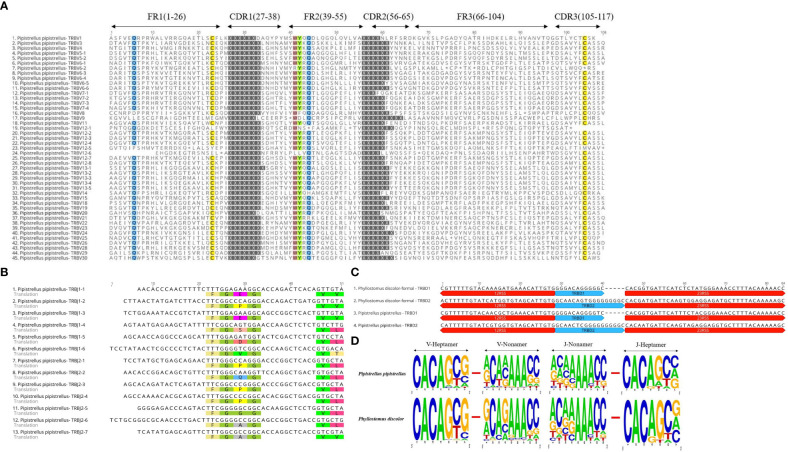
Annotated Germline Genes Display. **(A)** Displays 45 TRBV amino acid sequences with a consistency threshold of 90%. **(B)** Displays the nucleotide and amino acid sequences of 13 TRBJ genes with translated conserved FGNG motifs. **(C)** Shows four TRBD genes along with their 23RSS and 12RSS nucleotide sequences. **(D)** Compares the conserved sites of TRBV’23RSS and TRBJ’12RSS in P. discolor and P. pipistrellus.

In the Multiple Sequence Alignment (MSA) of TRBV genes, we identified several conserved sites, including 23Cys, 41Trp, 89Leu, and 104Cys, as well as Gln at position 6 and Tyr at position 42. Gln at position 44 was also highly conserved in *P. pipistrellus*. Additionally, most TRBV genes contained CASS motifs at the end (**Figure 3A** and **Supplementary Figure 2A**). *P. discolor* had a higher number of germline genes and more gene duplications, resulting in a greater number of pseudogenes (17%) compared to *R. ferrumequinum* (7%) and *P. pipistrellus* (11%). However, the number of functional genes (V and J genes) in *P. discolor*, which exceeded that of *R. ferrumequinum* and *P. pipistrellus*, was 97 (**Supplementary Table 2**). Like primates and carnivores, Chiroptera contains two D-J-C clusters named according to their chromosomal location. The only difference between the three bat species regarding the number of genes in each cluster was the number of J genes in the second cluster. All TRBJ genes in bats contained conserved FGNG motifs except for TRBJ2-3 in *R. ferrumequinum* (**Figure 3B** and **Supplementary Figure 2B**). In addition, all bats’ TRBD genes were guanine-rich sequences, with 23RSS and 12RSS downstream and upstream (**Figure 3C**). There were no significant differences in the RSS between bats and other mammals, and the most conserved site was the first four sites (CACA) of the heptamer, with three continuous adenosines in the middle position of the nonamer (**Figure 3D** and **Supplementary Figure 3**). We created a MiXCR reference library for HTS data analysis using nucleotide sequences and amino acid sequence MSA results. To organize the germline genes family of three bat species, we combined them into a single family with allelic nomenclature methods. (**Supplementary Table 3**).

### 3.2 TCRβ repertoire constructing, sequencing, analyzing

**Figure 4:**
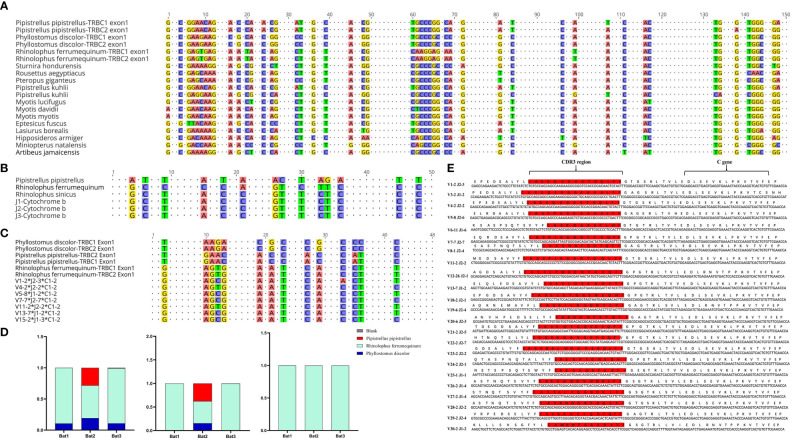
Homology analysis of R. affinis samples and annotated bat species. **(A)** Alignment of TRBC Exon1 nucleotide sequences of 15 Chiroptera species used for primer design in the 5’RACE method of R. affinis. **(B)** Comparison of Cytb nucleotide sequence results of R. affinis samples with P. discolor (PD), R. ferrumequinum (RF), and P. pipistrellus (PP). **(C)** Comparison of a partial C gene fragment from the bat1 sample with those of the three annotated bat species. **(D)** Attribution statistics of the sequencing results of the three R. affinis samples in the three annotated bat species. **(E)** Output of 25 TRBV genes and 14 TRBJ genes in the bat1 sample.

The Cytb sequencing results of the three experimental bats were analyzed, and an average sequence length of 488 base pairs and 98.5% sequence identity was observed. The bats were identified as *R. affinis* through a BLAST analysis, as shown in **Supplementary Table 4**. Furthermore, we constructed the TCRβ chain repertoire using the 5’RACE method, which involved comparing the conserved regions of the TRBC Exon1 nucleotide sequences of fifteen different types of bats and designing primers accordingly. The nucleotide sequence identity of 19 TRBC Exon1 varied from 77.66% to 98.9%, and several fully conserved regions were suitable for repertoire construction and sequencing, as illustrated in **Figure 4A**.

ImmuQuad Company conducted the sequencing, and the library was confirmed by a peak between 600 and 700 base pairs in the *R. affinis* samples, as shown in **Supplementary Figure 4**. MiXCR was used to process raw data from nine samples, which were analyzed and can be accessed at NCBI_PRJNA877449 (SRX17468175, SRX17468176, and SRX17468177). The 5’race method yielded an average of 4.27 million TCR reads and 24,904 clonotypes per bat sample, while the mouse samples had 15 million TCR reads and 1.23 million clonotypes per sample. The multiplex-PCR method resulted in 3.8 million TCR reads and 75,000 clonotypes per human sample. Despite variations in sequencing depth across bat samples, all samples met the CDR3 sequence depth analysis requirements for HTS sequencing, as indicated in **Table 2**.

To explore the degree of bat species divergence, we compared the Cytb nucleotide sequences of the three experimental bats (*R. affinis*) with those of *R. ferrumequinum* and *P. pipistrellus* (**Figure 4B**). The results showed that *R. ferrumequinum* had a higher identity (87.9%) to *R. affinis* than *P. pipistrellus* (74.6%). Additionally, the TRBC nucleotide fragment exhibited identity between R*. affinis* and *P. discolor*, *R. ferrumequinum*, and *P. pipistrellus*, with respective identities of 77.1%, 93.8%, and 75.0% (**Figure 4C**). After categorizing the sequencing results of the experimental samples, we found that most of the output sequences of *R. affinis* belonged to *R. ferrumequinum* (**Figure 4D**). These comparison results further indicated that the sequences of *R. affinis* were more similar to those of *R. ferrumequinum*. We then demonstrated the completeness of the repertoire by exporting 25 V and 14 J gene sequences from the Bat1 samples (**Figure 4E**).

### 3.3 Comparison of CDR3 repertoire in bats, humans, and mice

**Figure 5:**
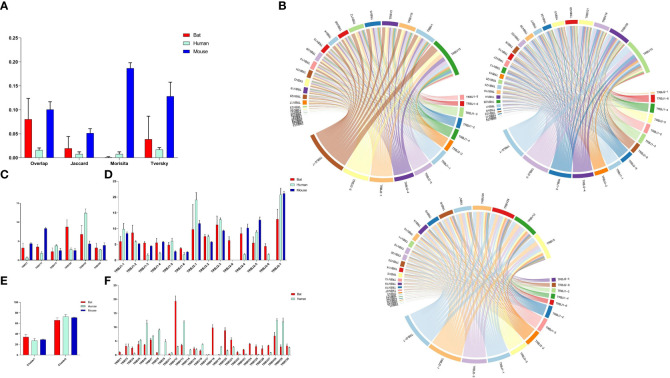
Comparative Analysis of TCR β Chain Repertoire Genes and Motif Usage in Bats, Humans, and Mice. **(A)** Overlap index of CDR3 sequences in the three species. **(B)** V-J pairing analysis of the three species, with the order being Bat1, Human1, and Mouse1. **(C)** Homologous TRBV gene usage analysis of the three species. **(D)** TRBJ gene usage analysis. **(E)** J gene usage in cluster. **(F)** TRBV gene usage analysis of bats and humans.

Four indices (overlap coefficient, Jaccard, Morisita, and Tversky) were used to assess the CDR3 repertoire in bats, humans, and mice (**Figure 5A**). The analysis showed that the bat CDR3 repertoires were variable and significantly different from each other, with some overlap coefficients slightly higher than humans but significantly lower than mice.

Based on the phylogenetic tree result, we analyzed the homology TRBV gene usage and V-J pairing (**Figure 5B** and **Supplementary Figure 5**) in bats, humans, and mice. Six homology TRBV genes were expressed, with TRBV15 and TRBV30 exhibiting similar usage (**Figure 5C**). Furthermore, we compared the usage of all TRBV genes in bats and humans (**Figure 5F**). The most commonly used TRBV genes in bats were TRBV12, TRBV18, and TRBV20, whereas in humans, they were TRBV13, TRBV28, and TRBV29. Notably, TRBV9 and TRBV19 were either not expressed or exhibited low expression in the bat samples. Additionally, the TRBV9 group was exclusive to *P. discolor*, with all four TRBV9 members being pseudogenes. Among the three species, all functional TRBJ genes, with the exception of TRBJ2-8 (pseudogene) in bats and TRBJ2-6 (pseudogene) in mice, were expressed (**Figure 5D**). Through comparative TRBJ analysis, we observed that high usage of TRBJ2-1, TRBJ2-3, and TRBJ2-7 occurred concurrently in bats, humans, and mice. Additionally, most of the J genes used in the three species were derived from the second D-J-C cluster (**Figure 5E**). This finding suggests a correlation between gene usage during rearrangement and distance.

**Figure 6:**
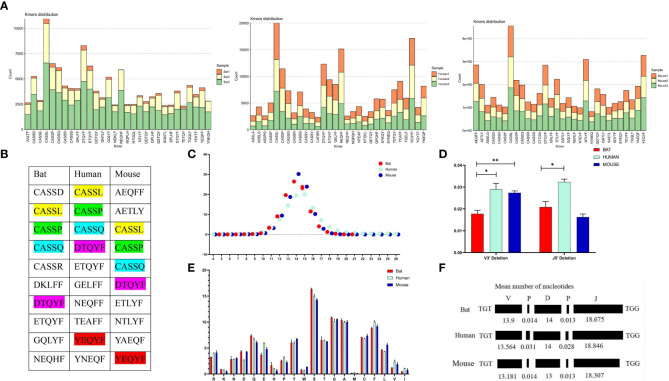
Comparative analysis of the CDR3 region features of the TCRβ chain repertoire in bat, human, and mouse is presented in this figure. **(A)** The top 30 5 AA length motifs used in the CDR3 region are analyzed. **(B)** The top 10 shared motifs are labeled in the same color. **(C)** The CDR3 region length is analyzed. **(D)** Deletions at the 3’ end of the V gene and 5’end of the J gene are compared. **(E)** AA usage in the CDR3 region is analyzed. **(F)** The composition of the CDR3 region is presented.

We analyzed the CDR3 region motifs (5 amino acids) and found that four of the top ten high-frequency motifs were shared among bats, humans, and mice, with most being CASSN motifs (**Figures 6A**, **B**). This suggests that the cell populations involved in the immune response in the three species may be similar. The CDR3 region length was also characterized, with a bell-shaped distribution of 14 amino acids for bats and mice, and 15 amino acids for humans (**Figure 6C**). We assessed the length effects caused by insertion and deletion of nucleotides in the CDR3 region and found that humans and mice had similar deletions at the V’3 end, while bat and mouse had similar insertions at the V 3’ end and the J gene 5’ end (**Figures 6D, E**). Finally, we observed that the amino acids in the CDR3 region were consistent, with high frequencies observed for S, G, and A in all three species (**Figure 6F**).

## 4 Discussion

The study of bat immunity has been hindered by the lack of basic genetic information, despite their importance in virus transmission and possession of unique physiological traits. Previous studies have identified T, B, and macrophage populations in *Pteropus giganteus*, with a higher T/B cell ratio in the spleen and lymph nodes when compared to mice (24). Further investigations have revealed that CD4^+^ T cells dominate the blood lymph, whereas CD8^+^ T cells dominate the spleen in fruit-eating bat *Pteropus alecto* (25). These findings suggest that T/B cell immunity plays a role in bats, but the understanding of TCR remains limited.

In 2021, the TR/IG loci of the Greater horseshoe bat and the Egyptian rousette bats were annotated separately (20, 26). However, no public database or tool has been used to analyze TCR/BCR sequences for bats, and the TCR/BCR repertoire characterization of bats has not been reported. To create a bat germline gene database, a complete annotation of TR/IG loci was a priority. It is important to note that the TR/IG loci annotation is different from normal genome annotation, requiring a high-quality genome due to the short genes (such as D and J genes) and the loci’s large span on the genome. For our research, we selected three bat species with high-quality genomes from the database: *R. ferrumequinum, P. discolor*, and *P. pipistrellus*. Creating germline gene databases involves identifying all potential genes based on specific structural features, which is the central issue. Due to the short sequence of D (10-15 bp) and J (30-50 bp) genes, we used the RSS sequence search method simultaneously to avoid losing sequences from the homology search. However, non-classical RSS (12/23+/-1 nt) may impact the final result, although such genes are rare in humans and mice. Moreover, pseudogenes lacking RSS cannot be involved in the rearrangement process, implying that they have no impact on the productive repertoire and pathogenic response (27).

In Chiroptera, previous studies have found varying degrees of replication of immune-related genes, but for TCR, gene duplication events were mostly reported in Artiodactyla such as cattle (28, 29) and goats (30), which have diverse T cell receptor pools. Surprisingly, massive gene duplication events of the TRBV12 family in *P. discolor* and considerable variation in TRBV gene numbers among Chiroptera have never been reported in mammals. In comparison, studies of primates (31–33), carnivores (34–36), and camels (37) have shown minimal differences in TRB loci structure and the number of germline genes among species of the same orders. The distribution and evolutionary direction of TRBV gene families in the three bat species are similar to Artiodactyla, with random and massive replications. The lack of gut-associated lymphoid tissue (GALT) in *R. Hildebrandti* and *P. pipistrellus* makes it unlikely that they possess a gene conversion-related mechanism to generate receptor diversity, as observed in the chicken immunoglobulin system (38–41). Instead, T cells use gene rearrangement as the predominant mechanism for generating TCR diversity in bats. This difference in rearrangement likelihood suggests that TCRβ chain repertoire diversity might be present in at least the three annotated bat species. The anchor residues in TRBV genes are important for MHC peptide binding, and while MHC genes in bats are more diverse than in other mammals, previous research has shown high sequence similarity between bat and human, mouse, dog, and cow MHC genes (42–44). Moreover, the anchor residues in the annotated TRBV and TRBJ genes of bats are highly conserved, indicating that TCR recognition in bats is MHC-restricted.

We have established a bat TCR β chain reference library and analyzed the TCR β chain repertoire using three *R. affinis* samples. From a total of 4.2 million reads on average, we found that the number of TCR clonotypes differed among the bat samples, with bat1 having 3498, while bat2 and bat3 had 25443 and 45771, respectively. There are several possible explanations for this variation. Firstly, it may be due to differences in the expression of TCRs between individual bats. This suggests that the diversity of individual bats may vary. Second, since this is the first time we have performed repertoire building and sequencing of bats, the sequencing depth may have an impact, and increasing the number of sequenced samples would help to address potential issues of individual bat diversity and low TCR expression due to limited sample collection. After analyzing the sequence output of each bat sample, we discovered that 25 TRBV genes and 14 TRBJ gene families were present in every experimental bat. This finding indicates that we have, for the first time, achieved a high level of completeness in identifying the TCR β chain CDR3 in bats. Our results provide valuable technical resources and data analysis tools to assist in the design and optimization of primers for high-throughput sequencing analysis of TCR repertoire from bats belonging to different families using 5’ RACE. The TCR β chain sequences of *R. affinis* were highly similar to the annotated genes of *R. ferrumequinum*. Specifically, 99% of the C genes belonged to *R. ferrumequinum*, and the TRBV and TRBJ genes were nearly identical to the annotated V and J genes of *R. ferrumequinum*. This suggests that certain immune response genes are genetically conserved among bat species of the same family.

We conducted an analysis on the usage and pairing patterns of TRBV and TRBJ genes in bats, humans, and mice. Despite humans having over 50 and mice over 30 germline V genes, their specific immune response may rely on only one or a few of them (45). The low expression levels of several gene families may be explained by a variety of factors. Firstly, it’s possible that the low expression levels are due to *R. affinis* itself. Additionally, the TRBV17 family has very low homology in the three annotated bats, which suggests that it may undergo significant mutations in the *R. affinis* genome and therefore not be detected. These factors could contribute to the overall low expression levels of certain gene families. A kind of mutation might lead to functional genes becoming pseudogenes, a situation that is very common across species. For example, the TRBV1 gene in bats is functional gene, while TRBV1 in humans is pseudogene. There are also cases where functional genes are not detected as expressed, for example, TRBV18 is barely expressed in humans but relatively highly expressed in bats; similar to TRBV23, TRBV24, TRBV26, etc. TRBV30, which is located in a specific position downstream of the second C gene and in a position opposite to transcription in bats, humans, and mice (TRBV31), was observed to be common in all three species. Several studies have reported that V and J gene usage during rearrangement correlates with position in the locus (46–48). Interestingly, in all three species, over 60% of the J gene is from the second D-J-C cluster. TRBV12-5 in humans and TRBV13-2 in mice are often linked to autoimmune diseases and account for over 50% of NK-T cells. Additionally, mouse MAIT cells express a TCR-α chain with TRAV1 and TRAJ33 and paired β chains of TRBV13 and TRBV19 (49–53). Notably, both TRBV12 in bats and TRBV13 in mice are highly utilized V genes. Bats exhibit significant differences from humans in their usage of multiple TRBV genes, indicating a specific biased amplification in the evolution of bat TCR immune response genes. This may be linked to evolutionary pressure or coevolution with viruses.

The usage of TRB CDR3 region amino acids is highly similar in bats, humans, and mice, with a consistent pattern of high-frequency motifs. However, there are significant differences in motif usage throughout the CDR3 region, indicating that the V-terminal and J-front ends of the CDR3 regions in these mammals are more conserved. This suggests that typical antigenic selection encountered in evolution may play a role. The length of the TRB CDR3 region is shorter in bats and mice compared to humans, which we attribute to differences in insertion and deletion at the V3’ and J5’ ends across the three species, as well as differences in the evolutionary length of the D gene and V/J involvement in the CDR3 region genes. While the overlap between individual bat CDR3 regions is higher than that of humans, it is significantly lower than that of mice. This finding suggests a higher concordance of CDR3 regions between different individual BALB/c mice with the same genetic background.

We annotated the complete TRB loci in three species of bats and constructed a database for analyzing the bat TCR repertoire. This allowed us to study the TCR evolution and specific immune response mechanism in bats and provide a new theory, technical tools, and data for comparative analysis of the mechanism of virus tolerance in bats.

## Data availability statement

The datasets presented in this study can be found in online repositories. The names of the repository/repositories and accession number(s) can be found below: https://www.ncbi.nlm.nih.gov/, PRJNA877449.

## Ethics statement

The studies involving human participants were reviewed and approved by Zunyi Medical University. The patients/participants provided their written informed consent to participate in this study. The animal study was reviewed and approved by Zunyi Medical University.

## Author contributions

XY, LM and HZ designed the experiment and wrote the paper, while LM and HZ conducted the experiments, analyzed data, and created graphs. Other contributors, including JL, LL, DZ, YW, XW, JZ and QM, assisted with sample collection, data analysis, manuscript revision and library construction for humans and mice. All authors contributed to the article and approved the submitted version.

## Supplementary material

Supplementary Data Sheet 1 GenBank files for Phyllostomus discolor, Rhinolophus ferrumequinum, and Pipistrellus pipistrellus.
