# Supplementary material for: New insights into the germline genes and CDR3 repertoire of the TCRβ chain in Chiroptera
Source: Front Immunol. 2023 Mar 27;14:1147859. doi: 10.3389/fimmu.2023.1147859 (PMC10083501; doi:10.3389/fimmu.2023.1147859)
Supplement: Supplementary Data Sheet 1 — GenBank files for Phyllostomus discolor, Rhinolophus ferrumequinum, and Pipistrellus pipistrellus. [file DataSheet_1.docx]

LOCUS Phyllostomus dis 558490 bp DNA linear UNA 23-JUL-2022

DEFINITION Phyllostomus discolor isolate MPI-MPIP mPhyDis1 chromosome 10,

mPhyDis1.pri.v3, whole genome shotgun sequence.

ACCESSION NC_040912

VERSION urn.local...1d-f3dsknw

KEYWORDS .

SOURCE Phyllostomus discolor (pale spear-nosed bat)

ORGANISM Phyllostomus discolor

Eukaryota; Metazoa; Chordata; Craniata; Vertebrata; Euteleostomi;

Mammalia; Eutheria; Laurasiatheria; Chiroptera; Microchiroptera;

Phyllostomidae; Phyllostominae; Phyllostomus.

FEATURES Location/Qualifiers

source <1..>558490

/organism="Phyllostomus discolor"

/mol_type="genomic DNA"

/isolate="MPI-MPIP mPhyDis1"

/db_xref="taxon:89673"

/chromosome=10

/sex="male"

/tissue_type="muscle"

/country="Germany: Munich, Collected from captive colony,

LMU"

/collection_date="25-Oct-2016"

/collected_by="Pedro Rodenas-Cuadrado, Uwe Firzlaff,

SonjaVernes"

/label="source Phyllostomus discolor"

gene complement(62734..71534)

/gene="LOC114507355"

/note="Derived by automated computational analysis using

gene prediction method: Gnomon."

/db_xref="GeneID:114507355"

/modified_by="Administrator"

/label="MOXD2"

mRNA 63392..63637

/%_Identity=100

/Motif="TCAGACTCCAGCCTGCAGGGTGGCGAGGAGCCAAGAGAAGGCCCCCTGCC

CAAACAAGAGGGCAGCCAGGGGGATGTGGCTCAGGGTGGCCGTTCCTGAGGCTGAGAG

GGGCATGGGGGCAGCAGCCCTGAAGCCCGCAGGGGTCTCGTCCTGGGGCTCCACTTTG

CCCCCCCATGACTCCAAGCAGGGCCCCCGAGGTGTGGGGATGATCTCTGGAATCCAGC

CTGTCATGTTTTCCACTATCTC"

/annotation_group="MOXD2 mRNA: 63,392 -> 63,637"

/modified_by="zhouhao"

/label="MOXD2 mRNA"

mRNA 76170..76226

/%_Identity=100

/Motif="ATGGCAGCACTTCCCATTCTGTGGGGCTGGGTGCTCGTGGCTGCCGTGAG

GACAG"

/annotation_group="L-part1: 76,170 -> 76,224"

/modified_by="zhouhao"

/label="L-part1"

mRNA 76495..76506

/created_by="zhouhao"

/modified_by="zhouhao"

/label="L-part2"

gene 76507..76793

/%_Identity=100

/Motif="TCAAGTTTTGTGGAGCAAAAGCCCAAGTGGGCCCTGGTGCGTCGCGGAGC

GGCTGAAACGCTGCAATGCTTTCTGAGGGATACCCAGTACCCCTGGATGAGCTGGTAC

CAGCAGGATCTCCAGGGGCAACTCCAGGTGCTGGCCACTCTGCGGTATTCTGGGGACA

AGGAGGTCATATCCCTTCCTGGAGCGGATTACCGGGTCATGCGCGTGGATGACAAGGA

GCTGAGGCTGCACGTGGCCAACGTGACACAGGGCAGAACCCTGTACTGCAGCTGCAGC

AAAGAC"

/annotation_group="TRBV1: 76,507 -> 76,794"

/modified_by="zhouhao"

/label="TRBV1"

sig_peptide 76794..76832

/Mismatches=0

/%_Identity=100

/Motif="cacagtgagaaactctccttagacaaatgaataaaaacc"

/annotation_group="23RSS: 76,794 -> 76,832"

/label="23RSS"

mRNA 121076..121126

/%_Identity=100

/Motif="ATGGGCCCCAGGTTCCTCTGCTGTGTGGCCCTCTGCCTCCTGGGAGCAG"

/annotation_group="L-part1: 121,076 -> 121,124"

/modified_by="zhouhao"

/label="L-part1"

mRNA 121232..121243

/created_by="zhouhao"

/label="L-part2"

gene 121244..121530

/%_Identity=100

/Motif="GACGCAGCAGTTTTTCAGGCTCCAAAATACCTTGTTGCACGGGTGGGGGA

TACAAAGTCACTAAGATGTGAACAGAAGCTGGGCCACGATGCTATGTACTGGTATAAG

CAAGACTCCAAGCAGTCGCTGAAGGTTATGTTTGCCTACAATAACAAGGAGCCGTTTC

TAAATGAGACGGCTTCAGGTCGCTTCTTACCAGAATCTCCTGACAAAGCTCATTTGAA

ACTTCACATCAAGTCCCTGGAGCTCAGCGACTCTGCCGTGTATCTCTGTGCCAGCAGC

CGAGA"

/annotation_group="TRBV: 121,244 -> 121,530"

/modified_by="zhouhao"

/label="TRBV3"

sig_peptide 121531..121569

/Mismatches=0

/%_Identity=100

/Motif="cacagccctgcaaagtcatcgcctccctctacacaaacc"

/annotation_group="23RSS: 121,531 -> 121,569"

/label="23RSS"

mRNA 126247..126297

/%_Identity=100

/Motif="ATGGGCTGCTGGCTGCTGGGCTGTGTGGCCCTCTGTCTCCTGGGAGCAG"

/annotation_group="L-part1: 126,247 -> 126,295"

/modified_by="zhouhao"

/label="L-part1"

mRNA 126402..126413

/created_by="zhouhao"

/label="L-part2"

gene 126414..126700

/%_Identity=100

/Motif="GACACTGGAATTACTCAGGTACCAAAGTTCCTGGTGATGGGAATGAGAGA

TAAGAAGTCTTTGAAGTGCGAACAACAGCTGGGACATAACGCTATGTACTGGTACAAA

CAGAGCCCTCACAAGCCGCTGCAGCTCCTGTTTGTCTGTAGCTACAAGGAACTCACTG

AAAACAATACTGTCCCAGAACGCTTCACGCCCAAACGCCCAGACACCGCTCAGTTACA

CCTGCTCGTGGATGCCCTGGAGCCAGAAGACTCGGCCCTGTACCTCTGTGCCAGCAGC

AAAGA"

/annotation_group="TRBV: 126,414 -> 126,700"

/modified_by="zhouhao"

/label="TRBV4"

sig_peptide 126701..126739

/Mismatches=0

/%_Identity=100

/Motif="cacagccctgcagggccaccgcctccctgtgcagaaacc"

/annotation_group="23RSS: 126,701 -> 126,739"

/label="23RSS"

mRNA 129509..129559

/%_Identity=100

/Motif="ATGGGCTCCAGGCTGCTCTGCGGGGTGGCCCTTCTTCTCCTGGGACCAG"

/annotation_group="L-part1: 129,509 -> 129,557"

/modified_by="zhouhao"

/label="L-part1"

mRNA 129677..129694

/created_by="zhouhao"

/label="L-part2"

gene 129695..129974

/%_Identity=100

/Motif="GATTCTGGAGTCACGCAAACTCCAAGATACCTGGTGAAACCCAGAGGACA

GACGGTGACACTGAGATGTTCCCCGATCTCTGGACACACCGCCATCTATTGGTATCAA

CTGGCCCCGGGGCCAGGGTCCCAAGTTCTTCATTCAGTATTACAGAGGGACAGCAGGA

GCCAAAGGAGACATGCCTAACCGATTCTCGGGGAAACAGTTCGATAACAACAGCTCTG

AGCTCACCGCGAGCTCTCTGGAGCTGAAGGACTCGGCCCTGTATCTCTGCGCCAGCGG

CTCAG"

/annotation_group="TRBV: 129,688 -> 129,974"

/modified_by="zhouhao"

/label="TRBV5-1 P"

sig_peptide 129975..130013

/Mismatches=0

/%_Identity=100

/Motif="cacagccctgcaaagtcccctgggttctgtgcacaaacc"

/annotation_group="23RSS: 129,975 -> 130,013"

/label="23RSS"

mRNA 132069..132119

/%_Identity=100

/Motif="ATGGGCCCCAGGCTCCTCTGCTGTGTGGCCCTTTGTCTCCTGGGAGCAG"

/annotation_group="L-part1: 132,069 -> 132,117"

/modified_by="zhouhao"

/label="L-part1"

mRNA 132247..132255

/created_by="zhouhao"

/label="L-part2"

gene 132256..132541

/%_Identity=100

/Motif="GACTCTGGAGTCACGCAGACCCCAAAACACCTGATCAAAGCAAAAAAACA

GCAAGCGACGCTGGGGTGTTCCTACATCTCTGGGCACCGCTACATGTTTTGGTACCAA

CAGGCCTGGGGCCAGGGACCCGTGTTCCTCGTTGAGTACTACAATGGGCAAGAGCGAG

AAAAAGGGAAATTGCCAGATCGATTCTCAGTGAAGCATTTCAGTGACTACCGCTCGGA

GCTGACCGCGAGCTCGCTGGAGCTGAGTGACTCCGGCCTGTATCTCTGTGCCAGCTAC

TCAGC"

/annotation_group="TRBV: 132,256 -> 132,542"

/modified_by="zhouhao"

/label="TRBV5-2"

sig_peptide 132542..132580

/Mismatches=0

/%_Identity=100

/Motif="cacagccctgcaggcgcggcagcctcctgcacagaaaca"

/annotation_group="23RSS: 132,542 -> 132,580"

/label="23RSS"

mRNA 133380..133430

/%_Identity=100

/Motif="ATGAGCGGCGGCCTCCTGTGCTGCGTGGCCTTGTGCCTCCTCCGTGCAG"

/annotation_group="L-part1: 133,380 -> 133,428"

/modified_by="zhouhao"

/label="L-part1"

mRNA 133525..133536

/created_by="zhouhao"

/label="L-part2"

gene 133537..133831

/created_by="zhouhao"

/modified_by="zhouhao"

/label="TRBV6-1"

sig_peptide 133832..133870

/%_Identity=100

/Motif="cacagcgctgcacagccgcctcctctctgcacagaaagg"

/annotation_group="23RSS: 133,832 -> 133,870"

/modified_by="zhouhao"

/label="23RSS"

mRNA 134953..135003

/%_Identity=100

/Motif="ATGGGCACCAGGCTCCTCTGCTGGGCGACCCTCTGTCTCCTGGGGGCCGG

T"

/annotation_group="L-part1: 134,953 -> 135,003"

/modified_by="zhouhao"

/label="L-part1"

mRNA 135090..135101

/created_by="zhouhao"

/label="L-part2"

gene 135102..135391

/%_Identity=100

/Motif="GAGGCTGGAGTCTCCCAGTCCCCCAGTCACATAGTCACCAAGAGGGGACA

GAATGTGACATTTCGGTGTGATCCTATTGCTGGCCACATTAGCCTATACTGGTACCAA

CACGTGCCAGGGCAGGGCCCGAAGTTCCTGGTGTACTTTCAAAACAAGGAACCTCTGG

ACTCCTCCGGGATGTCTAACGATCGCTTCTCTGCTGTGAGACCTGACAGCTCCTACTC

CACTCTGAGCATCCAGCCTGCAGAGCCTGGGGACTCGGCCGTGTACCTCTGTGCCAGC

ACTCCGAC"

/annotation_group="TRBV: 135,102 -> 135,391"

/modified_by="zhouhao"

/label="TRBV7-1"

sig_peptide 135392..135430

/Mismatches=0

/%_Identity=100

/Motif="cacagtgtgtcaccgtcaccgcctccctgctcacaaacc"

/annotation_group="23RSS: 135,392 -> 135,430"

/label="23RSS"

mRNA 136103..136153

/%_Identity=100

/Motif="AGGGGCTCTTGGCTCCTCTGCTGCGTGGACCTGTGTCTGCTGGGTGCAG"

/annotation_group="L-part1: 136,103 -> 136,151"

/modified_by="zhouhao"

/label="L-part1"

mRNA 136256..136267

/created_by="zhouhao"

/label="L-part2"

gene 136268..136554

/%_Identity=100

/Motif="GCTGCTGGAGTCACCCAGTCCCCAAGACACCTCATCAAAGGCCGTGGTGG

GGAGGCTGTTCTGAAATGCCACCCCATCTCTGGACACAGCCGTGTGTCCTGGTACCAG

CAGGCTTGGGGGCAGGAACCCGGGTCCTTCATTCAGTATTAGGAACAGCAGGAGTCCG

GGAAAGGAAACATCTCTGAACGCTTCAAAGGCAAAATGTTCAGTGACTACCGCTCTGA

GCTGGCTATGTGCACCTTGCAGCTGGAGGACTTGGCCTTGACCTCTGGGCCTGCAGCT

TAG"

/annotation_group="TRBV: 136,269 -> 136,553"

/modified_by="zhouhao"

/label="TRBV13-1 P"

sig_peptide 136555..136593

/Mismatches=0

/%_Identity=100

/Motif="cacagccctgcagggtccatggctttctgttctagctca"

/annotation_group="23RSS: 136,555 -> 136,593"

/label="23RSS"

mRNA 140411..140461

/%_Identity=100

/Motif="ATGGACCCCAGGCTCCTCTGCTGTGTGGCCCTTTGTCTCCTGGGAGCAG"

/annotation_group="L-part1: 140,411 -> 140,459"

/modified_by="zhouhao"

/label="L-part1"

mRNA 140592..140597

/created_by="zhouhao"

/label="L-part2"

gene 140598..140884

/%_Identity=100

/Motif="GACTCTGGAGTCACGCAGACCCCAAAACACGTGGTCAAAGCAAGAAAACA

GCAAGTGACACTGAAGTGTTCCTACATCTCTGGACACCTGTCTGTGTATTGGTACAAA

CAGGTTCACGGACAGGGTCCCGATTTCCTCATTCAGTACTACAATGGGCAAGAGCGAG

AAAAAGGGAAGCTGCCTGATCGGTTCTCCGTGAGGCTGCTGGATGGCGACCGCTCCGA

GCTGACCGCGAGCTCGCTGGAGCTGAGCGACTCGGCCCTGTATCTCTGCGCCAGCGGC

CCTG"

/annotation_group="TRBV: 140,598 -> 140,883"

/modified_by="zhouhao"

/label="TRBV5-3"

sig_peptide 140885..140923

/Mismatches=0

/%_Identity=100

/Motif="cacagccctgcaggcgcggcagcctcctgcacagaaaca"

/annotation_group="23RSS: 140,885 -> 140,923"

/label="23RSS"

mRNA 142302..142352

/%_Identity=100

/Motif="ATGAGCGGCGGCCTCCTGTGCTGCGTGGCCTTGTGTCTCCTCCGTGCAG"

/annotation_group="L-part1: 142,302 -> 142,350"

/modified_by="zhouhao"

/label="L-part1"

mRNA 142447..142458

/created_by="zhouhao"

/label="L-part2"

gene 142459..142745

/%_Identity=100

/Motif="ATGGCCGGCGTCACTCAGTCCCCAGCCTCCCAGGTTGTGACAACAGGACA

GACGGTGACCTTGCGGTGTTCCCAGGACTTGAAACACGACGGCATGTACTGGTACCGA

CAGGACCTGGGTCACGGGCTGAGGCTGATCCATTACTCAGTGGGCCCTGGGATCATGG

ACAAAGGAGAGGTCCCCGATGGGTACAGTGTCTCTAGATCAAAACAAGAAGACTTCCC

CCTCACGCTGGAGTCGGCCACCCCCGCCCAGACATCTGTGTACTTCTGCGCCAGCAGT

GAACC"

/annotation_group="TRBV: 142,459 -> 142,745"

/modified_by="zhouhao"

/label="TRBV6-2"

sig_peptide 142746..142784

/Mismatches=0

/%_Identity=100

/Motif="cacagtgctgcatggccgcctcctctctgcacagaaagt"

/annotation_group="23RSS: 142,746 -> 142,784"

/label="23RSS"

mRNA 143861..143911

/%_Identity=100

/Motif="ATGAGCACCAGGCTCCTCTGCTGGGTGACCCTCTGTCTCCTGGGGGCCGG

T"

/annotation_group="L-part1: 143,861 -> 143,911"

/modified_by="zhouhao"

/label="L-part1"

mRNA 144022..144033

/created_by="zhouhao"

/label="L-part2"

gene 144034..144323

/%_Identity=100

/Motif="GAGGCTGGAGTCTCCCAGTCCCCCAGGCACAAGGTCACCAAGAGGGGACA

GAATGTGACATTTCAGTGTGATCCAGTTTCTGGACACACTGTTCTTTACTGGTACCGA

CAGACACTGGGGCAGAGTCCGGAGCTATTGGTGTACTTCCAAGGCAAGGACCCTGTAG

ACACCTCCGGGATGCCTAAGGATCGGTTCTTCATTGTGAGACCTGACGGCACCTCCTC

CACTCTGAGCATCCAGCCTGCAGAGCCTGGGGACTCGGCCATGTACCTCTGTGCCAGC

AGTGAAAC"

/annotation_group="TRBV: 144,034 -> 144,323"

/modified_by="zhouhao"

/label="TRBV7-2"

sig_peptide 144324..144362

/Mismatches=0

/%_Identity=100

/Motif="cacagtgtggcaccgtccccaactgcctgctcacaaacc"

/annotation_group="23RSS: 144,324 -> 144,362"

/label="23RSS"

mRNA 147231..147236

/created_by="zhouhao"

/label="L-part2"

gene 147237..147527

/created_by="zhouhao"

/modified_by="zhouhao"

/label="TRBVU1 P"

sig_peptide 147529..147567

/%_Identity=100

/Motif="cacagtgattttgctgactcggccacgcacagaatgaaa"

/annotation_group="23RSS23RSS: 147,529 -> 147,567"

/modified_by="zhouhao"

/label="23RSS"

mRNA 152541..152591

/created_by="zhouhao"

/label="L-part1"

mRNA 152725..152736

/created_by="zhouhao"

/modified_by="zhouhao"

/label="L-part2"

gene 152737..153023

/%_Identity=100

/Motif="CGTGTGTACTGGTACCAACAGGTTCTGGGGCAGGAACTCAGCTTTCTCAT

TCGGTATTATGAACAGCAGGAGTATGGGAAAGAAAATTTCCCCAACCGCTTCAAAGGA

AAGCAGTTCAGAGACTACAGCTCCGAGCTGACCATGCACACCTTGCAGCTGGGGGACT

CGGCCGTGTACCTCTGTGCCAGCAGCTTAGA"

/annotation_group="TRBV: 152,827 -> 153,023"

/modified_by="zhouhao"

/label="TRBV13-2"

sig_peptide 153024..153062

/Mismatches=0

/%_Identity=100

/Motif="cacagccctgcagggcccatgcctttctgtacccaaacc"

/annotation_group="23RSS: 153,024 -> 153,062"

/label="23RSS"

mRNA 154769..154819

/Mismatches=0

/%_Identity=100

/Motif="ATGGTCAGCAGGGTCTGCTTCCGTGTGGCTCTTTGTCTCCTGTGGGCAGG

T"

/annotation_group="L-part1: 154,769 -> 154,819"

/label="L-part1"

mRNA 154769..154819

/%_Identity=100

/Motif="ATGGTCAGCAGGGTCTGCTTCCGTGTGGCTCTTTGTCTCCTGTGGGCAG"

/annotation_group="L-part1: 154,769 -> 154,817"

/modified_by="zhouhao"

/label="L-part1"

mRNA 154922..154936

/created_by="zhouhao"

/label="L-part2"

gene 154937..155215

/%_Identity=100

/Motif="GGAATCACCCAGAGTCCAAGGTACAAAGTCACAGGGACAGGAAAAAGGTG

ACACTGAGGTGTCACCAGACTGATAACCATAACGCTATGTCCTGGCATCGACAAAACC

TGGGCCATGGCCTCAGGCAGATATATTACTCAGGGGGTGTTGGGGTCGCCAGCAAAGG

AGAGGCTCCCGATGGGTACAGTGCATCTAGAGTGAACATGGAGGACTTCCTGCTCACG

CTGGAGTCGGCCACCCCCTCCCAGACATCTGTGTACTTCTGTGCCAGCAGAGAC"

/annotation_group="TRBV6-2: 154,929 -> 155,206"

/modified_by="zhouhao"

/label="TRBV6-3 "

sig_peptide 155216..155254

/Mismatches=0

/%_Identity=100

/Motif="cacatcgctgcacggccaccctctctctgcacagaaagg"

/annotation_group="23RSS: 155,216 -> 155,254"

/label="23RSS"

mRNA 155519..155572

/%_Identity=100

/Motif="ATGCCTCCAGGGCGGGGAGTCATGAACACCATATCCACGTAGATCCCTGC

GAGT"

/annotation_group="L-part1: 155,519 -> 155,572"

/modified_by="zhouhao"

/label="L-part1"

mRNA 155759..155773

/created_by="zhouhao"

/modified_by="zhouhao"

/label="L-part2"

gene 155774..156060

/created_by="zhouhao"

/modified_by="zhouhao"

/label="TRBV9-1 P"

sig_peptide 156061..156099

/%_Identity=100

/Motif="cacagagtggccccgtcaccccttcatgcgcacaccctc"

/annotation_group="23RSS23RSS: 156,061 -> 156,099"

/modified_by="zhouhao"

/label="23RSS"

mRNA 158988..159038

/%_Identity=100

/Motif="ATGGGCCCCAGGCTCCTCTGCTGTGTGGCCCTTTGTCTCCTGGGAGCAG"

/annotation_group="L-part1: 158,988 -> 159,036"

/modified_by="zhouhao"

/label="L-part1"

mRNA 159169..159174

/created_by="zhouhao"

/label="L-part2"

gene 159175..159461

/created_by="zhouhao"

/modified_by="zhouhao"

/label="TRBV5-4"

sig_peptide 159462..159500

/%_Identity=100

/Motif="cacagccctgcaggcgcggcagcctcctgcacagaaaca"

/annotation_group="23RSS: 159,462 -> 159,500"

/modified_by="zhouhao"

/label="23RSS"

mRNA 161074..161124

/%_Identity=100

/Motif="ATGAACGGCAGCCTCTTGTGCTGCATGACCTTGTGTCTCCTCCGTGCAGG

T"

/annotation_group="L-part1: 161,074 -> 161,124"

/modified_by="zhouhao"

/label="L-part1"

mRNA 161219..161230

/created_by="zhouhao"

/label="L-part2"

gene 161231..161517

/%_Identity=100

/Motif="ATGGCCGGGGTCACTCAGTCCCCAACATTCCAGGTTGTGACAAAAGGACA

GATGGTGACCTTGAGGTGTTCCCAGGACTTGAACCATAACAACATGTACTGGTACCGA

CAGGACCTGGGTCACGGGCTGAGGTTGATCCATTACTCAGTGGGTGTTGGGGTCAAGG

ACAAAGGAGAGGTCCCGGATGGGTACATCGTCTCTAGATCAAAACAAGAAGACTTCCC

CCTCACGCTGGAGTCGGCCACCCCCGCCCAGACATCTGTGTACTTCTGTGCCAGCAGT

GAACC"

/annotation_group="TRBV: 161,231 -> 161,517"

/modified_by="zhouhao"

/label="TRBV6-4"

sig_peptide 161518..161556

/Mismatches=0

/%_Identity=100

/Motif="cacagtgctgcacggccgcctcctctctgcacagaaagt"

/annotation_group="23RSS: 161,518 -> 161,556"

/label="23RSS"

mRNA 162658..162708

/%_Identity=100

/Motif="ATGGGCACCAGGCTCCTCTGCTGGGTGACCCTCTGTCTCCTGGGGGCCGG

T"

/annotation_group="L-part1: 162,658 -> 162,708"

/modified_by="zhouhao"

/label="L-part1"

mRNA 162794..162805

/created_by="zhouhao"

/modified_by="zhouhao"

/label="L-part2"

gene 162806..163095

/%_Identity=100

/Motif="GAGGCTGGAGTCTCTCAGTTCCCCAGGCACAAGGTCACCAAGAGGGGACA

GAATGTGACATTTCAGTGTGATCCAATTTCTGGACACACTGTTCTTTACTGGTACCGA

CAGACACTGGGGCAGGGTCCGGAGTTTTTGGTGTACTTCCAAGGCAAGGACCCTGTGG

ACACCTCCGTGATGCCTAAGGATCGGTTCTTCATTGTGAGACCTGACGGCTCCTCCTC

CACTCTGAGCATCCAGCCTGCAGAGCCTGGGGACTCGGCTGTGTACCTCTGTGCCAGC

AGTGCAAC"

/annotation_group="TRBV: 162,806 -> 163,095"

/modified_by="zhouhao"

/label="TRBV7-3"

sig_peptide 163096..163134

/Mismatches=0

/%_Identity=100

/Motif="cacagtgtggcaccgtccccaactgcctgctcacaaacc"

/annotation_group="23RSS: 163,096 -> 163,134"

/label="23RSS"

mRNA 171306..171356

/%_Identity=100

/Motif="ATGGCCCGTCTGGTCCTGCCTGAATCATCCAGGGGCACCCAGCTTCTCTG

TT"

/annotation_group="L-part1: 171,306 -> 171,357"

/modified_by="zhouhao"

/label="L-part1"

mRNA 171490..171501

/created_by="zhouhao"

/label="L-part2"

gene 171502..171788

/%_Identity=100

/Motif="GCTGCTGGAGTCACCCAGTCCCCAAGACACCTCATCAAACGCCGTGGTGG

GGAGGCTGTTCTGAAATGCCACCCCATCTCTGGACACAACCGTGTGTTCTGGTACCAG

CAGGATTGGGGGCAGGAACCCAGGTTTCTCATTGAGTATTATGAAAAGCGCGAGGTCA

GTAAAGGAAACTTCCCAGACCGCTTCAAAGGAAAGCAGCTCAGAGACTACAGCTCCGA

GCTGGCCATGCACACCTTGCAGCTGGGGGACTCGGCCGTGTACCTCTGTGCCAGCAGC

TTAG"

/annotation_group="TRBV: 171,502 -> 171,787"

/modified_by="zhouhao"

/label="TRBV13-3"

sig_peptide 171789..171827

/Mismatches=0

/%_Identity=100

/Motif="cacagccctgcagggcccatgcctttctgtacccaaacc"

/annotation_group="23RSS: 171,789 -> 171,827"

/label="23RSS"

mRNA 173531..173581

/Mismatches=0

/%_Identity=100

/Motif="ATGGTCAGCAGGGTCTGCTTCCGTGTGGCTCTTTGTCTCCTGTGGGCAGG

T"

/annotation_group="L-part1: 173,531 -> 173,581"

/label="L-part1"

mRNA 173531..173581

/%_Identity=100

/Motif="atggtcagcagggtctgcttccgtgtggctctttgtctcctgtgggcag"

/annotation_group="L-part1: 173,531 -> 173,579"

/modified_by="zhouhao"

/label="L-part1"

mRNA 173676..173684

/created_by="zhouhao"

/modified_by="zhouhao"

/label="L-part2"

gene 173685..173978

/%_Identity=100

/Motif="GATGCAGGAATCACCCAGAGTCCAAGGTACAAGGTCACAGGGACAGGACA

AAAGGTGACACTGAGGTGTCACCAGACTGATAACCATAACGCTATGTCCTGGCATCGA

CAAAACCTGGGCCATGGGCTGAGGCAGATATATTACTCCGGGGGTGTTGGGTTCTCCA

GCAAAGGAGAGGAT"

/annotation_group="23RSS23RSS: 173,685 -> 173,864"

/modified_by="zhouhao"

/label="TRBV6-5"

sig_peptide 173979..174017

/%_Identity=100

/Motif="cacagcgctgcacggccaccttctctctgcacaaaaatg"

/annotation_group="23RSS23RSS: 173,979 -> 174,017"

/modified_by="zhouhao"

/label="23RSS"

mRNA 174524..174535

/created_by="zhouhao"

/label="L-part2"

gene 174536..174825

/created_by="zhouhao"

/modified_by="zhouhao"

/label="TRBV9-2 P"

sig_peptide 174826..174864

/%_Identity=100

/Motif="cacagggtgaccccgtcaccccttcatgcgcacacaccc"

/annotation_group="23RSS23RSS: 174,826 -> 174,864"

/modified_by="zhouhao"

/label="23RSS"

mRNA 177778..177828

/%_Identity=100

/Motif="ATGGGCCCCAGGCTCCTCTGCTGTGTGGCCCTTTGTCTCCTGGGAGCAG"

/annotation_group="L-part1: 177,778 -> 177,826"

/modified_by="zhouhao"

/label="L-part1"

mRNA 177959..177964

/created_by="zhouhao"

/label="L-part2"

gene 177965..178251

/%_Identity=100

/Motif="GACTCTGGAGTCACACAGACCCCCAAACACATGGTCACAGCAAGAAAACA

GCAAGTGACGCTGGGGTGTTCCTACATCTCTGGACACCTCTCTGTGTATTGGTACAAA

CAGGTTCAGGGTCAGGGCCCTGAGTTCCTGGTTGAGTACTACAATGGGGAAGAGGGAG

ACAAAGGGAACCTGCCTGATCGGCTCTCCGTGAAGCCGCTGGATGGCTACCGCTCCAA

GCTGACCGCGAGCTCGCTGGAGCTGAGCGACTCGGCCCTGTATCTCTGTGCCAGCAGC

CCTGA"

/annotation_group="TRBV: 177,965 -> 178,251"

/modified_by="zhouhao"

/label="TRBV5-5"

sig_peptide 178252..178290

/Mismatches=0

/%_Identity=100

/Motif="cacagccctgcaggcgcggcagcctcctgcacagaaaca"

/annotation_group="23RSS: 178,252 -> 178,290"

/label="23RSS"

mRNA 179859..179909

/%_Identity=100

/Motif="ATGAACCGCAGTCTCTTGTGCTGCGTGACCTTGTGTCTCCTCCGTGCAGG

T"

/annotation_group="L-part1: 179,859 -> 179,909"

/modified_by="zhouhao"

/label="L-part1"

mRNA 180004..180015

/created_by="zhouhao"

/label="L-part2"

gene 180016..180302

/%_Identity=100

/Motif="ATGGCCGGGGTCACTCAGACCCCCACATTCCAGGTTGTGACAACAGGACA

GACGGTGACCTTGAGGTGTTCCCAGGACTTGAACCACAACGCTATGTACTGGTACCGA

CAGGACCTCGGTCACGGGCTGAAGCTGATCCATTATTCAGCGGGTGTTGGGTTCAAGG

ACAAAGGAGAGGTCCCCGATGGGTACAGCGTCTCTAGATCAAAAAAAGAAGACTTCCC

CCTCACGCTGGAGTCGGCCACCCCCGCCCAGACATCTGTGTACTTCTGTGCCAGCAGT

GAA"

/annotation_group="TRBV: 180,016 -> 180,300"

/modified_by="zhouhao"

/label="TRBV6-6"

sig_peptide 180303..180341

/Mismatches=0

/%_Identity=100

/Motif="cacagtgctgcacggccgcctcctctctgcacagaaagt"

/annotation_group="23RSS: 180,303 -> 180,341"

/label="23RSS"

mRNA 181442..181492

/%_Identity=100

/Motif="ATGGACACCAGGCTCCTCTGCTGGGTGACCCTCTGTCTCCTGGAGGCCGG

T"

/annotation_group="L-part1: 181,442 -> 181,492"

/modified_by="zhouhao"

/label="L-part1"

mRNA 181579..181590

/created_by="zhouhao"

/label="L-part2"

gene 181591..181880

/%_Identity=100

/Motif="GAGGCTGGAGTCTCCCAGTCCCCCAGGCACAAGGTCACCAAGAGGGGACA

GAATGTGACATTTCAGTGTGATCCAATTTCTGGACACACTGTTCTTTACTGGTACCGA

CAGACACTGGGGCAGGGCCCGGAGCTGTTGGTGTACTTCCAAGGCAAGGAACCTGTGG

ACACCTCGGTGATGCCTAAGGATCGGTTCTTCATTGTGAGACCTGACGGCTCCTCCTC

CACTCTGAGCATCCAGCCTGCAGAGCCTGGGGACTCGGCCGTGTACCTCTGTGCCAGC

AGTGCAAC"

/annotation_group="TRBV: 181,591 -> 181,880"

/modified_by="zhouhao"

/label="TRBV7-4"

sig_peptide 181881..181919

/Mismatches=0

/%_Identity=100

/Motif="cacagtgtggcaccgtacccaactgcctgctcacacacc"

/annotation_group="23RSS: 181,881 -> 181,919"

/label="23RSS"

mRNA 188398..188478

/%_Identity=100

/Motif="ATGGCCCGCCTGGTCCTGCCTGAATCCTCCAGGGGCACCCGGCTCCTCTG

CTGCATGGTCCTGTGTCTGCTGGGACCAGGT"

/annotation_group="L-part1: 188,398 -> 188,478"

/modified_by="zhouhao"

/label="L-part1"

mRNA 188582..188593

/created_by="zhouhao"

/label="L-part2"

gene 188594..188880

/%_Identity=100

/Motif="GCTGCTGGAGTCACCCAGTCCCCAAGACACCTCATCAAACGCCATGGTAG

GGAGGCTGTTCTGAAATGCCACCCCATCTCTGGACACAAGAGTGTTTACTGGTACCGA

CAGGCTCGGGGGCAGGAACCCAGGTTTCTCATTGAGTATTATGAACAGCAGGAGTCAG

GGAAAGGAAAATTCCCCGACCACTTCAAAGGAAAGCAGTTCAGAGACTACAGCTCCGA

GCTGACCATGCACACCTTGCAGCTGGAGGACTCGGCCGTGTACCTCTGTGCCAGCAGC

TTAG"

/annotation_group="TRBV: 188,594 -> 188,879"

/modified_by="zhouhao"

/label="TRBV13-4"

sig_peptide 188881..188919

/Mismatches=0

/%_Identity=100

/Motif="cacagccctgcagggcccatgcctttctgtacccaaacc"

/annotation_group="23RSS: 188,881 -> 188,919"

/label="23RSS"

mRNA 190607..190657

/%_Identity=100

/Motif="ATGGTCAGCAGGGTCTGCTTCCGTGTGGCTCTTTGTCTCCTGTGGGCAGG

T"

/annotation_group="L-part1: 190,607 -> 190,657"

/modified_by="zhouhao"

/label="L-part1"

mRNA 190749..190760

/created_by="zhouhao"

/label="L-part2"

gene 190761..191054

/%_Identity=100

/Motif="GAAGCAGGAATCACCCAGAGTCCAAGGTACAAGGTCACAGGGACAGGAAA

AATGGTGACACTGAGGTGTCACCAGACTGATAACCATAACGCTATGTCCTGGCATCGA

AAAGTCCTGGGCCATGGGCTCAGGCAGATATATCACTCATTGGGTGTTGGGGTCGCCA

GCAAAGGAGAGGATGCCGACGGGCACAGTGCGTCTAGAGTGAACATGGAGGACTTCCT

GCTCACGCTGGAGTCGGCCACCCCCTCCCAGACATCTGTGTACTTCTGCACCAGCAGA

GACGTGCTGATC"

/annotation_group="TRBV: 190,761 -> 191,054"

/modified_by="zhouhao"

/label="TRBV6-7"

sig_peptide 191055..191093

/%_Identity=100

/Motif="CACAGCGCTGCACGGCCACCTTCTCTCTGCACAGAAAGG"

/annotation_group="23RSS: 191,055 -> 191,093"

/modified_by="zhouhao"

/label="23RSS"

mRNA 191592..191606

/created_by="zhouhao"

/label="L-part2"

gene 191607..191896

/created_by="zhouhao"

/modified_by="zhouhao"

/label="TRBV9-3 P"

sig_peptide 191897..191935

/%_Identity=100

/Motif="cacagggtgaccccgtcaccccttcatgtgaacatgccc"

/annotation_group="23RSS23RSS: 191,897 -> 191,935"

/modified_by="zhouhao"

/label="23RSS"

mRNA 196678..196726

/%_Identity=100

/Motif="ATGGGCCCCAGGCTCCTCTGCTGTGTGGCCCTTTGTCTCCTGGGAGCAG"

/annotation_group="L-part1: 196,678 -> 196,726"

/modified_by="zhouhao"

/label="L-part1"

mRNA 196859..196864

/created_by="zhouhao"

/label="L-part2"

gene 196865..197151

/created_by="zhouhao"

/modified_by="zhouhao"

/label="TRBV5-6"

sig_peptide 197152..197190

/Mismatches=0

/%_Identity=100

/Motif="cacagccctgcaggcgcggcagcctcctgcacagaaaca"

/annotation_group="23RSS: 197,152 -> 197,190"

/label="23RSS"

mRNA 198577..198627

/%_Identity=100

/Motif="ATGAACGGCAGCCTCTTGTGCTGCGTGACCTTGTGTCTCCTCCGAGCAGG

T"

/annotation_group="L-part1: 198,577 -> 198,627"

/modified_by="zhouhao"

/label="L-part1"

mRNA 198722..198733

/created_by="zhouhao"

/label="L-part2"

gene 198734..199020

/%_Identity=100

/Motif="ATGGCCGGCGTCACTCAGTCCCCAACCTCCCAGGTTGTGACAACAGGACA

GACGGTGACCTTGCAGTGTTCCCAGGACTTGAACCATAACTACATGTACTGGTACCGA

CAGGACCTGGGTCACGGGCTGAGGCTGATCCATTACTCAGTGGGTGTTGGGGTCAAGG

ACAAAGGAGAGGTCCCGGATGGGTACAGCGTCTCTAGATCAAAACAAGAAGACTTCCC

CCTCACGCTGGAGTCGGCCACCCCCTCCCAGACATCTGTGTACTTCTGCGCCAGCAGT

GAACC"

/annotation_group="TRBV: 198,734 -> 199,020"

/modified_by="zhouhao"

/label="TRBV6-8"

sig_peptide 199021..199059

/Mismatches=0

/%_Identity=100

/Motif="cacagtgctgcacggccgcctcctctctgcacagaaagt"

/annotation_group="23RSS: 199,021 -> 199,059"

/label="23RSS"

mRNA 200159..200209

/%_Identity=100

/Motif="ATGGGCACCAGGCTCCTCTGCTGGGCGACCCTCTGTCTCCTGGGGGCCGG

T"

/annotation_group="L-part1: 200,159 -> 200,209"

/modified_by="zhouhao"

/label="L-part1"

mRNA 200314..200325

/created_by="zhouhao"

/label="L-part2"

gene 200326..200618

/created_by="zhouhao"

/modified_by="zhouhao"

/label="TRBV7-5 P"

sig_peptide 200619..200657

/%_Identity=100

/Motif="cacagtgtggcactgtccccaactccctgctcacacacc"

/annotation_group="23RSS: 200,619 -> 200,657"

/modified_by="zhouhao"

/label="23RSS"

mRNA 208836..208916

/%_Identity=100

/Motif="ATGGCCCGTCTGGTCCTGCCTGAATCCTCCAGGGGCACCCGGCTCCTCTG

CTGCATGGTCCTGTGTCTGCTGGGACCAG"

/annotation_group="L-part1: 208,836 -> 208,914"

/modified_by="zhouhao"

/label="L-part1"

mRNA 209020..209031

/created_by="zhouhao"

/label="L-part2"

gene 209032..209318

/%_Identity=100

/Motif="GCTGCTGGAGTCACCCAGTCCCCAAGACACCTCATCAAAGGCCTTGGTGG

GGAGGCTGTTCTGAAATGCCACCCCATCTCAGGACACAACCGTGTGTACTGGTACCAA

CAGGCTCGGGGGCAGGAACCCAGCTTTCTCATTCGGTATTATGAACAGCAGGAGTACG

GGAAAGAAAATTTCCCCGACCACTTCAAAGGAAAGCAGTTCAGAGACTACAGCTCCGA

GCTGACCATGCACACCTTGCAGCTGGGGGACTCGGCCGTGTACCTCTGTGCCAGCAGC

GTAG"

/annotation_group="TRBV: 209,032 -> 209,317"

/modified_by="zhouhao"

/label="TRBV13-5"

sig_peptide 209319..209357

/Mismatches=0

/%_Identity=100

/Motif="cacagccctgcagggcccatgcctttctgtacccaaacc"

/annotation_group="23RSS: 209,319 -> 209,357"

/label="23RSS"

mRNA 211062..211112

/%_Identity=100

/Motif="atggtcagcagggtctgcttccgtgtggctctttgtctcctgtgggcag"

/annotation_group="L-part1: 211,062 -> 211,110"

/modified_by="zhouhao"

/label="L-part1"

mRNA 211204..211215

/created_by="zhouhao"

/modified_by="zhouhao"

/label="L-part2"

gene 211216..211509

/%_Identity=100

/Motif="GATGCAGGAATCACCCAGAGTCCAAAGTACAAGGTCACAGGGACAGGACA

AAAGGTGACACTGAGCTGTCACCAGACTGATAACCACAACGTTATGTCCTGGCATCGA

CAAAACCTAGGCCATGGGCTGAGGCAGATGTATTACTCCTGGGGTGTTGGGTTTTCCA

GCAAAGGAGAGGA"

/annotation_group="TRBV: 211,216 -> 211,394"

/modified_by="zhouhao"

/label="TRBV6-9"

sig_peptide 211510..211548

/%_Identity=100

/Motif="cacagtgctgcatgaccatcttctctctgcacaaaaaca"

/annotation_group="23RSS: 211,510 -> 211,548"

/modified_by="zhouhao"

/label="23RSS"

mRNA 212092..212100

/created_by="zhouhao"

/label="L-part2"

gene 212101..212352

/%_Identity=100

/Motif="AAGCGGGGCCAGGGAGTGGTCCTCAGGTGTGATCCCATATCTGGACACTT

GACATTTACATGTGCTGACAGGTCCTGGGACAGGGCCCAGAGTTTGTGATGTACTTTC

ACCTCCTGGATCCCCAGACTCCTGCGGGAAGCCTAGGACCGGTTCTCTGCTCAGAGGC

CAGGGGGATCCCACTGCACTCTGAGGAGCCAGCCTGCAGAGCTGGGGGACTCGGCCGT

GCCCCTCTGTGCCAGCAGCTCCAGCACAGGGTGGTCCTGTCACCCCTTCATGCGCACA

CACCCTCATCCTTCTCCCTCCATGCAGCTCCCGCACTCTAAGCAGCCCTTCTTTGCTC

ATCATTCACCAGGG"

/annotation_group="TRBV: 212,107 -> 212,460"

/modified_by="zhouhao"

/label="TRBV9-4"

mRNA 215291..215341

/%_Identity=100

/Motif="ATGGGCCCCAGGCTCCTCTGCTGTGTGGCCCTTTGTCTCCTGGGAGCAG"

/annotation_group="L-part1: 215,291 -> 215,339"

/modified_by="zhouhao"

/label="L-part1"

mRNA 215472..215477

/created_by="zhouhao"

/label="L-part2"

gene 215478..215764

/%_Identity=100

/Motif="GACTCTGGAGTCACGCAGACCCCAAAACACGTGGTCACAGCAAGAAAACT

GCAAGTGACGCTGGGGTGTTCCTACATCTCTGGACACCGCTCTGTGTATTGGTACAAA

CAGGTTCAGGGTCAGGGCCCTGAGTTCCTCATTCAGTACTACAATGGGCAAGAGCAAG

AAAAAGGGAACATCCCCGATAGATTCTCCGTGAGGCCGCTGGATGGCGACCGCTCCGA

GCTGACCGCGAGCTCGCTGGAGCTGAGCGACTCGGCCCTGTATCTCTGTGCCAGCAGC

CCTG"

/annotation_group="TRBV: 215,478 -> 215,763"

/modified_by="zhouhao"

/label="TRBV5-7"

sig_peptide 215765..215803

/Mismatches=0

/%_Identity=100

/Motif="cacagccctgcaggcgcggcagcctcctgcacagaaaca"

/annotation_group="23RSS: 215,765 -> 215,803"

/label="23RSS"

mRNA 216342..216392

/%_Identity=100

/Motif="ATGGGCACCAGGCCCCTCTGCTGGGCGACCCTCTGTCTCCTGGGGGCCGG

T"

/annotation_group="L-part1: 216,342 -> 216,392"

/modified_by="zhouhao"

/label="L-part1"

mRNA 216528..216539

/created_by="zhouhao"

/label="L-part2"

gene 216540..216829

/created_by="zhouhao"

/modified_by="zhouhao"

/label="TRBV7-6"

sig_peptide 216830..216868

/%_Identity=100

/Motif="cacagtgtgtcaccgtcaccctctccctgttcacatacc"

/annotation_group="23RSS23RSS: 216,830 -> 216,868"

/modified_by="zhouhao"

/label="23RSS"

mRNA 217519..217599

/created_by="zhouhao"

/modified_by="zhouhao"

/label="L-part1"

mRNA 217703..217714

/created_by="zhouhao"

/label="L-part2"

gene 217715..218001

/%_Identity=100

/Motif="GCTGCTGGAGTCACCCAGTCCCCAAGACACCTCATCAAAGGCCCTGGTGG

GGGGGCCGTTCTGAAATGCCACCCCATCTCTGGACACAAGAGTGTTTACTGGTACCAG

CAGGCTCAAGGGCAGGGACCTAGGTTTCTCATTCAGTATTATGAAAAGCAGGAGTACG

CGAAAGGAGACATCCCAGATCACTTCTCAGTACAAGTGTTCAGTGACTACAGCTCCGA

GCTGACCATGCACACCTTGCAGCTGGGAGACTCGGCCGTGTACCTCTGTGCCAGCAGC

CTAGA"

/annotation_group="TRBV: 217,715 -> 218,001"

/modified_by="zhouhao"

/label="TRBV13-6"

sig_peptide 218002..218040

/Mismatches=0

/%_Identity=100

/Motif="cacagccctgcagggcccatgcctttctgtacccaaacc"

/annotation_group="23RSS: 218,002 -> 218,040"

/label="23RSS"

mRNA 219724..219774

/%_Identity=100

/Motif="ATGGTCACCAGGCTCTTCTTCTGCGCAGCTGTTTGTCTCCTGTGGGCAGG

T"

/annotation_group="L-part1: 219,724 -> 219,774"

/modified_by="zhouhao"

/label="L-part1"

mRNA 219867..219878

/created_by="zhouhao"

/label="L-part2"

gene 219879..220165

/%_Identity=100

/Motif="GATGCCAAAGTTACCCAGAGTCCAAGAAACAAGGTCACAGTGACAGGAAA

GAAGGTGATACTGAGTTGCAACCAGGCTTATGACCACAACTATATGTACTGGTATCGA

CAAGATCCAGGTCACGGGCTGAGGCTGATCTATTATTCATATGGTATTGGCAGCATTG

ACAAAGGAGAGGTCCCTGATGGGTACAGCGTCTCTAGATCAGATATAAAGAATTTCCT

CCTCACGCTGGAGTCGGCCACCCCCGCCCAGACATCTGTGTACTTCTGCGCCAGCAGT

GAATC"

/annotation_group="TRBV: 219,879 -> 220,165"

/modified_by="zhouhao"

/label="TRBV6-10"

sig_peptide 220166..220204

/Mismatches=0

/%_Identity=100

/Motif="cacagtgctgcatggccacctcctctctgcacagaaagt"

/annotation_group="23RSS: 220,166 -> 220,204"

/label="23RSS"

mRNA 222314..222364

/%_Identity=100

/Motif="ATGGGCACCAGGCTCCTCTGCTGGGCGACCCTCTGTCTGCTGGGGGCCGG

T"

/annotation_group="L-part1: 222,314 -> 222,364"

/modified_by="zhouhao"

/label="L-part1"

mRNA 222450..222461

/created_by="zhouhao"

/label="L-part2"

gene 222462..222751

/%_Identity=100

/Motif="GAAGGTGGCGTCACCCAGACCCCCAGACATAAGATCCTAGGAAAGAGTCA

GGCGGCCACTTTGTGGTGTAATCCCATTTCGGGCCACCAAACCCTTTACTGGTACCAG

CAGTTCCAGGGACAGGGCCCACGGCTTTTGGTCCGCTTTGAGAATGAAGAAGCAGTGG

ATGACTCTCAGCTGTCTAAGGATCGATTTTTTGCACTGAGGCCCAAAGGAGCAGACTC

AACTCTGAAGATCCTGCGTGCAGAGCGGGGGGACTCGGCCGTGTATTTCTGCGCCAGC

AGCTTAGC"

/annotation_group="TRBV: 222,462 -> 222,751"

/modified_by="zhouhao"

/label="TRBV11"

sig_peptide 222752..222790

/Mismatches=0

/%_Identity=100

/Motif="cacagcgtcacagagacgcgtccttcctgtgcacaaacc"

/annotation_group="23RSS: 222,752 -> 222,790"

/label="23RSS"

mRNA 228569..228619

/%_Identity=100

/Motif="CTGGGAACCTGGGACCTCTGCTGAGTGGGCCTCTGGCTGCTGGGAGTAGG

T"

/annotation_group="L-part1: 228,569 -> 228,619"

/modified_by="zhouhao"

/label="L-part1"

mRNA 228711..228722

/created_by="zhouhao"

/label="L-part2"

gene 228723..229012

/created_by="zhouhao"

/modified_by="zhouhao"

/label="TRBV12-1 P"

sig_peptide 229013..229051

/%_Identity=100

/Motif="cacagcgctgcacaggcaccccctccctgggcagaagcc"

/annotation_group="23RSS23RSS: 229,013 -> 229,051"

/modified_by="zhouhao"

/label="23RSS"

mRNA 232209..232259

/%_Identity=100

/Motif="ATGGCCACCAGGCTCCTCTGCTGGGTGGCCCTTTGTGTCCTGGGAGTAGG

T"

/annotation_group="L-part1: 232,209 -> 232,259"

/modified_by="zhouhao"

/label="L-part1"

mRNA 232355..232366

/created_by="zhouhao"

/label="L-part2"

gene 232367..232656

/created_by="zhouhao"

/modified_by="zhouhao"

/label="TRBV12-2"

sig_peptide 232657..232695

/%_Identity=100

/Motif="cacagcgctgcacagtcgccccctccctgtgcagaaacc"

/annotation_group="23RSS: 232,657 -> 232,695"

/modified_by="zhouhao"

/label="23RSS"

mRNA 235158..235208

/%_Identity=100

/Motif="ATGGCCACCAGGCTCCTGGGCTGGGTGGCCCTTTGTGTCCTGGGCATAGG

T"

/annotation_group="L-part1: 235,158 -> 235,208"

/modified_by="zhouhao"

/label="L-part1"

mRNA 235304..235315

/created_by="zhouhao"

/label="L-part2"

gene 235316..235605

/%_Identity=100

/Motif="TATGCTGGAGTCACCCAGACACCCAGACACAAGGTGACAAAGAGGGGACA

AGGAGTGACTCTGGGTTGTGAGCCAATTTCAAGCCATACCTACCTTTACTGGTACAGA

CAGACCTCAGTGAGGGGACTGGAGTTTATGGTTTATTTCAGCAGTGAAACTATTGTAG

ACGACACTGGGATGTCCAAGGACCGATTCTTAGCTCACATGCCTAATGGGTCATACTC

CACTCTGACGATCCAGCCCACAGAACCCGGGGACTCAGCCGTGTACCTCTGTGCCAGC

AGCTTAGC"

/annotation_group="TRBV: 235,316 -> 235,605"

/modified_by="zhouhao"

/label="TRBV12-3"

sig_peptide 235606..235644

/Mismatches=0

/%_Identity=100

/Motif="cacagcgctgcacagttgccccctccctgtgcagaaacc"

/annotation_group="23RSS: 235,606 -> 235,644"

/label="23RSS"

mRNA 237227..237275

/%_Identity=100

/Motif="ATGGCCACCAGGCTCCTGTGCTGGGCAGCCCTTTGTCTCCTGGGAGTAG"

/annotation_group="L-part1: 237,227 -> 237,275"

/modified_by="zhouhao"

/label="L-part1"

mRNA 237372..237383

/created_by="zhouhao"

/label="L-part2"

gene 237384..237673

/%_Identity=100

/Motif="GATGCTGGTGTCACCCAGACACCCAGGCACAAGGTGACAAAGAGGGGACA

AGAAGTAACTCTGAGTTGTGAACCAATTTCAGGCCATGCTGGTCTCTTCTGGTACAGC

CAGACACCAGGGCAGGGCATAAAGTTCCTTATTTACTTCAACAATCAAAGTCCTGTGG

ATGACACAGGGATGTCCAAGGAGCGATTTTCAGCTCAGATGCCTAATAAGTCATTCTC

CACTCTGACGATCCAGCGCACAGAACCCGGGGACTCAGCCGTGTACCTCTGTGCCAGC

AGCTTAGC"

/annotation_group="TRBV: 237,384 -> 237,673"

/modified_by="zhouhao"

/label="TRBV12-4"

sig_peptide 237674..237712

/Mismatches=0

/%_Identity=100

/Motif="cacagcgctgcacagtcaccccgtccctgtgcagaaacc"

/annotation_group="23RSS: 237,674 -> 237,712"

/label="23RSS"

mRNA 239955..240005

/%_Identity=100

/Motif="ATGGCCACCAGGCTCCTCTGCTGGGTGGCCCTTTGTCTCCTGGGACTAGG

T"

/annotation_group="L-part1: 239,955 -> 240,005"

/modified_by="zhouhao"

/label="L-part1"

mRNA 240102..240113

/created_by="zhouhao"

/label="L-part2"

gene 240114..240403

/%_Identity=100

/Motif="CATGCTGGAGTCACCCAGACACGAAGGAACAAGGTGACAACAAAAGGACA

AAGAGTGACTCTGGGCTGTAAGCCAATTTCAGGCCATGATGGTCTTTACTGGTACAGA

CAGAGCTCAGGGCAAGGAATGGAGTTTCTGATTTCCTTCAGCTATGCAAAACCTCTGG

ATGACACAGGACTGCCCAAGGAGCGATTCTCAGCTCACATGCCTAATAGGTCCTTCTC

CACTCTGACGATCCAGCCCACAGAACCCAGGGACTCAGCCGTGTACCTCTGTGCCAGC

AGCTTAGC"

/annotation_group="TRBV: 240,114 -> 240,403"

/modified_by="zhouhao"

/label="TRBV12-5"

sig_peptide 240404..240442

/Mismatches=0

/%_Identity=100

/Motif="cacagcgctgcacagtcgccccctccctgtgcagaaacc"

/annotation_group="23RSS: 240,404 -> 240,442"

/label="23RSS"

mRNA 242067..242117

/%_Identity=100

/Motif="ATGGCCACCATGCTCCTGTGCTGGGTAGCCCTTTGTCTCCTGGGAGTAGG

T"

/annotation_group="L-part1: 242,067 -> 242,117"

/modified_by="zhouhao"

/label="L-part1"

mRNA 242212..242223

/created_by="zhouhao"

/modified_by="zhouhao"

/label="L-part2"

gene 242224..242527

/created_by="zhouhao"

/modified_by="zhouhao"

/label="TRBV12-6"

sig_peptide 242528..242566

/%_Identity=100

/Motif="cacagtgcagcacagtcaccccctccctgtacagaaacc"

/annotation_group="23RSS23RSS: 242,528 -> 242,566"

/modified_by="zhouhao"

/label="23RSS"

mRNA 244847..244897

/%_Identity=100

/Motif="ATGGCCACCAGGCTCCTCTGCTGGGTGGCCCTTTGTCTCCTGGGCATAG"

/annotation_group="L-part1: 244,847 -> 244,895"

/modified_by="zhouhao"

/label="L-part1"

mRNA 244993..245004

/created_by="zhouhao"

/label="L-part2"

gene 245005..245294

/created_by="zhouhao"

/modified_by="zhouhao"

/label="TRBV12-7"

sig_peptide 245295..245333

/%_Identity=100

/Motif="cacagcgctacacagtcaccccctccctgtgcagaaacc"

/annotation_group="23RSS: 245,295 -> 245,333"

/modified_by="zhouhao"

/label="23RSS"

mRNA 246908..246958

/%_Identity=100

/Motif="atggccaccaggctcctctgctgggtggccttttgtctcctgggcatag"

/annotation_group="L-part1: 246,908 -> 246,956"

/modified_by="zhouhao"

/label="L-part1"

mRNA 247064..247078

/created_by="zhouhao"

/label="L-part2"

gene 247079..247353

/created_by="zhouhao"

/modified_by="zhouhao"

/label="TRBV12-8 P"

sig_peptide 247354..247392

/%_Identity=100

/Motif="cacagcgctgcacagtcaccccctccctgtgcagaaacc"

/annotation_group="23RSS: 247,354 -> 247,392"

/modified_by="zhouhao"

/label="23RSS"

mRNA 248987..249037

/%_Identity=100

/Motif="ATGGCCACCAGGCTCCTGTGCTGGGTGGCCCTTTGTCTCTTGAGCAGAGG

T"

/annotation_group="L-part1: 248,987 -> 249,037"

/modified_by="zhouhao"

/label="L-part1"

mRNA 249131..249142

/created_by="zhouhao"

/modified_by="zhouhao"

/label="L-part2"

gene 249143..249432

/created_by="zhouhao"

/modified_by="zhouhao"

/label="TRBV12-9"

sig_peptide 249433..249471

/%_Identity=100

/Motif="cacagcactacacagtcgtcccctccctgtgcagaaacc"

/annotation_group="23RSS23RSS: 249,433 -> 249,471"

/modified_by="zhouhao"

/label="23RSS"

mRNA 251066..251116

/%_Identity=100

/Motif="ATGGCCACCAGGTTCCTGAGCTTCCTGGCCCTTTGTCTCCTGGTAATAGG

T"

/annotation_group="L-part1: 251,066 -> 251,116"

/modified_by="zhouhao"

/label="L-part1"

mRNA 251211..251222

/created_by="zhouhao"

/modified_by="zhouhao"

/label="L-part2"

gene 251223..251512

/created_by="zhouhao"

/modified_by="zhouhao"

/label="TRBV12-10"

sig_peptide 251513..251551

/%_Identity=100

/Motif="cacagtgctgcacagtcgccccttccctgtgcagaaacc"

/annotation_group="23RSS23RSS: 251,513 -> 251,551"

/modified_by="zhouhao"

/label="23RSS"

mRNA 253846..253896

/Mismatches=0

/%_Identity=100

/Motif="ATGGCCACCAGGCTCCTCTGCTGGGTGGCCCTTTGTCTCCTGGGAGTAGG

T"

/annotation_group="L-part1: 253,846 -> 253,896"

/label="L-part1"

mRNA 253846..253896

/%_Identity=100

/Motif="ATGGCCACCAGGCTCCTCTGCTGGGTGGCCCTTTGTCTCCTGGGAGTAGG

T"

/annotation_group="L-part1: 253,846 -> 253,896"

/modified_by="zhouhao"

/label="L-part1"

mRNA 253991..254002

/created_by="zhouhao"

/label="L-part2"

gene 254003..254292

/created_by="zhouhao"

/modified_by="zhouhao"

/label="TRBV12-11"

sig_peptide 254293..254331

/%_Identity=100

/Motif="cacagcgctgcacagtcgccccctccctgtgcagaaacc"

/annotation_group="23RSS: 254,293 -> 254,331"

/modified_by="zhouhao"

/label="23RSS"

mRNA 255908..255958

/%_Identity=100

/Motif="ATGGCCACCAGGCTCCTCTGCTGGGTGGCCCTTTGTCTCCTGGGGGTAGG

T"

/annotation_group="L-part1: 255,908 -> 255,958"

/modified_by="zhouhao"

/label="L-part1"

mRNA 256053..256064

/created_by="zhouhao"

/label="L-part2"

gene 256065..256354

/%_Identity=100

/Motif="CATGCTGGAGTCACCCAGACACCCAGGCAGAAGGTGACAAAGAGGGGACA

AGATGTGACTATGAGCTGTGAGCCAATTTCAGGCCACCCTGACCTTTTCTGGTACAGG

CAGACATCGGGGCAGGGCATAAAGTTACTTATTTACTTCAGCAATCAAAGTCCGCTGG

ACAACACAGGGATGCCCAAGGAGCGATTCTCAGCTCAGATGCCTAATGAGTCATGCTC

CACTCTGAAGATCCAGCCCACAGAACCCGGGGACTCAGCCGTGTACCTCTGTGCCAGC

AGCTTAGC"

/annotation_group="TRBV: 256,065 -> 256,354"

/modified_by="zhouhao"

/label="TRBV12-12"

sig_peptide 256355..256393

/Mismatches=0

/%_Identity=100

/Motif="cacagcgctacacagtcaccccctccctgtgcagaaacc"

/annotation_group="23RSS: 256,355 -> 256,393"

/label="23RSS"

mRNA 258677..258727

/%_Identity=100

/Motif="atggccatcagtctcctgtgctgggtggccctttgtcttctgggagtag"

/annotation_group="L-part1: 258,677 -> 258,725"

/modified_by="zhouhao"

/label="L-part1"

mRNA 258821..258832

/created_by="zhouhao"

/label="L-part2"

gene 258833..259122

/%_Identity=100

/Motif="CATGCTGGAGTCACCCAGACACCCAGGCACAAGGTGACAACAAGGGGACA

AGAAGTGGCTATGAGTTGTGAGCCAGTCTCAGGCCATGA"

/annotation_group="TRBV: 258,833 -> 258,921"

/modified_by="zhouhao"

/label="TRBV12-13"

sig_peptide 259123..259161

/%_Identity=100

/Motif="cacagcgctgcacagtcgccccctccctgtgcagaaacc"

/annotation_group="23RSS: 259,123 -> 259,161"

/modified_by="zhouhao"

/label="23RSS"

mRNA 261346..261396

/%_Identity=100

/Motif="ATGGTCACCAGGCTCCTCTGCTGGGTGGCCCTTTGTCTCCTGGGAGTAGG

T"

/annotation_group="L-part1: 261,346 -> 261,396"

/modified_by="zhouhao"

/label="L-part1"

mRNA 261490..261501

/created_by="zhouhao"

/label="L-part2"

gene 261502..261791

/created_by="zhouhao"

/modified_by="zhouhao"

/label="TRBV12-14"

sig_peptide 261792..261830

/Mismatches=0

/%_Identity=100

/Motif="cacagcgctgcacagtcgccccctccatgtgcagaaacc"

/annotation_group="23RSS: 261,792 -> 261,830"

/label="23RSS"

mRNA 263457..263507

/%_Identity=100

/Motif="ATGGCCACCAGGCTCCTCTGCTGGGTGGCCCTCTGTCTCCTGAGCAGAGG

T"

/annotation_group="L-part1: 263,457 -> 263,507"

/modified_by="zhouhao"

/label="L-part1"

mRNA 263603..263614

/created_by="zhouhao"

/label="L-part2"

gene 263615..263904

/created_by="zhouhao"

/modified_by="zhouhao"

/label="TRBV12-15"

sig_peptide 263905..263943

/%_Identity=100

/Motif="cacagcgctgcacagtcaccccctccctgtgcagaaacc"

/annotation_group="23RSS: 263,905 -> 263,943"

/modified_by="zhouhao"

/label="23RSS"

mRNA 265537..265587

/%_Identity=100

/Motif="ATGGCCAGCAGGCTCCTCTGCTGGGTGGCCCTTTGTCTCCTGGGAGTAGG

T"

/annotation_group="L-part1: 265,537 -> 265,587"

/modified_by="zhouhao"

/label="L-part1"

mRNA 265682..265693

/created_by="zhouhao"

/label="L-part2"

gene 265694..265983

/created_by="zhouhao"

/modified_by="zhouhao"

/label="TRBV12-16"

sig_peptide 265984..266022

/%_Identity=100

/Motif="cacagtgctgcacagccgccccctccctgtgcagaaacc"

/annotation_group="23RSS23RSS: 265,984 -> 266,022"

/modified_by="zhouhao"

/label="23RSS"

mRNA 267606..267656

/%_Identity=100

/Motif="ATGGCCACCAGGCTCTTGAGCTGGGTGGCCCTTTGTCTCCTGGGAGTAGG

T"

/annotation_group="L-part1: 267,606 -> 267,656"

/modified_by="zhouhao"

/label="L-part1"

mRNA 267752..267763

/created_by="zhouhao"

/modified_by="zhouhao"

/label="L-part2"

gene 267764..268053

/created_by="zhouhao"

/modified_by="zhouhao"

/label="TRBV12-17"

sig_peptide 268054..268092

/%_Identity=100

/Motif="cacagcgctgcacagttgccccctccctgtgcagaaacc"

/annotation_group="23RSS23RSS: 268,054 -> 268,092"

/modified_by="zhouhao"

/label="23RSS"

mRNA 270385..270435

/Mismatches=0

/%_Identity=100

/Motif="ATGGCCACCAGGCTCCTCTGCTGGGTGGCCCTTTGTCTCCTGGGAGTAGG

T"

/annotation_group="L-part1: 270,385 -> 270,435"

/label="L-part1"

mRNA 270385..270435

/%_Identity=100

/Motif="ATGGCCACCAGGCTCCTCTGCTGGGTGGCCCTTTGTCTCCTGGGAGTAGG

T"

/annotation_group="L-part1: 270,385 -> 270,435"

/modified_by="zhouhao"

/label="L-part1"

mRNA 270529..270540

/created_by="zhouhao"

/label="L-part2"

gene 270541..270830

/%_Identity=100

/Motif="CATGCTGGAGTCACCCAGACACCCAGGCACAAGGTGACAAAAAGAGGACA

AGGAGTGACTATGAATTGTGAGCCAATCTCAGGCCATAGCTACGTTTACTGGTACAGA

CAGACCTTGGTGGAAGGCATAACGTTTCTGATTTCCTTCCTCAATCAAAGCCCTCTGG

ACAAGACAAGTCTGCCCAAGGAGCGATTCTCAGCTCAGATGCCTAAAGAGTCGTTGTC

CACTCTGATGATCCAGCGCACAGAACCCGGGGACTCAGCCGTGTACCTCTGTGCCAGC

AGCTTA"

/annotation_group="TRBV: 270,541 -> 270,828"

/modified_by="zhouhao"

/label="TRBV12-18"

sig_peptide 270831..270869

/Mismatches=0

/%_Identity=100

/Motif="cacagcgctgcacagtcgccccctccctgtgcagaaacc"

/annotation_group="23RSS: 270,831 -> 270,869"

/label="23RSS"

mRNA 272465..272515

/%_Identity=100

/Motif="ATGGCCACCAGGCTCCTGTGCTGGGTGGCCCTCTGTCTCCTGAGCAG"

/annotation_group="L-part1: 272,465 -> 272,511"

/modified_by="zhouhao"

/label="L-part1"

mRNA 272610..272621

/created_by="zhouhao"

/label="L-part2"

gene 272622..272911

/%_Identity=100

/Motif="CATGCTGGAGTCACCCAGACACCCAGGCACAAGGTGACAGACAGTGGACA

AGAAGTGGCTCTGACTTGTGAGCCAATTTCAGGCCATAACTACCTTTACTGGTACAGA

CAGACCTCAGTGGAAGGCATAAAGTTTCTGATTTCCTTCAGCTTTGCCAAACCTCAGG

ACGAGTCAGGAATGCCCAAGGAGCGATTCTCAGCTCAGATGCCTAATGAGTCATTCTC

CACTCTGACGATCCAGCGCACAGAACCCGGGGACTCAGCCGTGTACCTCTGTGCCAGC

AGCTTAGC"

/annotation_group="TRBV: 272,622 -> 272,911"

/modified_by="zhouhao"

/label="TRBV12-19"

sig_peptide 272912..272950

/Mismatches=0

/%_Identity=100

/Motif="cacagcgctgcacagtcgccccctccctgtgcagaaacc"

/annotation_group="23RSS: 272,912 -> 272,950"

/label="23RSS"

mRNA 274539..274589

/%_Identity=100

/Motif="ATGGCCAACAGGCTCTTCTGCTGGGTGGCCCTTTGTCTCCTGGGAGTAGG

T"

/annotation_group="L-part1: 274,539 -> 274,589"

/modified_by="zhouhao"

/label="L-part1"

mRNA 274685..274696

/created_by="zhouhao"

/label="L-part2 "

gene 274697..274986

/%_Identity=100

/Motif="GATGCCGGTGTCACCCAGACACCCAGGCACGAGGTGACAACAAGGGGACA

AGATGTGACTATGAATTGTGAGCCAATTCCAGGCCATGACTACCTTTACTGGTACAGA

CAGACCTCAGTGAAGGGACTGGAGTTTATGATTTACTTCAGCTATGCAAAACCTCTGG

ATGAGACAGGGCTGCCCAAGGAGCGATTCTCAGCTCACATGCCTAATAAGTCATTCTC

CACTCTGACGATCCAGCGCACAGAACCCGGGGACTCAGCCGTGTACCTCTGTGCCAGC

AGATTAGC"

/annotation_group="TRBV: 274,697 -> 274,986"

/modified_by="zhouhao"

/label="TRBV12-20"

sig_peptide 274987..275025

/Mismatches=0

/%_Identity=100

/Motif="cacagcgctgcacagtcaccccctccctgtgcagaaacc"

/annotation_group="23RSS: 274,987 -> 275,025"

/label="23RSS"

mRNA 276617..276661

/%_Identity=100

/Motif="ggccaccatgctcctgtgctgggtagccctttgtctcctgggag"

/annotation_group="L-part1: 276,617 -> 276,660"

/modified_by="zhouhao"

/label="L-part1"

mRNA 276760..276771

/created_by="zhouhao"

/label="L-part2"

gene 276772..277075

/%_Identity=100

/Motif="CATGCTGGAGTCACCCAGACACCCAGGCGCAAAGTGACAACAAGGGGACA

AGGAGTGACTCTGAGTTGTGAGCCAATTTTAGGCCATAATGTCATTTACTGGTACTGA

CAGACCTCAGTGCAGGGACTGGAACTTATGATTTACTTTCGTAGTCAAACTACTGTAG

GTGACACAGGGCTGCCCAACGAGCGATTCTCAGCTCAGATGCCTAAGGAGTCATGCTC

CACTCTGAAGATCCAGCCCACAGAACCCGAGGACTCAGCCGTGTACCTCTGTGCCAGC

AGCTTAGT"

/annotation_group="TRBV: 276,772 -> 277,061"

/modified_by="zhouhao"

/label="TRBV12-21 P"

sig_peptide 277076..277114

/%_Identity=100

/Motif="cacagcgctgcacagtcgccccctccccatgcagaaacc"

/annotation_group="L-part1: 277,076 -> 277,114"

/modified_by="zhouhao"

/label="23RSS"

mRNA 279405..279455

/Mismatches=0

/%_Identity=100

/Motif="ATGGCCACCAGGCTCCTCTGCTGGGTGGCCCTTTGTCTCCTGGGAGTAGG

T"

/annotation_group="L-part1: 279,405 -> 279,455"

/label="L-part1"

mRNA 279405..279455

/%_Identity=100

/Motif="ATGGCCACCAGGCTCCTCTGCTGGGTGGCCCTTTGTCTCCTGGGAGTAGG

T"

/annotation_group="L-part1: 279,405 -> 279,455"

/modified_by="zhouhao"

/label="L-part1"

mRNA 279550..279561

/created_by="zhouhao"

/label="L-part2"

gene 279562..279851

/created_by="zhouhao"

/modified_by="zhouhao"

/label="TRBV12-22"

sig_peptide 279852..279890

/%_Identity=100

/Motif="cacagcgctgcatagtcaccccctccctgtgcagaaacc"

/annotation_group="23RSS: 279,852 -> 279,890"

/modified_by="zhouhao"

/label="23RSS"

mRNA 281944..281994

/%_Identity=100

/Motif="ATGGCCACCAGGCTCCTCTGCTGGGTGGCCCTTTGTCTCCTGGGAGTAGG

T"

/annotation_group="L-part1: 281,944 -> 281,994"

/modified_by="zhouhao"

/label="L-part1"

mRNA 282088..282099

/created_by="zhouhao"

/label="L-part2"

gene 282100..282345

/%_Identity=100

/Motif="CATGCTGGAGTCACCCAGACACCCAGGCACAAAGTGACAATGAAGGGACA

AAAACAGAAGCACATGTCTTTCTGGTATATCATGTTTTACTATAAATTTATATGAGCC

AGGACAAATCTATGAAACCTACAGATCAAGTTTTTCCCCGTAATTCAACAAAGTGAGA

CTGGAGGTGGAAGAGGGGTGATGCTTACTGCTGTGTTCCATATCAATTTGTCTCAGTT

ACTAGCATGTTCATTGTCAAGT"

/annotation_group="TRBV: 282,100 -> 282,345"

/modified_by="zhouhao"

/label="TRBVU2 P"

mRNA 282709..282759

/%_Identity=100

/Motif="ATGGCCACCAGGCTCCTCTGCTGGGTGGCCCTTTGTCTCCTGGGAGTAGG

T"

/annotation_group="L-part1: 282,709 -> 282,759"

/modified_by="zhouhao"

/label="L-part1"

mRNA 282854..282865

/created_by="zhouhao"

/modified_by="zhouhao"

/label="L-part2"

gene 282866..283155

/created_by="zhouhao"

/modified_by="zhouhao"

/label="TRBV12-23"

sig_peptide 283156..283194

/%_Identity=100

/Motif="cacagcgctgcacagtcgccccctccgtgtgcagaaacc"

/annotation_group="23RSS: 283,156 -> 283,194"

/modified_by="zhouhao"

/label="23RSS"

mRNA 284771..284821

/%_Identity=100

/Motif="ATGGCCACCAGGCTCCTCTGCTGGGTGGCCCTTTGTCTCCTGGGGGTAGG

T"

/annotation_group="L-part1: 284,771 -> 284,821"

/modified_by="zhouhao"

/label="L-part1"

mRNA 284916..284927

/created_by="zhouhao"

/label="L-part2"

gene 284928..285217

/created_by="zhouhao"

/modified_by="zhouhao"

/label="TRBV12-24"

sig_peptide 285218..285256

/%_Identity=100

/Motif="cacagcactgcacaatcgccccctccctgtgcagaaacc"

/annotation_group="23RSS: 285,218 -> 285,256"

/modified_by="zhouhao"

/label="23RSS"

mRNA 287572..287622

/%_Identity=100

/Motif="ATGGCCACCAGGCTCCTCTGCTGGGTGGCCCTTGGTCTCCTGGGAGTAGG

T"

/annotation_group="L-part1: 287,572 -> 287,622"

/modified_by="zhouhao"

/label="L-part1"

mRNA 287718..287729

/created_by="zhouhao"

/modified_by="zhouhao"

/label="L-part2"

gene 287730..288019

/created_by="zhouhao"

/modified_by="zhouhao"

/label="TRBV12-25"

sig_peptide 288020..288058

/%_Identity=100

/Motif="cacagcgctgcacagtcgcccccctccctgtgcagaaac"

/annotation_group="23RSS23RSS: 288,020 -> 288,058"

/modified_by="zhouhao"

/label="23RSS"

mRNA 289672..289722

/%_Identity=100

/Motif="ATGGCCACCAGGCTCCTCTGCTGGGTGGCCCTCTGTCTCCTGAGCAGAGG

T"

/annotation_group="L-part1: 289,672 -> 289,722"

/modified_by="zhouhao"

/label="L-part1"

mRNA 289817..289828

/created_by="zhouhao"

/label="L-part2"

gene 289829..290118

/created_by="zhouhao"

/modified_by="zhouhao"

/label="TRBV12-26"

sig_peptide 290119..290157

/%_Identity=100

/Motif="cacagcgctgcacagtcgccccctccctgtgcagaaacc"

/annotation_group="23RSS: 290,119 -> 290,157"

/modified_by="zhouhao"

/label="23RSS"

mRNA 292447..292497

/%_Identity=100

/Motif="ATGGCCACCAGGCTCCTTTGCTGGGTGGCCCTTTGTCTCCTGGGAGTAGG

T"

/annotation_group="L-part1: 292,447 -> 292,497"

/modified_by="zhouhao"

/label="L-part1"

mRNA 292591..292602

/created_by="zhouhao"

/label="L-part2"

gene 292603..292892

/%_Identity=100

/Motif="CATGCTGGAGTCACCCAGACACCCAGGCACAAGGTGACAAAGAGGGGACA

AGAAGTGGCTATGAGTTGTGAGCCAGTCTCAGGCCATAATACCCTTTACTGGTACAGA

CAGACCTCCGTGGAAGGCATAAAGCTTCTGATTTACTTCGTCAATCAAAGCCCTGTGG

ACAAGGCAGGGCTGCCCAAGGAGCGATTCTCAGCTCTGATGCCTAATAAGTCGTTCTC

CACTCTGACGATCCAGCGCACAGAACCCGGGGACTCA"

/annotation_group="TRBV: 292,603 -> 292,863"

/modified_by="zhouhao"

/label="TRBV12-27"

sig_peptide 292893..292931

/Mismatches=0

/%_Identity=100

/Motif="cacagcgctgcacagttgccccctccctgtgcagaaacc"

/annotation_group="23RSS: 292,893 -> 292,931"

/label="23RSS"

mRNA 295147..295197

/%_Identity=100

/Motif="ATGGTCACCAGGCTCCTCTGCTGGGTGGCCCTTTGTCTCCTGGGAGTAGG

T"

/annotation_group="L-part1: 295,147 -> 295,197"

/modified_by="zhouhao"

/label="L-part1"

mRNA 295291..295302

/created_by="zhouhao"

/label="L-part2"

gene 295303..295592

/%_Identity=100

/Motif="CATGCTGGAGTCACCCAGACACCCAGGCACGAGGTGACAAAGAAGGGACA

AGAAGTAACTATGAGCTGTGAGCCAATTTCAGGCCAGAACTTCCTTGTCTGGTACAGA

CAGACCTCAGTGGAAGGCATAAAGTTTCTGATTTCCTTCAGCTATGCCAAACCTCTGG

ACAAGTCAGGAATGCCCAAGGAGCGATTCTCAGCTCAGATGCCTAATGAGTCGTTCTC

CACTCTGACGATCCAGCCCACAGAACCCGGGGACTCAGCCGTGTACCTCTGTGCCAGC

AGCTTAGC"

/annotation_group="TRBV: 295,303 -> 295,592"

/modified_by="zhouhao"

/label="TRBV12-28"

sig_peptide 295593..295631

/Mismatches=0

/%_Identity=100

/Motif="cacagcgctgcacagtcgccccctccctgtgcagaaacc"

/annotation_group="23RSS: 295,593 -> 295,631"

/label="23RSS"

mRNA 297253..297303

/%_Identity=100

/Motif="ATGGCCACCAGGATCCTCTGCTGGGTGGCCCTCTGTCTCCTGAGCAGAAG

T"

/annotation_group="L-part1: 297,253 -> 297,303"

/modified_by="zhouhao"

/label="L-part1"

mRNA 297398..297409

/created_by="zhouhao"

/label="L-part2"

gene 297410..297699

/created_by="zhouhao"

/modified_by="zhouhao"

/label="TRBV12-29"

sig_peptide 297700..297738

/%_Identity=100

/Motif="cacagcgctgcacagtcaccccctccctgtgcagaaacc"

/annotation_group="23RSS: 297,700 -> 297,738"

/modified_by="zhouhao"

/label="23RSS"

mRNA 300035..300085

/%_Identity=100

/Motif="ATGGCCAGCAGGCTCCTCTGCTGGGTGGCCCTTTGTCTCCTGGGACTAGG

T"

/annotation_group="L-part1: 300,035 -> 300,085"

/modified_by="zhouhao"

/label="L-part1"

mRNA 300180..300191

/created_by="zhouhao"

/label="L-part2"

gene 300192..300481

/%_Identity=100

/Motif="GATGCTGGCGTCACCCAGACACCCAAGCACAAGGTGACAAAGAGGGGACA

AGATGTGACTATGAATTGTGAGCCAATTCCAGGCCATGACTACCTTTACTGGTACAGA

CAGACCTCAGTGAAGGGGCTGGAGTTTATGATTTACTTCAGCTATGCAAAACCTGTGG

ATGACAGAGGGATTCTCAAGGGGCGATTCTCAGCTCAGATGCCTAATGAGTCATTCTG

CACTCTGAAGATCCAGCCCACAGAACCCGGGGACTCAGCCGTGTACCTCTGTGCCAGC

AGCTTAGC"

/annotation_group="TRBV: 300,192 -> 300,481"

/modified_by="zhouhao"

/label="TRBV12-30"

sig_peptide 300482..300520

/Mismatches=0

/%_Identity=100

/Motif="cacagcgctgcacagccgccccctccctgtgcagaaacc"

/annotation_group="23RSS: 300,482 -> 300,520"

/label="23RSS"

mRNA 302103..302153

/%_Identity=100

/Motif="ATGGCCACCAGGCTCCTCTGCTGGGTGGCCCTTTGTCTCCTGGGAGTAGG

T"

/annotation_group="L-part1: 302,103 -> 302,153"

/modified_by="zhouhao"

/label="L-part1"

mRNA 302249..302260

/created_by="zhouhao"

/label="L-part2"

gene 302261..302550

/created_by="zhouhao"

/modified_by="zhouhao"

/label="TRBV12-31 P"

sig_peptide 302551..302589

/%_Identity=100

/Motif="cacagtgctgcacagtcgccccctccctgtgcagaaccc"

/annotation_group="23RSS: 302,551 -> 302,589"

/modified_by="zhouhao"

/label="23RSS"

mRNA 304893..304943

/%_Identity=100

/Motif="ATGGCCACCAGGCTCCTGTGCTGGGTGGCCCTTTGTCTCCTGGGAGTAGG

T"

/annotation_group="L-part1: 304,893 -> 304,943"

/modified_by="zhouhao"

/label="L-part1"

mRNA 305036..305047

/created_by="zhouhao"

/label="L-part2"

gene 305048..305337

/created_by="zhouhao"

/modified_by="zhouhao"

/label="TRBV12-32"

sig_peptide 305338..305376

/%_Identity=100

/Motif="cacagcgctgcacagtcgccccctccctgtgcagaaacc"

/annotation_group="23RSS: 305,338 -> 305,376"

/modified_by="zhouhao"

/label="23RSS"

mRNA 307685..307735

/%_Identity=100

/Motif="ATGGCCACCAGGCTCCTGTGCTGGGTGGCCCTTTGTCTCCTGGGAGTAGG

T"

/annotation_group="L-part1: 307,685 -> 307,735"

/modified_by="zhouhao"

/label="L-part1"

mRNA 307829..307840

/created_by="zhouhao"

/label="L-part2"

gene 307841..308130

/created_by="zhouhao"

/modified_by="zhouhao"

/label="TRBV12-33"

sig_peptide 308131..308169

/%_Identity=100

/Motif="cacagcgctgcacagttgccccctccctatgcagaaacc"

/annotation_group="23RSS23RSS: 308,131 -> 308,169"

/modified_by="zhouhao"

/label="23RSS"

mRNA 309755..309805

/%_Identity=100

/Motif="ATGGCCACCAGGCTCCTCTGCTGGGTGGCCCTCTGTCTCCTGAGCAGAGG

T"

/annotation_group="L-part1: 309,755 -> 309,805"

/modified_by="zhouhao"

/label="L-part1"

mRNA 309900..309911

/created_by="zhouhao"

/label="L-part2"

gene 309912..310200

/created_by="zhouhao"

/modified_by="zhouhao"

/label="TRBV12-34"

sig_peptide 310201..310239

/%_Identity=100

/Motif="cacagcgctgcacagtcaccccctccctgtgcagaaacc"

/annotation_group="23RSS: 310,201 -> 310,239"

/modified_by="zhouhao"

/label="23RSS"

mRNA 312546..312596

/%_Identity=100

/Motif="ATGGCCACCAGGCTCCTCTGCTGGGTGGCCCTTTGTCTCCTGGGCATAG"

/annotation_group="L-part1: 312,546 -> 312,594"

/modified_by="zhouhao"

/label="L-part1"

mRNA 312690..312701

/created_by="zhouhao"

/label="L-part2"

gene 312702..312991

/%_Identity=100

/Motif="CATGCTGGAGTCACCCAGACACCCAGGCACAAGGTGACAAAGAGGGGACA

AGATGTGACTATGAGTTGTGAGCCAGTCTCAGGCCATGACTACCTTTACTGGTACAGA

CAGACCTCCGTGGAAGGCATAAAGTTTCTGATTTACTTCGTCAATCAAAGCCCTACGG

ACAAGACAGGGCTGCCCAAGGAGCGATTCTCAGCTCAGATGCCTAATAAGTCGTTCTC

CACTCTGACGATCCAGCCCACAGAACCTGGGGACTCAGCCGTGTACCTCTGTGCCAGC

AGCTTAGC"

/annotation_group="TRBV: 312,702 -> 312,991"

/modified_by="zhouhao"

/label="TRBV12-35"

sig_peptide 312992..313030

/%_Identity=100

/Motif="cacagcgctgcacagtcatcccctccctgtgcagaaacc"

/annotation_group="23RSS: 312,992 -> 313,030"

/modified_by="zhouhao"

/label="23RSS"

mRNA 315060..315110

/%_Identity=100

/Motif="ATGGCCACCAGGCTCCTCTGCTGGGTGGCCCTTTGTGTCCTGGGCATAGG

T"

/annotation_group="L-part1: 315,060 -> 315,110"

/modified_by="zhouhao"

/label="L-part1"

mRNA 315206..315217

/created_by="zhouhao"

/label="L-part2"

gene 315218..315507

/created_by="zhouhao"

/modified_by="zhouhao"

/label="TRBV12-36"

sig_peptide 315508..315546

/Mismatches=0

/%_Identity=100

/Motif="cacagcgctgcacagtcgccccctccctgtgcagaaacc"

/annotation_group="23RSS: 315,508 -> 315,546"

/label="23RSS"

mRNA 317843..317893

/%_Identity=100

/Motif="ATGGGCACCAGGCTCCTGTGCTGGGTGGCCCTTTGTCTCCTGGGAGTAGG

T"

/annotation_group="L-part1: 317,843 -> 317,893"

/modified_by="zhouhao"

/label="L-part1"

mRNA 317988..317999

/created_by="zhouhao"

/label="L-part2"

gene 318000..318289

/created_by="zhouhao"

/modified_by="zhouhao"

/label="TRBV12-37"

sig_peptide 318290..318328

/%_Identity=100

/Motif="cacagcgctgcacagtcaccccctccctgtgcagaaacc"

/annotation_group="23RSS: 318,290 -> 318,328"

/modified_by="zhouhao"

/label="23RSS"

mRNA 320361..320411

/%_Identity=100

/Motif="ATGGTTTGCAGGCTTCTCAGCGTGGTGTCCCTTTGTCTCCTGGGAGCAA"

/annotation_group="L-part1: 320,361 -> 320,409"

/modified_by="zhouhao"

/label="L-part1"

mRNA 320494..320505

/created_by="zhouhao"

/label="L-part2"

gene 320506..320795

/%_Identity=100

/Motif="TCCGCTGGAGTCACTCAGTTCCCCAGCCACAGGGTCATAGAGAAGGAACA

GGCCGTGACTCTGAGATGTGACCCGATTTCCGGACATGAGACTCTCCTTTGGTACCAA

CGTGCCACGGGGAAGGAAATAACCTTTCTCATCAGCTTCCTGAAAGAGCTCGTGCGGG

ATAAATCAGGGATGCCCGCCGATCGGTTCTCAGCCGAGAGGACCGAAGGGACGTTTTC

CACCCTGAAGATCCAGCCTGCACGGCCGGAGGACTCCGGAGTTTACTTCTGTGCCAGC

AGCCTCAC"

/annotation_group="TRBV: 320,506 -> 320,795"

/modified_by="zhouhao"

/label="TRBV14"

sig_peptide 320796..320834

/Mismatches=0

/%_Identity=100

/Motif="cacagcgctgcagactcaggccctccctgcgcgaaaacg"

/annotation_group="23RSS: 320,796 -> 320,834"

/label="23RSS"

mRNA 322159..322209

/%_Identity=100

/Motif="ATGGGCCCCAGGCTCCTCCACTGTGTGGCCCTTTGCCTCCTGGGAGCGAG

T"

/annotation_group="L-part1: 322,159 -> 322,209"

/modified_by="zhouhao"

/label="L-part1"

mRNA 322331..322342

/created_by="zhouhao"

/label="L-part2"

gene 322343..322629

/%_Identity=100

/Motif="TCTCTGCAGGTCATGTGCGAGCCACGGTCACCCAGAGCCCAAGATACCAG

ATTACCAGGACGGGGCAACCAGTGACCTTGACTTGTTCTCAGGACCTGAGCCACGACA

CCATGTACTGGTACCAACAGAAGCTGAGCCAAGCCCCAAAGCTGCTGCTCTACTACTA

TGACCAGATACTTAACAAAGAAACAGACACCTCGGACAACTTCCAGCCCAGCCGGGCT

AACACTTCCTTCTGCTCGCTGGGCCTGCGAGCGCCGGGCTTGGGCGACTCCGCCCTGT

ACCTCTGTGCGAGCAGCAAAGG"

/annotation_group="TRBV: 322,326 -> 322,629"

/modified_by="zhouhao"

/label="TRBV15"

sig_peptide 322630..322668

/Mismatches=0

/%_Identity=100

/Motif="cacagagggcgagcgctgcctccccaccactcacaaacc"

/annotation_group="23RSS: 322,630 -> 322,668"

/label="23RSS"

mRNA 326239..326289

/%_Identity=100

/Motif="ATGGGCGCCGCAGCCATCTGCTCCATGGTCCTCTGTCTCCTGGGTGCAGG

T"

/annotation_group="L-part1: 326,239 -> 326,289"

/modified_by="zhouhao"

/label="L-part1"

mRNA 326377..326391

/created_by="zhouhao"

/label="L-part2"

gene 326392..326678

/%_Identity=100

/Motif="GCAGCAGAAGTCACCCAGACCCCAGGGCACCTGATCACAGGGAAAGGACA

GAATGTGAAAATGTCTTGTGTCTACCCACAGGGACATCGTTATGTTTACTGGTACCAA

CAGATCCTGGCAAAAGAGTTCAAGTTCTTGGTTTCCTTCCAGGACAAAAATGTTTTTG

AAACAGAAATGCCCAAGGACAGATTTTTAGCTGAGTGTCCCCCAGACTCACCCTGTAG

CCTGGAGATCCAGGGTGTGGAGCTGCAGGATTCAGCCACGTACCTCTGCGCCAGCGTT

GAACG"

/annotation_group="TRBV: 326,392 -> 326,678"

/modified_by="zhouhao"

/label="TRBV16"

sig_peptide 326679..326717

/Mismatches=0

/%_Identity=100

/Motif="cacagtgctaaatatcagctctgcctgctgcacaaactc"

/annotation_group="23RSS: 326,679 -> 326,717"

/label="23RSS"

mRNA 328947..329000

/%_Identity=100

/Motif="atgcggctgctgaccgtgcccagggcaccaggctcctccgccggacccac

gctctgtctccgggaggcat"

/annotation_group="L-part1: 328,947 -> 329,016"

/modified_by="zhouhao"

/label="L-part1"

mRNA 329109..329117

/created_by="zhouhao"

/label="L-part2"

gene 329118..329357

/%_Identity=100

/Motif="TGGAGGTAGCTGGAGTCCCTGCCCCAGGGTCACACACGTGGGACCAGGGG

TGATTCTGAGATGTGATCCAATCTCTGGGCACGTATTTGTTTACTGCTATCGACAAAA

TCTCAGGCAGGAAATGGAATTCCTGATTTCCTCCCAATGCCGAAACCCTGAGGCTAAG

CTGGGAATGCCCGAGAAGACCCCGCCCACAGAGCTGGCGGACCCTGCTCGGTGTCTCT

GCGCCTCGGTGGTGCTGGCGTGGTGAGGCCGGGTGAGTTCCCTCCAGGGTGCAACTCA

GGCGCCACTAGCTTCTTCCTCTCCCCCCAAGTCCACAAAGACACTTGAAAAGGCCCCT

TCCTCACCTCTCTGCCCCAGAACGAGCAGG"

/annotation_group="TRBV: 329,117 -> 329,486"

/modified_by="zhouhao"

/label="TRBV17 ORF"

mRNA 332701..332751

/%_Identity=100

/Motif="ATGGGCTCCACACTCCGCTGCTGGGTGCTCATCTGGCTCCTGGGGGCAGG

T"

/annotation_group="L-part1: 332,701 -> 332,751"

/modified_by="zhouhao"

/label="L-part1"

mRNA 333030..333041

/created_by="zhouhao"

/label="L-part2"

gene 333042..333331

/%_Identity=100

/Motif="AATGCCAGCGTCACCCAGCACCCCAGACACCTGGTCGTGGAGCGAGGACA

GGAAGCGACCCTGAGGTGCAGCCCCATGAAAGGCCACACTTATGTTTTCTGGTATCGG

CAGCTCCCAGAGGAAGGGCTGAAGTTCATGATTTCCCTGCGGAGACAACAAATCATGG

ACAAGTCAGGAATGCCATCAGAACGCTTTTCTGCCACATTTCCCAAAGAGGGGCCCAG

CGTCTTCAACGTCCAGCGGGCAGAGCCCGGTGACTCCACCGTGTATTTCTGCGCTAGC

TCCTTGTC"

/annotation_group="TRBV: 333,042 -> 333,331"

/modified_by="zhouhao"

/label="TRBV18 "

sig_peptide 333332..333370

/Mismatches=0

/%_Identity=100

/Motif="cacaccagctcagagtcacatcctcccagagcacaaacc"

/annotation_group="23RSS: 333,332 -> 333,370"

/label="23RSS"

mRNA 335493..335543

/%_Identity=100

/Motif="ATGGGCAGCCAGGTGCTCTGCTGCGTGCTCCTTTGTCTCCTGGGAGCAGG

T"

/annotation_group="L-part1: 335,493 -> 335,543"

/modified_by="zhouhao"

/label="L-part1"

mRNA 335673..335684

/created_by="zhouhao"

/label="L-part2"

gene 335685..335971

/%_Identity=100

/Motif="GGTGGCAGAATCACTCAGACCCCAAAATACCTGTCCAAGGAGGAAGGACA

AGATGTGACCCTGCACTGTGAACAGGACTTTGGTCATGACTACATGTCCTGGTACCGA

CAGGACCCCGGGCAGGGGCTGAGGCTGATCTACTTCTCCAGAGGTGAAACCAGTGTTG

AGGAGGGAGACCTGGCTACAGGCTACCGGGCCTCTCGGGAGAAGAAGGCATCCTTTCC

TCTCACCGTGCCATCGACCAGGAAGAACCAGACAGCTGTGTACCTCTGTGCCAGCAGC

TTAGACA"

/annotation_group="TRBV: 335,685 -> 335,973"

/modified_by="zhouhao"

/label="TRBV19-1"

sig_peptide 335972..336010

/Mismatches=0

/%_Identity=100

/Motif="cacagtggggcacagccacctcctgtctgtgcacaaatg"

/annotation_group="23RSS: 335,972 -> 336,010"

/label="23RSS"

mRNA 339951..340010

/created_by="zhouhao"

/modified_by="zhouhao"

/label="L-part1"

mRNA 340206..340220

/created_by="zhouhao"

/label="L-part2"

gene 340221..340513

/%_Identity=100

/Motif="GGTGCTGTCGTCTCTCAGAGTCCAAGAAGGGTCGTCTGTGCACGTGGGAC

CCGTGTGACGATCGAGTGCCATGCAGTGGACTTTCAGGCCTTCACTGTGTTTTGGTAT

CAGCAGTTCCCGAATCAGGGCCTCGTCCCCATGGCCACTTCCAATGTGGGCTCTCCTG

CCACACATGAGCAAGGTTTCCCCCAGGACAGCTTTCGCATCAGCCACCCAAACCAGTC

TTTCTGGGATCTGACGGTGACGAGTGCGCGCCCTGAACACAGCGGCCTCTACTTCTGT

GCTGCCAGTGA"

/annotation_group="TRBV: 340,221 -> 340,513"

/modified_by="zhouhao"

/label="TRBV20-1 "

sig_peptide 340514..340552

/Mismatches=0

/%_Identity=100

/Motif="cacagcgctgggcagacatgagaggcccaggcaggaacc"

/annotation_group="23RSS: 340,514 -> 340,552"

/label="23RSS"

mRNA 341691..341741

/%_Identity=100

/Motif="ATGGGCAGCCAGGTGCTCTGCTGCGTGCTCCTTTGTCTCCTGGGAGCAGG

T"

/annotation_group="L-part1: 341,691 -> 341,741"

/modified_by="zhouhao"

/label="L-part1"

mRNA 341871..341882

/created_by="zhouhao"

/label="L-part2"

gene 341883..342169

/%_Identity=100

/Motif="GGTGGCAGAATCACTCAGACCCCAAAATACCTGTCCAAGGAGGAAGGACG

AGATGTGACCCTGCACTGTGAACAGGACTTTGGTCATAACTACATGTACTGGTACCGA

CAGGACCCCGGGCAGGGGCTGAGGCTGATCTACTTCTCACGAGCAGAAAACCTTGTTG

AGGAGGGAGACCTGGCTGCAGGCTACCAGGCCTCTCGGGAGAAGGAGGCATCCTTTCC

TCTCACTGTGCCATCCACCCGGAAGAACCAGACAGCTGTGTACCTCTGTGCCAGCAGC

TTAG"

/annotation_group="TRBV: 341,883 -> 342,168"

/modified_by="zhouhao"

/label="TRBV19-2"

sig_peptide 342170..342208

/Mismatches=0

/%_Identity=100

/Motif="cacagtggggcacagccacctcctgtctgtgcacaaatg"

/annotation_group="23RSS: 342,170 -> 342,208"

/label="23RSS"

mRNA 346412..346471

/created_by="zhouhao"

/modified_by="zhouhao"

/label="L-part1"

mRNA 346665..346679

/created_by="zhouhao"

/modified_by="zhouhao"

/label="L-part2"

gene 346680..346972

/created_by="zhouhao"

/modified_by="zhouhao"

/label="TRBV20-2"

sig_peptide 346973..347011

/%_Identity=100

/Motif="cacagcgctgggcagacatgagaggcccaggcagcaacc"

/annotation_group="23RSS23RSS: 346,973 -> 347,011"

/modified_by="zhouhao"

/label="23RSS"

mRNA 348882..348932

/%_Identity=100

/Motif="ATGGGCAGCCAGGTGCTCTGCTGCGTGCTCCTTTGTCTCCTGGGAGCAGG

T"

/annotation_group="L-part1: 348,882 -> 348,932"

/modified_by="zhouhao"

/label="L-part1"

mRNA 349062..349073

/created_by="zhouhao"

/label="L-part2"

gene 349074..349360

/%_Identity=100

/Motif="GGTGGTGGAATAACTCAGACCCCAAAATACCTGTCCAAGAAGGAAGGACA

AGATGTCACCCTGCACTGTGAACAGGACTTAGACCATAACGCCATGTACTGGTACCGA

CAGGACCCCGGGCAGGGGCTGAGGCTGATCTACTTCTCCCCAGCAGAAAACCTTGTTG

AGAATGCAGACCTGGCTGGAGGCTACCAGGCCTCTCGGGAGAAGAAGGCATCCTTTCC

TCTCACCGTGCCATCGACCAGGAAGAACCAGACAGCTGTGTACCTCTGTGCCAGCAGC

TTAGA"

/annotation_group="TRBV: 349,074 -> 349,360"

/modified_by="zhouhao"

/label="TRBV19-3"

sig_peptide 349361..349399

/Mismatches=0

/%_Identity=100

/Motif="cacagtggggcacagccacctcctgtctgtgcacaaatg"

/annotation_group="23RSS: 349,361 -> 349,399"

/label="23RSS"

mRNA 353480..353521

/%_Identity=100

/Motif="atgctgctgcttctgctgcttctgctgcttctggggccca"

/annotation_group="L-part1: 353,480 -> 353,519"

/modified_by="zhouhao"

/label="L-part1"

mRNA 353824..353838

/created_by="zhouhao"

/label="L-part2"

gene 353839..354131

/%_Identity=100

/Motif="GGTGCTGGCGTCTCTCAGAGCCCAAGCAGGGTCGTCTGTGCACGTGGGAC

CCGTGTGATGATCGAGTGCCGTGCAGAGGGCATTCAGGCCTACACTGTGTTTTGGTAT

CAGCAGTTCCCGAATCAGGGCCTCGTCCTGATGGCGACCTCCAATGTGGGCTCCTCTG

CCACACACGAACAAGGTTTCCCCCAGGACAGCTTTCGCATCAGCCACCCAAACCAGTC

TTTCTCGGATCTGACGGTAGCGAGTGCGCGCCCTGAACACAGCGGCCTCTACTTCTGT

GCTGCCAGGGA"

/annotation_group="TRBV20: 353,839 -> 354,131"

/modified_by="zhouhao"

/label="TRBV20-3 "

sig_peptide 354132..354170

/Mismatches=0

/%_Identity=100

/Motif="cacagcgctgggcagacatgagaggcccaggcaggaacc"

/annotation_group="23RSS: 354,132 -> 354,170"

/label="23RSS"

mRNA 355301..355351

/%_Identity=100

/Motif="ATGGGCAGCCAGGTGCTCTGCTGCATGCTCCTTTGTCTCCTGGGAGCAG"

/annotation_group="L-part1: 355,301 -> 355,349"

/modified_by="zhouhao"

/label="L-part1"

mRNA 355481..355492

/created_by="zhouhao"

/label="L-part2"

gene 355493..355779

/%_Identity=100

/Motif="GGTGGTAGAATCACGCAGACCCCAACATACCTGTCCAGAGAGGAAGGACG

AGATGTGACCCTGCACTGTGAACAGGACTTTGGTCATGACTACATGTACTGGTACCGA

CAGGACCCGGGGCAGGGGCTGAGGCTGATCTACTTCTCCCGAGCAGAAAACCTTGTTG

AGGAGGGAGACCTGGCTGCAGGCTACCAGGCCTCTCGGGAGAAGAAGGCATCCTTTCC

TCTCACCATGCCATCGACCCGGAAGAACCAGACAGCTGTGTACCTCTGTGCCAGCAGC

TTAGA"

/annotation_group="TRBV: 355,493 -> 355,779"

/modified_by="zhouhao"

/label="TRBV19-4"

sig_peptide 355780..355818

/Mismatches=0

/%_Identity=100

/Motif="cacagtggggcacagccacctcctgtctgtgcacaaatg"

/annotation_group="23RSS: 355,780 -> 355,818"

/label="23RSS"

mRNA 359632..359664

/%_Identity=100

/Motif="ATGCTGCTGCTTCTGCTGCTTCTGGGGCCCAGT"

/annotation_group="L-part1: 359,632 -> 359,664"

/modified_by="zhouhao"

/label="L-part1"

mRNA 359967..359981

/created_by="zhouhao"

/label="L-part2"

gene 359982..360274

/%_Identity=100

/Motif="GGTGCTGTCGTCTCTCAGAGTCCAAGCAGGGTCGTCAGTGCACGTGGGAC

CCGTGTGACGATCGAGTGCCGTGCAGAGGGCGTTCAGGCCTCAACTGTGTTTTGGTAT

CAGCAGGTCCCGAATCAGGGCCTGGTGCTGATGGCCACTTCCAATGTGGGCTCGCCCG

CCACACATGAGCAAGGTTTCCCCCAGGACAGCTTTCCCATCAGCCACCCAAGTCTCAG

TTTCTCATCTCTGACGGTGACGAGTGCGCGCCCTGAACACAGCGGCCTCTACTTCTGT

GCTGCCAGTGA"

/annotation_group="TRBV: 359,982 -> 360,274"

/modified_by="zhouhao"

/label="TRBV20-4"

sig_peptide 360275..360313

/Mismatches=0

/%_Identity=100

/Motif="cacagcgctgggcagacatgagaggcccaggcaggaacc"

/annotation_group="23RSS: 360,275 -> 360,313"

/label="23RSS"

mRNA 361447..361497

/%_Identity=100

/Motif="ATGGGCAGCCAGGTGCTCTGCTGGGTGCTCCTTTGTCTCCTGGGAGCAGG

T"

/annotation_group="L-part1: 361,447 -> 361,497"

/modified_by="zhouhao"

/label="L-part1"

mRNA 361627..361638

/created_by="zhouhao"

/label="L-part2"

gene 361639..361925

/created_by="zhouhao"

/modified_by="zhouhao"

/label="TRBV19-5"

sig_peptide 361926..361964

/Mismatches=0

/%_Identity=100

/Motif="cacagtggggcacagccacctcctgtcagtgcataaatg"

/annotation_group="23RSS: 361,926 -> 361,964"

/label="23RSS"

mRNA 365491..365532

/%_Identity=100

/Motif="atgctgctgcttctgctgcttctgctgcttctggggccca"

/annotation_group="L-part1: 365,491 -> 365,530"

/modified_by="zhouhao"

/label="L-part1"

mRNA 365842..365862

/created_by="zhouhao"

/label="L-part2"

gene 365863..366149

/%_Identity=100

/Motif="GGTGCTGTCGTCTCTCAGCATCCAAGCAGGGTCGTCTGTGCTCGTGGGAC

CCGTGTGACAATCGAGTGCCGTGCAGAGGGGCGTTCAGGCCACAACTATGTTGTGGTA

TCAGCAGTTCCCCAGTCAGGGTGTCGTTCTGATGGCGACTTCCAATATGGGCTCTCCT

GCCACACATGAGCAAGGTTTCCCCCAGGACAGCTTTCGCATCAGCCACCCAAACCAGT

CTTTCTCGGATTTGACAGTGACGAGTGCGCGCCCTGAACACAGCGGCCTCTACTTCTG

TGCTGCTACTGA"

/annotation_group="TRBV: 365,856 -> 366,149"

/modified_by="zhouhao"

/label="TRBV20-5 P"

sig_peptide 366150..366188

/Mismatches=0

/%_Identity=100

/Motif="cacagtgctgggtggagatgagaggcccaggcaggaacc"

/annotation_group="23RSS: 366,150 -> 366,188"

/label="23RSS"

mRNA 371674..371724

/%_Identity=100

/Motif="ATGTGCCTAAAACTTCTCTGCTGCGTGGCCCTCTGTGTCTGGGGAGCAGG

T"

/annotation_group="L-part1: 371,674 -> 371,724"

/modified_by="zhouhao"

/label="L-part1"

mRNA 371829..371840

/created_by="zhouhao"

/label="L-part2"

gene 371841..372130

/%_Identity=100

/Motif="GACGACGAGGTCACCCAGAGTCCCAGACACCTGGTCAAAGGAAAAGAACA

GAAAGCGAGGATGGAGTGTGTCCCCAGAAAAGGACACAGCTATGTTTACTGGTATCGT

CGGAAGGCTGGAAGGGGAGCTCACATTTTTGGTTTACTTACAGAATGACCAAGTCATT

GAGCGAGCAGAAGCGGTCAAGGAACGATTTTCATTTCGATGTCCCAAAGACTCGCCCT

GCAGCCTGGAAATCCAGCCCACTGGGCCAGGGGACTCAGCTCAGTTTTTCTGCGCCAG

CAGCGAGTA"

/annotation_group="TRBV: 371,840 -> 372,130"

/modified_by="zhouhao"

/label="TRBV21"

sig_peptide 372131..372169

/Mismatches=0

/%_Identity=100

/Motif="cacagtgctagccatgccagctcctcccagtgcacaaac"

/annotation_group="23RSS: 372,131 -> 372,169"

/label="23RSS"

mRNA 374915..374965

/%_Identity=100

/Motif="tgggcagctgggctctctgctccgcggccctttgtctcacctgggagcag

"

/annotation_group="L-part1: 374,915 -> 374,964"

/modified_by="zhouhao"

/label="L-part1"

mRNA 375087..375095

/created_by="zhouhao"

/label="L-part2"

gene 375096..375382

/%_Identity=100

/Motif="ATGGCTGCTGATATCTACATGCCCCCATTCCGCCTCGCTGGGGCCGGCGG

GGACGTGACCCTGACGTGTAAACAGAACCTGAGGTACAACGCCATGTACTGGTACCGG

CAGGACCCAGCACAAGGCCTGAGGCTGATTTATTAGTCAACGGTGGAGAAAGATGTGC

AGAAAAGAGACGTAGGTGAAGACTACGGTGCCTCTCGAGAGCAGAAGGGGCTGTTTCC

TCTCACTGTGCGCTTGGCCCACGCCAACCAGACGGGCCTGGACCTGTGTTCTGGCTGC

TGCAC"

/annotation_group="TRBV: 375,096 -> 375,382"

/modified_by="zhouhao"

/label="TRBV22 p"

sig_peptide 375383..375421

/Mismatches=0

/%_Identity=100

/Motif="cacagcggggcgcggccaccgccctgcctgcgcgcaaac"

/annotation_group="23RSS: 375,383 -> 375,421"

/label="23RSS"

mRNA 376591..376641

/%_Identity=100

/Motif="ATGGGCTGCAGGCTCCTCTGCTGCGTAGCCCTTTGTCTCCTGCAAGCGGG

T"

/annotation_group="L-part1: 376,591 -> 376,641"

/modified_by="zhouhao"

/label="L-part1"

mRNA 376736..376747

/created_by="zhouhao"

/label="L-part2"

gene 376748..377037

/%_Identity=100

/Motif="GATGCCCAAGTCACTCAGACTCCAGGATATCTGGTCAGAGGCAAAGGACA

GAAAGCAAGGATGACTTGCACCCCCCAAAAAGGACACCCTGTTGTTTACTGGTATCAA

CAGATCCCGAGAAAAGAGTTTAAGTTTTTGATTTCCTTTCAGAACGAAGATATGATTG

ATCAAATCGAGTTGGTGAAAGAGCGATTCTTAGCAGGTTGCCCCAAAACCTCCCCGTG

CAGCCTGGAAATCCAGTCCTCTGAACTAGGCGACTCGGCTGTGTATTTCTGCGCCAGC

AGCCAGGC"

/annotation_group="TRBV: 376,748 -> 377,037"

/modified_by="zhouhao"

/label="TRBV23"

sig_peptide 377038..377076

/Mismatches=0

/%_Identity=100

/Motif="cacagtgctgcgatgtcagctgctcctcatgcacaaacg"

/annotation_group="23RSS: 377,038 -> 377,076"

/label="23RSS"

mRNA 382243..382293

/%_Identity=100

/Motif="ATGAGCTCCCTGCTCCTCTGCTGTGTGGCCTTTTGTCTCCTGGGAACAGG

T"

/annotation_group="L-part1: 382,243 -> 382,293"

/modified_by="zhouhao"

/label="L-part1"

mRNA 382423..382434

/created_by="zhouhao"

/label="L-part2"

gene 382435..382722

/%_Identity=100

/Motif="GATGCTGGAGTTACCCAGACCCCAAGGAATCAGATCGCCAAGACAGGAAA

CAGCATCACTCTGGAATGTTCTCAGAATATGGATCATTACAACATGTACTGGTACCGA

CAAGACCCAGGACTGGGGCTCCGGCTGATCTACTATTCCACTGATGTCCAAGACATTA

ACAATGGAGACATCCCCGATGGCTACAGTGTCTCCAGGACCGAGAAAATGAACTTCCC

CCTCTCTGTGAAGACTGCCAGCCCCAACCAGACAGCTGTCTACTTCTGTGCCAGCAGT

TACTCA"

/annotation_group="TRBV: 382,435 -> 382,722"

/modified_by="zhouhao"

/label="TRBV24"

sig_peptide 382723..382761

/Mismatches=0

/%_Identity=100

/Motif="cacagcgtttccgggccacctgctctctgcacagaaaga"

/annotation_group="23RSS: 382,723 -> 382,761"

/label="23RSS"

mRNA 384670..384732

/%_Identity=100

/Motif="atgtcacctgacgctgcgtgagcagcaggctcctctgctgtgtcgtcctt

tgtctcatctga"

/annotation_group="L-part1: 384,670 -> 384,731"

/modified_by="zhouhao"

/label="L-part1"

mRNA 384866..384877

/created_by="zhouhao"

/label="L-part2"

gene 384878..385149

/%_Identity=100

/Motif="GATGCTGTGGTCATACAGTTCCCAAGACATAGGGTTTCAGGGACAAAAAA

AGGAGTTAACTCTACCGTGTTCATTCTGCAGTGTACTGGCACCGTCAAGACCCAGGAT

ACGGACTATAGCTGATCTACTTATTCCCTTAGTACCCGCACCATTGAAAAAGTTGTCC

CCGGTGGGTATAGTGTCTCTTGAAGAGAACTGAAGCATTTTCCCCTGACCTTGGAGTT

GGCCAGCACCCACCAGGCCTCCGTGCACCTCTGCGCCAGCAGTGGGTC"

/annotation_group="TRBV: 384,878 -> 385,149"

/modified_by="zhouhao"

/label="TRBV26 P"

sig_peptide 385150..385188

/Mismatches=0

/%_Identity=100

/Motif="cacagcgaagctcaaccgcctcctctctgcacaaaatag"

/annotation_group="23RSS: 385,150 -> 385,188"

/label="23RSS"

mRNA 388975..389025

/%_Identity=100

/Motif="ATGGGGCCCCATCTCCTGGGCTGTGTGGTGCTTTGTCTCCTGGGAGCAGG

T"

/annotation_group="L-part1: 388,975 -> 389,025"

/modified_by="zhouhao"

/label="L-part1"

mRNA 389150..389161

/created_by="zhouhao"

/label="L-part2"

gene 389162..389448

/%_Identity=100

/Motif="GAAGCCGAAGTGACCCAGATGCCGAGACACCTCATCACAGAGACCAAAAA

GAAGTTGACCATGACTTGTTCTCAGAATATGGATCATGAGGTTATGTACTGGTATCGA

CAAGACCCTGGACTGGGGCTCAAGCTGATCTATTATTCCATTAACGTGGGCCATGCTG

AACCCGGAGATGTCCCTGATGGGTACAAGGTGTCTCGAACAGAGAAGACAAATTTCCC

ACTGACCCTGGAGTCGGCCAGCATCAGCCAGACCTCTCTGTACTTTTGTGCCAGCAGT

ATATC"

/annotation_group="TRBV: 389,162 -> 389,448"

/modified_by="zhouhao"

/label="TRBV27"

sig_peptide 389449..389487

/Mismatches=0

/%_Identity=100

/Motif="cacagcatgtcaccaccaggggccctccccacaaaaagg"

/annotation_group="23RSS: 389,449 -> 389,487"

/label="23RSS"

mRNA 395988..396038

/%_Identity=100

/Motif="ATGGGACTCAGGCTCCTCTGTGGCGTGGCCTTTTGTTTCCTGGGAGTAGG

T"

/annotation_group="L-part1: 395,988 -> 396,038"

/modified_by="zhouhao"

/label="L-part1"

mRNA 396177..396188

/created_by="zhouhao"

/label="L-part2"

gene 396189..396474

/%_Identity=100

/Motif="GATGCCGAAGTAACCCAAACCGCAAGATATCTGGTCAAGAGAAGGGGAGA

GAAAGTTGTGTTGGAATGTTCCCAGGATATGGACCACGGAAGAATGTTCTGGTACCGA

CAAGACCCAGGTCTGGGGCTGCGGCTGCTCCATTATTCATACGGGGTTGACATTATTG

ACAAAGGAGATTTCCCTGATGGGTACAGTGTCTCCAGGAAGCAGAAGAAGAGCTTCTC

CCTGACCCTGGAGTCGGCCACCCCCGCCCAGACATCTGTGTACTTCTGCGCCAGCAGT

GATCC"

/annotation_group="TRBV: 396,189 -> 396,475"

/modified_by="zhouhao"

/label="TRBV28"

sig_peptide 396475..396513

/Mismatches=0

/%_Identity=100

/Motif="cacagtgctgcacagccacctccctcctgcacaaaaagg"

/annotation_group="23RSS: 396,475 -> 396,513"

/label="23RSS"

mRNA 397510..397545

/%_Identity=100

/Motif="ATGCTGACTTTCCTGCTACTTCTCCTGGGCCGAGGT"

/annotation_group="L-part1: 397,510 -> 397,545"

/modified_by="zhouhao"

/label="L-part1"

mRNA 397801..397815

/created_by="zhouhao"

/label="L-part2"

gene 397816..398105

/%_Identity=100

/Motif="GCTGCTGTCGTCTCTCAAAAGCCGAGCAGGTTCATCTGTCAACGCGGGAC

CTCCGTGACCATCCAGTGTCAGGCTGACAGCCAACTCGCCCTCATGTTCTGGTACCGT

CAGCTTCCAGGACAGAGCTTGACACTGATTGCAACAGCGAATCAGGGCTCTGATGCCA

CTTACGAAAGTGGATTTACAAAGGACAAGTTTCCCATCAGCCGCCCGGACCTAACGTT

CTCCATCCTGACTGTGAGCAACGCCGGCCCCGAAGACAGCAGCTCTTACTTCTGCAGC

GTTGGAGA"

/annotation_group="TRBV: 397,816 -> 398,105"

/modified_by="zhouhao"

/label="TRBV29"

sig_peptide 398106..398144

/Mismatches=0

/%_Identity=100

/Motif="cacagtgccgggcagagatcaaagatccgagcaagaacc"

/annotation_group="23RSS: 398,106 -> 398,144"

/label="23RSS"

sig_peptide complement(416946..416973)

/%_Identity=100

/Motif="cacaatgtttcatctttgtacaaaaacg"

/annotation_group="12RSS: 416,946 <- 416,973"

/modified_by="zhouhao"

/label="12RSS TRBD1"

gene 416974..416985

/%_Identity=100

/Motif="GGGACAGGGGGC"

/annotation_group="TRBD: 416,974 -> 416,985"

/modified_by="zhouhao"

/label="TRBD1"

sig_peptide 416986..417024

/%_Identity=100

/Motif="cacggtgattcatctctatgggaaacctttacaaaaacc"

/annotation_group="23RSS: 416,986 -> 417,024"

/modified_by="zhouhao"

/label="23RSS TRBD1"

sig_peptide complement(417553..417580)

/Mismatches=0

/%_Identity=100

/Motif="cacagtgacgtggggccaggaggaaaac"

/annotation_group="12RSS: 417,553 <- 417,580"

/label="12RSS"

gene 417581..417627

/%_Identity=100

/Motif="CGAACACCCAACTTTTCTTCGGAGACGGCACCAGGCTCACGGTTGTAG"

/annotation_group="TRBJ: 417,581 -> 417,628"

/modified_by="zhouhao"

/label="TRBJ1-1"

sig_peptide 417628..417633

/created_by="zhouhao"

/label="Donor-Splice"

sig_peptide complement(417686..417713)

/Mismatches=0

/%_Identity=100

/Motif="cacatcagaacaggaccactctgaaatg"

/annotation_group="12RSS: 417,686 <- 417,713"

/label="12RSS"

gene 417714..417760

/%_Identity=100

/Motif="TTAACTATGACTATACCTTCGGGACAGGGACCAGGCTGACGGTCGTAG"

/annotation_group="TRBJ: 417,714 -> 417,761"

/modified_by="zhouhao"

/label="TRBJ1-2"

sig_peptide 417761..417766

/created_by="zhouhao"

/label="Donor-Splice"

sig_peptide complement(418269..418296)

/Mismatches=0

/%_Identity=100

/Motif="cacagcctcctgggtccatttcaaaacc"

/annotation_group="12RSS: 418,269 <- 418,296"

/label="12RSS"

gene 418297..418345

/%_Identity=100

/Motif="GTGTTCTGGAAACACCGTCTACTTTGGGCAGGGCAGCCGGCTCACAGTTG

TAG"

/annotation_group="TRBJ: 418,294 -> 418,346"

/modified_by="zhouhao"

/label="TRBJ1-3"

sig_peptide 418346..418351

/created_by="zhouhao"

/label="Donor-Splice"

sig_peptide complement(418882..418909)

/Mismatches=0

/%_Identity=100

/Motif="cacagcacggaagcctcatagagaaact"

/annotation_group="12RSS: 418,882 <- 418,909"

/label="12RSS"

gene 418910..418959

/%_Identity=100

/Motif="GCGGGTAATGAAAAGCTGTTTTTCGGCAGTGGGACCAAGCTGTCTGTCTT

GG"

/annotation_group="TRBJ: 418,909 -> 418,960"

/modified_by="zhouhao"

/label="TRBJ1-4"

sig_peptide 418960..418965

/created_by="zhouhao"

/label="Donor-Splice"

sig_peptide complement(419144..419171)

/Mismatches=0

/%_Identity=100

/Motif="cacagcacaatggacgtgtacaaaaccc"

/annotation_group="12RSS: 419,144 <- 419,171"

/label="12RSS"

gene 419172..419220

/%_Identity=100

/Motif="CAACAACCAGGCGCAGCACTTTGGAGCTGGGACTCGCCTCTCTGTCGTAG

"

/annotation_group="TRBJ: 419,172 -> 419,221"

/modified_by="zhouhao"

/label="TRBJ1-5"

sig_peptide 419221..419226

/created_by="zhouhao"

/label="Donor-Splice"

sig_peptide complement(419616..419643)

/Mismatches=0

/%_Identity=100

/Motif="cacagcggtggaggcctgtggtaaaacc"

/annotation_group="12RSS: 419,616 <- 419,643"

/label="12RSS"

gene 419644..419695

/%_Identity=100

/Motif="TTCCTATAATTCACCCCTCAACTTTGGGGCCGGCACCAGGCTCACCGTGA

CAG"

/annotation_group="TRBJ: 419,644 -> 419,696"

/modified_by="zhouhao"

/label="TRBJ1-6"

sig_peptide 419696..419701

/created_by="zhouhao"

/label="Donor-Splice"

gene 422324..423680

/created_by="zhouhao"

/label="TRBC1"

mRNA 422324..422716

/%_Identity=100

/Motif="GACCTGAAGAAGGTGCAGCCACCCAGGGTCACCGTGTTCGAACCATCGGA

AGCAGAGATTGCCCGCACCCAGAAGGCCACGCTCGTGTGCCTGGCCACAGGCTTCTAC

CCCGACCACGTGGAGCTGAGCTGGTGGGTGAATGGCAAGGAGGTACTGAGTGGGGTCA

GCACCGACCCCCAGCCCTACAAGGAGCACCCCAACCGCAATGACTCCAACTACTGCCT

GAGCAGCCGGCTGAGGGTCTCCGCCGACTTCTGGAGCAACCCCCGCAACTACTTCAGC

TGCCAAGTCCAGTTCTACGGGCTGGGAAAGAACGACACTTGGAAGGAGGAGGACGGTC

CCAAGCCCACCACCCAGAACGTCAGCGCCGGGGCCTGGGGCAGAGCAGGT"

/annotation_group="TRBC Exon1: 422,327 -> 422,716"

/modified_by="zhouhao"

/label="TRBC1 Exon1"

mRNA 423269..423376

/%_Identity=100

/Motif="GCGTCCTATAAGCAAGGGGTCCTGTCTGCCACCCTCATGTATGAGATCCT

GCTGGGGAAGGCCACCGTGTACGCAGTGCTGGTCAGCGCTCTGGTGCTGATGGCCATG

"

/annotation_group="TRBC Exon1: 423,269 -> 423,376"

/modified_by="zhouhao"

/label="TRBC Exon3"

mRNA 423657..423680

/%_Identity=100

/Motif="GTCAAGAGAAAGGATTCCTGATGC"

/annotation_group="TRBC Exon4: 423,657 -> 423,680"

/modified_by="zhouhao"

/label="TRBC Exon4"

sig_peptide complement(426119..426146)

/%_Identity=100

/Motif="cacaatgctacaccatgatacaaaaagt"

/annotation_group="12RSS: 426,119 <- 426,146"

/modified_by="zhouhao"

/label="12RSS TRBD2"

gene 426147..426163

/created_by="zhouhao"

/label="TRBD2"

sig_peptide 426164..426202

/%_Identity=100

/Motif="cacgatgattcaagtggaggagatgcttttacaaaaagc"

/annotation_group="23RSS: 426,164 -> 426,202"

/modified_by="zhouhao"

/label="23RSS TRBD2"

sig_peptide complement(426805..426832)

/Mismatches=0

/%_Identity=100

/Motif="cacagtgggaaggggctgcccagaattc"

/annotation_group="12RSS: 426,805 <- 426,832"

/label="12RSS"

gene 426833..426881

/%_Identity=100

/Motif="GTGCTCCTACGCTGAGCAGCACTTTGGTCCAGGCACCCGGCTCACCGTGC

TAG"

/annotation_group="TRBJ: 426,830 -> 426,882"

/modified_by="zhouhao"

/label="TRBJ2-1"

sig_peptide 426882..426887

/created_by="zhouhao"

/label="Donor-Splice"

sig_peptide complement(426999..427026)

/Mismatches=0

/%_Identity=100

/Motif="cacagccccggggacccaggcgcaaacc"

/annotation_group="12RSS: 426,999 <- 427,026"

/label="12RSS"

gene 427027..427076

/%_Identity=100

/Motif="CAAACACCGGACAGCTGTACTTCGGCAAAGGCTCCAAGCTGACCGTGCTT

G"

/annotation_group="TRBJ: 427,027 -> 427,077"

/modified_by="zhouhao"

/label="TRBJ2-2"

sig_peptide 427077..427082

/created_by="zhouhao"

/label="Donor-Splice"

sig_peptide complement(427275..427302)

/Mismatches=0

/%_Identity=100

/Motif="cacagcgccgagacccaggacaaaaacc"

/annotation_group="12RSS: 427,275 <- 427,302"

/label="12RSS"

gene 427303..427350

/%_Identity=100

/Motif="AACACAGATACTCAGTATTTCGGCCCAGGCACTCGGCTGACCGTGCTAG"

/annotation_group="TRBJ: 427,303 -> 427,351"

/modified_by="zhouhao"

/label="TRBJ2-3"

sig_peptide 427351..427356

/created_by="zhouhao"

/label="Donor-Splice"

sig_peptide complement(427422..427449)

/Mismatches=0

/%_Identity=100

/Motif="cacagcacccgcgcacagcacaaaaaca"

/annotation_group="12RSS: 427,422 <- 427,449"

/label="12RSS"

gene 427450..427497

/%_Identity=100

/Motif="AGCCAAAACACTCAATACTTCGGCCCGGGCACGCGGCTGACCGTGCTAG"

/annotation_group="TRBJ: 427,450 -> 427,498"

/modified_by="zhouhao"

/label="TRBJ2-4"

sig_peptide 427498..427503

/created_by="zhouhao"

/label="Donor-Splice"

sig_peptide complement(427540..427567)

/Mismatches=0

/%_Identity=100

/Motif="cacggccccggagccccacacaaaaacc"

/annotation_group="12RSS: 427,540 <- 427,567"

/label="12RSS"

gene 427568..427614

/%_Identity=100

/Motif="GACGGAGAGACCCAGTACTTCGGGCCCGGCACGCGGCTCCTGGTGCTAG"

/annotation_group="TRBJ: 427,567 -> 427,615"

/modified_by="zhouhao"

/label="TRBJ2-5"

sig_peptide 427615..427620

/created_by="zhouhao"

/label="Donor-Splice"

sig_peptide complement(427633..427660)

/Mismatches=0

/%_Identity=100

/Motif="cacggccccggggctccccaccaaaacc"

/annotation_group="12RSS: 427,633 <- 427,660"

/label="12RSS"

gene 427661..427712

/%_Identity=100

/Motif="CACTACAGGCGCAGCCCTGACTTTCGGGGCCGGCAGCCGGCTGACGGTGC

TCG"

/annotation_group="TRBJ: 427,661 -> 427,713"

/modified_by="zhouhao"

/label="TRBJ2-6"

sig_peptide 427713..427718

/created_by="zhouhao"

/label="Donor-Splice"

sig_peptide complement(427857..427884)

/Mismatches=0

/%_Identity=100

/Motif="cacagagaggcagccccgcacgcaaacc"

/annotation_group="12RSS: 427,857 <- 427,884"

/label="12RSS"

gene 427885..427930

/%_Identity=100

/Motif="CTCGTATGAACAGTACTTCGGCCCCGGCACCAGGCTCACGGTCGTAG"

/annotation_group="TRBJ: 427,885 -> 427,931"

/modified_by="zhouhao"

/label="TRBJ2-7"

sig_peptide 427931..427936

/created_by="zhouhao"

/label="Donor-Splice"

gene 431183..432537

/created_by="zhouhao"

/label="TRBC2"

mRNA 431183..431575

/%_Identity=100

/Motif="GATGACCTGAAGAAGGTGCAGCCACCCAGGGTCACCGTGTTCGAACCATC

GGAAGCAGAGATTGCCCGCACCCAGAAGGCCACACTCGTGTGCCTGGCCACAGGCTTC

TACCCCGACCACGTGGAGCTGAGCTGGTGGGTGAATGGCAAGAAGGTCCTGAGTGGGG

TCAGCACTGACCCCCAGCCCTACAAGGAGCACCCCAACCGCAATGACTCCAACTACTG

CCTGAGCAGCCGGCTGAGGGTCTCCGCCGACTTCTGGAGCAACCCCCGCAACTACTTC

AGCTGCCAAGTCCAGTTCTACGGGCTGGGAAAGAACGACACTTGGAAGGAGGAGGACG

GTCCCAAGCCCACCACCCAGAACGTCAGCGCCGGGGCCTGGGGCAGAGCA"

/annotation_group="TRBC Exon: 431,183 -> 431,572"

/modified_by="zhouhao"

/label="TRBC2 Exon1"

mRNA 432127..432234

/%_Identity=100

/Motif="GCGTCCTATAAGCAAGGGGTCCTGTCTGCCACCCTCGTGTATGAGATCCT

GCTGGGGAAGGCCACCGTGTACGCAGTGCTGGTCAGCGTCCTGGTGCTGATGGCCATG

"

/annotation_group="TRBC Exon: 432,127 -> 432,234"

/modified_by="zhouhao"

/label="TRBC Exon3"

mRNA 432520..432537

/%_Identity=100

/Motif="GTCAAGAAAAAGGATTCC"

/annotation_group="TRBC Exon: 432,520 -> 432,537"

/modified_by="zhouhao"

/label="TRBC Exon4"

sig_peptide complement(444489..444527)

/Mismatches=0

/%_Identity=100

/Motif="cacactgcgctgggtggggcagacctctgtgcaaaaacc"

/annotation_group="23RSS: 444,489 <- 444,527"

/label="23RSS"

gene complement(444530..444811)

/%_Identity=100

/Motif="TGATACTCCAGGCGCAGAGGTAGAGGCCAGAGTCGTTGAGGAGGAGCTTC

TCAGAATGCAGGATGAAGCGGCCGTCCTGGGGCCTGGAGGCCGTGAAGTGCTGGGGCT

CCTGAGACTCTGCGTGGCCAGCACTGATGGAGAAGAAGAGCAGCTGGAGGGCCCCACC

TCCGGCCTGCCGGTACCAGTACAGGTTGGGGCTTGATGTCCCCTTCACCGTGCAGTCC

AGGGAGAGTGGGCTGCCCGCACGCTGGATCTTGGTAGGTGGCCACTGATGGATGGTCT

GAGC"

/annotation_group="TRBV: 444,526 -> 444,811"

/modified_by="zhouhao"

/label="TRBV30"

mRNA complement(444812..444823)

/created_by="zhouhao"

/label="L-part2"

mRNA complement(445178..445220)

/%_Identity=100

/Motif="ATGCTCTGCTCCCTCCCTGCCCTTCTCCTAGGCACTTTCTTAG"

/annotation_group="L-part1: 445,178 <- 445,220"

/modified_by="zhouhao"

/label="L-part1"

gene 473815..488404

/gene="EPHB6"

/note="Derived by automated computational analysis using

gene prediction method: Gnomon."

/db_xref="GeneID:114507584"

/label="EPHB6 gene"

mRNA join(473815..474016,474298..474496,479050..479220,

480558..480757,481039..481103,481386..482139,

482873..483028,483326..483676,483782..483906,

484114..484276,484471..484585,484816..484868,

485091..485210,485324..485571,485841..486014,

486735..486884,487140..487333,487470..487625,

487731..488404)

/gene="EPHB6"

/product="EPH receptor B6, transcript variant X2"

/note="Derived by automated computational analysis using

gene prediction method: Gnomon. Supporting evidence

includes similarity to: 3 long SRA reads, 21 Proteins, and

100% coverage of the annotated genomic feature by RNAseq

alignments, including 8 samples with support for all

annotated introns"

/transcript_id="XM_036010980.1"

/db_xref="GeneID:114507584"

/label="EPHB6 mRNA"

ORIGIN

1 aatatatttg acatacaaca ttgtgtacat tgaacgtgcc caccatgttg attagataca

61 tttcactatt gcaatatgat tgccctcatc accatggccc ttctatcaca tcacagattt

121 tcctccctcc ctccctccct tccttccttc cttcctctgt cttcccttcc ttttcttttt

181 tctttctttc tctctccctc ccttctccct ctctttcttt ccctccccct ccctccctcc

241 cttccttcct tccttccttc tccctccctt cctccttctc cctcccttcc tccttctccc

301 tcccttcctc cttctccctc tctctcttcc ttccttcctt ccttccttcc ttcttccttc

361 ctagaatgat taagatctag tctctagaat gcttgcccct gaccttctaa taatactgag

421 agctgagaag caatatgtga ttagaagaaa gtgttattta ttcttaaagt cctcttgcct

481 cacaatttct atttctttcc catgacaata aattcaactg tggtaatgga gcaagatgct

541 gagaggctca tgtgaaacct atcttagcgc atgtattttt agtttacatg agacttacgg

601 agctttcatt ctgaggtagt gatgtaggca tttaagttgg ctgcttcgga accgattttc

661 aggggcagtt gagcttttga aaatatttta aatcaaatca tgtagtcagc cttctctgcc

721 agttacgtgt ggggtcagcc ctcgaggact ggatgatggg agatttacta acaccggtac

781 ttcacctaga agttcacgtg gctgtacttc ttgttgctcc tgggcagtta acttggcaaa

841 acttgtcttt tcagggttat cagtgacccc ttacctaact gcccacttag ttcttaccat

901 cgactttgta tgcaatatac ttatttctgt tgagtttctt tctcattggt tttagctttt

961 catgaaatag tagagaactt tccttccttc tccaaaccaa ttatttgctt ttactgtttt

1021 gtttgatgtg aatttctaaa gtcccaggta gactgatttc acatgcaagt acaaagtcag

1081 gccatggtca tgtaaaaatt gttttgttta tctgaaaagt agattatcca gacctgtgag

1141 tcttttcagt catattctta tttgaatggc atatcattag aaattcgcat ataattaaaa

1201 taatttaatt ataatagata gctacctcca attatgaaca cataacagga atcatttgat

1261 ctaagatgtt ggcacaggta gtagtgccag aggtgatgtg ggttaatgat aaaggaattt

1321 ggtgaaaatg aacgctccag ggcctgctcc aggtaccgtg ttgctgtagg tgattacttt

1381 gtcacacccc ctgttccctg aggttcaaag ttacctgtgt ctattacatg gtgccccatc

1441 gcctaaaaca aagaaccagg agatagatag atagatagat actcattata ccgtctgaac

1501 attatgttgt ccctcaatga caacctacaa attggttggg aaaataagta acaatgtatg

1561 cacaaaagct tgtatcctgt taattcagaa ttttcagcaa tttggctatg atcatatctt

1621 tgtgccattt gtggaaaatg ttaaaaacca atctttgata gaaacagtta ggatgagaag

1681 ttagaatatg aagtatttaa cctaagaagc acaagattca tttaaacatt taaaaaaaac

1741 attgtttata tgcctcaaac caacatatta tttataaaca gcattattca aagcgagttt

1801 tagatgatct tcagttagca acatattaaa caaattcaat cattcttttc taaaatatta

1861 cctagttaga ctttctagta ttttaaacag ttctctcaat tggcctgaca tgatgaaatt

1921 ttgctttttt tttttttagt catagaaaaa agacatttga tggcttctca tctatataat

1981 aagttgtttt tccttttttc ttttcttgct ttggttcctt gcatctattg taacatttag

2041 cttagggagg agggtcaccc gttgtgatct gtgatggtga gctacatggg gcttccagca

2101 gaggagtcag tagccagcag cgcatgcatg agtgacccgt ccaacaaact gcccggcctt

2161 ctttgccctt cagggatatc ccggatggac agtctttcct gattatacga atccagcttg

2221 tcctccgtgg tggatagaag agttcagaaa atttcataca tctctgaagt ttgatggagt

2281 gtggattgta agtcgctatt ccaggattaa acttattttg gagacggatc tttcaaattt

2341 tgagatgaac tttgagtata atctataata aaatcattta cttctcatag tgatattaaa

2401 atccttttac ttaatgctgg gtttatatgc aataaattag atcctcaaaa ctctggattt

2461 taattctcag tctgctacca gctggttaag ttcttttgga gaaagtcttt tgctttctca

2521 tggctgcagc tttttgcttc attgactctt ttagctttaa aaaaatacta ttttactaga

2581 aattacaact actattatta gtagcaatgg ttcattaaaa tttaaattaa atcattagag

2641 aagaatttag tgggtacctt ttaattttta gcaaacaggg agtgaggaag agtctgaaat

2701 ttgaaaaatt tctctgttag caggtttatg catcggctac ttttaacctt ctttaaagaa

2761 atggatttga tctcaattgt atcttcttct tgcactgcat taagcagaat aaaagggaaa

2821 attataaaat agaatactac cctatcttag gttctttaaa acaatgctga agccatatca

2881 gtaactataa aatcatagcc tgtaagaatt agcctgtgag atagtatttc ttcattaacc

2941 gactgcaatc aatgtgtaaa ggttaaatgt acctggtatc atttagagtg gaaagatacc

3001 ttagatataa cctaatccaa aggatgatat actctggttt ctcttctgat catattactt

3061 gccttttaca aaagatgaaa cagaggtcaa aaaaatgtca gaagattact tacgatcccc

3121 aaatctgtta ctaagagacc ccagctatcc agcttctagt tcagtaaaca ttcccgaaca

3181 actgcctgtt tatcggtgac ccttcccctc tgtgtgtttt gtgtctttga aggaactgga

3241 cgaagtctct aacttgctcc cagcttctga ccgtagctgt gaagacaacc agttgaactt

3301 ccctcccttc actcctcgta agtttttgcc cgttggctac gatgcaggca cgagacgtct

3361 aaaaccacct cggtggaact cagtgtggcc gggagcagtt ttccttctca gtttaaggga

3421 ctgcccccac cgaagccccc tgagcacccc ttcctgggga ccttgctgcc ttgtttgttc

3481 tctgcttgcc ttttcacagt catagtccaa gggcaggagt gtggtggagg tcgtatctcc

3541 cgtggcggtg cctcctgtct cgagttctac atgctcccac cctgttcact gcctctaaac

3601 ccctttaaaa tgtggttcaa gcttaaactt catgacagtg tcagaatact gggagcacac

3661 atcaccttcc attggagtaa atctgtaatt tgtgtgtgta tttgtttagc tcattaccaa

3721 cagccttgtc aagggttcct gctagttctt attttgagtc tcatttctag tccaagtcca

3781 ttttccaggc cctgattttg aaagaatgtg tgtgtgtgtg tgcatgcgtg caataatata

3841 ttctttgtgg aaacaaatgc actccaacat ctcagtggag tacacactac aagtttattt

3901 tttgcccgta taatactcca ctgtgggagg tactcccatc cacgcagtga ctcagcaatc

3961 tagttcactt ccattgcccg cttctccttt cctggagcct tttgtttgca gcgtcctggg

4021 gaaaggagaa agagaagggt agtgggaact ttatggaaag gcctggaagt gcggtgagtt

4081 caatttgctc acattttttt gctcacgctt gcccttggaa tccaatcgga atccaatcag

4141 atagtctcta ctaaacttca agcaaaaatg tggatataat gttatactcc caggaagaag

4201 acacaggctt ggggagcatc tggtcagttt gttccagttt gttctggttt ctccacccgt

4261 accctcggag caaagtttag ctgtcccttt gccccgttag ggcactgaag gggtgaagaa

4321 ctttttttgg agaatcttga cttcaaaccg ttgaacttct tgcaaagtct gctgtttcct

4381 tcccctgctc attctttatt cctctctgtt cattctttgt tccctgattt tttttttcac

4441 cgtggattag tttatcatgt ttctccttta gcctctgaat cttccactta gcccaaacta

4501 agttgttctt gtcatggttt ccctgcagaa cacagcaatc tcacccacct tctgtctgct

4561 cctgggaagc acccgctccc tgcctcccag ccttctgtct gcctgcacgg gacgagctgc

4621 tctgcctctg gtctgctttt aacctttcag gaagagaacg ttcgctttag cttgaaaacg

4681 atttagatgt gtgacctttg cccctgggac agttttagtg ccccgtctta taatggaagt

4741 gactgtgaaa ctcaagccac gaattttgcc atgccagaaa tgtaactgcc tttataactt

4801 atcgttaaac gtgattcgtc acttcaaacc ctgattctgt tgggctggcg ggaaaactca

4861 accccggtca ttcctggctc cacctcccac tggcactgtg cccaggctga ggttctacga

4921 acaggaaggc ttcctgaagg aggggaggtg cgggcccccc caggtattgg tctggaagga

4981 gaaggagaag aatgagactg caggcctcgg gacagaaaat gttgggcgtt ttaggcatga

5041 gaaagggaaa atgctgtaac aatgctgttt ccaaaggtgg catctaagtt cttattcatg

5101 aaaccaccct agtgtcatat ctcagatttt tttaaaaagt gtggctgagg tgagaatgct

5161 tgaaacattg ggatgaatat gctccctttt cactgccctc atatctgttc tttgtgcact

5221 cagctctcct tgattaagtc ggtgtagttg gatgtgtgcg cttcatttat gttttatctc

5281 aaatcaacgt tccgttgctc tgagcttgtt ctgggtcgcc gccactcagc accactctct

5341 gtgagctgaa gagaatgcaa attgatttta atgatctatc tccccaaggg gctcagtgta

5401 tgccccaggt aggaagggca tttaggatag gatttaagat ctggaaaggg ttttgtcagg

5461 tggaggttgg tggggaaggt acaggcgcca ggaagcacgc agggcggtta ctttgtgcga

5521 gtttgctgac ggggaggggg ctggctgacg gatatttccc cacaggagtg ctggaccgct

5581 tgctgttcgc aaggaccctc tgcatggatg ctgagtttca ctggggcctc cactatgacg

5641 tccacagctt gtacggctac tccatggcga gagccaccga cgcgtaagca ctcggttccc

5701 gcctcggtcc agacgtgggg agctgggagg tggagggttt ctcttgtcat cttgaagcat

5761 atctttcaaa tatattctgg ggttataaga atctagaaac tcagatctta caggctgctt

5821 aagagggctt ctcatatatt cccttggttt ctgcacagga ttatttttga ttggcttgtg

5881 actgaggact tgatactagc aggcaactat gtgcgaggta tttagaaccc ctacctggaa

5941 gccagcgctt gcttgggttc tattactgca catgctgtgg tcatcacact agggtttcct

6001 gatttagcaa atgaaaatat agcacaccca gttaagcttg caatggacat aaagactatt

6061 cattgtttac atgaaattca agtttacctg agtggcctat atttatacag caatgttata

6121 ccacaccctc ctggcttcct tccctgctgc ctgctccaga ctccctcttg attttcccct

6181 cagccccaac acgcacacat caccaccacc ccagcactac catcaccacc accagcagca

6241 ccaccagcac caccatcact actaccacca gtaccactaa caccatcacc agcaccaccg

6301 ctaccaccat catcactata accaccacca ccatcactac taccaccagt accaccatca

6361 ccaccaccag caccaccacc agcaccacca tcactactac caccagtacc accagcacca

6421 tcaccagcac caacaccacc accaccacca gcaccaccac tcagtatgaa agtcgaacaa

6481 agggacagat gcttcctgca aaggattcag tccttttcag accttgtcac atgaactttt

6541 tgctaatgac tttgtaaatg gaggactagc tccctgcaaa acccccacca agtgccttcc

6601 ttggaaggtg gtaaaatata agcattacac cgaggagaga agaaattcag ggccaagctt

6661 aagaagattt taatttgaat gtgcaaaaca gatggagtga cttaggaaag aacgtcttgg

6721 tttttctctt cttatttcct tttggtgttt ctagaactct gtggtgggga atagggacag

6781 ggagactttg atgtccttct agaacttttc tcagtccttt cacttgactt ctctagggca

6841 cacggcgtca ctgaccactc ctttgaaact cggttccctt ggttccggca caccgctctc

6901 gcttgactca ctggccctgc tcccagcatc cctcaggacc tctgttcctc ttcaacctct

6961 gggtcttttt tatcttctct ctcactgtcg ataagctgta ggggtccacc cctagactct

7021 tttttttcat tctctgtcaa tgcaaaggta atttatttct tttcagtttt tactggaatt

7081 tattttgata attttaaagt ctatgattcc gtaacattat agcgtaggtc tctccctgcc

7141 ttccagaccc agatacccac agcagccttc tgatcttctc tacttggttg ttgtcttgtc

7201 cctcccatgc cgtgcacctt cccctggtac ctgtttctgc ccaacctcca cagactggct

7261 aatcattttt gaaacgatta aactacccat cttggttggt ggctacacca tccctacctc

7321 ccagaccccc aagagagaaa cttggtgtca tccttgagct atttctttcc atcacaccct

7381 cttttacaat cagtcttcaa tccttttgag tataaaacat ttctataact gtctcctctc

7441 ccctacactt ctttccatcg tcaacactca cacgaagctc acatgagctc cacgactact

7501 agtcttcctt ccctgacttt agctttatct cctactcatc tgtcctcttc tgttgctatt

7561 caaagtgcgg tccaggacca cactggcatc acctggaaac atatcaggaa tgcagcatct

7621 tggccctcac cttagaccgt ggaaatgaga atctgagttt tagcaggatc tccaggggca

7681 ttgaatgtgt gtgaaagttt cctaagcacc actctctccc actgctcagg tggtctttcg

7741 gaatcacacg tctgcccatc actctcccca atgaaaatct gctcacttct ctccccactg

7801 aaaaatcacc ttagccttgg tatcttccat acaataaaag gcagccaccc tagccaaacc

7861 tgtaaggtac ttcataaaaa aacgctttct tagtattttt cttccatctc tgctcctcct

7921 catattttat gctgtggtaa acataactgc ttactgctcc tgaatagaaa actattttgt

7981 tctgccatgt ctttgcctgt gtgctctgtc tgcacgggat gcccttcccc gccccatcca

8041 ttttgcaaac acgcacttat ctttaaagcc tcacctcgag tgttaattct gcaaacctac

8101 ctgacagtcc tagggaaaga ttatctcctc cctctgcctt tatcttgggc tacctttgtt

8161 catattgcac tatgcctaaa gtccaaacat ctgtattata atttttattc acttttcttc

8221 cttgctgcac tgtaaattct ttgagggcgc agtcaccccg tgtgtgctta gcctgaggaa

8281 ttgggtgtgt cctacaggca cggccattga ataaaggcct ggcctgagaa agggaagaag

8341 ggcaggacag gcccagcggg accgggtgac ttagaagcag agactgggcc tcttttgttt

8401 tcctgcacca tcttgtcctc gggacttaac tatgttgctg gctcgtccag gtgagagagc

8461 atccttgccc agtaaatgca gatgttttta tttcaataca cgaactgctc catgaagaga

8521 gacacatatc tgatttcatc ttctcttttc tgactttttt tcccctcatt tgacctttcc

8581 tgatggatca gacagttccc tcatcttcgg agtgagagtg ttgggttgat ggtccccaca

8641 tcccttcttg ttctgaccta tgtagactca cagatgcttc tccctgagat gggaaggtgg

8701 gcagagagtg ggtaaatggc cagggtgcga aggaaggggt aagggagggt gcgtggcagg

8761 aagggaagat tcaaagtgct ttattgctag agcctccatg tgataaaagt gctataaaat

8821 cataatgggg ctggttaata agtcctaata gctttggtta atggttaaaa ggtcgaagtt

8881 cccagagaac agtggaggta aatggcactt ggcacctggg catcaggtag agctggtgaa

8941 aaagcagaca agagtctcat tcagtggggc ctggccacca ggagacttta aatcataaat

9001 ctgatgaaga tgtggctctg ccttagtggg aagtaaagct tggggacagt ggctactggc

9061 ctaccgtgac aatgaaatgc actgtcctgt ggcctaaaag cagaaggaac ccagttcctc

9121 tcagagatca aggcctgagc ataactagac caaccctgag ggatgcaaaa cagaggcttg

9181 gggatgtctg ggcagaagtt tccctctaag ccaaaataaa aagcaggccg tcatctaatg

9241 cgtgttacca acatggcctg accaaatcaa ccctaaactc agatagcttc attcctgttt

9301 cagtttcact ttacgccacc tgaatgtgtt ccctttatga tgggtggttt gcagggtgcc

9361 ataaaccccc tcgccagcgg cgataaaggc aaagcatcgt tagtcccaat gaaggagaaa

9421 tgaggacgga agtgggagag cgagccctct ccattgtgcc ctgatagttt gctcattaaa

9481 atgtcaggac ggggcacttc attttcttaa atgagtcttc tacttccgaa ttacccccct

9541 ttccccacga tcccttgtcc tgcgtgtatg actccccaca gcatcctcag ggatgcaaag

9601 acggaaagcc ttggtggctg ggagagtacc gccaggaacg gcccagggcc cgccggtgcc

9661 ctggtctcag accgtgtgct ggaaggaaag acagaagagc ctgcctgcga agagctagac

9721 gtgactgtgg ctcagccatc actctcctct agttgtaata ccgctccatt tcaaaacatt

9781 aaaagaaagt gtggaccgtt taggaaagga gagggcaagg agaggcctct ttgaaaaagc

9841 attccaaata aaagctagaa aaccgcctac atgtcactgt gttatcccag ccccacccca

9901 tcctttctga gttcctcacc tttaattcct ttgctccact ttctatagag ttttattgga

9961 tgggtaatct tttatatgta atacagtgaa aatagtttat agtttagttg cttttaactt

10021 cattaagagg atcatatgcc atcttctgag acttacgtgt ttgaatggat attagtttgt

10081 gcagattaag atctcctgat gtggggaatt gctaataatg agttctgaag cttctgagga

10141 tgccaggggg cacacaaaat ctcctctcgg gcaagcggca ggtacagggt tctgacctta

10201 gctggtgctg agctgaacgg gattatgaac gggcagcaag gcacggaacc cagtgacgct

10261 gggaaatttt tttctagcgg agaacgtgag ggtcaagaac ccatggttcc aatgtattgt

10321 ggaccaacag ggcaagatca caacagatcc aagatgcaga aaacataatc agggggacgc

10381 gagcccagag agagcccttg ggggtgcttg tactccctca tctgaggtct gaaccctgat

10441 ctaggagtga gatagaagct cttagagagg ctactttcat ctgctcgcct tcatgatgcc

10501 ctgccgtctt ttctcagtgt gatcggaaac ctctgctgcc gttcttcggc aacgggtaag

10561 ccccagacac tcacagctcg cctcccattc attcagtcgg ctgtgctctt tttcagggcc

10621 ctgaataatg tctcccctca aaagaggaac ttcatcttat cccgctctac ttttgccgga

10681 tctggcaagt ttgctgctca ttggttgggg gacaatgcag ccacctggga tgatctccga

10741 tggtccatcc ctagtatcct tgagttcaac ctgtttggca tccccctggt gagtgtcacc

10801 ttggagcttt caccctttat ccttggacat tcttcccagt acccatcttc gaagtgtctg

10861 aaaaccaggg agcatatgtc tccccgtgag atgcattgca aaagtccatt tgtgttctat

10921 ggtgtgagtc gctttctgag gattggatag tagcgtttta ccactgggtt gttgtccatg

10981 gaatctctga aattaggaag acacctccat gagtgaaatg tgttagtatc acaggaaaat

11041 tctattatca gagcattagt gaaattggga accgaatatt ggggatgagc ccttggttca

11101 acagtccgtc ttcctctctt cagctggtcc acggtggcac tcttgtttta tttccctttg

11161 ggctggggga ttagccctga aagcctgaga ccccttgaca cacggcatga tgggaataag

11221 agattccctt tttccttatc tcgcaggtgt acaatcaatg ctttctcctt tctctctgac

11281 cactgtgctc atgtgtgtgt gctcattctc caggtaggcg ccaatatctg tggttataca

11341 aaaaatgtca cggaggagct ctgcagaaga tggatgcagc ttggagcatt ttatccactc

11401 tcgaggaatc acaacgggcc tggcttcaag gtaaggtccc cacgagaagt gacggacact

11461 tcttgggtta tctaatctat ttgtcgtcaa agagaggttg aagaatatcc tctgggttgg

11521 ccctaattgc agcagcaaaa tagataaatt tggagtgata gacctaaaaa tcatggtatg

11581 ggggtgacgg tatgtatgcg ttattgctgg aaactcacca acaaaggtga ggagggtggc

11641 tggagttcct acagtgctgc tgtcacactt gtattacctc ctgtatacgg tgggtcaatc

11701 ggagttcgct gatacttcag tcagcacaag gcaagccact cctagataca ggggagcaag

11761 gagaaaggat ttcgaaaggg gcaggcctgt gatctgaaca tttttttccc ttgttgcaac

11821 attttgtctt tctgtgaatg gtgtgagaga ccacttccca ctacttctca tgacatatcc

11881 cacctttctc cttttctacc ccaaatgaca ttttttttta tgattttggt aagcgtatag

11941 aaatgggttc aaggtccctc ttaaggagat gtttatggtg atggtaggag caagttgggt

12001 gggaggccct ttcttttaat gtagaaatct tctcattgag tagtttagct cctaataggc

12061 tcactggagt aactcactga taatcatttt agctgcagtt cttgacttta tctctgacct

12121 gggagaaatt tgtatcagat cttcaaagga ttttccccct gggctgttcc cagccacttg

12181 tggtctagag agagatggac tcttcctgac ttctaagcag gtggctggga gcagggaggg

12241 caggtgctct tcctgaagag gtggcctgaa ggacttccgt cggtaagagc gagtggtcac

12301 tcatgctgcc cgggcgcagg gcgggttttc gtccccctca actagtgctg accacacagt

12361 gccgacggga accacacatg gaagtttaga agcaggagca attattttta atttatttta

12421 tataactcaa tccatcctga acatcatttc aatgcctgtt ttatacaaaa tattaataat

12481 gagccatttt atatcctttt ccttgtatac taagccttca aaagtcagtg tgcattttgc

12541 acttgtaata catctcagtt tgggtgctgt actttcagca ggtggaggga aaggtaatcc

12601 caccagcaca ataaagttgc atttcactgg aaaaaaattt tttacattga ttcagttttt

12661 aggtttatat ttaaaataag tacaatgaaa tctaattaaa agtccagttc ctcaatctta

12721 ctaacaacag ctcaggtgtt cggcagcccc acgtggcttg cggttgccct ggggcacagt

12781 gtggctctag gctttcccag ttggaggcca gggtgaggta gggtgatgtg gagtggtatt

12841 tccttcggcg tttggcctca gggttttcac tgaaccctac actggggcgt tgagtggtct

12901 catggagtag ttctatttgg agagggaaat gggcaactta agaaacaaaa gaaggggata

12961 aattcagata attgtttcta aaattcagtc cccattccgc agtacaggat gacaggtcga

13021 aacatatgca caactttcaa agtacaagga aagtgaacaa catgagggga aatttatgtt

13081 tctgggtcct gagacaatta gatttccttt gtgaagggtt gtttcattcc tcctagttga

13141 aatctgccaa aaactctctt catgattgct ctgcactttg tgtgttgaag aacaaataga

13201 ggtcactatc tttgggacag aggaagtaaa acaaattttg ctggaaaaac cagacataaa

13261 ttataaagat catagatgca tagcccacgg acacagacga caacatggtg aaggcctggt

13321 gggggtgggg tggggaaggg gagacatctg taacactatc aacaataaca aataaaataa

13381 atgaaataag ttatggcata taatgtacag tatagggaat agaacccata agaaaataac

13441 tatgtgatga cggacggctc ctagactaac tttggtggtc actttgtgag gtacataaat

13501 gttgaaccac tacattatac acctgaaact aatagattct ttatcaactt taatgaaaat

13561 aaagtttaaa agatcaataa acaaagtttc agattcataa aagagcaggg aaagaagaga

13621 tggattccag atcgtggtgg ttaggcaata tgtggcttaa ggcaacgggt ttcaaattct

13681 ctcttagtag taggatgact gtattctcat tatttgccaa atgacgtagg atgagaaacc

13741 tcgatatctg agacccatgg gagcagcccc gaggggcaga gcccacgcta ggccctttat

13801 ctccttggca acctccgtag gctcctctgt cgccctgaag cccccacaga ccagtgtcgg

13861 aagacctccg tccagtgtaa accctgccac ttaactcgca gtttaacctc tgatgtgctt

13921 ttctcatcta taaaacgaag gggcaggacc cagctccttt cagttctgga aatgctttga

13981 catcttcaag ttcgacaacg taactccttt catatgagaa agcttctcat caaaggggac

14041 ttctacaata tggtggagat gtctagtaac tgcttacggt tccttctagg accaggatcc

14101 ggcggccttt ggagaagact ccctgctgtt gaaatcctcc aggcactatc tgaatgtccg

14161 ctacaccctg ctgccctatc tctacaccct cttctaccgc gcgcacaccc tgggcgagac

14221 tgtagccagg cccctggtcc acgagtgagt ttcctgaccc aagaagacat cctttcagat

14281 cgtaattctc cttccctcgc tggtgctaat aaggttcatg agaacctcat tgccctacaa

14341 ggagctgccc gccaggactg gggacttcac ctgccttctc tgtgttccag gttctaccag

14401 gacccagcta cgtgggaggt gcacgagcag ttcttatggg ggcccggact cctcatcaca

14461 ccggttttac atgaagtatg tctgtaatct gaacagtggg gtggcacctc acacttgtcc

14521 tcatggctct ctgtagctga gtaagctgga gcctgagtgt ctttggctgc atttaaacat

14581 aaatatgaat ataaaggaga gaaagattct ggaagtttgg aaattagaaa aatagtaact

14641 cttgttccta ctgcctgacc aagaaaaaca atttccattt ttgcgagatt gcattcaagc

14701 ccatgtcttt aagcatactg atgccgacag ggccctcaaa tgccttggga tggccatctg

14761 cctgtgtttc cctttgacca gagaggatgt gatattgggg tcaggaagga gttcctgtgg

14821 acttcatggt gtgatccggg gcagctgtgg gtgggggaag tgggggagag aggggcggga

14881 tcagtagtct gccgatcagt ttgtgccatg aaagatcacc aagtttcccg tgccaaaggc

14941 cctaggagct ggtgccttcg ccacaccgtc tcatagccgg cccagttggc cagtgagtgg

15001 tcctgctgga aggctctcct gggtgcccga ctagtccagt ctcctcccac ctgtcagatt

15061 acacaagtaa ctcagtgaat gggcttcccc tgcatctctt aggaagttca ggagccagga

15121 agcaaaacaa acctaattgc tggtacggcc cagcctcact gggcgtctgc acttttactg

15181 gtcgagtggg agggctgttc agaggggcac gtggagccac acggggaggt gtgtgtcaac

15241 tgtaaagggg cacgctaatg tcgctcatca ctgttgtttc cctagggtgt ggaccaggtg

15301 aaagcataca taccagatgc catctggtac gactatgaga cagtaagtga ggtggcgcac

15361 ttggcccaga gacccggctc tagcagagga ccgtgttaag gcagcatctg tgtttggggc

15421 ttggggaagg atgatactac aaggttttac atgtagttgg ttgtgggtac tcttccctgt

15481 ggaaggcctt tccaagggta cggtgaaata atttgcacaa ctggggtagg ttgatgggaa

15541 ttttcattat gtccacctgg ccactgtgca gctgtcagga ctagccattt ggacgtaggc

15601 ggggatagct cacggaacga aagccctggt agaatcacag cgtggggtca cacaaagaag

15661 tgaggtgggt gggagtctca gctttgctgt ctacttccca cctgtatgcc ttggacaagt

15721 cacttccctt tggcgggggc agaggggtgg gcggtgtctg gttttctctt atttaaaatg

15781 aggcagctaa gcaataagct ctccaaggtg ccttctagcc caaacagcca atgatcggcc

15841 cagttttcct ttgctggcta gggagtggcc acccagtgga ggaagcaatg ggtgcagatg

15901 ctgctgcccg ctgagaagat aggacttcac cttcgagggg gctgcatatt ccccacccag

15961 cagccaagca caaccaccga ggccaggtaa gctcctcact cttccaaggc gggactgctc

16021 tcaagctcca agacacaggg cattgtggga aaggtgaata ccgggactaa gtatggatga

16081 acccagggag gacatcccca gtcagggatc ctcctcccct cctttcaggg ttgacccaac

16141 attagcttct tagaattcac tcagtggcgg taaacgtagc ctctgttcct ttgcacgtcc

16201 gtagtctttc ccttatggat tgaatcggat tacaagaaga gtcaaatgat cgctcaggag

16261 gatttcttta tggtcgaaaa ttgccctcaa agagattccc ttagacatga gagaatgcaa

16321 gttaaatagg agccaaagtc aacatcgctt caaggtcaaa agtccgaaat tttgggggtt

16381 taagccccaa ttctacaaca taccagatgt gttacgatgg ccacttactc tctgtatttc

16441 cgcttatcac tcgtaaactg gggatgatga tagttttact tttagagtaa ttataaggat

16501 caaatggaat aacacacata gtttaatgtg tataagacag ttagaggagt tcctgacaca

16561 tggcaagccc ctaatggagc tcccttggtg gttagtgtta ttgcaaatcc agttttcaac

16621 gggagccctc cccttcaact ctgcttctta tgctcattcc ctgctaacct ctgctgtccg

16681 cccacgcccc acccccttgt ctccatacaa ggtcagtgac cacacagtgt ctgggagaga

16741 ctctgggatg acctgcctgt ggggcacgaa gggggctggt tgaccccact tcttatctca

16801 tcttccttct ctgaaactga gacctgctct tttccagtcg gaagaattcc ctggggctca

16861 ttatcgcctt ggaccacaag cgagaagcaa agggacagct gtactgggat gacggggaat

16921 ctgcaggtag acttccctgc acgccgtcgc tgcggtgctc cgtgagagca gtcactgcag

16981 gctgcccagt gcggcgccga cgctgttgtc atcgctgctt acttctgcag ctcacagcca

17041 gttcatgctg aggcctggtt ggccaagcgc aatgcattaa agtagacgtc aggtggtcca

17101 catgcatgtg attgactcta tccaagcaac agacattatt ggatttaatt agtctctcct

17161 ctgggtacca ttattaatac ctaataatac attttgctag tagttttgca aactacttct

17221 ttggttacta ttaagaggaa ggatctcatt tgtgtagtat gtttttccac ggtaggattt

17281 ccacgtgagc acctcacata agcctatggt ctatgattat atgaaaatcc aaccctgcac

17341 aaaattagca tttattaata tttattgact gtttatcatt gcactgtatg caggggatat

17401 agaggtcaat aaaacataga cattgcactt ggggatctcc tgctgcagca ggatagatac

17461 accctgcatg agactaacta caatgtgagt caaatgtttt caatgccagc gagatagtcc

17521 ctcatagtgc cacgggagct cggaggtgga gcacttgctg tggctgaagg cgctgggaag

17581 cctttgtgga ggtttgacaa aaacgggtaa cggtggctaa gagcgatccg tggcaaaggc

17641 actcccgcag aagagaaacg cctcttcttc ccctgtaaca gggtctcccc cgctctctct

17701 ctttcagatg ctgtaactgg aaataattac actctctacg agttttctgt cacctccgta

17761 agtactgctt ttgaggaaca tgcagggtac cttcttttct gtgactgctt ttgagttctg

17821 ggtttgctac agaagcttag ccatggggca cagggacagt gtctgattcc aacggccccg

17881 gaaagggtga aggttcaaat gggagttttt aatctgtgtt ggagatgtgt ctgtaagaat

17941 tcagtgtgac tttggtgaag aagtgtgttt tttttttcct ggaaaaattt ccttgactct

18001 gagttgtata tgatgagtcg tggcttccaa gatgtgccct tattcggcag ggggtggggc

18061 tgcaagtgtg tgtggtgggg ggggtgtggg tgggagtggg gagccgtgca gtgcctgttt

18121 gaggtccttc catgacgacc ttcctttccc gcccctaaac ttggccttgg gcacaggtat

18181 gacgtggcgg ttttgagagg ctgggagaac atcatttgca tcgtctgttt cccggggggg

18241 actaagagga agaaaggcag tcagcccacc agaggacggg agcattctcg tgtgggtttc

18301 tcggaagtct gattgttcga gggcagagat ttctttgatg gggatctctc ccttatttct

18361 tttcttttaa atttatattg tatttttttc ctattttact ttttattttg agatgcctgt

18421 agagccccgt gttgttgtaa gaagcaatac agacagatct cttttactcc tctcccagtc

18481 tccccaacgg tagcatctta gaaagctact gtttagtttc agaacaagga tgctcccagg

18541 atggagggca tttccatcac cccaggggtc cctcagccac ccttccgaag ccacacctat

18601 tgcctcctcc cctcccccct ccttagcccc tggcaaccac tgttctgtct gtcatttcta

18661 taattttgtc ctttcaagaa cgttctcgaa atggaatcat acagcacata gtcttgggga

18721 tgggcttttt tcattcagtg taattccctc aatagtcatc cacgttgttg tgtctacagg

18781 gtcctttcca tggtgagtag cgtccgagtc atagctgccc agctttggtt gagccagctc

18841 actcggtaga gagagcatct gggctgtttc tctcagggcc tgttccgaag aagccgctgc

18901 gaatgttgtg gacaggtttt tgtgcgaaca taggttttca tttctctggt gtaaaagtcc

18961 gggagtgcca acactgggtt ctatagtcag ggcatgttta gttttataag acagtgacaa

19021 acttttccag agtggcggga ccgttgtcca ttcacaccac caatgtatga gtgatgcagt

19081 ttccacgcat cctaaccagc atctgacgtc atgattttca aaggttattt tagcccttct

19141 tgtaagggtg tgccgacagc tcaccggggt cccagtttct ccttctctgt tgcctgaggg

19201 tgttggccat ctcttcaggt gcccacttgc cattcgtatg ttctcctagg caaactgtct

19261 atcccgctct tttgcccatt ttctaactgg attatctgcc ttatttttct gttttagttt

19321 tgagagtgtt ttttaatatt ttagaaaccg gtcctttgtt gggtttacgg tttgtaaata

19381 ttttctttca gtctgtaaat gtctcttttc atcattgtaa tgaagttttt catagagcaa

19441 acatttttaa atttaatggg cctgatttat cctttttgtg tgtgtgtgtg gatcaaactt

19501 tttggtgtcc agaataagaa ctcctcctac ccctagagct tgaatatttt ctcttatttt

19561 tacctaaaaa gtgcatagtt tgaccttttc acacttaagt ccacgatcta ttttgagtta

19621 tgtaattaca caacatgtgt aattctacat gtaattttta tatgaggtgt gaaatttagg

19681 ttgaggttta agttcttccc tatgttcatc cggctgcccc atcccatctg ttaaaaggct

19741 cttctttctc cgctccattg cttttgcacc tttgcagaaa ccagttgtgg gatgccaagt

19801 ctttttattt tttattttat ttttaaaaga ttttatattt atttatttat tttggagaga

19861 ggtgaaggga ggggggaaaa gaggaagaga atcatcgctg tgagagagga acatcagttg

19921 tctctcgcac acgccccagt cagggacctg gcccacaacc cagccatgca ccctgactgg

19981 gacttgaact ggcgaccttt cattttgtgg ggtgacgctc aaccaactga ggcaggctag

20041 tcagggcatg cctagtcttg ttaaacatca aagtgacccc ctttcttttc tttcgtcttc

20101 agctatacct tcttcatcat ggctcaactt gctcatgttt gaactatggt tctctgaata

20161 agttatctaa tttctctggg cctcagtctc ctcatctgaa aaatagagat caatatagct

20221 ctaacctcat agtttgccgt gagaattgaa tggggtaaca catgtcaaat gcttcaccca

20281 gtgcctggca catcgtacgc atttatatag ttacttctta tccaccttat cattgaaata

20341 aaaagagaaa ttatttgggc actatttcgt gtgtatgtgc atgtccatac cttcgtgtgc

20401 cgtgggtttc ccacgtgctc gagtgttttt gtttgctttg ctagtatcca tcttatctgc

20461 ttgaaaatcc cacgatgtca ggaacctgtt ttctgaagag gagaatcaat aaataaccag

20521 tgaggcaggg caagcacaaa ctgttactaa aagactgagg accaccagga gtctgggtta

20581 acgagttagc tccctgggat gaccccatgg caccgtgtag aacctcagcg ataaagggcg

20641 tgacttcagt ctcaggaaga aagatatgaa gtggcatttg ggcacagtta gagtcccttg

20701 ttttctaaac tgactttttt ttttcagaac cgtctacagg caaagatcac gactaataaa

20761 tataaggaca ccgacaacct catgtttaca gatatcacca tcttgggaat ggacaagcaa

20821 cctgctgact ttactctcct tatgggcgct tccaccccca ttgcaaatgt cagctacaac

20881 acatcaacga aggtgggtga ttgtcccgtc cccccatcct gtggggccct tgcagaaggc

20941 cctggactca gattctgaca ggcagctctc ccttgctctg tggaggtcgg gcgggcacag

21001 agtcgactag catgctggga aatctaccgc acacaggcag gacgcagagg gagaaacgtg

21061 tgcctgctga gcagggcccc tgcactctgt gtcagtggga ccccgaaaga aggcagttct

21121 tctctgggcc aatttaaagc tgggctgcgg accccacctg tcatacagtg tgatacagta

21181 ttcagactat attaattttt actttatttt ttattgattt gagagagagt aaggagagag

21241 aaagagacat tggtttggtg ttccaccaat ttgttgttcc aattatctct gcatttgttg

21301 gttgattctt gtctgtgccc tgaccaagga ttgaacccgc aaccttgatg tatcgggaca

21361 atgctctaac ctactaagct acccagccaa ggctcaaact gcatgataca atgcatcagc

21421 ccagttctag agggtattta cccatctgcc ctctcatttc aattaaaggc cctctttgag

21481 acacagctga tagttttcct tagtgtttta agcagtaatt gtatcattga agaaaagggg

21541 agttaaataa aacacaaggg gtagtccggt tttgtcccta ttgaatgatc ctcttacatg

21601 ataatctcgg acaaaagatt cccattgtta gctgagtaca tcaagtgtgg gaaatgcacc

21661 atttcttagg gcagcccctt ccatttagac aacacctgtg gcaaaggata ggcgccgggc

21721 ttctacagtt aggctgaatg ggattccaat ctaagctctg ccatccttga gctgtgagat

21781 cttgggcaaa gcattaaccc tctgcaaacc tcagctttct gttttacaaa tcagagagag

21841 ccgtatggag atagccaaat tgcagctagc caatttgaag tacgggattg ctgcagcact

21901 aatggagaga atgcatgcga agtagtcatc gccatgtctg gcccatacgc atgctcgata

21961 aaacttagtt tagctattgc cgtaactgcc atcatgataa aaaggtgtgt gcaccactcc

22021 tagaaggcca ttgcctaaca atgatggccc tgcccacaat ctgtacccgt taatgaagtc

22081 gaaggccttc ccattggctg ctccaggagg tgagaaccat cggaatgagc caatgagccg

22141 agtgcctttc cctgcagcga tgtgaaaagc cccagaggag aaacagtaat atgccaactc

22201 tcccgtgggg aagtagtatt gcaggaacag ctagctcccc caccccatcc tgacgtccag

22261 gccatcaggg aaggacgtca tccaggtgtc tctccctctc ctcatgtgtc accatggcct

22321 cctttctccc tccacaggtg gtgaagatca ctgatctcaa gggactggtg ctgggacagg

22381 agttctccat tcagtggagg cttccggtca gcgacctgga gaagttcaac tgctaccccg

22441 aggagcccgc cgcctctgag gagaagtgca gccagcgggg gtgcctttgg gaggtaaggg

22501 tgagggcaga tcaccccagc ggtggggggg aagtttgtcg gaactgccac acgagtagtc

22561 gggcagctag aaaacacttc caagcattgt tgggtagaac gggagtcatc cttcaacact

22621 gtgccgacgg gtgccttctc ggtgaactct gagcgagagg cagtcagtgt ttgcctcaga

22681 gagttggctc tctctttcct ccagctgact tattttaacc cccactttta tttttaacat

22741 aataagaata agaataacac accgtgcatt gagtatttgg agtgattttt ttccatccat

22801 tagagtccga gcaggaaggt aaggtaaggt atgagccttc ctctccactt gaaaaatgga

22861 gggaatgaaa gccagaggaa ataaatgact tcctcaaggt cacggtgggg gcaaccatcc

22921 cacagcaaag cagggtcgct ttgggaggct gggggcagaa cctgccgtag gaccccagac

22981 tccagggcag gcttcctgct gacccttcac tgctcatgtg ttacaggagg ggtgtgtgtg

23041 tgtgcacgcg tgtgtgtgtg ttaaatggat gcattcattt gctagagttg ccataacgag

23101 gttccataaa ctgggtgact gaaaactaca gaaatatatc acatcagtgt gttgagagct

23161 acgagttcaa agtcaggggg ttggcagggt tggctgcttt cgagggctgt gagggacagt

23221 ctgttccagg cctctctcct aggttctcaa ggtttcctgg cgatctgagg tgttccttgg

23281 ttcataaaaa cttcaccgca atctctgcct tcatctttac gtggtgtcct ttccccgggt

23341 gcatgtttgt ctctgtgtcc gaatctccct acttaatacg gacactagtt atacccgagt

23401 aggccccccc tagtgatctc gttgaactag gccatctgca aagaccctgt agacatccgc

23461 ataaggcaca aggtgtcagg acttaacatc ttgtgggggg aatgcagttc aacccataac

23521 aatggcgtgg cggcatggac ttagtctggg ctacctaact tcttcagtaa ttagagcccc

23581 agagtgtggt gttcaaggcc aactgggaga cccctcctct cgttccagcc caccaccgtt

23641 cctggcgtgc ccacctgttt ctatgacact atccctaatt atgctgccag taacattcag

23701 tatttgccca ccggcatcgg catggacctt acgcatctga cggcctctga gtccccacga

23761 gcagcagcac cgccaccatc accacggggc gcgctccctc tgcccgcagc agccgcgacc

23821 tctgaccctc tctctgcaaa gatcagcttc cttaagctca gcgtgaccta ccacacagaa

23881 agcatgctgc agttcaaggt aactccccgt gttgcacatt ctggtcctcc acagggtgct

23941 cctatgggtg gaagtcattg aaacatcttt ttgtccagat ccctcatttt agagatcacc

24001 gaggcccgga gaggtcaata aaggtcaaga aaggtcaaga aaggtcagga tggtaggagg

24061 tagtagggcc aggctgtgtg tggccttggc gccactaggg ttgtcagttt cctgcagcga

24121 tgggtcctct acatcagaaa gatcccccaa gaatccctcc tcagcccaga gaaaaatgtg

24181 cctgctttgt gataaactca tacgagtggc atcaacacac ttcccacgtt acagttgcct

24241 ctcctgcagc tgatgctttg gcttaacatg catcagaatc acccacaggt cctgttcaaa

24301 cgtggattgc taggcctcct gcccagggtt tcctgcccat gaatttgcat ttctgacaag

24361 ttcccaagtg aggcagatgc tgcacgcacg ttaaggaacc acacgtggag aggcaccgct

24421 ctgctcaagt ggaaattcag tgggaggcac gtgtgttttc caataaccgt gtatttaagc

24481 acatatttga aaaaattcag gtgaaattga ttgtaatagc atttgattca accaatgtgc

24541 taaaatgttt tcatcatgga atccaaatgc aattattaag atacttttga atcttataaa

24601 catagtaagt cttcacaatc tggtgtggat tttgcattaa cagtgagtct ccgttcagac

24661 tagcacattt caagggctta agggccccac gtgggtagtg gataccgccc cggacagctt

24721 gctctagacg ctgcttttca cattttaacg tgggaggaat caccgtaggg atcttgtgaa

24781 aatggaaatt ctgactcaga aagtctgggg gcggggccct agtttctgca ttgctaacag

24841 gctcccagat agtgttgctg cttttggtct gagaaccaga atttggagaa gcaaggcttc

24901 caacaaaatg tgtcctaggt gtaacaggtg ggctaatact ggtttgagaa aaagggtgat

24961 cgctcatgat ggcgtgacgg tgacactacc cgaccttgta taacacgtgg ccctctctca

25021 cttggcccgc acggcagtcc tgtgaggtgg gtagaagtga gtggtaggta cagaggagca

25081 gtcttcagaa gggaaaagct gagccacatc tggaagctca actagtaaga atagctcagc

25141 atttatatga cagctcagtc ctctgatttc tgccatacac acatgtgctc acacacacag

25201 ttccatgcac atacctgtcc acaggtcgca ctcataaaca cacaccatcc tgtctgtgag

25261 attctgtacc cttaaatgac aaactcttta aaggatgtgc cccacctggc tctgaacaca

25321 tgccactgag acatttttgc agttcaggct ctgcgctggg gacagtgggt gggcaggccc

25381 cacgtctgtc tgttcgcttt ggggcagttg gggcctcccg aggggaactg aattgtctga

25441 gcgggcgtga gggcccttgg gggtgagatt ccatcagcaa gtgtcctcct ctctcctcgt

25501 cagatctatg accccactaa taaaaggtac gaggtcccag tgcctctgaa catcccttcc

25561 tctcctgttg gctcccccga gaaccgtctg tatgacgtcg agattcaaac caatcctttc

25621 gggatccaga ttcgacgaaa aggctccgga actgtgatgt aagcactgtt tatttggttc

25681 taattcgagt gattccagat ctaattatgg tggttctaat taatgcaagt gaaatgttga

25741 ccttcgagac tatgtgtgtg tttggttttc tacagagaga atatctattt ccccctactt

25801 tttaaaaaag attttatata cttattttta aggaagggag ggagggagag agagagagag

25861 agagagacat caatgtgcgg ttgctggggg ccatggcctg caacccaggc atgtaccctg

25921 actgggaatc gaacctgtga cactttggtt cacagcccgt gctcaatcca ctgagctacg

25981 ccaacagccg gggctttgtt tccccctact ttgacgggga tttcattttc tcttagtcct

26041 taattcaaca agaaaaatta atcaatcata ggtttaaaag tactaagcga tgcttgattt

26101 cagaaaaaga aaaatacaca tcctactcat tgttcttgtt aagtagaaca caacattttg

26161 tttgccccat aaagaaatac taacagaaga gtcaggagag aagggcaagg gtcagtgagg

26221 agagagaaat ggtgtcttat ggtctttgct tctattgaca atgtagactt acagcttttt

26281 cacaacattg agtgtgaaag gaattgtcac ctctcacccc agtcctgctg ctgaatggca

26341 tgaacccaag gtctaggttt tgcctggggc cgaagctctc caccccaata gaaactgtcc

26401 cctgtatggg gacgtgtgga cagcctccgg ctctctttga ccatgtgtct gactttctgt

26461 ctgttgatgt ctttggtaag aagcgctttg gccaatccca aacagcctgt gtttcccact

26521 tttctattgg aacatttgct tggacattat tcactgattc gagctgctat gccaggcaat

26581 gagaactaga aatacagagc tgagggagac tctcaagagt ccataaatca gaaaacgagt

26641 tgtcagtctt gcaaaccaca gaataaaaat gcaaacacag aactttggta gggagccatg

26701 gccattcata aggcctgggt ccgctcacag tgtttgaacc tgggtccagg tgtgcagtca

26761 ttcagtgagc ttcgggatca gccagacatc ccgatcgtgt tccccgttgg ctctggcctg

26821 tgagcagctc agctcgaagg tgcgcctttg tgttgcagtt gggactccca gctccccggc

26881 ttcaccttca acgacaagtt cctttccatt tctactcgcc tcccctctga gaacatctat

26941 ggctttggag agactgagca cacagctttc agaagaaaca ttagctggaa cacgtggggg

27001 atgttcgctc gagacaagcc accaggggta agcacagaag agggagatta tcgatcagtt

27061 tctccctgtg tacatttatt tattattatt actaatccac acccgagaat atgtttactg

27121 attttagaga gagggaggga gagagagaaa catcgatgtg agagagaaca tcaatcagtt

27181 gcctcccgta tgcaccccag ctggggactg aacctataac ctaggcatgg accgtgacca

27241 ggaattgaac ccacaacctt ttagtgtatg ggacaaactg ccaaccaact gagccacctg

27301 accagggcac tctgccaatc tattttactt catacacggt accctattca ccaacttcag

27361 cttatgtcga taacttgtgc tgtcaaatga aataggccac atgttatata atgctcttct

27421 gttcataaat gccatttagt cttttagaat atatatatac acatatatat gtgtgatata

27481 tacacacatg tattttatgt gtactacgaa ttttacttta taaactttta gttactatgc

27541 tctatgtgcc aggcacacac agaataagac agtcttgttt cataagtcac cgtcttatgt

27601 cacaaaacag tgtctttgtt ctccaagggc ttataggctc atgagtaaga cgcagaagta

27661 gcggtggcca tgtagtcagt gtgataatag aactgtgtga gaatgaacaa ggagggcgtg

27721 ggggggatgg cacttggcct gttgcttggg gtggaaggga gggtcaagga aggcttcatg

27781 aagggtctga cattgttcta aatcttcagt gttgggtaga ggggctggct gtttgggtta

27841 taagatggaa tgagtttttt tggcttttcc cttgttgttt ctcatctact ttccccatga

27901 ttctcccagt acaagaagaa ttcctatggc gtccaccctt actacatggt cctggagaac

27961 agcggcagtg cccacggagt gcttctgctc aacagcaacg ccatgggtaa gccagaggct

28021 ccttcccgtg tccacttcat tgggatctgg gatatcaata gaaataagga catctggggc

28081 caaggaagga aatgggacac attacctgac ctttcattca ttcattcatt catttcttca

28141 tgaatgaaag agatttatta agatctctag ccccagtatt agaagtaaac ttctgactac

28201 acagggtccc tagttgtgta tgaccttatt ttactgtgac acgttaattc cctttcgtgg

28261 agggttataa aatgttgaga ctgagattat atttggggca cctgaaaatc cttctcggat

28321 cccactgcat gggagaagac tttctagagg agctgggcgt gaaaggtgga gagtgaaaat

28381 agcaaatatt agggcaggtt tcctgtctgg gtcatttata gttccaacga tgcacctcac

28441 ctgtttttgt tttttttttt ttttaacaga tgtgacagtc cagcccacgc ctgccctgac

28501 gtaccgcacc acgggaggga ttttggattt ttatgtggtt ttggggccaa cccctgagct

28561 cgtcacccag caatacacgg aggtcagaag tcatcctatt cattaacaca ccatccgatg

28621 cttatcgggt attgccacgt tcgtagcact gggaaaggcc aattttgggt gtcgctagta

28681 catcattact gtcttttctg agagtagggt tttagtgtta agggcccata aaagctcatt

28741 tatatcctat acgtctctta attctttgct acaaatcagc aattagcact gaagtctcag

28801 gttacctaga gcagcagctc taacatatag ggaggcttcc catcgtcctg cttccccgtg

28861 gttgtgcccc atttatttga ctttcttatt ttccttgaat gggtcgctta aatcagacaa

28921 tactcagcag acgtgctcaa gttccttcaa gcataagcca accatctttc cttccctgag

28981 aaacctacct agatccattt ttcttttttc ttctagttga ttggtcggcc ggcaatgatt

29041 ccgtactggg ccttgggatt ccagctgagt cgctatggat accagagtga tgctgaaatc

29101 tccagtttgt atgacgcgat ggtggaggcc cagatcccct atgtatgaag cacccccccc

29161 accccgccag cctgtggttt aattagttat gggaacctgt ggcttactat ggattgaaaa

29221 tatcttcata gaaatccctg atgactacct cagtacaggg gaaatgtagc cgtggagctc

29281 atcctctgtg gaaacattgc tttttaaagt tatttcatgc caccctttta aaccaaggtt

29341 tcaaaactga agggcaggag tcctccagaa gttagtggag gatattgtta cttactgcct

29401 cttgccagaa atatcctaca gaagccatag gtggtcttaa gccatgactc tggggacttc

29461 taagtgggaa tcatgtcaag attatgacaa ttttagacta tactgtgact atttttggag

29521 aggatgggta caaagtgaca atatgaaaga aagtagttaa aatcaagcct tccctctgac

29581 ttaaacgcaa atactcacgt atgtgaatcc tgtagtgaca acctggccac tagggaatcc

29641 ccgagaagtg cagtcagtcg ctgccgacaa gcagaattgc agaaacagca cgttctttcc

29701 tctcttgtaa tgatcgagtt cacaccccac attctctttc tctgctctta actctcctcc

29761 tgagatctgg aggacagtca atgattagaa gatgaacata aagtgttcca gtgttcccat

29821 caagggtagc tggaaacatt cacccaagct tatttttttt cctttttata tactactata

29881 taagatactt attaaacatc aattggaaaa gataggcaaa ttagaagatg aaagaaatcc

29941 ataggtctct ctcaccaagg caaccaatgt taataatata tattggttca ttcttttcta

30001 agctttggct tatgcccaag tctctgttcc ctcagacaag tttcacttag agtcataaga

30061 ccatacagat gattataacc ttcaggcgaa agaaaaggca tatctctttc ttcaaccagt

30121 accatatgtt gctgtgtata atgcattaat gcatccccgt gtttttgtgc acattgtaca

30181 cggggttatt atgcccatgt ataatgtgca tcctcatttt tccctcaaaa atttgggcaa

30241 aaagtgtgca ttatacacag caaaatatgg tgttaaccac tttagccatc atttgacaat

30301 cctgaaccgg gtacatttca tttgcagaaa cctaggttgg ttcgtgctgg gttgagaaaa

30361 gggaagacaa ggttctcctc tcaaagacca tgcagacaaa tggggatggt aggaagagta

30421 tactatcggt ctactttagg gtgctataaa gtcacatagt tcttaaatgt gaataaattc

30481 agtcagacgc agtggagggg aaagagaaaa cagatataca accttatctg tatttcagca

30541 gagtcctgct ttctttggac atttaactag catctttttt tttggaaggc tcactaagtt

30601 tgagatttga agaacggaaa agacaaattg agggaaggaa aggggagaga gttccgctcg

30661 gggaagaggg gcagaaggag gaggaggaat ggggacaggc gatctgtacg cgggaatcag

30721 gcagccattg taaccagctg aactgaacgc ccctctggtg gaggcaggag atgcattcgg

30781 ggggccctgg gctggggtcc acaagcagca gagcgggagc ctggtggagc agttggctgc

30841 cctgagggat actactgcgg gaccccaaac accctgctcg ccccttcctg taggatgttc

30901 aacatgcaga catagattac atggaccgga agctggactt caccctcagc agcagctttc

30961 aaaaccttgg tcccctgatg gagcgaatga agcaaaacgg catgaggttc atcctcattc

31021 tggtaggtgc ttgcgaagat gaggcctcat ttctgctcct gtgtccgtgg tttgaaaata

31081 gttccattgg gggagacgag gtgaagagtc taggacaact tcctgtggat ctataattct

31141 tttcttaagt ttgaaaagag caaataaaca aaggagaaaa gaacaaagag ggcttttaag

31201 aaaataatac ttacaatctt tatttttcgt gactgagttc tccttactct tggtagagct

31261 agaactgtgg gctaaaacac caaatatagc aaaaaaaaaa aaaaagtctc actgtttcag

31321 gctgtgcctt ggaataactg aatcttagtc tcatctttac tggaggggtt tcaaaaacct

31381 aactgtcatt atcacccaga cgtggtgaag tgcaggggac ttatgtctcc ttaatgaaac

31441 tcacaaacaa cctagtagag atccagaaga ccaacttaat agtttacatt ggaagtcaaa

31501 cacagaagta gttacccaca aatggtcctc ccttctagct tccatacatt agagcattag

31561 ttgacgactt ctgttcgttc attcattcat tcattcattc atccattcaa gtgtttatta

31621 agcacagggt ctttgaaaat cattgttcta aaaggagtat gtgtgtgtgt gtgatctaaa

31681 tgaatgaaga agaccatgtc ctttttctta gagggattat actttattgg tgggatacag

31741 ccaataaata aacattaatg atctaatttt gggtcgtgat gatttatggg accatcgtga

31801 aacaggatac ttatagaaag gaactgggca ggggagggat ggggttaaag gggaagatgc

31861 gcaagtctga tgcaggatag aagaaagaag ttccattgtc gtaaggagcg ttgctgtgac

31921 actgggacac tgtcgcaaca cagcatgttg cctttctctg tttgaagata ctgtcttctt

31981 cacctggctt atctctagga cttgtttacg aacactggca tcctttttcc cctgggaatt

32041 ttatagcgaa acatcaatgt attcttttac ttctcagcca cccagtagac ataaagcata

32101 ttatccatgg gctgagttgt ttgtttgttt gtattgaagt acaccatctc tctctgtact

32161 gtgaattgtt aaagttttgg agagcgtttt ccttggaaat cctttctcat gttttctgtg

32221 tttaacccct ttattgcagg acccagccat ttctggcaac gagacccagt atctcccatt

32281 caccagaggc caggaaggca atgtgttcat caagtggcca aacagcagtg acattgtctg

32341 ggggaaggtg aggactggca ggagatgccc ataattaatc agcatcacaa tgtcctaatg

32401 aaaagatgct cttaataaat cctggtaaag tatattgggt gacttataaa ttgagtgagt

32461 agaaatctgt ccaatgctga ttagcttaat agacattaat aagaccaagg tcataggtcc

32521 cgtctctaac atatcagtga gttttatttt gttgcctgga aatattatgg tatcccacct

32581 ctcacgtagg tcagtgggtc atttaacaca cataaaggat ctctttggga gcggtcctga

32641 actctgtgtc ccagtcaccc gtgcactcaa gacttgtact cacctttgac ctatgatgct

32701 tatcagcctt catatttaag taaatcacta actcttgtgt attatcctcc ttctttcacc

32761 tcctttaatt tcattttcag ggccctggtc ctcatcctta ttgcctggtg tagagcaaca

32821 gggttttagg tgaactccct gattccaggt gatagtggtc cttagccaag attcacattc

32881 cgctctgtag tgacattgta cactttgtct tgctctggtg tgtgccatgc ccgctgtgcc

32941 tttttgtgtg tggccttctc ccagcatacc caagtagtac gtgcactcaa gagcgactga

33001 aacattcctc tttaggatgc attcagtgcc tagtttcctg tttctccctt ttctctgttg

33061 ttgcccgcag agagtacagc atacaacaca gtacttatgt tctgtctttg aactcttaaa

33121 aaataaggac tgtttcttac ttagatccaa gtagatacag accatagaac agtgcctggc

33181 acagagaagt gcttaataaa tgtttcttga atgaattaat tcatgtatat agttaatgtc

33241 cccctaatta cagatgacat ggattgataa tgtggatttt tgttttttaa aagattttat

33301 ttatttttag agagaaggga agggagaaag agagggagag aaacatcagt gtggggttgc

33361 tgctcatgca ccccctactg gggacctggc ccacaaccca agcatgtgcc ctgactggga

33421 attgaattgg caaccctttg gttcacaggg tagcactcag tccactgagc tacaccagcc

33481 agggcaataa tatggatttt tttaatgcta catatacttg ggtttcccaa taagtcatga

33541 tgctgcttaa aagtattgag gcttagttca tgggttgtaa atagaaatcg atcttgtttc

33601 tttagttaca tttgttattt aaaaagggaa ttttgttata ggtccgtgat aaacttatca

33661 gagaactgag taatcgtttg aaaggtgatt tgttcagagg acttatttaa aatggattta

33721 gtcaatcatt aagttcaaaa gtaaaatttt ggtagcttac ggaatgagtc aagagaccca

33781 gcttattttc acctaatgga actttgttct tccaaacctt agaattcagt taatggccaa

33841 gatccctggg atttttatgg agatgcatgt cagtagagtg taacatgtga aacatttctc

33901 tgcctgtatg taatgccttg gaaaggtgtt agtttttcct gtttttctct aggtttggcc

33961 ggatctgccc aatgtgcagg tggatacgtc ccttgaccat gaaacgcagg ttaaggtatt

34021 gttacaaaca gatttttttt tgtctccatt gccctaaata tttaaatact cacaagtttt

34081 atgctgacgt ttggttaagt agctttaagg tgagtatgtt tgtcgtctgg taatttcttg

34141 gccgaaaaaa atatcgccaa atggaatact caacaagcaa gtaaactgga aatgcatagg

34201 aggaaatcat aagtgaaata ccttcacagg cgtgctttta atgggctgtt ttgtttgaat

34261 ctaatgttgc ttgaaaagta ctgaacccaa gccgagagta ttttcactgg ggagggcgtg

34321 cacacactgc ggggcagtga attgtcagcg tggaatccag tcggaaggag caacgtctcc

34381 tatcccagca cggctgtccc ttcggctcgg ccatccagca ccctcccact ccacggctca

34441 cttcccgtgg ttccgctggg cagctggtct ccttcatttg acccgtgaag aaggcagtac

34501 tggaatctct gaagtctcat agtttagaca cagacagtta attttccagt tcttacgatg

34561 aaaacgtcca ttcataaaag gaatcccatg tttaaaaagg actcaagact gtctaacctg

34621 ggaaaataat tcagccgtcc ctggtctcag tacctgcagt gaactctgtg caggggaagc

34681 tgcaggcctg gcttagggct ccccctgctg gggacagcac cgtgcctcgc accgcactgg

34741 cttgggcccc ttcgcatccc cgcacccctg tccctgaaca ggctgagacc cagtgtgagg

34801 gcctgtgttg ctcttacagc ggttccgagc ccacgtggcc tttcctgact tcctgcggaa

34861 cagcacagcc gcctggtgga agagggaaat aggagagctc tacaccaacc cccgggagcc

34921 gacaaagagc ttgaagttcg atggactgtg gatcgtaagt gcatccttag tggtttgtct

34981 ttggaaacgc cagcactcca aacccggaga ggcaattccg agactagcga agcagggagc

35041 taggaatgaa agagagggtg ctagcacaga attcattgcc aggaccacca gaacaagtgg

35101 agggagtcgc agggacatat tatctggagg aagtcagaga ccgtggaggg gcgtgccacc

35161 actggcaagg ctgccagaga aaaaggcagc ggataccctg gcttctctaa tctcccatcc

35221 tccaatctct tgccgctgcc tcccattggc ccttcccacc cggaaggcag cgatgtgtga

35281 gcctgggagc caccctgtga cacggggcag ggctccaggc aaggaaggcc cggatcttag

35341 acaaaataga cgtttatcac agtgctcgtg gagacgcctc cgatatttgt tacacgtgtc

35401 agctgggcgg aagagcaggg cttcccagga tgtctcttcc tgggacggat gtgcaaaggc

35461 tcttctgttt gtgcatttct agagatggga agtgtcgcta cacttccatc ccagtttcct

35521 tccccactgc tcagggaccc aaaaccctgc gtggggtcta ggccagctct gcagagcccc

35581 tcaggtctgg cccgggctgc ctgtgactca tgcagagaca gaagtccaag cccaccggcg

35641 gcagcgcagg gacttcccgg tcacttgctt taggacagat gggcaccaag gagacacgtg

35701 caaaagggca cgggcccacc caagcgggcc aagcacagat gggggagttg aatcaagagg

35761 agatttcctt aggccttaga tccagcttca tctcggtctt tctgccacta ggaaacaatc

35821 ctcagaagct gaaaactgta acctcaactg ggaattgaac gtgctgctta aaccaacatg

35881 cattcttagg gtccattcgg gcccgtgcct gagattaaac cgggtcaggg agtcaagcag

35941 ctgtgtgagg gcttaaataa gacacttttg tctccaggag gtcagcgtgc ccccaagcta

36001 aggccacacg ggctctgaca tcacagaacc tttcttgctg attcatttca ggatatgaac

36061 gagccatcga actttgtgga tggatctgtc gggggctgcc gcgatgaaat tcttaataaa

36121 ccgccttata tgccatgtat gtacaatgat gacctcatta agtgacctcc ccgctcaaaa

36181 tcccttgaac gctttcccct tgttgtcaaa gtgaagcctc attttggggt gagaaaaaca

36241 ttctccaaca cgagctaaaa tcccacatag aataaccttg aataaagtgc aagtgaatta

36301 ctaatcaagc gtagaaataa cgactcgatg tggcgtggaa gaaactgagc gatgtagggt

36361 acatctgatg acattggtaa ctatgaaccg atggctacaa ctgtaaataa cccccgtaca

36421 tacatgtggg caccctggtt gtcttatggt acgaacagaa acacagactc ctaggccttg

36481 aaggtctaag gcatctcaga aagctgagct ctctcgcctt acagagacat gggggaggaa

36541 gtcattcgcc ttcctaaaag cactgtgcca gtaatttgtg agatgaaatc tggaaatcaa

36601 gttttttaat gtctaggtca cggctatgct tcctttcctc cctccctccc tccttcctcg

36661 ttttcctcct tcctcccttg atttaatatt tgtgtatcag gcaaaccaga acgatttagc

36721 aacaccaata caaactcgat tgaaaccaga tctattccct tgagaacatt gccatctagt

36781 gggagacaca gtcatgacac caaaggagtg tgcctttacc agctccatcc cctgtcctta

36841 cccttcatta gttataaggt cacagaacat tgattttttt ttttttaaga ctaaagcaat

36901 tcttgaaatt ggagctgtta ccgttaacag agtcattgat tgaaatagag caatgccagg

36961 gtgattggcg tttccagtga atgggcagct caggttgttc agaacataac tcggcatctc

37021 ccctcagatt tggagtccag ggacggtggt ctgagcagca agaccctgtg catggagagc

37081 gagcaggtcc tgcccgacgg ctcccgggtg cggcactacg acgtgcacag cctgtacggg

37141 tggtcccagg ccagacccac atacgagtaa gtgtgctcag tcccttgcag caaggatcat

37201 tttccacatt gttgctgttg tcttgtcata catagaggga tgcgagttta atggatgaag

37261 gtcaagaaga agcctgcctc aggttagcct ccccagtccc cttaaatgcc ttgcctctgt

37321 gtctctcacc ccttgctaag ggggtgagtg tgagaaggct caccttcggg cgtccggcag

37381 tttgtaagac agccttttac tacctgtctg cccaggcctt tgaattcagc tggaagtcag

37441 tccctggaac cttctccttc cctggttctc tcttaggcat gcacaaagcc ttgtgcttgc

37501 gtagccttag gtcctcagga atacatggta acttttctaa ttctccactg caacaactgt

37561 gcacatgccc tctggcccac cagccctgct gcgggcggtt tggactgcag aggccccagc

37621 acctggtcct ctttctggcc agcctggggc tcaggaaacc ctttcttcta aaaagctttt

37681 ggctgtgaaa gttttggttt ttttttaacg ggttctaatt ctctgggact gacttttaaa

37741 ttaccagcca agcttttgtt tgcccccact gaaaccacag cctgagtgcc tatgatgttg

37801 ccagaaaatg gttattgttt tagttagtcc ctgtggtggg gggagggggg agggaggcgg

37861 ggtagggaga gggggcagac tttttctctc tgccgagttg aactctgagt ctaatcaaat

37921 ggccacagca ccctgggaac aacaattttc ccgaggactg gaagttagaa ataaaaaatg

37981 tattgtgctc ttgggatggg gcttttacca gggccacaga aatggccatt cctgtccgtt

38041 tgctgcaggg ctcctggctt ttggtgccaa cgagccaggg gcgggtcagg ctggaaaaag

38101 gccaagttaa aatccggtat aactctctgt cttgccaaga tttagtagtt tctcctggat

38161 agacaatttt ttttcttctt cgtatggctg gttaatttct aaagttctga actggtcagt

38221 ggcagagttc tctgcatcac tcacatccat ccttactcta catccctgcc tatggcaggc

38281 ctgtgttagc ttttactcat acgtgtgcac gcgtgtgccc agtctccatc cattttttga

38341 atttattttg ctgtgaaata ttggaagtga cctaagtggg ttagtctcca ctacactcgc

38401 tggcttcttt tccagacatc atttattgaa caattcatct gtgtctattg attggtgatg

38461 ctttatttag caaatatttg gattgcatta tatcctagca tctgttaata gactttccct

38521 ttggttccat tgatttttct ctcacacaaa ttgtttttat tctctgactg aggctgcgtg

38581 cagaaggccc accttcaatc aaggaatatt taaacatcct ttctgtatgt ttgtctctgt

38641 gcgcaaacgt gcctctgctt tttgaggagc tgcgtaggag agtgaatatt accaaaacaa

38701 aaaaagctat agtcatatgt acttttattt atggaccata tctgcctcct tgctcttcaa

38761 gtgacttttt gacaaatgat cgaacttccc tgagcctgtt tctgaatagg acaggaaggg

38821 aaggaaaccc tccttgtgag gtagtgagac agggacacga aagtgaccgg tataatacct

38881 ggaacagagt aattgctcca taaatgcatc ggccctacac tccacccctc ctcctactct

38941 tattgacggg agagacctgg ttcttcctgc tgacacatgt aaagaattac atatcagcaa

39001 atctagtagt gtgtaatcag tttattagtg gtccgcttcc atggaagagc acaggactgg

39061 gtagagtagg gggtgaaaag caaagtttca gggcaaagca gggtgagaaa agcaagagag

39121 gctggagacc aggcagaggg aagcatataa ataattaaca aagtgtgaca agggtttccc

39181 actacataga ggatggccgt aaagagatgg cagtttattt gaaaagaaat aaatagaaat

39241 cctggagctg aaaagcacaa taactaaaat aacagatttc ctggggggac tgagctcaac

39301 atcaggtctg agctagaaga agaaagcatc agcagaactg aagataggtc catagccgtg

39361 atccagtcta aataacacaa agacagagag agagtgaatc ctttgttctc aggacttgta

39421 tagttggtgg gagaggaggg aaaaaagtta catattgatt agcgggtaaa tgtggcgcca

39481 gctttcctgg gggaatgtga ctacgcaaat ttagggatga atgggggtct gtccgcccaa

39541 atccttatct tgctccagac tccatttggt catcctgttg taaaacaaat acatgacagg

39601 aggcagttaa ttaaaattgg ggcactttcc caagttattt atgggctctt tctgtaagca

39661 tcagggactc cctggtaaaa cagatagtga ccagaggtag gcaattaacc acagttgttt

39721 tatgggctgt ttctttgggc accggggacc ctctcccaca ttcaggcctt gggataaaag

39781 cacagtttca aggctttctt tccagatagt ggaaacgata catagaattt caaggactta

39841 cttttttgct aatatagtgt ttcttgtgat attgaaacac ctggcaatgt tatcagcaca

39901 ggcttaggac tgctgagtta gcatgtgaga cttgtcccct tcctcctccc tgcctcggtt

39961 tactatttat gctacctaat tggtaaaagg cgtttcctgg caaccagggt gctaggtgct

40021 ggccagtgcc agtattgcag tctgagagtt cttcctatgc tgtctcctgc ttcactttca

40081 tgtggacagc tacttaactc gatttttttt tttaaagaaa ggaaatccac ctgttaggtg

40141 atgccctatc tctggttgtc tggctctgaa gttcccccgg actctcagcc tcactttcaa

40201 atagcaggtg ctcgagcaaa gaggctctct ggctaaaatt gagccccgct gctgccccgt

40261 gctcagggct gaagctttgc tccttagtga agttccctgg ctgtccagtc catcctccaa

40321 ctcacctccc tgtgcccctc caaccagagc tgtgcaggag gccacaggaa agcgaggggt

40381 cctcatcacc cgctccacct tcccctcgtc tggccgctgg ggcggacact ggctgggaga

40441 caacacggcc gcgtgggacc agctgcagaa atccatcatt ggtgcgtgga catcccgggg

40501 cctgtgcatt gcttctgtct aatcaccatc cttgtgggca ggtgctgagt tctccggggg

40561 cgtgagtttc agtccacacc catctctttg tttgctcgcc acgactgggc cggggctttc

40621 gaatggggta cagaagagac agggcacatc cgccctccca aagggtgcac ggtccaacgc

40681 agctgtgctt ctcattgcag gcatgatgga gttcagcctc ttcggaatac cttatgtaag

40741 tcctgctgga gccgttgcca attgcccagg tggattttag ggggtctgcc acgcgcgaca

40801 gtcccgcctc acgccccgat tttgcctctc ctcagacagg agcagacatc tgtgggttct

40861 ttggagatgc cgaatacgag atgtgcgttc gctggatgca gctgggggcc ttctacccct

40921 tttcccggaa ccacaacacc atggggacga gggtgaggca gcagctggtg ctgcgggtgt

40981 tgcgggtcct caagtcacga ctttgctttg gatgttgaaa aggaccgtga ttatttatgg

41041 aggaagctcg tacagatcaa cagaccctcc catttcttaa gaaaatccac cagttcaaaa

41101 ccccatagta accaatgact tttcaagaat acactttttc tcatctcctg gtggaattgt

41161 tgacggtgcc cgccctatgg ggctgacata aaagaagcca ccttggcctg gaagaagagt

41221 gaatatgtgt tttgctccgg tctgtccaag agatgttagt gcctcccaga gacccatccc

41281 tctgcgcatg tgcagggcct gagcgagggg aaggggggtg ggggacgggg gacaaagagg

41341 agggagcaag agactggggc ttagtaagaa ggcgcgagtg gggggagggg caggctggaa

41401 atgaagggag gcgtgttgtg agtctaccta aggggcctgc gccttctggt acttctctga

41461 ggtccctagg acagagtgaa aaagaagcgc cctttcttat gcacctttat gcccttagag

41521 acaagatccc gtggcctgga attcaacctt tgcgatgtac tccagaagcg tcctgcagac

41581 cagatacacc ctgctgccct acctctacac cctgatgcac aaggcccacg cggagggcag

41641 cacggtcgtg cgccccctac tgcacgagtg agtgcggcct gtttccccca gcggcgggtc

41701 tgggcgtggc ctgtgagtga ccctccgcca ccgcagtctt gacggtctga gtagactttc

41761 tgtacctact tttaaaggtt cagcgaggac agaagaacgt gggacataga ccgccagttc

41821 atgctgggcc ctgccatctt agtcagccct gtgctgcaat ctgtgagttt tccgtcctag

41881 accgcaaacc ctccaaccgc gacctaagga acaggaaggg cgttgaaaga ccatttgggc

41941 cagatcacta tttctggaat atttggggga tttttttctt ctttttttca aggtctccgt

42001 taatgaaaac ttaatctcag tggatttctt tctttttttt tttaattact ttttaatcct

42061 cactcattga ttttagagag agggaaaggg agggagagag aaagagaaac attgatcagt

42121 tgcctttcat atgcaccctg accagggatg gaacctacaa cctaggtatg tgccctgact

42181 gggagttgaa cccatgacct tcaggtgtat ggaatgatgc tccaaccaac tgagccacac

42241 tggccagggc tgatttcttg ttttaatgtt ttgtcacctt ccatccttat aaagttgacc

42301 tttttaaaaa tttttatttt tctgtatatt tgaaacaaat gtcccaggat ctttctcaac

42361 aaaacctccc aaactcttaa aattcattag gaaaattccc atttttatgt gaatgctaca

42421 gagagaattt tttaaaaatt aaaactggat tgttgagata ttcacatgga cccattgatt

42481 ttctattcca gaacacgttt gagatccaag cctatttccc tgcaacccat tggtatgact

42541 atagcacggt aagaacaagc ggaaatattt tgtgaagaac cggggtggtc catgcaggct

42601 ggagaggtgg ctggagtttc ctcctggctt ccagacaccc atttgatgag gcaatagcgt

42661 ttaacctttc agcgcggtac aaggtttctt taaacaaaag tatccagtaa agcgtaactg

42721 ggtcagatct gactcccaca tcaccctgag tcctccccca tcccccatcc tttcctggag

42781 aataaagaga gaagaccaga tttccagagt tttcagcaaa gctgaaaagc ggagattctc

42841 ttttcgattt gcagtgtttt gctgactttc caacgcacgg ccttggggcg gacggactgg

42901 tgaggaggca tacatttgct tcccctgcca ccgacccttg cctgtcttcc ctctccactc

42961 cagggaattg gcaacacctc gacaggcgag tggagaaccc tgcaggctcc cctcgaccac

43021 atcaaccttc acatcagagg aggctacatc ttgccctggc aagaggcggg aatgaacacc

43081 cactccaggt gagggaggcg ctgtgcttca cgccgcatgc tcgcaggggg gtgcccgcag

43141 caccaggggc tctgtgcaaa agtaaccctg gtcccaggtg gttcactcct ccgatgctgt

43201 gagcaccctt tgagcgggac ctggaggagg acaagctcat catggtggcc tcagggagac

43261 agtttctgtg gagctctgtg tcagagggcg gggcaaaaaa tttgtggggc acagcagaac

43321 ctctttcaga ggtacagaaa agtacctctt tctgtactat taaaaatctg ggacatactg

43381 ctgagctctt tgatctgtga tggctatcca gtgcgcattc cctgttctga aagccactgc

43441 aaggtttctg tttgtttgtt tttttggggg gtgtcttctt cctatagaga agtagttttt

43501 catcatatta cttataatat gtaagtaaag gatatgtatt tggtttttga aatgtttcat

43561 taaactttaa atattatttc tattaccttt gaattatctt gtattttatc tcgtattaca

43621 ttttttggag atttatgtgc attttaactc catcgccttt cttttgttgt cctggatgaa

43681 taataatttc aaaagatgct gtcttttatt cttacaactt actatctaga gaaaaataac

43741 ttttctgttt ccatagtcga caaaaattga tgggattgat tgttgctttg gatgacgctg

43801 ggggagccga aggccaggtg ttctgggacg atggagaaag catcggtgag ttggagcttc

43861 ctgtgttctc cacctgccac tcagagaaac cgcacccctg tggttcggga tgtaatcggc

43921 ataaactgag aatccatctc atttctgctg ctgttagtaa agtagtggca ttgggagaat

43981 ttttgcacca tttacctgct tatctggata ttacaccgtc attattatag aggagtatgt

44041 gacccccagc tttgtaggtt cagcagactg atgtcgtagc ttcagctggg gtgaactctc

44101 tctgtgtaac tgtctcactc tgatgaaaaa acaagtgtgg ctgcaaatac caggctgaca

44161 gctcactcag tctcgctggc ccgcagacct ggctgtatga tttgggccgg ccgtgtgtcc

44221 tcagccaggc ttttcctcct aagcagggcc acacgggaga caaatcaggg cttagaaact

44281 ttcctagtgt ctcagaaaca gggaattgta tattgtcaga aggatacctc catcaaagaa

44341 atagtactat gtgtgcttcc tttcctcaga ctaggtactc gatagatagt tctactttag

44401 ataattggta tttgtcgctg taatgaaaac atcctcatcc tgagacgttt ccatggaaat

44461 actgatgcac tgttaaccaa ccaaccccat tttccaatac ttattactgt ggaccaccct

44521 gatcttgtaa tatgtcacat gtgccaaaat tatagcttat ttttcactag tagtatttct

44581 aaaggtttcc tattaacgcc actataatta gcgtattcca ctgagagatt atgccataat

44641 ttacctgttg ataattattt tctatgcagg atacaggata ctttcacttt ttatttttgt

44701 aaataacatc ctcaagtatt aacctttctt tttacttcat attatttcca tagaatagat

44761 tcctagaagt gaaattactg tttttttaaa aagatcaact ttattgagtt atatttcaca

44821 agttaaacgt atccatttta aatatatagt ttaataagct ttggcaaatg tatatagttg

44881 tttagccatc acgacgatca aggtaatgga atctttttat aaacatggag gcttaattac

44941 acagacagat aacatgtttt aatatataca gaggactcct ggcccatgaa tacagagaaa

45001 agtatcatta tcatgcagga taaataaacc tccattacaa gctgttaagg tgagatagtt

45061 cacacgtaaa tcactgtggt taatcttaca acagaaagtt ccaacgtcag agaaaggaga

45121 tccaggtttt acgggagtgg caggacccaa gcctcgtgag ggagggatat acccccggcc

45181 atgttttaca tgtaacagca gtgctcacta acggactaga atgttcccac cgactgggaa

45241 atgcatattt ctccttcagt taaagatgaa atcttcatca aggatttgtt tggggagcag

45301 gaattcacag caaaaagagt cagtgtccat gatgaggtgt tcagcactct gtcgcttcag

45361 agacacgctt gccaaaggga ctccagacga tttgcatggt cagatcatgc caagaacata

45421 cgtgggtgat gaggcaggaa gtactgggtt ggccaaaaag tccgttatgt ttttttccat

45481 atgatgtctc aggtagtgct tagttgtttt taactttatt caaaaaaaca attttgttag

45541 attgtgttgt gacagctaca cacaatattg tgtcatatta gcgtgaatta aaaaaaactt

45601 atcaaaaatt ggtgaatttt tgtgcagcca ttgttatatt gaagatggaa gaaaatatgc

45661 aacattttca gcatactatg ctttatcatt tcaagaaaga taaaatcaca agtgaaatgc

45721 aaaaaaaaag agatttgtgg tgtatatgga gaaggtgcta tgactgatca aacatgtcaa

45781 agtggtttgc ggagtttctt ggtaccactg acattttggc caaagaattc tttgcagtgg

45841 agttgtctta tgcattggaa gatgtttagc agcacccctg gcctctcccc actagaagaa

45901 atagtgggag atagccaaca tactcaaaat atccaaacca ataaagttat tggtgaaaat

45961 ttaaaaatat gccttttatt ttacagaaaa actagacaga gtttttggcc aacccaataa

46021 ttccatcatc agggtataaa atgtgggcaa taaagccagt ttagggaaga atgaaatatg

46081 aggtcagctt ggcacactgg gggtctgaga tgcgtgtggg gtctcctggc tggagagaga

46141 caggcagagt ttggacaagt gggtgtgatg attggcttga aaccctggtg aggagcggag

46201 cagcaaacct gggagcatag acctgggagc ataatgacat gtaaaaagca tacactgtgg

46261 gtgcgggctg caaaccaaag tgtcgcaggt tcgattccca gtcagggcag atacctgggt

46321 ttcaggctac ggctcccagc aaccacacat tgatgtttct ctctctctct ctttctccct

46381 cccttccctc tctaaaaata aataaataaa atcttttttt tttttttttt aaagcgtaca

46441 ctggattgta aaggtgaatc agtagaaatt ttttggaaaa gcatattttt tatgaaaaag

46501 aaataccttc ttttcataaa aaaactaaga ccttagttct tggtcctgac tttaactctt

46561 ttttacaata gcattattga aatataattc acacaccaag taattcagcc atttaaagtg

46621 tacagttcat ggtttcagtc tatgcaccaa gttgtttaac tattatgcag tcaattttag

46681 aacagtttct tcacccccaa aataagccct gtacctctca gatatcaccc ctctatcccc

46741 cactaccact attctctcca accccacgga acatatgcag ccctaggcat taactaagct

46801 actttctgtc actgcatgtc tacatatgct gcagatttca tataaatgga atcatgtgga

46861 gtcatgtaaa tgcaagaact atggcctttt atgactgact ggctttcact taacataata

46921 tattcaaagt ctcccagtgt ctctatgttt aatgttttgg gggaccccca ggttccttta

46981 cgaagaggct gccccatttt acattcccac caacagtgtg tgaggggtcc agattctctg

47041 catccttttc aggactcgtg atgttctgtc tttgagaata gccatcctac tgggcgtgaa

47101 gtaatatcac attgtggttc caactgaact gcattcccct gaaaactgat ggcatcgagc

47161 atcttctcat gtaattactg gccatttata tatcttcttc ggagaaatgt gtattgtagc

47221 ctgtgccaat ttttgaatta ggctgccttt ttattattga aatgaaagtg ttctttatat

47281 tctagataga tggtaagtcc ttgattagat ttatgatttg caaatatttt ctcccatctg

47341 gtgtattgtc ttttcacttt attaagtgtc catttggaac ataaaagttt ttcaattgga

47401 tgaagtcaaa tttatttttt ttattttgtc attggtactt tttgtgtaca gtggccaact

47461 ccagggtcac aaagatttac cctttttccc taagagtttc atagttaaca ctattatgtt

47521 taggtctttg agccattttg ggtggatttt tgtatatggt gtgaggtagg gggtccagct

47581 ttggtctttg cacatggata cccagttgtc cccatctttt tgtgggaaga ttattttttc

47641 accattgaat tatttttact tttggtaccc ttgttaaaga tcaagtgatt ttaagtgtga

47701 aggtatatat gcacagttga agacacagat aaaaccttca cattaaaaag attatacatg

47761 tatatattat atataaaaga ttatatatgt gtgtatatac atatacatgt gccggtacca

47821 ctctgtcttg agtacagttt ctttgaagta agttctgaaa ccaggaagaa agagtcctcc

47881 aactttcttc ttttttcttc ttcaaggaca cggctttttc agagcccgag ttctcagtct

47941 caatagtatg gacattttaa gcgctataat tcattgttgc tggcgatggc tgggctgtca

48001 cttacaagat gtctagcagt ttctccaccc tctctgccca ctagatgcca aagtattccc

48061 ttcaacccca agttgtaaca accaaactcg tctctagatt ttgccaagtg tttcaccagc

48121 aaagtcactg ctagttgaaa ccagtgtttg atgggcatct ggagtctttt tatcagcttg

48181 gcctgatcag gactagggat cgatgtcatt gcgggtcaat aaccccacag tcagagtgta

48241 aaatcagcct cctatggaca agagacaggg tccagcaaaa atgttgcccc tttcttttta

48301 ttacaaagtc ataagcatgt aattctgtaa cataacaata tcacactcaa gcataccata

48361 tggcatttta gttgacatgt tcaaattaaa actataaatt attacaccca gattatgacc

48421 ctaccaacca cactcaagca ggcattactt ctgctggacc atgtatttca aaaatttaag

48481 agaaaaatca attttctata aaattttagg tttctacaag gaataggttt tagtctcaag

48541 gttaaaaatc aatagtccct gaaagtttta agctttttaa cttctataga gaagctctct

48601 gcttcaccaa acagactcat acattcaaat ttaaagtaac aagatacagt gtaagtgtaa

48661 aattaatagg aagttttcat taacaggttt gtaactgtta agcttctcct tacttacata

48721 aggagatttc atagctagtt taaggatctt aaatcaccta tgttaacaga atctcattct

48781 ttaaacattc ttttttttaa aaaaagattt tctttatttt tagagaggag aagggaagga

48841 gagagagaaa catcagtgtg tggttgcctc tcactcgccc cctgctgggg acctggcccg

48901 caacccaggc atgtgccctg actgggaatc aaactgacga ccccttggtt tgcaggccag

48961 cgctcagtcc actgagccac accagccagg gctcattctt taaacattct ttaaaactgc

49021 taaaataggc tataatattt gcttaggttc cattttctag cagagactag atttaaaaat

49081 ggccctttat gattgtttgt ttccacaaga gtggaggtgc tttagaaaat gaagacgagg

49141 atttgctggt tcagggagag aacgcgttgt aatgtcggta agcggtgggg ccaagggcca

49201 aggtgatgac aagggccaag acgagaacgt gaagtgacgc tgacttttag aaagcaggag

49261 gtatagaggc ctcataagta ttatggttta atatactgca tcactggtga ctttgagagg

49321 atccaactca gcaaaaaaag gacacgggag ctaaagcata ggagatggag aattgaatta

49381 tgacttgtgt atgcagacat agcaagcaga ggcgactttt tttttttttc tcagacctat

49441 ggccctagat aggagatatg gtaacttgaa acagagagaa aaaagtcagg gatctgtcag

49501 aatggaatgc tctggagctt aattggacct tgaggagtag gtagggatgg gaagaactga

49561 tggagcaagc cctggagagg tggcggggcg gggggcttgg gatgtagagc actgtgggag

49621 ggtgcactaa gggtagggcg agaataataa agatggagac gacagtgact ttggtggtga

49681 cagtgaacca caaattttga gcactcactg tgcacgaggg catggtatat gaatcttatt

49741 gcccattttt tttagaacta gtcccatttt tttaaatctc ccttttctgg atggtaaaat

49801 gttaaggaac ttgcctgagt ttgcacgact actaactaca agggtgggga ttggaatgtc

49861 agtctgactc gaaagcctga atccctaact gctctcctcc gcttcggggc cgtctcggca

49921 cagccgtcgg aattctcatg gccacgggct tgctgtgtgg tcacagaaga aaaagatcag

49981 tagtcttcac acttcttcac ctgtgatagg agaccttcgt tcaacacagg ttctcgggtc

50041 tttgcaacca cctgaccctt acccccgaac accgtctgcc tcatgcacct ccccgcgctg

50101 acctggagcg ggaggttccc ttggctcctc ccatctgtgc tccctggaga agtcagggtt

50161 atcctgacca ggaattgcga tcccatggag acactttcag actctgtgta agagtatctg

50221 ctacggctga aggaatgagt agttgcttat ttcgtttctt gcttgcagat acctatgaaa

50281 atggaaacta cttcttggca aatttcacag cagctcaggt aagactttta aaatctgatt

50341 ttagaaatcc tctttccgtt agcgacagat gaactatgat ctgtgagctg tgtaaacata

50401 gtatcccacg tcttcatggg aagatccccc ccccccaccc ggctattata aatcaggtat

50461 atggatgtta aaacagggaa gaaacagccc ttgttttgat aattatccag tatgccatca

50521 ctctataaaa agaaaccggt acttcatcta atgctggggg gaagtgagtc ttttgtacat

50581 aacagtgcct caggcatttc atggtgtctg cctcatacac tgtctgctcc ttcttcttgg

50641 aatcacaccg cccaggctaa ctccccctgt tctgtgccta aactagtttt cagatccgat

50701 gattccattt ggattactgt tagtacagat tttgcttaaa aatggtaccc aggatttaga

50761 gaaattctta gagctgcatt atcccatatg atcactctgt tactatttgg tatcagcaga

50821 tttattgttg ctggtactca gaaagtatgt cttcatgaaa gtgtttaatt aaaagtaaaa

50881 cagcaaacct gcactatagt aaactgaggt ttcttaacat gttagcgtgg aatatagttg

50941 atcaacagat gtaatgtgta tttttcactg ttatgcattc tctatagaac acactgcgaa

51001 tccagaccgt gcacaataag tatttgagta actcgaatcc actgaaagtt ggaaatatta

51061 caatttgggg ggtctaccct acttacgtga cacaagtccg tgtcacctat gacaaccagc

51121 agtctgtggt gacgaatttc atcagtgacc cttaccaaca ggtatgtctc aaaataatgt

51181 tgttatatcc tacgttaatt ttgttattct gcaaagttat taccactatt attatcattt

51241 atctttgaga ggttataggt gtgaaaagtc aatatgttcc tgaaaagttg tgagtgttga

51301 attatattgt aaaatataaa atgccagtgg ctctccccag ggagcgatgg ccaacagtct

51361 cctgagatcc attccacgtg tgtcccaaag aaactctgct gtttgttgag gctggtctcc

51421 caggctagca cgacaagact tacgcctgtg gcagaggctc agacaaagac aggggtgatc

51481 tttaaaaata aaccgaggtc actaggtgtc cacacagcta gggtaaaagg gcagcagaca

51541 catcggtctt ctgggttggt gagaactggc agtgacctgt ggtgactgtg aagatgaata

51601 cgtcccagcc gcgatgtgag atcagttatc tggtcagcag aacagtgagc taggcagcag

51661 gccctactaa accctctcgt caagggtgct ttagacacca cagccaacta gctggaagac

51721 aaattaaaag tagtgttacg aagagcaaca gaagaatagt gggtaactat tcttacccac

51781 ttttgaacac tgcttgatct ttcataaaca cagattcagc aactcagaga gggcgggtta

51841 gaaaagtgaa catgggtaag aatagttact gttttctaaa aggtgaaaaa gttgccacgt

51901 tggtatttga attttgggaa acaacgctct gcagttcaaa cagattctct ctctctagtc

51961 tcttgtagtt ctccacttca aaacatgttt cttgaactac tgagttcaaa accactcttt

52021 atacgtgacc attgctcctt aagactgata gcagaagacc tctgaacacc tcctttttgg

52081 gaaagtgtgc ggcataccat acctgttcgt tttgtgctgg tctccacctg tcaataacca

52141 aggcagtgac cagactagct cttgtggttc ttaatacgaa tgtaagacgt tattcagtga

52201 taaccatcag gaaacttggg agagatacag gcttatcttt cccgtcctgg aaaccacaca

52261 gcccccgggc catacaagga ggtcctgagc agagagacag agagagagcc tgggcatggg

52321 cctcgggctc aacattttgg ggggcggagg atggggaggt ctagtggttc cagggatcac

52381 tctttattgg tgaatttaaa acagggtgaa ttttgccctg accagtgtgg cttggttgga

52441 gcgttgtcct tgcacaccaa aatgtcgggg gttaggttcc cagtcagggc acatacctag

52501 gctgcaggct ccatcccagg ttgggtgtgt atgggtgtag ccaactgata tttctccctc

52561 tctcccccca tctctctaaa agtaataaat gtttaaaaaa taataagtaa catggtgcat

52621 ttccagcctg ggaagagaaa acacacaagt ggcccaaagg tcagtcatta aaatcgacga

52681 agatctggaa aacaaaggag cctcaacggg cttgcgatgg gtgtcccttt ggaatgggtg

52741 gatctggcca atggagccta agtcagatgc acataataaa aaagaagtaa aacccaaaga

52801 gcagggctca cactgcccta ctatgtgccg ggcagtctac taggggttgg tgggaacagg

52861 aaagcagcct gccctcagga aggttaaaat ctagcggcaa agccaagaga tctttgtgaa

52921 cattgattct atcaaaggct caattacttg ggagtcgcgt ggttctaact aaatgaagtg

52981 cacagtagga gacgttccca gcacatctct ccgtatttag ctgtctgact cagtgtggtg

53041 taggagcgaa ccttctatta ctccctgggg gaaaaaaatg atacatcatt ttgatggtgg

53101 ttgttaaatg tggtatttaa gaagttctaa aatactaacg ttatctcttg ttttctggca

53161 gatactaaat attcaattgg gtgacaggat tatcagcctg gaaaagttaa ctgaggtcac

53221 ctggactcac ggtggtccta tagcttctac tacaccgagg accagcacca ccttgctgac

53281 cactccacat tctacattta ctactgagac tgccacttcg gggactattg tcactagttt

53341 gtctactgta actaccacta ctgccctgct tagcacaacc acttctttcc caacgagtac

53401 gatagaattt acaccagata ctactgtccc taatactacg actgctatcc ctacaaatac

53461 tactgctgcc agtactagta ctgactttcc cggtataagc acttctttcc ccacaagtac

53521 tattgaagtc acacctgata ctactgttcc taacacaact agtcctttcc cggtaaatac

53581 tactacggcc agcactagta ctccctttcc tattacaacc acgatttttc taacaagtac

53641 tcctgacctt acaactagta ctgctattct caatactaca acttccttcc ctacaaattc

53701 tactaccgct aacactagta ctgactttcc tggtataacc acttctttcc caacagatac

53761 ccccacagtt acaactagta ctactatttc taataccacg actcctttcc ctacaaattc

53821 tactaccact aacactagta ctgactttac taccacagcc acttctttct caacaggtac

53881 tcccacagtt acaactagta ctactatttc taacaccacc actcctttcc ctacaaattc

53941 tactaccgct aatactagta ctgactttac taccacagcc acttctttcc caacaggtac

54001 tcccacagtt acaactagta ctactatttc taacaccacg actcctttcc ctacaaattc

54061 tactaccgct aacactagta ctgactttcc tggtataacc acttctttcc caacaggtac

54121 tcccacagtc acaactagta ctactatttc taacaccacc actcctttcc ctacaaattc

54181 tactaccgct aacactagta ctgactttac taccacagcc acttctttcc cagtagatac

54241 tcccacagtt acaactagta ctactatttc taacaccacc actcctttcc ctacaaattc

54301 tactaccgct aacactagta ctgactttac taccacagcc acttctttcc cagtagatac

54361 tcccacagtt acaactagta ctactatttc taacaccacc actcctttcc ctacaaattc

54421 tactaccgct aacactagta ctgactttcc tggtataacc acttctttcc caacaggtac

54481 tcccacagtt acaactagta ctactatttc taacaccacc actcctttcc ctacaaattc

54541 tactaccgct aacactagta ctgactttcc tggtataacc acttctttcc caacaggtac

54601 tcccacagtc acaactagta ctactatttc taacaccacc actcctttcc ctacaaattc

54661 tactactgct aacactagta ctgactttcc tggtataacc acttctttcc caacaggtac

54721 tcccacagtc acaactagta ctactatttc taacaccacg actcctttcc ctacaaattc

54781 tactaccgct aacactagta ctgactttcc tggtataacc acttctttcc caacaggtac

54841 tcccacagtt acaactagta ctactatttc taacaccacg actcctttcc ctacaaattc

54901 tactaccgct aacactagta ctgactttcc tggtataacc acttctttcc caacaggtac

54961 tcccacagtt acaactagta ctactatttc taacaccacg actcctttcc ctacaaattc

55021 tactactgct tatactagta ctgactttac taccacagcc acttctttcc cagtagatac

55081 tcccacagtt acaactagta ctactatttc taacaccacc actcctttcc ctacaaattc

55141 tactaccact aacactagta ctgactttac taccacagcc acttctttct caacaggtac

55201 tcccacagtt acaactagta ctactatttc taacaccaca actcctttcc ctacaaattc

55261 tactaccgct aacactagta ctgactttcc tggtataacc acttctttcc caacaggtac

55321 tcccacagtt acaactagta ctactatttc taacaccacg actcctttcc ctacaaattc

55381 tactaccgct aacactagta ctgactttcc tggtataacc acttctttcc caacaggtac

55441 tcccacagtt acaactagta ctactatttc taacaccacg actccttttc ctacaaattc

55501 tactaccgct aacactagta ctgactttcc tggtataacc acttctttcc caacaggtac

55561 tcccacagtt acaactagta ctactatttc taacaccacg actcctttcc ctacaaattc

55621 tactactgct aatactagta ctgactttac taccacagcc acttctttcc caacaggtac

55681 tcccacagtt acaactagta ctactatttc taacaccacg actcctttcc ctacaaattc

55741 tactactgct aatactagta ctgactttac taccacagcc acttctttcc caacaggtac

55801 ccccacagtt acaactagta ctactatttc taacaccacg actcctttcc ctacaaattc

55861 tactaccact aacactagta ctgactttcc tggtataacc acttctttcc caacaggtac

55921 tcccacagtc acaactagta ctactatttc taacaccacg actcctttcc ctacaaattc

55981 tactaccgct aacactggta ctgactttcc tggtataacc acttctttcc caacaggtac

56041 tcccacagtc acaactagta ctactatttc taacaccacg actcctttcc ctacaaattc

56101 tactactgcc agcactagta ctgcccttcc tagaacaact actttttctc caagttctac

56161 tgaagtgaca aggagcacta ctgtccctaa cagcactaca ctttctcctg caaatactac

56221 tgccctcagc ccttccgtta catctcctat caccaccact cttttgccaa caagcactac

56281 tactactacc gttggtgcta ctgctattgt caccgctctt tccccaacaa gcactgatgc

56341 tactagcact aataacactg ttctagttac aactattcct tctcttacga acactggtac

56401 tgataccact agcatgcatt cggttactac aaatatttta aatggtacca ccactgcagg

56461 aagcacagaa acaactacag gcatcagcac agttactttg gtttcaaacg ctacttctga

56521 acaagctacc gctcagatgg tgcccacagc actccctaca ggcactgctg gtacccacac

56581 cacagacagt gtgtctccag tggcaccgac cggtgtcagt accaccactt atcctccagg

56641 taccactacc cgtgcttcaa gcaccacgtc catgggcacc cctaatgcaa atgttaccag

56701 tgtagtaact ggtgttagtg acattacagg aactcccagt agagacagcc cagctactgt

56761 tgatgtcaac accacgacga ttcccgtcat gaacactttt tcaacttctg cagacgcagc

56821 agttactggt gccggagact tggccactcc ctcacacgta aacacgacag gcattgaatc

56881 tcccactgcg gcaactgaaa atgccaccac agcagatgct gccactatcc ctacacacat

56941 tttcagcact aaaacgacaa attcaacgac aacttctgtc acagtcctta caaacacatc

57001 cattccgagt accacagcta ttcccaccac caaaactgtg agcaggacag agcacacagc

57061 aagtactgct gatgcaggta gctccaggag tgttgcaggt gacactgcac atgtttccat

57121 tccaaatgcc acgatagcct cagacacttt aacctctgcc cccctaggtt cagtgactga

57181 ccatcctgtg accgtaaatt ctacaactag gttttcacct gtacttatga ctaatgctac

57241 cacagacatt acacgtgcta ccatatatac ttcagatact accactcctg acagaacagc

57301 cgtgagtgct gccactgcat caacttacgt tgcaaatacc acaaatactg ctctagttcc

57361 ctgaatacta ttcaaggcag gataactacc ccagacatct cttgaaaaac aactattaca

57421 gacataggaa gtcactgaac tgatacgtga ttttatgcta catgcatttt cacaaatatt

57481 agcactagct ttcatagcta tggctgatgc aagcactgta gacattgagg acagatctca

57541 agatgtgccc agtactaact ttgtgggtac ttcttttgga accaaaatat atactactat

57601 tttgtaaatt agaattatac ctctgatagt gccatcagtt aaactacagg tattttattc

57661 atcttgctac agacttcaca tatgatttta atatcttgga taatactgta aatactattg

57721 ctctgtaaat actattgctt cagatgttgt cattgaatta acccgacttg ttccatagta

57781 ccatacctca gtgatggcat cacaggtgat aatgccacac ctgagagcct ggtgatgcct

57841 gccactcaaa tgactgctat agacgagcta ttactattac agtacattat ttaattagaa

57901 tgctaaacat tgcactttag gttcctaaga gcttttccaa ataccatgcc taccaatgta

57961 gtaatttgct attatacata ctgaaagtat tattgcctct aatgggatcc tatccatagt

58021 tatcaatatg aaaaacacta ccattgtaga tcttctatat actttgatgc caggaaatga

58081 aatcattaag tgaggactat tacaggttac ttcactgtaa aaggaagtaa aaatatgatg

58141 atagacgtta aaaacataac ttcccaaact tggcaaccat tcctgccata ggtactactg

58201 gtactcttac tacagatttg ataagcactt atatttatag acagtaaaca tacctccagg

58261 aaatcacaac tgatcttact taggagataa ttttagatct ctgctgttgc cactagtggc

58321 tgggctcact tgcaaggatc tgatctttca gagaccagaa atcaccccgg cagaggtgca

58381 cacgtaaagc cacagcgtca gggaggcact ttctgccctg gaatcggagt tcatatcctt

58441 ctctgttttt tcttgtatcc acaagctttc cctttcttat tttaccctcc ttctgggggt

58501 taggacttgc agcatgttct tgctctttca tacccaaact cactctttat ctttgatcta

58561 ttactttctt atatcaccca aatgcccttg catagactca tggttgaggg ctattgaagg

58621 cttaactcca gagcagtatt ttcaaaacgt agtttgggga cgcatgggca gcttcagaat

58681 tcttccttgg gtgtacaagc tccaatccat tttcatcaca ttatgttgtt gtttgcctac

58741 ttcactctca ttctcccgtt agggtacagt ggcactttcc agagactcta tgctgtgtga

58801 tgtcaccata gatgcatacg gagacagatg tgacaatcaa gccatcttct gttaaaccag

58861 acactaaaca gttttatagc atagaaaatc atgtcattct tttcactcat tttgtttttc

58921 ataaaaatgc attatttatg ttaacatgga gttgctatat tgttattttt aatgaattaa

58981 aaaatatttt acagaatgtt gtacaaatga ctcaatttta atatctcatg cagtgaatgt

59041 gagcagatcc aacccaccta cacgaagcat agggccctca ctaaatctgg atgtaaaggg

59101 gtcctgagat cagagggttt ggggaccgct tccctggggc aacaacatcc tagtttcact

59161 ctcggggcct ggtcactcat gctgtgagct ttcccggcac gtaggagcca cgtaggagct

59221 ccagcccctg tggcttaagg ttcttactgc acccaaatgt ctaccaccta ctctgaaccc

59281 atttgcacaa ctttctgacc tacttccacg ctgttcggtt attgtttggg tggcttgatg

59341 tcactccaca tgagggggac agatcattgg ttagctgcat ttgccttttg gaatccaagc

59401 agaagcaacc gccctctgaa ccaagttgat agaccttacg ccagggtaca cgtgcctctt

59461 aacctgccct tccgactcag agcacaacag aacccctaat tctcaactgt gccagggcag

59521 ttggtcatag agtccctcac gtggttctta actgagggag tccccagtgc actcatcttg

59581 ttggccctac aagccctgac aggggacaag gggctctggg tcctggggca cactggtcct

59641 gcagccagcc ccacaccagc acagactggc tggccaaaga cttcacctaa aacatggagt

59701 ctggaaactg gggatgaatt tgtccgaaca accacagctc tgcagttact gaaattcagt

59761 cctttgagtg cagaattcag cggtgactac agcatgggac tttgaacact aagtactcct

59821 gaaaatgaaa cagaacaaac aaacaaaaaa aagaaaacct ggacttaact tcagatggtt

59881 gaggacactt gagatttgga aacttgaagt ctacgcctga gaggcggccc tggaaacagg

59941 cagaaaacct ggagagggag gaggtcgcag caaacatcag cgcgctgctc tgactccagg

60001 atctgccagc gctggggcct cccccagatc ccaccacaca cgggggactc tacagggcca

60061 gttctgagag catcacctgc aaccaggtgg gttcagggac tactgcttcc gggtgacact

60121 cctttccttc cctttcctgt ctcagtgtgc ccgcccataa ggtggcataa acagcgaagc

60181 tgcagtgttc acactctccg tccgtaaaag ccgatgcacg gttaatcacg gctcttccac

60241 tattccctga gtgttccaat gtctgtgtag actttccctt cttctccccc tgcctttcct

60301 gatagcacag cacagtctcc ctagtgctgg tgctttataa ggaggctgct ccgagtctct

60361 cgtttttcaa actgaagttc tgtaaaagaa gattcttctc caggagtgtt tctggtgtgt

60421 ctcaaactgg gcttcggggg aaaggctgtt ttctgttcgc tcgcttgatg ctgaagcaga

60481 atgtttcact gctaagttaa ataacgccca tctttcctca ctttacaaaa ttatttggtc

60541 agtggaatct tgttattttt cttaggcttt ttatacgtgc tgctattcat atgttgcctt

60601 ctagagagct ctagctgagg acacaatgtg ccaacaaata acagagaaga aaaagtttga

60661 aatagccaat cacgttcaca tgggcagtgt gcacgtgtga ttgtgtgcat tgttttatag

60721 ggcaaacata tttcctggca gtacaattgt ctttggtttg tgtgatgcaa ttccacttat

60781 ccgtgtttca ttggtttttc caagagcact gttatctgat gatgagtcac tgaataaacg

60841 tgagtgttat tgcttcgtgg gggcttggct tagccctgcc ctagagtgag acgttgagtt

60901 tcacggtacg tgcccagcct ctgctctagg tgtggacagg gtgctagctc ttcctcagtc

60961 cacctgccca tggcgaaggg gtgtgaggcg agatggagct ggttctgtac cgggagcagg

61021 agcaacagat gtttcagtga cactcagtga tggagaaacc tcagagacgt tgacctcaac

61081 tcaggcaaaa tggctcattt cagcacaagg tgcctaaaga tctttgggaa accgcccacg

61141 ggcagaagtt tcatcatagg ataatgacaa gagtaatacg aggtctacta atttcagatt

61201 agactggagg ttctgttttt ctccctggca agctatcatg tatcatccca acaatcccca

61261 tgaccttcat aaatatgtct gtttctaatt ccagccggag aaaaaaatgc gccttgtatt

61321 taattattta aaaagatgat tcatgagagc attgctcacg tggaggacag actgtgttgt

61381 ggatgcgaca caagtgtatc ccccggcgct cgcccacacg agagcacagc gcccgtggga

61441 cagacgttag cgcccacacg gccggacatc tccaacccct gaaaggcttg ctctccacct

61501 gattgtccta gccctgggca acacccaccc actgcctcag aagtctgccc gttggattgc

61561 tgggcagcgt taatgtttct gatatggaga gaacgtttgg cttctcagaa gtccattcaa

61621 aagttgattt tttttttatc ttaagccatc tctctgctag cctttcatct cttcaatgtg

61681 gctgctgttt tcctacgaat cattcagatc aacgctccgt ctttctcaaa cacccctctc

61741 gtgacctctg gttcaaaaaa ctggcctgtt gacttctatc aaatgtagcc atttcgctaa

61801 catagccatt taaataattg tctacaaaac ccggtgattc gcagctctga gaaatgcatt

61861 atgccgctca cttgcggtgc ctgacagctg aggtgaatcc tgcagcttgc agactggggt

61921 tgtccactca gagggccaaa aactcttgct tgggaaaacg gccccttttc tgaaaggaaa

61981 gcctccattt tccttgcatg atgaccagag atgcccaaaa gggggcacag accctctggt

62041 ttcaggggag cctcgttttc cccacaaccg cgaactgtga gaagccttga tttgaagaca

62101 gcaagcccct ctgctgagtg gccccaggcg ctgcgagtgc ccagcagaga gaggaggagc

62161 taaatgtgag caccaaaagg ctgtgggatc tcggaaggga gcccccagaa tcctccagaa

62221 ggaatgacag ggaaggtaaa gggctggctg cctcccatgt gcctcgccat tcccagagga

62281 cgggtgttgc tctgccagga gtcaaggtcc gcctcagcac ctgggcatag ccagagtaat

62341 gatccctccg cggacacaaa caatgagcca ggcatttcat ggagcggcgc agtgacatta

62401 aactccgaag gacagggtgg cacagagctt ttcaaactta gaagacagaa tgcccccaga

62461 tgctctcaga gcagccagcc ccaccgccag cctgtcaacg gtggttgcaa cctgccggct

62521 ttgtccgtgt ggaccccctt gatgtggctg gcgccccaga gctaaggact tcccctctac

62581 cctgcccttg acccgttctc agagcccaat tctgctggcc tggacccgga aggctctcca

62641 cacctgctga cttgagtgtt gagaatgtga catttgatgg gataaataaa gatcatccag

62701 tggtagaggc atgggggggg gggggcgggg acagcaagga ccgcactgta tgtggcattt

62761 gagggtgcct tttaccaagc cattcaagag acttcccagc tgcttgtccg ggaccaaagg

62821 cttaaaggaa agagaaagcg ccaggaagca agtttagtcc tatttaatgc gatgtctgaa

62881 gctttgtccc ttataaacac agcctgtctc tcgaccacat atcacctgtg tggcttgccg

62941 tctctgcctc atggggaggg gcagacagaa tgtcagaaga aggcagaagc aggggccccc

63001 tcgggggcca gcccgcacgg agaaggaggg gtcccagtcc tctccatctg cattcgagcc

63061 ccctcctccc cacgtggcct cccgctgagt gagccggagc aggagacttc ccaggagtgt

63121 cccaccgcct cacacctacc cctggatccc gattttgtca gaacatctgt ggccccacca

63181 gggtttctga agctttcaag gatgaattgc attgatttac agagtaccgc agtgcctggg

63241 cctgcagcga tgcgccctgg gccacgggaa gggacacaga tgggggcttt ggggaagcaa

63301 agcacaggga agtttctgtg tttgatggcc aggggagggc tgagcagaca gagggctgag

63361 caggcttcag ggctggacgg gagagacact gtcagactcc agcctgcagg gtggcgagga

63421 gccaagagaa ggccccctgc ccaaacaaga gggcagccag ggggatgtgg ctcagggtgg

63481 ccgttcctga ggctgagagg ggcatggggg cagcagccct gaagcccgca ggggtctcgt

63541 cctggggctc cactttgccc ccccatgact ccaagcaggg cccccgaggt gtggggatga

63601 tctctggaat ccagcctgtc atgttttcca ctatctcctg cgggagaagg gaaaaagcag

63661 gttgaggtag aagacccccc cacctcgttt cttttcctcg gacctcactt ggtcatcccc

63721 tgtccgcaca gcctcccagg ctaaccggga gactccggtc ccacccccaa cctggagctg

63781 cctcagccca actgcctgct cccccagcct cgccacccca gccccgtgtt cctaccagcc

63841 tgcacctccc tcactcttct ccattcctga cttctcccaa tgccatcccg tatccctgcc

63901 tccctgaccc gaggtggcag agtctcctgg ggacactcac gtcaatggtc tttattatca

63961 ccgtctgctg cacctccttg caggctttct cagccacccg gatgctctcc gggctccact

64021 ccacattgtt catggccatc gcgccctcca tggaactgcg tgggtggggg cgggggaagg

64081 ggggagaaca gtggcttaag acgcagagct ctgacaacat gtgtgtgaaa tcccttcctg

64141 tccgtcagcg tcagtttcct gtctgcgtac actcgaagga aggccttacg accgcgtgag

64201 accgtcagga gtttggcgaa ggagacgtat ggtgggaact cagagctgag aacttttacg

64261 agggaggaag ggacacttct gatgattcaa agaagtggct tctcatctgc gggccctgaa

64321 gttcagggag gttggtggag ggacttcagg gaacctgtaa atgcccgcag gtcgtagatg

64381 aaacggggcg cggatgcaca gacgtgcctc tttctggcaa aggtgtttca gagctcctgt

64441 ggcgcctcag ggaggaccct tgcccccgaa tactagagag tcaccaccag aaaggaaaca

64501 cagtctgact gagtcaggtc gcgcatgcga aaaaggtggg ccgagaatct gtccacaagg

64561 gatatctcac tttgggaggt cacgctcgag ggggctcatc atcctggggg gactcactct

64621 gatacctcct cccccagctc gtgggccacg gagatgatgt cagggtaccc ctggcagctg

64681 gagatgttgt tccgggggta gtagaagagg aagatgaggc acatctcatt gacggtgctg

64741 ggaccgccct ggaggagaac agggggcaga tggagagaca gagatacaag tgcctgcagg

64801 gggaacagga cggggggggg gggggcgggg gggcagggga gaggccatcc accaatgacg

64861 gtgctttcct ttccccctga tctgcagcca gctcactgat tcctacgtac ccctgctcac

64921 cacacctcct ccttcctcct cctctatttc tggccggtgg acagcgatgg atattatttc

64981 caaaaataat cccacatacc tgcccccacc ctctgttttc ccactgcccc tgtcttggtt

65041 tgggaggata agatacaata ttgaattttt tgaaaaaagc cactccctgg ggaactcctc

65101 cagaatgccc catcggtgca cagctaagtg ggacccaccc caaagggtca caggttctta

65161 tctttacggt tgacccccgt gaattcccct agggcattct ctccagcctc cctcctccta

65221 acacaacctc actgccgacc ttgtatttga gccctctgaa cgggggctgc cttttcttcc

65281 tcagtgacaa tggcaagcaa tgccttttgt ctccgagaag cctggcacca tccctgacgc

65341 acaatagcta actctagcta actctagctg agcatccctg caagccagac aacggcctag

65401 atgttttacc cgcatcaatc ggttctcaca acactcctcc gcggtagaca gtattgctga

65461 ttcttttatg aactaggaga ctgagactcg aagaggctgg gtaacttgtc taaggtcaga

65521 gtgatagggg acttaagact tggaaaattt tagctgaagg taaatagcca cacatctagc

65581 aagtaatgtg tatgttaagc tttttgtttc agaaactaca gctaaggccc atctggcgcc

65641 tgaggaaaaa aaaaacggct gacctcctgg ggtgctgtct ggagagaccg catgaagaca

65701 tccttaccag gtgactaccc ctccccttgg aatttcaagg cttttgccaa cctgtttgct

65761 ctcccccacc cctcttataa aagatctggc caggaaagaa agaagaagat ggtttgttag

65821 ggtatgagcc cgccatcttc tctgatcgcc gaccatctga ataaagcacc tataaaagat

65881 tcaatcgctg tcattactta ttggatttgg tagtggcagg cagccgaatg ccggtgtctt

65941 ttccggttac aagagagcca ctcatgccgg tgttggcatt cagacccagg ccccaaggcc

66001 ccgagtccat gcactgagtt gtgataatgt actgtctttc cgggaatcct tgcacccatc

66061 ttgcaaccac tggggtgtga gaacctgaaa acccccctgt gactgggcga caccactaga

66121 gaagggaggg ggattcagct ggaacaatca gctgcttgat agccaaggtc agggatgggg

66181 ctgcgctctg aagctgttgt catggtgact gtggcgggca gggagctact tacaaaagtc

66241 agggagtctc ggtccagcgt ctcgtactga cactccacca gcaattcatc tccctgtcga

66301 gagtgaaaca caaaggctgc aggcaaggcc aggggggtct ggtgcagtct tagagctctc

66361 ctggctgaca gcaatgagcc ccgagactgc ctgtccctcc agctccactg tgcctcccca

66421 gggcgcccac cggcttgatc tccacccgat gaggcaaatc tcgagtctcc tgcaggctga

66481 agtcgtagga atcgtcttta cagattgttt ggagttgtgc tctatttcta gagggagagg

66541 aagggagggg acaggaatga gaagaggagg gtgaggtggg agggtgtgta tctacgaaga

66601 agccttgtgt ggggtcatcc tgtgggtaca gagtgtccct aaagcaccat ggctcctcct

66661 cccaccgtct tcctccaggc gtctctgctc tgtggacggt tctggctcct ccttgcgccc

66721 agccttctgc tgcagaataa atgactgttg ctaagctcta tttcccacgt gcacaaggga

66781 aaccttcaga tacgtgtctc cttcccttac ctgtactgca cggccttcag agcccgccca

66841 gccagatggg tgtggagcag gtagccgaac acctgtaagt caggcaccgg ggccccgttc

66901 atctgtgaca gggaggaggg gcgtctggtt tggtaagaat tctcatcttg ccctgcccct

66961 tgattttccg cccttagtac ctgacacctt cctctcagga ctgacatgaa ccccttcagt

67021 tttaaccagg ccctcctggc cgcagctcct gcccccctgt ccccacagga tggactgact

67081 cctgcatcct gtgcatgatc caggccagtg aggttcctga gctccgggct gtggtcctcg

67141 tcggagggtg gggccgtgca ggccgcgtag gctgtggatc aggctggatc tgtggtgggg

67201 agccagaacc ctcagggccc gcgcaccttc cagcctcacc tcttggaact tctccgtctt

67261 acacagcccg taggaccgga aggactcggc gcccgggggg atgaagtgga tggggaacgt

67321 gaggaagccc agctgcagca cgcccatgtc gtgtttgcgc agctgggccg agtagtacat

67381 ccgaatgccc gaggtgtcgt acaaacctgg tgcacaaagg cgcgatcaca ggaggcagga

67441 gagggggggt cccaacgtgg ggggaggggc aggtgcaaaa aagctggggt gggatgtgag

67501 tgacaggcgt ccgcttgcct cccacaaccc cttcccagct cccaaaggtg tccggcctct

67561 gtagctcttc ctccgtcaac actctagatg ctcaccagga aggttgtgaa aattgctgta

67621 atgaatctcc aggcggaccc actgggggtc tgagggcatc ccgatggaga tgcccacgtc

67681 atctggaaac tggtaactct gttgcgtgga gggagagggg cagcgagatg gctgggagct

67741 ggtgcaggga cccatcccag atctcccttg cctcccagcc tcccaccagc ctggccaacc

67801 cagctcccac ggcacacgcc gcttagccgc ctcgctcctc aagccaggac tcactgtgcc

67861 cccgacagcc cagcccacga tgacctggga gcagagggag aaggcggggt cggccccgta

67921 gcagtcgctg atgcccgtgg gcagaatgct ggcgttgccg caggcgtaca agaggatgtg

67981 gtgcaccaag gtctcgttgt ggtccagcag ccggggctcg aactgggtgg gcggaggagg

68041 gcggggcaca gtgaagatcc tccgtgtctc gtcctagtcc ccaccttccc gttccccaaa

68101 taaaactaca cctgtcccct gcatctctgt ttctccttcc cctccatttc ttctcttttt

68161 cttcccacgt ggggaagctc aggtctacac ggcctgagcc ccacactccg tcgtcgccct

68221 gtctgacgcc tctctcagca gccgtccctg agagcgtggc aggctaaatc ccgggacgcc

68281 agcctttccc cgcataaccc ctccgcgggt ccgtgctgtc tgggcgcgca agcgtgcctc

68341 attggactcg ctctctagag aaaatatggc tcccgcacgt ttgccacgta ttccaagatc

68401 ccatttttat ttatacgatc aaaactggaa tcgcccaaat gtgactttag gtgcccaagt

68461 ttgcgttccc tctggccaga tgtcccttgg ccacgcccct cccagccaac acctcctcct

68521 gggctgacgg cctgattctg gggccagggt ggggcttgca ctgggtggtt tgcaaaaact

68581 aattaactcc taatgaactc atgaccttca tcaacagctc tggctgcagc gtggggctcg

68641 aatgaaactg cgctgggagc tgaagctccg ggctcaggaa cggctgacac gcccacggcc

68701 cccccacctg gctggcccca ggttggcccc atgcttcctc ccagcagtcc gccgactgac

68761 accatcccct ctgtgcctgc tgtgcccact gagctgcctt gaccgggtcc ctgccagcct

68821 cgccggtacc ctgtagatgt ggtgtttctc gctgacgatg gggagaggca ggaaggtgca

68881 ggcgtaggtg gtgtcgtcct cagggatgag gaactggaga gagagacaca cagaggctgg

68941 gctgggctgg gctggggtcg tggtgggggg gtagctcctg gagaagcaag gaaagggcca

69001 ggccagggag gggtgctccc ttcgaggccc atttggctgt cctccctgga ctgagggaag

69061 tcagaggaac ctcagttcta ggaacgtgct gagtcctggg gacaagggca gggtctggcc

69121 agggccggat gccgggaagg gggctggagc aggctgggtg gggtgggggc ggggtctcac

69181 gtcggtgatc tccaagtcgt ggatgatggt gttctcagga acgtccaggt cgtctgggtg

69241 aaatatttgt agcaggaaga tggacttgac gaacatgcgc tcccgatcca gctgcagcgt

69301 gtcgtctggg ccgtaggcag ccagcaccct catggtgtca ctctgggggg gtcgcaggca

69361 tggaggtgct gcgcccacga gccgcacgcg gcacccaccc acacccaggg gctgacacga

69421 gccacctgct cacgtgtaca cggtggggtg gcaagggctt ccatggggac ggggcagaac

69481 tagtcagggc cccgttctgc ccccggggcc aacttgccgg gaacattgag cgagtcacac

69541 cccctttctc attccctctc taggcctgtg tccacatctg ggaagcgaca ccccaaagtt

69601 cagtgaggtc gggcagggtt cttcaggagg ggaaggctgg ggtgggtggg gtggccactg

69661 agtccgtgag aagccacccc gccgagggct tgtggactct aagaggggac agttggggcg

69721 ggagggacgg aagtcctggg gactgtgact ggccacctgg gatgcatggg cactccagcg

69781 tgcaggtgac cacgaccccc cgcccccgaa gagtatgctg ctcgtgagcc cgtgttttcc

69841 tctctcccga atgtaccgtg atgtcttggt cgtgagggtc acaggagcgg aagggcctgg

69901 agaagcgcat ggtggtgtag acggcatctt ccgtgagccc cagcagctcg gcatcctggc

69961 tgccgtcctt ctccagggtg tgttcatctg ccaggtgctg atcctggggt ccgggggagg

70021 tcaaggagta aacaccagga gccccaagtg cccagggctt ggaagcagcc tgtgtgccct

70081 tagctccccc aaggtaccgg cttaacagaa cccagggctc ccttattagg gagtttgctc

70141 ctgggacaga ccacggtgtc cagggtggag cctttgcgca gaggaacgga cagcgtctgg

70201 cgggagtttc gccacctgac cttgcttgac ttccgttcca agtcccatcc cccgtcagtc

70261 ctgatattcc tgtgtcccta accctggaaa gctcatggat tctctgaact ctctgtgcta

70321 ttcatcctgt ccagtggctc ctaacgttgg cgcctcccta taaaggagat tgggagatgg

70381 gtgagcttcc tcatctgaaa aatggagtag cattcaccca cctcatagct gatgctgtga

70441 ggtctaatgt gcagaggatt tggagcaatg gctggttaat caaaagtgca taacattgtt

70501 aagctgttac ggcctcgggg gctgtggtta ttagactatt tcacgctgtg gtctacctga

70561 cagagcttac gggaaatgga gggtgaagac tgtatctcag cagctgcgcc tccccctccc

70621 tgccccccgt ggcccacatt taggggctat tagcaggcat gtgggtcagg gctctctccg

70681 tccagtgggt ctgtccctga ccaccgaggg atgcgcagtc tggaaggtgt agactgagtc

70741 cctccagtgg accccttgtg ctgggagagg cagccgccag ctggtgttct gggcatgtgc

70801 cggcctgtgt ttggaacatg tggctccttc ctggccttgg agccatcccg aacccagcaa

70861 gggaccctca ccctggggag agagtcgccc tgggacttac cgagaaatag acattgccct

70921 ccggcaagac gcctccgaca accagatcgc tgcccaccat agtgtagcga ttcgtgatgc

70981 ccaagcccac ccagccagct gtccggacct ggagctcaaa ggtgattatc tcagcctcaa

71041 ggtcaaagtc ccagcgcagg aaaacggcgt tggaaggatc taggaacctg gaataacgca

71101 agcgcgaggc tgggccctga cggctgccct gggagggggc tgcgaggacc gtaagcagga

71161 ggagcccgaa ggggagggtg caggccatgg ctcctggggt cgggggcacc tctcctgtgg

71221 tggacactca agaggtgtgg acttatatgt gcttctcccg gtgccgggca ccgagccatc

71281 ggggagggac agactggggc tcttctgatc cgatcgtcat cccctgggtt cagagtggtg

71341 ccacttacat aacccagagg tgtgaggtgt ccagcagctt gtggggcagc ttgctattat

71401 tgccctggac gggggacctg gcatcagatt ctcaggagca aatcttttaa actcgtctcc

71461 cccaacctca tgctggggtc cagccaacag ggacctcaca gagtcaagga ggtctctcct

71521 ctcctctctt ctcttctcct ccccctccca ctttctctcc ctccctccct tccttccccc

71581 ctcccctgga tccttggatg tccttactga gcactctggc ctctggcagg tgacccccac

71641 cccggttttt atggacagtt cctgttttgt ctctatacag aactggagca cgcagagggg

71701 tgccataggg gtgcatggga gcctgccttt ccgcctccca gcccagcagg aagggcagac

71761 cttgctggga ggggtcaagg gaaggttgcg atttccatct ccctgaggag tctttgctgc

71821 taatcccgga aaggacacac acagctgaag gagccctaat cagctttcag gtgggcgagc

71881 cggggtagct ttgcgtttct gcacattctt gggaggagca agggggaggg ggcatgaggg

71941 gaggacgagg gcaagatggg aataaacctg aacttgcatt tgagaggctt tgtgctttat

72001 ccttcccccc acgaattgcc cttcagagga gtcacctcca gggcctcccg ctggaggctt

72061 ggcccggtgt ccccacgggc cagcactttg gtgtccggca gataatagaa gggcagaaag

72121 aacgtcagaa tatgagaaca tacgagtaaa aaggagtatg agttccactt gattttagaa

72181 acattgcaat ctacctaatt ttcagatgag gaagcagaag caccaagggt gacatgaggg

72241 ggtgtctgtg tgcaggcaga gctcgcacag gacccagggc cctgccccca ggccagtgct

72301 ctttccaccc gtcccttagg acgccccttc ccccaggccc cagcccctgc cgtgccttcc

72361 caccagggct tatcctgggg ggtactggtg gcatctggtg tcatggctga ctgcttctta

72421 gcgagggcac cgaagtctca ccctgacccc aaagctcttt cctgccggag tcggacatag

72481 ctcctctgta gcaggaagtc acctcgctga cccctggggt ccgtcctcca ggagtcagca

72541 gagcggcaca gaaaagttaa caaggacacg tgtccggaga gcctctcccc gcacagacac

72601 tggaccaccc cccacccccc acaggaattc agctctaccg ggatggacag gacctggagc

72661 acctgcccgt gcctccattc agcacctgtg tgttcctctt actgcctact gcgtgagtcc

72721 ttcaaagccg ctaaggaccc aggtgaggcg gtttgcagcg gctctgagcc cctgagtagt

72781 cactgggctg tggtggaaga gaaaacacat ttcctagggg gacagccata atgcccagct

72841 cggggcatgg gaggaagtag ggagctaggc agccacctgg aaggaagagg cttaccttgg

72901 cccaggacgg gagccaggcc aagaccagac acagagcctg gggaggcgat gaccttgagg

72961 agcagggatt cagggaggag ataacaggag tatcagagac actctgatcc caatcacatt

73021 tgggattaag gcaaagggag gggtggcagg gagaccggac tgccagccct gtaaactgag

73081 acacactcag gattccttgg ctcacaatct gtctgtgggc ctcagcactg gcctcaggag

73141 gaggcagagt cacggggatg ggctggagtc acagccactg caactcaggg gctttgctcc

73201 aggccccggc aggctgctgc ccacggcctc actctccctg tccggtgtcc ccacccccct

73261 ttcctttcac tgtctgatcc ccagagtgac tctccccaca gccaaacgca gggtgaaatg

73321 catttttctg gtgttcagga cgtgtggttt gggcgatgcc acccagagct acagccctct

73381 ccctctccct ctccgggtga tgaggttggg aacaggtcag gaaacaaaat gcgatagagt

73441 gaatcagcac gttactctca gaaggtagca cagtggacct tctgtgcaca aatagatgat

73501 cgtgcaattt tgaagtaggg tgacacaaaa aatatcagtg aagtcacttc atacacaaag

73561 ctgtacagaa tgaaaaatct gtcttttgtt taaatcaaaa agcacacaat tccaacgtca

73621 gcaagccaag cgaatttata aactgtatta tgttgccacc cagtggatcc tttgagaatg

73681 ggaaccctat tgctgtgtgt attaaaattt cggaagaata aaaatataaa cagtgactct

73741 gcgaggtgta aacccccagg acgccaggcc actgctgggc cctgacgtgg ggactctcca

73801 gggatgtggc tctgcggtct ttccttgttc caattccctt tcatttatta tctgcggtga

73861 acagacagac acccacttgc aacactgact tgaacatctt gaaacagtga agttccgtgg

73921 ctgacccctg cgtatctccg tactgtcacc tgtaaaatgc agaggttgga gaggtgggag

73981 atcaccggtt tctgaacctt ctgaaaaatg aggcctctta atgttaaaaa agttagaggg

74041 acttgtgatt ctaatctctg ttgattatta ctaagtgata gacgattaga gagagataaa

74101 tgctactatt aatttgatca cattttagtg gaatttgaaa cttgccattg aacaattgaa

74161 gtccagttta caaagtgctt attctgtgcc aagtgttact ctaagaattt ttcatgtgtt

74221 tttaatttat tcctatgagg tcactttctt tcttcttttt ttttaaagaa gtcttttgcc

74281 tattttttga aatattttat ttatttttag agagagggga agggagggag aaagagaggg

74341 aaagaaacat caatgtgcgg ttgcttctca catggcccca ctggggacct gacctgcaat

74401 tcaggcatgt gtcctgactg ggaattgaac tggcgaccct ttggttcaca gcccgtgctc

74461 aatccactga gccacaccag ccaggtatga agttgctttc attattatct gcagttcacg

74521 aattgagacg ctgaggccca aggtcacaca gcacaatgag tcactgggaa aatacaatgg

74581 aaaacaaaag ctcctaactc agaagatctt gtcccaaagg cagaaaagaa aatccttcca

74641 ttattaaatc agtatgaagc cagaaggtga cacacgcccc tggcatttct ttaagagatt

74701 gctaagacca agaaacctca gcctttcaca caggcctgga gacccagccc attacaggca

74761 tgttttcgag ataagcaagg tctggtcctc aaacgagagg gtctgacagg accctctgtt

74821 aactcatagc tcaccccagg gtcagtgggt aactggggtg ttcctctgtg tttgctagtt

74881 ggctttatct gaagggaaaa taaacttggc atgcctttat gccggggtca tttgcgagtt

74941 ggtgtgagcc gtccgctcta aattaagtta ggctcctctt cctagatgtt tatatttcaa

75001 agggatggct gtggggtttt gagaacggta ttcctgggtc ttaaccctgg caacagtttt

75061 ttgtctttaa aaaaactttt taagtcctca cccaaggata tgtttgtttg tttgtttgtt

75121 ttcattgttt ttagaaagaa aggaggtagg ggaaagagag agaggaacat ccgtcagttg

75181 cctcctgtac atctcttgac cggggattga acctgcgaca gaggtatgtg ccctgactga

75241 gaatcaaacc tgaaaccttt tggtgtgtgg gacaatgctc caaccgagcc accaggacag

75301 ggagagctgt gtggctttta aaaagaggta cacacagccc tggctggtgt ggctcagtgg

75361 attgagtgtg gatctgggaa ccaaagggtc actggttcag tttccagtca gggcgcatgc

75421 ctgggttgca gtccaagtcc ccagtagggg gtgtgagaga ggcaaccaca cactgatgtt

75481 tctctttttc tttttctccc tcccttcccc tctataaaaa taagtaaata aaaaatcttt

75541 acaaaaaaaa ttaacataca tttcagagac agagaaagaa cttacaagtt ttctaaagta

75601 aacgctctaa gaaaatggag gggagggaag acccttccct tatttttaac caggagaatt

75661 aagcatctga tgttgaattt gtgcttgccc tttcagagtg gaacgtggga aattggagat

75721 gagcgtccat tgcccatagc ccagtgtggc ctggcagcag cctctgccca taggtgacac

75781 actgggggtg gagggaggtc ttcgtgtaat ctgcaagggt cctttggttg cattgagatg

75841 ttttaatccc tttccaggaa cctctgggac acagctggga gggtgcaggg ctggtctgtg

75901 aaggcactca gtgacctcgg ggagtgtgcc tgtgtatggg gggggggctg ggccagcaac

75961 taagatacgc tctccagttt gaggtttgag tctcagtgag aagctgagac gagacctttc

76021 gtttcctgga aaagccactc ccttttgaga gtcaagccca gggagtgtca gccactaact

76081 gccgcctcaa aggcccaggg cctgctctcc tgcctggctg gccggccctg tctcacggcc

76141 ctgtgggagt ggacctgtgg ggccagggaa tggcagcact tcccattctg tggggctggg

76201 tgctcgtggc tgccgtgagg acaggtcaga gccgggcaga gaaagccact cggtgggtgg

76261 acagaatgaa ggcgggacag ggtccctggg gcaccaggag gcacagaagg ttttgcttcc

76321 tggtcccagc ctggcccggg ccctcctaga cacagcctct tgtcccaaga aagtatcctt

76381 tctgctccca gatacttgct attggcaaat cccatcttca ggatttggct gccctagtta

76441 tttctggggg tgacctccac ccccagtccc tccagcctga cctgcctgtg tatttcaggt

76501 gccacttcaa gttttgtgga gcaaaagccc aagtgggccc tggtgcgtcg cggagcggct

76561 gaaacgctgc aatgctttct gagggatacc cagtacccct ggatgagctg gtaccagcag

76621 gatctccagg ggcaactcca ggtgctggcc actctgcggt attctgggga caaggaggtc

76681 atatcccttc ctggagcgga ttaccgggtc atgcgcgtgg atgacaagga gctgaggctg

76741 cacgtggcca acgtgacaca gggcagaacc ctgtactgca gctgcagcaa agacacagtg

76801 agaaactctc cttagacaaa tgaataaaaa cctccctggg ttcccacccc tgagcctaag

76861 ctcctcctcc tctgcctgcc cctacagcac cggagcctgc ttgtccccgt cccaggtcct

76921 cccttctggc ctcttgactg cacaggactc ggcctgcacg gcacaggggt ggcccacagg

76981 ctctggagga cccacctgca gaccttggct ctgccatctg tgagctgtgg ggacgacgtc

77041 ctctctttga aacttagttt ctgagcctgt acagacagta actctaccca gcgcagcggg

77101 ccctgtgagg attaaacacc ccagttcagg taaagccttt agaataagac acagatacac

77161 aggaagcact tagcttaagg agcattagct cttgtccaca caatctgcac acaagaaaca

77221 agcatattgt attttcatgc caatctcaat gtccagaggg cactggaaag aaacctggag

77281 gcattctggg agctcagaaa gggggagaat ggatgtctca gcctatggtc cccactcaca

77341 cccctggaat aaacacccgc ttttccagtt acatctccat tccccgtgct cactaccgcc

77401 tcagcttctc acacccctgt ctcagactga tcagtactta aaagtaagat cctccgagag

77461 ggtccgactt ggaaaggtcc aggatccgga atccacagtg gagggcttac ccagcacctt

77521 ttaaaacgcc actgctcagg gccggctctg ctcccgcctg ggagtcaggg gaaggcaggg

77581 ctaggccaga ccccgcaaac atctggaacc ggcagccggg aggtgcattg tgggagaggc

77641 ccagctcctg agacctgtgc agagcccgga ggcatggcta caggctacag gaggggagcc

77701 actctggctc cagttcggag cttctccacc ccaaaggagc aggtaactgc cgtgcgaggt

77761 gggcgtgaga gaagaggagg cgttgcaaag gccctgtgcg gtctctaacg gtggggtggg

77821 cggtgcagag gggctgtagg cgctctttcc tctgcgtttg tgtagaaggg ggcgcttttt

77881 ccatttgggg gcgccatctg cccacagttc gccattatgt gcgcagccag agggccacag

77941 gtgttgctct tctctcaact ccccaccccc ccaacatgga tgcctgtgtt ctcaccttcc

78001 tccaggaagg gtacctttat tttagaacac cttccctggg aggcccttgg ccaccaagag

78061 gctgtttaat accgaggagg tggcaggcca gagaggccct gggcaacctg cttttcttga

78121 aaagttgagg ccccctgctg gtagctcttg aaattactac tgccgagcac ctctggctgc

78181 agggatggaa tccaccattg ccctgctggg tcccctttaa cttcggcccc tgagacctcc

78241 caggtgcagt tctgctcgcc taacaccgtc gtccgccctt ggttccacgg tgattagccc

78301 tcccagactg acagatggta agagttcaaa caatgctttt tggtattgct tttaattcat

78361 tgtttggttg tgctgtgtca tccaatctga gaatgtttgc tttttacatt ttttaaaaat

78421 gtttttaatt acagttgacc ttgaattttg tatttcacac acgtactttt tcatcgagtg

78481 aattatgcta tctaacccct cgtactgaga tagataggaa atatctatgt gttggcagtc

78541 tcctgtcttg aggttgataa aagtgctttg cccatgtcat tacagagtgt tcgcaacttt

78601 attttattta ctttttaatt tttttattgt tattccatta cagttgtccc atttcttccc

78661 cctttgccct gcgccgccca gcccaccccc actcccacag tgaatcccca ctccattgtc

78721 catgtccgtg ggtcattcat acgtgttctt tgactagtcc cttcccctcc tttccaccat

78781 tatccccctt tgtactcccc cttcccctct ggtcgctgtc actgtgttcc atgtttccat

78841 gcctctggtc ctgttttgct tgttagttta tttttgttca ctaggttcct cttataggtg

78901 agatcatatg gtatttgtct ttcagcacct ggcttatttc acttagcata atgctctcca

78961 gttccagcca tgctgttgca aagggtagga gctccttctt tcttttgcag tgtagtattc

79021 cattgtgtaa atgtaccata gtttttttat ctactcattt accgatgggc atgtaagcta

79081 tttccggcag ctggctattg taaatagtgc tgctatgaac ataggggtgc ataaggtctt

79141 ttgaattggt gtttcggatt cttaaaagta tacatccagc agtggaattg tgggaaggca

79201 gttccatttt tagttttttg aggcaattcc atactgtttt ctacaggagt tgtaccagtc

79261 ttcattccca ccaacagttg caccagggtt cccttttttc catatccttt ccagcactta

79321 ttgatttatt aatgatagcc attctgattg gtatgaggtg atatctcatt gtggttttaa

79381 tttgaatttc tctgatgact agtgatgttg aacatctttt catatgtcta tgagtcatcc

79441 atatgtcctc catggagaag tgtctattca gttcctttgc ccatttttta attggattgt

79501 ttgtcttcct ggtgttgagt tgtatgagtt ctttatatgt tttggagatc aagcgctgtc

79561 tgatgtgtca ttggcaaata tgttcaccca tacagtcggt tcccttttca ttttgatggt

79621 gctttcttta gctatgcaga agcttttaaa tttgatgtag tcccatttta ttttttcctt

79681 tatttccttt gacctagaag atatatcagc aaaaatattg ctatgtagga tatctaaaat

79741 tttacttcct gtgtttgcct ttaggacttt tatggtgtca caatgtacat ttaagtcttt

79801 tattcatttt gagtttattc tgatgttctc aactttagtt tctcgtttaa tactttcaaa

79861 tacctgggat tcaacagggc caggtgggaa tagacagaat tatcctaatc aaatctaatt

79921 tcttggaggg aaagtgatat attttctgat tgtgtaaatc tgattctgct ccatagctat

79981 cccaaagttt ggattagttt taagatggtg gaattctaga actggcaggg gaatatagcc

80041 acctgagaca actgtctctg attcattttt gtctcagcca tctcagaatt aatcaataag

80101 tgcgtagact atctactcag tttccagtac ttcactagat agggagtgac attcaagaca

80161 tgtaaggcac gtttcttcac cttgacttcc tccaaatttt atccctcttc tttccttcct

80221 tattctttca tttaggaagt cctcagagag cagaaagcat gaacttaaaa tttccctggg

80281 agttctgaga ggcagagaaa ggaagagaaa cacctttatt ataattcaga ttcttgagca

80341 cagagcagag acacagcctg tgtttatggc ctgtaggcca tcattgcagg gcccctgagc

80401 aggagaggaa cagggtctgt gaaggtaagg gctacataca ggtgggcctg gggagagggg

80461 agttgataat gggttaataa ttatcagggc tttcaggatc cactcaagtc tcttgagctt

80521 atacatatgc atagggcccc gatgggtgct tgttaagtat agactgataa ggtttgggga

80581 tcctagcttt ttcagcctta gtaatggtag ttttaggggg tatgccctta ttcaatgctc

80641 tccaagtcct atcctgatgt cgttaggatt tgaggaggct aatgaatgat atccctattt

80701 tctcacataa attctggaca aagtgagaga gataaaggag tgaggcaggt ggagagggaa

80761 attcctagca aagttacttg aagagccgat aaagagtatg gcttagatat gtggcaacat

80821 ctggatttcc aaggctgcaa gttaaagtta taaaagactt aacccaccat ccaggatact

80881 gagtgatgga ttccattgtg ttttcataac caaagagcaa acaaacaaaa cacgacatat

80941 ttgatactag tggactgatg tgagtcagca aaactcagtt agttatattg actgattcaa

81001 tgccccttga atgaagggga gcctgcctgg gcacccgaga gaatggaccc tgttctgaca

81061 tagtcttcct gctagcctat aaaagtgacc tgagtctatt tacttggcga cttgcactct

81121 aagaacaaga acatagctaa ttttttttga gggatcctgg atcataatta gtactaattg

81181 gtgttggtgg tggttgtggg ggagacttat ttgtgatcca ctggtcagga tgaggcttat

81241 atggaaactg atggataaat ggatttttaa cttaagtctc tccaatagta attcaaaaaa

81301 tttagaattt tttcccctat ttcctctgta tactgtagca ggaggtgggg caggaactgt

81361 atgttttttc tacaactggt agaacctcta caactattac aatcgctgcc taacttctga

81421 agtgagggct tttgttggaa gaagggccaa gaggaaattg gtgaagtttc tcctttgtaa

81481 atctaaaaca tgggcacatt gcagtgatcg gaactaccaa cacaggcttg aagtttgctg

81541 gctgataatt tgtattataa tcccattcaa tacttcagca tggccaagcc aaaagattaa

81601 taaggaatga tggtgtttga ttactggaag gaatgatggt gtttgattac tgggtaatca

81661 ggaagtaacg ataatagtca ttatcccata tgtagccctc ttactagagc aaatccaacc

81721 actcacattt ggtatgcaac tattcaccta gaaagtacat cttgtacatc gtctttgaga

81781 agctcggaat taattagctt tcagttgtca ggggcagcag tatattttca cctccatgct

81841 caggcaatca ctgttgctct ggccctgaac ttgacaatct caaagggtac catgctggtc

81901 tgttatattg atgatactgt gttgatcgga ctagataaca ggaagtagca ggtttcctgg

81961 atgtcttgca aagacaggta tgtggtggtg ggtaggtgat acaagcagtg aaaacctgct

82021 actagaatga aacttgtggg tggatgttgg gttcttcccc tgacatgaac acgctgttgt

82081 accctgtgct ccctactatt aggaatgctg cctgaaggcc tctacattgt ataagcaaaa

82141 tctctctcat taggatatca tgctctgagc catttaatga gagatctagg aagttgctaa

82201 tttagtgtga gactatgtca gaaaagatta tccacccagc acaggtgttg aaatccaatt

82261 ggctctgttt cctgggttta aactctacag atttggtagt gctagaagta tcttgagcct

82321 gccaaatgaa gctcatggca aaggctaata ggaagatcag aacaggaatc cctacacagg

82381 gcagcagact catgctttct cctctttggg aaacggcctc tggcttgtta ctgtgttctg

82441 gtggagacta aacgcctgac tctacatcac caggtggcca tggagttgct ccaggtgagg

82501 ctgcttttct gccatcaact tggatggact cagcaatgct ccatcgtcaa caagaagcat

82561 gacgagaagc ataaatggaa cagaagtgaa tgaggccccg aaggtccaaa aaagttgcat

82621 aagcaattcc tctgggccat ctttcttcaa cccgcacctc aagaagacaa cttttccatg

82681 aacagttcac tgaggatgag atgtcacctc atttatgaat ggatctgttt gctctattgc

82741 taccgaccgc atgtgtactc ctgtggtgtt aaaacccctc agagggatgc cctgaaggaa

82801 agaggagaag agaaatcttc tcaaggggca ggattcaatc agcacatcta gctgttctct

82861 ttctggaggt ctcgctgtat atggatttct tgggagtggc tagtgctcag agggcatgat

82921 ggagggaatg aggatacgct ctttgaaaag aactatgtgg tatgggttgt cagggtcagc

82981 ttatgcagga gaataattgc gtccaatgag aattctcgcc aaaatgctct cacttaagaa

83041 gatctggata atcagatgga caagctggtc tgctctgaag tttttttttc ctcatttttc

83101 taagtgtttt ttatggcttc ataaaccaag aataatggaa ccaaagagga tgcatgtagg

83161 gacttcatgc aagtctcacc tggccaccat gtctgctgaa gaccccattt gcagtgttgg

83221 tgaccaaact cagccttgat ctattactgt agcccagtta acctaccagc caggtcgggg

83281 tagtagggcc ccatcctcac taaaaatgac atagtctagg tttgggtttt ctttcccttg

83341 ccatcctcac tcccagcccc accatccaga atcagctgaa tgccttatct attccacaaa

83401 aaaaggaatg catttttcaa ccaaggaagt caccttccaa tgatagtggc catacagcaa

83461 gctgatgtcc acgagattca gcgcccctaa cacatgcttc ctcacctaga ctcatctgag

83521 cttataagac atcaggatga cctgctcaaa tcttagcacc acttgaagct gggacacacc

83581 tcccgaggct gaggtgctca cctgcaaaaa ccacctgttt ctctcaaagc aaaactttca

83641 gtcacaactt acatagaggt gaagctctca caaatgaact caataaccca tgtacaaaat

83701 gttgctttca acactacgca ctgggcctca ttgccttaga ggttttagga ccatagagag

83761 gatagagttt actgctgtaa cttcttctgt cctaagcccc agggaacgtt tgtattaacc

83821 tctcctgccc caagtccctg cacagaatcc cgcaggatga atgaccagaa tctctgcctg

83881 gtgtagagtc ataaagatga ggagaaacaa aatgcagtac ctcccacaac ctccctcctt

83941 aagccctttc ccctcatctg tacttctggt tagcttgccc ttgtcacact gcctttttcc

84001 tactggcatg tgtaagttgc acatgttcaa atcagtgcaa gtaggagggg ttttgacacc

84061 caaagtggct accgagtggc ctgttttctc aataaatcca tgctcagtgt gtgagagaga

84121 acactgtccc cacttcacac acttcagctg cgtaagaggc attgagtttg ctgtcctcac

84181 tgagggggag gaagaagggc tgtcacgtat agtcaccgca caggtgtgta cccgtccccc

84241 tctctgcggg caggagagac agggatgcag ggtgcaaccc aaggcgtcta tttagattcg

84301 actctgaggc tggacatggg tacgggctgc atcgaatggc ccagagttcc agccatggga

84361 gctcagttgt tacgaattgt attttcaatc cagggtacgt agttaaagat tctggtatag

84421 atgccaacat ccgctctcaa aacacatcca tctgcaaaag ctaggattcc ttgaagtacc

84481 ccattgcaga ctgcgggggc cccagtgact tcctgccagg aaaaaaggaa accaggttat

84541 tgtctggcaa aaggcgcaag gaccaaatgg gaagaggaca gtagggagag cagctcacct

84601 tgcagggcag ccttctccct ggcacgatgc ccaagcacag catgctttcc gtgatgttgt

84661 ggctttgata ggcgtcctgg cattcagcct tggggaccac ggagatgttc acgctctgca

84721 gtgagtcagg ctccttggct agaggaaaga ggagtagaat gtccatgagc ctgattggat

84781 gccaggccgt ctcctctccc tcccctctgt ttcaacacac ttctcctcct cccagcagaa

84841 agggaaggag gtccaacagg gagagaaatc agaactgtgg ccgctgggac aacaaggaat

84901 tattggctta acagtttctc gagatacaag tgtctgtcgc ctcacaaaac agattaaact

84961 cttggaagtt atacgtaaat gtgagagatc tctgcaatga catgaaagaa attattttag

85021 aaactcctag aagtttaaac atgtaaagag tgtcctttgg gatggaattt tacctattta

85081 tttaggtctt gttatttgtt ccctatttta aactcaccac tgactccttt tcaggttatt

85141 tgctgaaaga tacttggctt ggccctgacc agtatggctc agttggctgg gtgtcatccc

85201 acaaagcgaa aggtcaaggg tttgatttcc gttagggcac atgcctgggt tgcagattcg

85261 gtccccagtt ggggcgcatg tgagaggcaa ctgatcaatg tttctcatag caatatttct

85321 ctccctcttt ctccctccct gcccttctct ctaaaaatat aaataaatga aatctttaaa

85381 aaaatgaatt atatttgact tgcaaggctg tgtacgttta aggtgcacat gttgatttga

85441 tacatttaca tagtgcaata tgatggccat tgtagcaata gtgagcatca atatcacatc

85501 acataattac catttccttt tttttggtgg gaataattaa gatctagtct cttagcaagt

85561 tcaattataa tacaatgcta ttgcctatat tcgctatact gggcattaga tcttcaggac

85621 ctacttctct gctggttgct acatgtgtat ttttagttta aagatatgta gagtcctctc

85681 accttagatt tttcattgtc agatcaaaaa atctagaatc tgctaaaaaa aaatcactca

85741 aaaagtgatt ttcctaaacc ccatgaaact agaatttaaa acagggtctc ttgatttcta

85801 attcaagtta gtttctttat atcaatggct tataaatact atggactgtg tagacccata

85861 acaaagcttt tttaatgtaa taaaatgaga aaaacaagaa caaaataata ttattatgta

85921 tgttaggggt tcttgccaag gacttttcat tggaaagaag tattttattt tgagattttg

85981 tttttctaat gggagcgtgt ttatataaat gtctttattt gtaaatggta tatttattct

86041 tgaaaagaaa atgtttaaag ttctttgttt tattttctag tctagcaata attgtgtggg

86101 tcagcatttc ttctggaaat gtatttttaa tttttttaat taaaaaaatt tcaattacag

86161 ttgacattaa atattctttt gtattagttt caggagcata gcgtattgta cgtctgcaaa

86221 tgcatttcca agaaatgctt acttcgctac ttcatgagcc agacccaaat cagctagatt

86281 ttttttgtaa gtccctgctc tttgctaaac acaatgagtc aaacatactg ctcgtgagag

86341 agacatttgc ttattttcaa aactcagatt gactctcaga agaacaaaga ctttctgctt

86401 atctagagga cactcactct gacaagtgaa aggcagcaga cgcaggaagc caaatactgc

86461 acggtctcac ctaagtgtgg aatggaaaca cgctgcactc atggaaacag agacgagcat

86521 ggcagtcgct ggggccgggg caggagggca tagaggcatg ctggccaaag ggcacaaact

86581 ttcagttata aggggagtaa gttatggagg tttaacgtac agcgtggtga gtacactgta

86641 taatactata ttgtgtgttt tgtaactctt accacacatg tacacacacg aacacacatg

86701 taatgtatac atatatcaaa atgtgtacct acaccagaac atggcattat acacaatata

86761 tgcatttttt gttaatcata cgtcagtaaa gctgagggga aaacaatttc tactttttga

86821 tatattgaaa acaacatgcc ccgagatctg gaggtatgtg ctgaagatct ggtgacgaag

86881 gttggagcga tggcagcatt atatgtgtgt agtcacccct tcacgtctag tgaaagaaag

86941 tagaggtggg tgcatgaata taatccgaca gccttgttca aaacgactct cctcccatac

87001 tgtggtgtct gttggatgat gtatgttcaa tatactcaca gaagttactc agaaataaaa

87061 ttctatcagt gattttccct aagggacaat atcatagttc cgttttcctc ataattaagt

87121 cagcacccct tccccagtat tcacggctgt attgggtcac agctgcagtg gcttagaatt

87181 tgcgtgaaca ttttggaatg ggataggagc agacagcaca aaacctagac aagagaaagc

87241 tgtcttaacc taaagatttt ttttttatgg aatctgaaaa ttgcgacaca gaagaaagct

87301 tagaatgtat agttcagtgg tttctaatta tacatgggag acttttactg acaaaatcat

87361 cagggccatt ttttttgatt tcctgaataa atgagagaac gttcagaatg tcatcatttc

87421 aaagtgtttc ggaaatattt ttcttgaact tgtaattatt tatctgtatc acagccttgc

87481 atacggaaag tctgcctgat taagcacaat attatatata tacctcatgt ttaatacatg

87541 aatcttattt gatgctattt caagactcta agtttctcga ttgcctggaa tagtttgaaa

87601 acatctgata tagtctgcgc cacatttttg attcaagaaa actgaggctc caaggtgaag

87661 tggcccacgg ccatgcagca aattagggcc ggagtacaca cgtcttccct ccactaacac

87721 cacttctgtc gtgtacgaaa gcaaagatgc ctggctggcg tagctcagtg gattgagcct

87781 gggctgcgaa ccaaagtatc gcaggttcaa ttcccagtca gggcacatgc ctgggttgca

87841 ggccacagcc cccagcaaat gcacattgat gtttctctct ctttctccct cccttccctc

87901 tctaaaaata aatacataaa atctaaaaac aaaaacaaag atggtagaaa gaggcagtgg

87961 gggtggggag cttgaaggac cccctcagtg gggtgtgggg aaagggggag aggatggaca

88021 acgaggttag gagaaagagc cggagggacg agcggaggga ggctgagagc caacaacggg

88081 taggagcgcg tccgacgctg cgagaggatc gtccggccgt cactcacaga ggtcgcatct

88141 gttgtagccc caggtggaga cggtgcacac ggcgttttcg ggggctggtt ctcggggcag

88201 gctggccagt ttcacgtagt tgtcgactcc gatgtatctt tccaacttga ttaacagcag

88261 attgtgctca atagaaatga tcgagaagta cgggtggtta atcatcttct cctcacggac

88321 cacctgcaca tttcgttcca tgtggttgga ggggttcgta acccccagta tcaccttaag

88381 gttcctggaa caacatgggt gttcttgagg gggctgggtg gagacggctc acccgtccgc

88441 ctgggccttt attagctcct ccccgcagag gtacttttct gcccctgaac atccccagaa

88501 ggcctcccta acccatgaag gctcaacagg cagcggtcgc aggcgggggt ctccgcctgg

88561 gtgcaacctg agaaccccaa gtgcgaaggg gcactggggg cttacgactc acggtaagtt

88621 gcagctggca gaggcaacca cccacagggg gtggatcagg actccagcgc agggcaggta

88681 atcagacttc aagtaggcca ggtagggggg agtgatggta ttggtgtaat cggggtcata

88741 ggtcaaagca cctggaaaga gaaacataga caggaaattc tgaggttatt ttttcaggaa

88801 tccgttgaaa aaaaaaaaaa aacccaaagg aaaatgactt catagatcca aggtttgtct

88861 gcctttctca ctagtaccat taactgtgag ctgaatttgt aagctcgatc cttttgttgt

88921 aaactataaa gaaaactgta gaaatgtatt tatttcaaaa gccgaaaggc attttccctt

88981 ctagttgtca gtaaatatcc ttgcagggaa aacacaagct ttctcaaata ccttccacaa

89041 ctcagtgggc gtatgttaaa tcagctcacg aagaactgaa ctatttttta ccatttttag

89101 aatgctttga ccgatctcat cccgtccccc caacaaccat gcaaggcaga atgatgtaag

89161 tgccacttga agaacagacc aaatgctaag agagtcgaag gaggacagtc agctgttcat

89221 tcaacaagaa actgctgagg acctggtggg tgccaggctc cgtctgaggg gcttgggccg

89281 cctagcgaat acgacagaga gatatccctg ctctcacaca ttctggtgag aggggacact

89341 cagcaataac aaccacaagc ataagtgaat ggctcggtat gttaggcggt gatcagtgct

89401 ttggaaaaag aaggaaagag ggggaggtag tagctggggc gtgtgcgcgc gcacgtgtgt

89461 gggaggcact gtgcggaatc gcatcagtag acagggtaga tctcatggat gtgaggggcg

89521 aacagtgaaa tgaagctagg ggaaggagat actgtatttt gctgtgtata atgtgccctt

89581 ttttgccaaa aattttgagg gaaaaaataa ggatgtgcat tatacctaaa tgtaatgagt

89641 acaatgcatg ggtataataa tcccgtgttt tgtgtgcaca aaatgtgggt gtgcattata

89701 cacagcaaaa tacagtaatc agaggtctgg ggaacaggat cccaggcaga ggatgcagct

89761 ggagaagagt cctaacacct gtttaaggag catcacagag gccaatgtgg ctggcatggc

89821 gagtgagggg gggaggagga gggaaggaga gaggacaaag gagtgatgcg ggcggggctt

89881 tgtagagtgt ggtaaggtcg tggacttgcc ttctgagaga aatggggtgt gtggtgagtg

89941 ataaaggagg gatgatagtc cccttaggga gaagtgttga gtataggcgt ataaagcaga

90001 gaggcctgtt acaatttgct gatccaggca agagatggtg gcttggacca tggtgagcag

90061 tggaggtggt gagatgtgat tggattccgg atttgtttag aagacagagt caacaaaatt

90121 gtccaaacag catgaacgtg gtgtgtacca gaaagaggat ttcggattat ctccacagcc

90181 ttggcctgaa taattgaaac cctgcctgcg ctagcccccc aaacccttga atgctcttct

90241 gggtccatta ttttggactc ctgctttcac cccctgcctc cacttctcag tgtctcatac

90301 cgaagtcagt tttgctctgg acaggccgag gggcagctct cagtcttcct aaagaggagg

90361 tccattactg ggggtggggc aatggccatg cctcccccaa aagcgtgcaa tagtcaatct

90421 ggggtgaggc aggcaggtaa tgtcatagga ggcagcctgg ggggatggag gcagtggcca

90481 gggatagaga accaagccta cccttgcggt ctgggatgtt gttcctgtct ttatccacct

90541 ttcctccccc cctaggattt ctctttggcc agtgaagaaa aatgtgcaga tactcacaag

90601 gcaggttcaa ggtcacccag aagaggataa acttcatagt gatattgaag gccttttcaa

90661 agaatgaggt tcagctccgg gtctctggaa gggatgaaga gaagagagag agagagagta

90721 aaggactaaa taagatgagg caggatgggg ttagggttag aaaacattgt catctgaatt

90781 cttgccgtgg caggattcag ttccaaaaga ggtgtttgtc agtcagagta ggtggtggtg

90841 tcaagcgtca gaaggacaag aacagacatc aggtgacttg ggccgagaca cggtagagaa

90901 cagatcagtg ttttgctcac tgtcagatta gaaagtccct aatctgagag acgtggaagc

90961 tcacagtgtg tccccagcct cctcccctct ggcccctgct ttgtcaaact ctgaagtcaa

91021 gtccctccct gagctccctg cagggtgctg gccccacccc tctgtgtgat gcaggcccct

91081 ggttgtgtgc ctctgacatt catctccggt ggctgtagcc cggcatcctg tgctgatctc

91141 agcccctgtg tctgactgca caccgcctcg agatacatta gccctttcct tcgtgtcact

91201 actcccacct ttatttattc agccagccag cccataatat atattatgca tcagatttgt

91261 tgaggatgcc taagtgggaa gacactgttt ctgcctgggg gcacagagtt aaaagcactt

91321 ttgtgaaagt gtgtgtagga aaggagaacc cagagactac actctaccca gggctcagag

91381 aaggggaagg agcttgtccg gcataaaagg aagagaaaaa gtaattcaag aagaaagaaa

91441 gggccctggc tggtgtggct cagtggatag agtgccagcc tgcaaaccaa agggttgccg

91501 gttctattcc cagtcagggc acatgcctgg gttgcaggcc aggtccccag taggggggca

91561 cactagaggc aaccacatat tgatgtttct ctccctctct ttctccctcc ctttccctct

91621 ctctaaaaga aataagtaaa taaataaata aacaaatatc tttattaaaa actattaaag

91681 aagatggaaa ggcttctgta taagcatgaa catgtgaaat gacatggtga gtttgtggat

91741 ctatgagtat tacaggaatg ttaagagctg ggaggagaga caagcaggtt tgaatcacaa

91801 aaagccttct tatataagcc ttgcatattt aaggcttgca gaagtcttca gtgtgtagat

91861 caggagccaa taaattcatt attcaaccaa attctattga atattgtgga ggacagacct

91921 gcccatgttt tatcccccga gtaatcctca attgcctccc cttgagtatg ggtgggacct

91981 atgacttgct tctgactagt ggaacatggc aaaaatatgg gatgtcctcc ccacggttgt

92041 gtttctgtat atgcctccac ttgctagcat gcaaagagag attcttccca ctggcttgat

92101 gcagaaagcc cacttggcaa agaactctgt gtgggtggcc tctgtgtact gaggagaatc

92161 ttaaggaccc cagggcagct ttcagctgac ggccagaaaa agcagaggcc ctgtgtcata

92221 caaccacaag gaaacgggtt ctaccaacca cccgaataag ctcagaagca aatgcttcct

92281 tcaaaacctt cgtagaccct aaagagaaga tccaactaag ctatgcctag acccttgact

92341 tacagaaact gtgatacaat aaatgtttta tgctgttcaa ttggtggtaa tgtgtttagg

92401 caggaataga aaactaacac atatgactat tgtgtgctgg cattctatta gatattgatg

92461 atgtaatggt aagagagata aggatcgagc tctcacagat cataaggact aataggggaa

92521 acagggggat gtttccatga atgtcacact ctgggaagta aagagatagg gaaacacata

92581 gtggagttgc caggtaagga tcagaagaag gccttcttga cgaagtgctg gttgattgag

92641 gctagaaggg tgaggagaag ttaaccaaag gaaggcatga tggaagcaat tcctgggaag

92701 atgaaccagg aatgaaaaga gagcagagtg cataccaaag tgtgaagaga gacagagaag

92761 acttttacag aatagaacat tcaggaattg tcaagaatgt cctgcgagga agacagagga

92821 ggcaaccaga gagtagagcc ggggagaagg tccttggcct ctgtaattta gatgggagag

92881 gctttatctt gctgagatgt ggacagaaag aatccaatag attgagggag ttgaaaaata

92941 aggtaaaggg gacataatgt ataaggaagt ccatgaggaa gtcatagaag atgagatcca

93001 tatgaagggt agagataaga agctaaagat agtttaaaaa acccactatg cttgtaggtt

93061 gggtgtggga agttacacag aggtatcttc tgagggtttc accatccttc tcctcccaga

93121 gctcgtctgc tgatggtgag ccctgaaggt cacgggagag aggaggatgt ttgatgcggc

93181 cactatggag aattgcacag atcatggact tggagaatat agaattgtca gtaagcattg

93241 aagacataat ttcagagtta taaaaagtga tcacagcaag gcacattggg caatggatca

93301 gaggcaggaa gcatggagaa actaggagtg aggctcctag ataaggcaag cagagaggac

93361 tgtggtttgg atgggccagc tgttacaaga aaaagcagca aagttgaagg aaatggcctg

93421 gtgtccaaat tcgagataat ttttgtgtac gtgagaggat taagctgtgg atggatatta

93481 gtcagaaccc agatttactt tttgtcttgt tttgtttttc acttcagtag gggtgagaag

93541 cattcacgtt caccatatat caatgtccaa gaaccaacct acctctgcaa tctctactaa

93601 cacacggatt cacttctcct tcaaaggatg gatctcctaa cctcctcatt ctgtgcagtc

93661 tctttctcca atggcacaat tctctctccc cccacctccc tgacccatgt tacactgccc

93721 ctctcctaca tgaaagtcca tggcctctcg aatatcagtc taaaccctaa cttttaggtc

93781 cagaatcctc tcaactctgt ctttttaggt atcttgggtc tgggctgcta ttcatctctc

93841 caccatacgc cattgtgaca gaaggactaa gcattcttca gagctcttcc tagtagcaca

93901 aaactctacc aaagcctgaa actgctttcc ataaatacag atttccccag ctgctatctg

93961 ggatccagat ctttgtctcc agctgtctgc tagaaatgat ggcctcccaa tcttccaagg

94021 atgccagcaa tcacatgccc catctctcct aattcattct catgctcctg atctttcaga

94081 gatgggaaca ttggttctga tgatattgtg tttgagaacc accacctagc tagttaccta

94141 agtcagaaac ttgggagtta gctcagacac ttccttcttt attcccagtc aaactcatct

94201 gtggatttat ggtacagaca cagcttcctt actctctccc aattcagaaa tctggcgata

94261 ctctctctag catggagaca ttaataacac cctctctgat gtctacaaga caagaaaatc

94321 taaatttctt catagaacag gtcaggtcct ttgctaacat tcactcacct acaaacagca

94381 gtcttttata acattttctc accattgtgt ctccagtatt agttatttac agatattttc

94441 cagaacatgc cacatttttt atgctttttc tgaattttac ttgagtatac cctcagtttt

94501 acaatattgt ctcctgattt gtctgtctct ttggtgacac ctcctgtgac atgttcttat

94561 tctatgtgct cacaacacat tttgctcatc atgattacgg cagttatcag aggctattat

94621 aattgtctgg ctgatggcaa agactatgtt gtattcaccc atgcacccct agcataggcg

94681 gtgccctctg agtgcacaca catggtgggg atgttgctga atttctcctt gttctcgata

94741 tgaggaagag gtcagggatc acagcatgaa tgtattgtca ctttttattg ggttttaaaa

94801 tgtggattct aaatgtggag ttggggatcc agtatagaag tcatgaaggg gttgccctgt

94861 ggggagggag gaacacggga gcctcagtgg ctcttaatga tttttaggat ccatctggcg

94921 taggggtgaa cttctgtgaa gaatccttca cttcccaggg tgacactgcc ttttgcccag

94981 gacaagattc catgcaacct cccagcacag acagctggag ccgctgaaac ttcctgccgg

95041 gaaaggggaa cagaaggtca atgtcattct gtcctcatgg aacaagaata aatcccaaca

95101 agtggcacat tagagtagca ggggcattga agggactgga cttatttttt tgtgtatttt

95161 tttagaggca acaaccctct cttcacatcc acacaggcct cacctgtctt gggtcaagct

95221 tggcccaacc tgagagcaag caggtaaaga aaggacagat ggttctactg aaaattcctt

95281 ccactctgaa ggagggaagg ggaatggatg acaaacccag agagtgagtg gggaaaggag

95341 aaggacttca ggcctttggg ataagtatca ttggattgtc cacagtcacc ttccctcccc

95401 gtggaccctt tgctgtcttc ctgtccgggg agaagggaag ataccttaat tgcatatagg

95461 gtgctcagag gttgtcccac acacatgatg ttggctgtcg ttctttggct tagtataccc

95521 tggcactcat gggaaggaag agaatattgg ctcgtccagg tgaggatgtc gggatcactc

95581 actgtgggaa tgagaagaga aatgaagccc agactgactt attctcctga tccaggtcct

95641 cctgggatca gcctccgttg tgcatatgca tgaatccctg agaatattca ttgccggtag

95701 taaacattcc aatcccctca gagtccctgg ttcagtgtct ctgtgtcaga acagtggatt

95761 cagggcagga gtccctcctc acatcggacg tcggatccga gtcctctcca ctatgcctaa

95821 ttcagaacct ttgaggaccc aggagaaaat catgagtgac tttgagaaga gacagacaca

95881 aagccttcgt ctccaagaaa tctgtcttct cagcacctac ttcatcttct ttaaatgcag

95941 aaatagaggg agaaaactgt ttttaatgtg tttaagagtg ttttgtggtg cttattttta

96001 aatggattat ttataagtgt agatgtgtat acatgtaaat atatgtacat atttccttta

96061 agtagttggt agtatctcta tggtagcagt tgttagtatc ctgttaatgt cagggtgcaa

96121 ggattctaat caagatatta tacaatttta atctctggta aagaatagca aggaggaaac

96181 aaaaaactga ccagagattg ctcaagcaaa acttaatgat ttgtggagca agattttttt

96241 cctggggtgt aaccaatcta atgtcctaaa gtagattaga ccagaaagtg tcaggtcaga

96301 aaagaaagga gttaggttca gttttcaact tttaggggaa ttgaatcaaa atacgtaaaa

96361 caagctaggt gaaagtagtc tagttacttg aaataatgaa ctcccaataa ctatggggag

96421 cctcgcacca gagggcttat agaattagtt tgtcaccatg gtcctgacct gcaggggaca

96481 gaccctcgcc atgggcttgt cacacagggc ctgtgtgata aaacgttcct atagttttat

96541 tttgttttca aatggtatgt aataacttgc ttttgtgatg tgtgatgtca ttattttgaa

96601 taagacttat aaagtcaagg ccaaaggtca taaatctttg acttgttcag aaaacatatt

96661 cacttagatt cccaggaatg tggctgctaa actgaaaatg aagttgccaa cagggaagaa

96721 tgtaaaagtt cccacgtttt atttgttttt gcaaaagaga attattttaa aagttcccac

96781 ttggtagttg tttttgcaga acagatgact tgttctgtag ccacgaccgg ggtctaaatg

96841 tgatgattac tgtccatgac attttgcctc agtggacaga gctttggcaa gtcaatcaac

96901 tgaggtcact tttaaagtca gtcaatcaga ccactccaaa aaacattcct cagaagacca

96961 accaatcaac aacaacctct cccaagtaac catacttctt acagatgagg tcaataccct

97021 ttagcctctg aaagtcctct ggtccctgtc cacaaacttc ccacaaaacc cgcatgaacc

97081 cagtagtctc tgctcagcga gactgtccct gctcagcatg ctcttcctca ctacaataag

97141 ccatacgttt agctttatca tttcatgtca gatgttgagg ggtggctcca tcatcccttg

97201 acaataccta aaaagaacat aatggtcatc agcagcccca tgaagccaag gccaccaagg

97261 accgacaaat gtgtaaggaa gcaacgttct tcatacaatg cccttcatac aatgatccag

97321 cagatctctg ctcacgccaa ttagagctca gagggcggag ggtgcctgca gcatgggttg

97381 cagcaatttt tccaatgcct gtctggccag agggacaccc agagagacac catgagcgtg

97441 ttatagcact caactcctgt gaaccccgcc agtcccctcc cctcagtggg aaccatcgga

97501 tggagccgca gatctaagag gcgcactctc tttgtggaaa cagatccggg acggaagaca

97561 tggctccccg agccaccccc gtatcgccca gggtccactc gaatcaccat cagcacaggc

97621 caggttgtct gtggtcgggg tgggaagcat cctcagctgc cttccaacag cacatgtttt

97681 attttggttt ccccactgct gtttccatga tggcttggtg ggcttccagt agccgtccct

97741 gaggcagcaa ggcaccctcc cacagtacac cttgatatta cccttagagt gagccactaa

97801 cccaccttta tttaaagatt ttacttattt atttttagag cggaagggag ggagaaagag

97861 agagagaaac atcaatgtgc ggttgctggg ggtcatggcc tgcaacccag gcatgtaccc

97921 tggctgggaa tcaaacctgc gacactttgg ttcacagccc gcactcaatc tactgagcta

97981 cgccagccag ggctcccact tttatttaaa ttcagttccc aggatgcaga tcctggttct

98041 ggagatgtaa cacttactgc ctattctgtt cgttgatgtc cattgggctg ctctctatca

98101 gcctctctga attttatcct ttgacctctc atgtcagtat tgttaggtcc tgaaagcaat

98161 cgctctgaag tcaagaacat tacctcggag cccccattcc cctcaagtat atccctccag

98221 gtcatcccca ggtcgaagcc gaacacttac ggtttttgta ttcattccac ctccaggtcg

98281 ggatgaagca ggagtcgttc agcaccaaag gttccagggc gatggcgatg gttcccacgt

98341 aggggttgag ggccacggcc tcggacagtt ttatcatcat caggtcattc tccagcgtgt

98401 gcgtgttgaa ttcagggtgg ggcacgatca gtgagtaatt ccgtatctgc tctttgttgt

98461 tcttgatggt gggttggaaa actcccagtc gaattttaac gctttgggag aaagagggca

98521 gagagacagt taacacacac acacgtgcac gcacgcacac acacacacac taaaacaggg

98581 tcataaaatg tcagaactgc aagatacaac agaggcaact ttctacaatc ctcttaattt

98641 tctctcttag acctgagaac caaaatgacc tcccttggtc tataaaatag tggtaatagc

98701 atatagcatt gttatgttcg agtcaatgta tatataaatc ctgacatggt acctgccacc

98761 caggacatac ctgtgcagtc taattgtgat ggtgatgatg atgatgataa tgatgctgat

98821 gatgatgatt tggataatct gattgagcag gcaatagaga tggcacctca atgcattatt

98881 ccagagccct tgcctgtact acactgtctc aagaccctct tgagaaagca tacagtgcac

98941 ttcgcttcta cacatcctta ggcccctccc agctcctccc aggcccatgt cattgtacgg

99001 aaaccacgtg ccgcctgcac tgatcacgga tccacccagc tcttggcagt ctgtcaggat

99061 tccccttcac cattttccat tatgcccccc ggacaaactc aaacccactc gcttccatcg

99121 ccagaaatcc agtcgtgtgc cagatacgag agccatttgc ctgcggtgga cagttcacgc

99181 agaatgtcag tttcttggag gggatgacct gtccttattt cctttgtatt tctcacaatg

99241 tgcagagcag gggttccgtc tgtgtttgag taaactgtct taagcagcca ggtggctgag

99301 agctctgagc atgaaaagac ccttgcgtgc ccctgaaact ctgaacaggt gccggtcgca

99361 ctgcgcactg ggacccagtc acaacggaga cgctgagatt tgcaagggac agtgttattt

99421 tgagccatgc actgttgcca ttctcctgcc tggcgctttg cccttgtccc gggaattgcc

99481 tcgccctctc ggtatactca gctgacagcg gtctcccaga taccctgact gcctgggcac

99541 tcgattccca ggctgtaggt acttggctgc cacctcctct tcccgtgggt cccttctcgg

99601 caaaggggcc gcacgtggcc acccgaacgg aagtggatct cttgtttcct tcctgtttat

99661 tgcagcgtgc cactcttttc atgctttctt tttggctgtt tgtttatttt ggcacttcct

99721 ctcctggaat gcaggctcca tgaggggaaa tttatttcca tctttttccc ctcgccattt

99781 tctcagtgca tgaagcagtc cctgccacat agtgagtgtt tagaaaagac actgagtgaa

99841 agaatagttt accctccatg agttagagtc ttctggcacc ttcctattgg cctctcttca

99901 tttaagaaac actttattta tttattttta gagagggaga aggagagaga gagagagaga

99961 aacatcgacg tgtggttcct tctcacacgc cccctactgg ggacctggcc tgcaacccgg

100021 gcacgtgccc tgactgggaa tcaaaccggt gaccctttgg tttgcaggtc ggtgctcaat

100081 ccactgagac acaccagcca gggctactga cctctcttaa actgacaaat gtgtatgatt

100141 ttccccattg ggaagaagat cctctctctt gctggggcct catagtcctt taggatgcca

100201 ttagcgcgtg ctggaatgcc tcaccagacc cccttttgtt taaccacacc cctcctggcc

100261 cccgagcaaa ggggtactca cggtaagggg cagtgagcag ctgtcaggat ccactcgggg

100321 ctgatgagag accccacgca gggttccggg ctggactgca ggtaggccaa gtaaggaata

100381 gtaaactcgc ctggtgagct caacttttcc tttttagact cgttggccag aacgaccgct

100441 aaagaatgga tgcggtcata ggtagaaaga aagacaacaa ggttgagagg tggccagggt

100501 agtttttccc agcccaaggt tttcgaaggt attttcgagg ccttccaatt atattccata

100561 ttcgtcttaa atcagggaga accaagtgtg ccgctcaagc cgcccacgtt aaggacaccg

100621 acacctgtcc ctgccaggca gggaaggaac aaggtaaagg cgccctgact ttgcaggtgc

100681 aagtgttagg cagcagctgg gaaggagcag ctggaggaga aagaaggcgg ggccccttgt

100741 cacgacagtc taacgagaac ttaacacggg aggctggaga caagggggag tcacgtctcc

100801 cccactgacc cagccgtcct gaacccggtc cgatacaatc gccaggtgct ctgagaatca

100861 caagcaacac tatgagagct caggaggaga ggagccctcg aaattctcag atgtcatctc

100921 cactagctga gaaagaaaac ctttaaaact gtgggttaac cccaaggtct agaagtggga

100981 cgaagagggg aacttaggca ctgactgaag tacatcaccc ctgaacccca atagtcagca

101041 cactcagatt aactgagggc ccacttgttg ccaggtgctc aagtgtctag aacaaactta

101101 ctaataaact taaaagcaaa ttcactaatg attttatttc ctccataggg gaggcactca

101161 gactagacag gtgcctgctt gcagcttcgg gcttgtatgc gttccataaa tatacttgct

101221 ctcctcccca ctatatgtgc ttgtctcttg tctctcgtct gagatcttct ctggtgagca

101281 agggaagaac tgaggagggg gagctcctga gtcgggtctc cccggtaaca tcttttagga

101341 gggggagccc cctgacttgg gtttcctggt aaccaaaggg atggacaagc ttggggaggg

101401 gccacgaggc ttcccatgtt ccagcccctg cagcccaggt tgagggaggg cctccttttc

101461 ctgtggccgt gcagaaactg aggccgtcag cacacaccac gctcaagagt caggcactta

101521 ccaggggcac atccaacagg ttcctgggcc tcggagggcg tggggagagt gctgatgggg

101581 tgggctgtgc ctcctgagcg agtgtggttc cttctgtttc cttcatcagt tacttgaagg

101641 aaatgccctc agcttcctag cccttgcact gtggccagtt acttcccttt ctgagcctcc

101701 cccgccccca acccatctct cccaaggcgg cagcaatggc tcagactctc accagtcgcg

101761 cccaggaggg tgacgatgag acagaacttc atggcggccg cgatctcacc aggagcagac

101821 atgtgggttc aaactggaaa caattccggc ctttgctcca agtgtctccg ttaagaacag

101881 ctggggagaa actttaactg ggggctgggg gagacgcagg gatcttacag gagtcttcaa

101941 actagtccgt atccaacccc tttgtaggac aaaagccggg agctgtgcct gctgtgggtg

102001 gaagagagga aggagcccac ccagtcaagc ccctggtgcc tcttctctgt ccctaaaaat

102061 ctccagagtg gtgaggtgtc cgcggacact ttccctcccc tctctgtgac atagcctttc

102121 tctgttgtcc cctcctgtct ttgtgaccgc caccctctct ccccgccctc acttgccgcc

102181 tccccttccc cagaggccct cagagaaccg tctcacagat gcatgaaaca ctggggtagg

102241 agacaaattt aatcaatcgt gccctccccc gccccccgag ctttagagat gaggacaccg

102301 tggtccgcag ggaagtcggt gcagcgtcag gacttggacc tgagcctgca gaggtcctca

102361 gacaggttgt tccatgccgg agccctgggc ccccggagga cagcagtggg caaaaaagtc

102421 ccctgggcgt tttgcatgct ggcaaacggc ttgccacgaa gaaataccct tgcccatgtg

102481 agttagagga gacttttcga ggctcccctg agacttcaga tgaccccttg gttcccaggg

102541 acaaggtcaa gcacagacct tccccctttg ccctggacgg gacacaggcc ctccagctcc

102601 tcatgctttg cctcatgaat gattagctga gcttagagct gtttccctgg aaacgggcca

102661 aacatagaca gctgaccgag gagcgcccca attgcaaaac tgcccccctg tggatctccc

102721 cacctgcacc ttgtctgttt ctcccaataa aaatctgagg caaaaccatc ctgctgagag

102781 attttcttcg agtccagaat gtcctcttat tgcaagagtc tgaatcaaat cgatattttt

102841 acctgttcag gtgtctttga cgcttgctct ttctgatgca ccacgctcgt ctttgtaagg

102901 tagccttaca tgcaaacttt ccaatgcccg ctccccaagc aatggaaaac ggtggggtca

102961 atcagggcta tgcaggggtg tccaacctgc gtcccgcagg ccacatgtgg gccgggacgg

103021 ctgtgaatgc cgcccaacac gaaatcgtaa atgaaaacat tatgagattt ttgtgtgtgt

103081 gattatatgt tgcaatgtat ttaagtgtag cccaagacaa ctcttcttct cccagcgtgg

103141 tccagagatg ccgaatgttg gacaccccta gatggccttc tgcggtgtcc ttctctggcc

103201 ttggggtctc agtagtgctg gccccataga atgagttcgg aaggggtctc tcctcagttt

103261 tggggggaag agtttgaaaa ggattggtgt taattcttca aatatttggt agaattcgcc

103321 agcgaagcca tctgtctctg ggtgttctgt gttgggagtt tgtttgtttt tgctcacggc

103381 catctctttg ctggttattt gttcagattt tccatttcta aagtgcttag agtggtgacg

103441 aacaggggcg aatagtcaac gctatatcaa tgcttgtgaa ataaaatagc tgtaaccttg

103501 ggacccccat gtttcccagg cttctgggga cattttttta tctgagtggc cagggattgt

103561 tgtgaagtcc ttagatcttt gcagcatttt catgtttgtt aacataaccg caaagagcag

103621 agaacacggg gtcagacttc tagggctgca atcccagcct cgggacctcc gtgagcccca

103681 ggattctaaa ctacaaaatg agagaaatgt tagtacctac ttcataggat acttgtgagg

103741 attaactgat gaaatgttat gtaaaagcac acacatagca tgtggcacat agaaagtcct

103801 ttgaaatgct tgtgcttatt cgtacatatt tgcctctgcg gaagcaagca gtgtgtcctg

103861 aattctgcca aggggcaaac cctcagcgca tcccccccgc cccacagact gtgtgttcgg

103921 ccccagctgg gcccggagca cctttcctcc tggaggcggg gattcagaat tgtcacacgc

103981 ctgcagctga aggtgtcccc agttcagcag agcttcctct actgagagac taggttatgg

104041 agtgtaccga gtagacgtaa attccttcta cccagtgtat acagcctacg tgaaaaaaaa

104101 agttatagct aagcttcgac atgctgatac tgctttatgt ttcatgcctg aagaaggtcc

104161 taacctcgaa gttgacaggt gagagccatt tcctcagaat tttctggagc aagtgataag

104221 taaattgacc tgttactata attagacaaa acaacaacag aaacaaatgt tttatctttc

104281 tgcattgtcc ctaatctcag acggttccct cgttacattt ccttagtttt gctacagaaa

104341 taccctgtct ttctggatct ttctgaatca ggaattcagt atttatttgt ggtaaatgta

104401 ttttggtccg agctctctct tctgactaaa aaatggtttt tattctcaat cgtagtttcc

104461 ataggtcttg atgcttctcg tggtccgtta gaagcagatc tgccttctgg tcacccactc

104521 ctttccccgg tttgccgggg ggcatgggcg ttgatcagca ttacagtccg taacctctgt

104581 ttgtttccgg tttacccagt gagcacgcac gtgccgtgga gacagggctt tgactgtgtt

104641 cttctctaat gtatctttag tgcctcaagc agggtcaggc acccaacagg ccctcgataa

104701 gtgtgtgtgg agtaaaggct cggacacatg gagagaaggg ttacattggg tcccccacag

104761 gacacaccgg gtatgtttag tcaatgctct gctaatagat atgagcgttg ttgctggaat

104821 ttcagggttc ggtagacaat gcactgagaa acatcctgga ttattctaat ggaggtcgtg

104881 gctgccttct taacacctac ttctcttcct cttcataaca acccttcgat tattttgtta

104941 accaatcgtg taccatgtag gtcatgtcta tcccagagag aagggtccac ctctcacttg

105001 gatggtaaac tgtgaaactc aattgtaagt taatagtgta atcgcattta acgaatggct

105061 cagggatctg taagtgacca aattcctata aggtgatacc atgcagtgtc tgctgggcgc

105121 tcccgggaaa ggaagcctct tagaccttct gggagtcact ctgtttcttt ctctccctac

105181 atgcacacca gggagagcat tgccttgaga ctttagtctt agggcaaagc tggcagtaac

105241 tcggagagca aacgtaaaga aagtgggtcg cactttattt gttgataaca tttttgggat

105301 caactcgtcc ttgaaggcct tcctaattct aaacttttat tagtgcattt tttaaggctg

105361 tgtgagttgg atattctttt ccttgcagcc atgtgattcc taaagcatag tcacatttat

105421 acattttata tacatatgtt aatgggatac atttctagaa gtcgattcct gggttaaaga

105481 gcatacagac ttccacctta atataaattc acaagttgtg aacggctttt aaacaaaaga

105541 gatagaaagt gatgagatgt gctgaaaagg gacttgtggg tggcaccaag aaacatggtt

105601 catttgtcct ctcatacttt tttttgtgct gccctggtct ttcttctgtt accatctcac

105661 ttatcctgcc tctgagtctc tcctctgtcc gcagtggttc ggtgaagcgc tgtgtattga

105721 ggatgggggt gaggtaggtg gaaacccatt tcagagggaa cgggcatctt tcaactcatt

105781 tgtttctggg gagaagcctg agatatatct aaggctcctt actttaataa ggagaagcta

105841 tagatgtaat taatttaggc caaatgcaaa tatagaagca actcaatgag aaggaagtaa

105901 gacaaggccc attaattatg accaaatgtt caagaccctt gaatgcagag ctcaggaatt

105961 tggactttat cctaacagca gcagcaggcg atgttaaagg gtcagggctg ctccctgcaa

106021 gtataccacc ccaagttaca aagggataga aaatttatta tggggacacg tgtcttccta

106081 tactctcctc ccccacgata cccactgtgg actgtttgtg ccccccccaa aattcctaag

106141 ttcaagccct gacccccaga gggtcggtat tgggagttgg ggcctttggg aggtagttag

106201 gtttggatga ggtcatgcag gtggaatccc atgatgggac tggtgtcctt ataaggagaa

106261 gacgttagaa ctagctgtct cagccacgtg aggagaaggg acaaggtaac tgtctataag

106321 gaagtgggct cttaccactt cctgctggcc cttgatcttg gacttcccag cctccagaac

106381 tgtgagaaat tgtctgttgt tcaagccacc aaatccacag ttctctggcg acagcagccc

106441 atgccgatga aggcacagct ctccttcacc tgcccactgg gaccacagac attttcctct

106501 ttcttttttt ttaaaagatt ttatttattt attttttaga gagtgaaggg agggagatag

106561 agagagagag agagaaacat caatgtgctg ttgctggggg ttatggcctg caacccaggc

106621 atgtaccctg actgggaatc gaacctgcaa cactttggtt cccagcctgc gctcagtcca

106681 ctgagctaca ccagccaggg ctctattttc ctctttcaga cctcccccac ctactccagc

106741 atttccatgc tcagccctcc tgtgtctttc tccttgagcc gtgccagaca tgcccagaaa

106801 cctcgcgttg ggactcgtgc tcccaaccgt cctctgggtg actgtggaac aacccagagc

106861 tggggagggc gttgcagagc taaagcaggc tgcccgtgct gcctttccag acggaacaga

106921 cagaacgaac atctgctctc tctcaccaag acttctgtga ccttaaagta atagatctat

106981 gttctgcaat cttatctgca ttcatacttt tttgtttttt gttttttgtt tttttgcata

107041 tacttcaaaa tagtacgtaa tgtgggctgg aggccggaga gactggtgtt ggaagagagg

107101 cccaaattgc cgcaggcatc atgcctgtaa tagcaataaa atcccagtta gaaaagaaga

107161 aagatcttgg acagccagca tataaatagg tgaaatcttg aataatgcag gaactctgaa

107221 attttaaaag ctttctcctt ctggagatgc tccttctcca gcctcgcaga gtggcatgga

107281 gattagagaa aaacgtagcg agaacgcagg gtggcagctg cagggacact tggggagtga

107341 ggacagccag ggcttcctcc cagtgttccc aggcactagc cgagtgactt tgggagggcg

107401 attttaactt tctgccttgt agtgtcactg tctgcttaaa aatgtgatgg tgacagcgac

107461 ccctcccctt attcatctcc atatgctccg tgtcccatac agcctggcac agagtaggtg

107521 agcagtgaag tcattctgat taggtgaata cataactaaa caacctttca aatcccaccc

107581 tcatataata gtctatgtcc accctctgac acagcagcca gtttcaaggg aaagcggatg

107641 aataccaagg aggccagttc tcggacatat ttgatgcggg acagaatgtt tttatttagg

107701 cactgtgttt gggcatggga agaaggcgac gtcgtctggc acggaggtgc acttagttgg

107761 cagcgatggt gttcttgatc cagctcacat agttgcagac cttggtgtac acgccaggct

107821 tgcccttcaa agcgcagccg tagccccagg agacgatgcc ctgcagctgg ccgttgcaga

107881 ccacggggcc gccggagtca ccctgagcag gagggagaag ccactcagaa ctcgcagcca

107941 tgcccgcatg ggtaaaggct cgatgtccct gcactatccg ggggccatcc cttcaggctg

108001 ctggtcctgc ataaagcccc ttctctcccc attagacatc taagcctcca tctccgtacc

108061 gcatccccat tcttcctcct ccctccctct agacccttct tgttccttag ttgtccaaga

108121 agaggtgggg atgggccagc gtaaggctac cctgcgagcc ctgctccttt cagggagcgc

108181 ccttggatgc aggcctcctc cggagggcgc cttggatgtc ctatttgcac atcatcctca

108241 gtgtatcttt ttacccttaa ttcgtaccat tcatatggca gaagaaaaaa cgatcatttg

108301 tcacaagcct acccctcagc cctcatgatt ggtcccagag aaaacctttc agtgttctgt

108361 gttccaggag agggaaaatt gggtggaggt gggtgaggtg gagtcggaaa gagaaggaaa

108421 gcgaaggaac tcacctggca agagtccttg ccgccctcca ggaagcccag gcacatcatg

108481 ttgttggtga tctggtctgg gtaggcattg cggcaagcgc tgtcggagag gatgggggca

108541 tccagacact gcggcagctc ggggtagtta gctgttgggc gcgggagtga aaatggaagc

108601 aggttatccg caggattcct ggcacattgc tgtcccacgg tctctcccct ccttcccaat

108661 gctgccacgc tttcatccac gtggccaata atttctgctt tggaagaaca acttttatgt

108721 ggaaggcctt ccaaagagtt ttgatccgca ggctgttgcc tctctgagac cagcatctgg

108781 gaccacagat ctcaaatcta gagacttcct ttctcaagca ggtctttgcc tcaggcagct

108841 ccaaacccct cagcctcggc tctgtgtacc actcagttcc cagttctctg caaatcacta

108901 ctcacttccg ttgactacct ggcatcttta ttacagctca tttgtgctgc gtttttcagc

108961 aaaggactct tcctcattcc catgaatata ttctaaggcc ccttatgtcc acggactccc

109021 tctgggaacc ttgtctcgag cggcagtttg gactaactga cccccccaca cgcccttgac

109081 ccccaggatt ctcagacctg gtgctcgtgt ttcatgggac acttactgcc gacgctctgg

109141 gtgttgcccc agccggagac gagacactgg gtgccaacac ttgcacaggt ttttggcaga

109201 gagacagtgg agacgcgaga gttgagagtg gcgggcgagc tcagtttaat cagcatgatg

109261 tcattatcga tggtctggga attgtatttg gggtggcgga tgatcttggc cgcattgatg

109321 aattgctcgt tgccctccgt aagttcgatg ttgtactctc ccagacgcac ctggatgcgg

109381 ctgggtgaaa ggattcaacg ttctctaagt tttcaacttg agctttcccc cttcctggcg

109441 aaccccagtc acgggcatgt gcacagacac cacccgcttc cggcattcac gcacacaggt

109501 agctgtgcac ggaaggccgt cccagcagac acggtgcagt tagagtatcg cttaggtggg

109561 tgtccctgca ggcacggact cacagccccc gctcttgctg gaatagcgag gcgtaggaat

109621 gggaaccgaa tgacaccagt gagacgacgg gagatgaggg gaacctgccc tcgctccccc

109681 ggttgggatg accaattatt gtggggaggc gtggcagcaa tcagagtcac gggagcagaa

109741 ggagcttcct tattgtcaat gtcctggaaa tttcatgtgt tttctcacct ccacagggtc

109801 ttgtgtctgg gtgagctatg aggattaacg ctctcaagct agttgtacgt gtacttcgcc

109861 acgccctatt cccctcccac gtccttattg gggaggtcat ccgggcactc taagggcacc

109921 caccctgtga aagtccctcc acacatcgtt cagtgcctgg gggaacaatg acgtctctag

109981 aacatgcatg ttcgcccctg aatcctaact ctgttggtta ccccgctcgg gctgtggcag

110041 ggccggggga cgagttgatt ctaagcacag ggaggtgagc cttggctact tacgacttgt

110101 agcagtgagc cgcagacacc acccactggt tgttgatgag ggagccgccg cagaagtggt

110161 agccggcgtt cagggacacc tggtagggga tgctgttttt tttgcaggtg tagcccccga

110221 cgatcttgtc atcgtcatca ctggggaaag caactgacag aggaaagttg gaaaactacc

110281 tgttaaacag gtccatcttg gcatttggac tcacctccaa ggatttgcag attaaccgga

110341 aaagatcaaa caatattctc ctgtcacaca taatcaggaa atggtgatat ttaatgcaat

110401 gtttctaaaa cctaattttt ctgtaccttt aacacatacc ctacatatcc agtggtaagt

110461 atctgatttg ttttaaatcc ttacctgaag acatgcttat tgatgtaaga gaaaggggaa

110521 aggagtgata gggggagaga gagaaacatc tcaccaatat ttctcattga tgttcggtca

110581 tcccagctgg gggccgaatc tgcaatccgg gcatttgtcc tggttgggaa ccgaacctgt

110641 gaccttttgg ttatgggatg acgctccaac caactgaacc acagcagcca gggacgtgat

110701 ttttagttta tgctttaaac ttaagaaata aactgtgtgg ttcatatgta atgccccccc

110761 cgcagatagg agactcttta cataataaat gtcccaaacc ccactaccta agaacaaagc

110821 cgtgctcagc agccatgcaa acaggtgttc agtgaagctt tgcaatatcc agggttatgt

110881 gtgtctgcag tggttggcct aggataggat acatatgccc aggaggtaag agaaagaaag

110941 ctttcagaaa ccagctgaga atgtgctgtc ctagaaggaa aatgggaatt ttatacttcc

111001 atccccctcc gtcacgccgg aaattctctt ttaggttctc acctaaaatt gtcacaaaat

111061 aattttatag aacattggaa gttttccatc tcctaccttt ctccccaacc ccaatgatct

111121 ctccaacatt tttcaattga ttcagatata ctacgatttc ttatggctcc aaaagtagtt

111181 cattgtatta tatgtgccct gttgtccatg tactttaaag tagtactttt gagaaagaca

111241 tttctgggca tatggaccac tgtatggaat ttacacatgc ccaatttggg gaaatttagt

111301 agtttaaaaa tagtcatttc tctaagctca tttgatggtt gactaacgtg ttacttttac

111361 cgtggagagg tttaattagc agagatgtaa aaccctgccc atagctttta ttcatgaaag

111421 ataaaatatc tgggcctgtt atggcccctg ctgaggggcc ctttagtcta gaggagactc

111481 ggggtctgac cctcccagcc tctgcaaggg gcatgagact cgtcgttagt cctggttatt

111541 attaactcca aatccttagc aaggtcagag gcgcatttcc tcactattga ctatcctttc

111601 ctttcctctc cctttcatag agtttacctt caacctcagg gcaggttcca tctctttctg

111661 cctgctctac tctggtcact tccactcagc aatctctccc caacaagtgg gacttatgtg

111721 cccatgtaga cttatggact gtgcacttgc tgtctcggcg ttttaagatt cttgagtaca

111781 aaagagatta tattcaacat cccttgtctt acagagtgtg gtgtgcaagc tcaaatacgt

111841 gctggactcc tatggcttca tttagctaca gagactcacc agcagctccc aggagagcga

111901 ggaagatgaa ggccttcatg gttgctcgct aggtctggat gcgcatagga tgagaagggc

111961 ttttgtccac tatttatacc tgcaggctgg tcatcgccgc catggccatg ggtgccagac

112021 acgtgatttc acaggcagct cacaaatccc agtttagaat tcagttctgt atcaaactga

112081 agataactca gcatgaaatg taaacatgaa ccactttttc caaagggttg tgggaatgaa

112141 agtgaggacc ccagctggtg ggagctgttc cctgtaatag agaagtgagt tgaaataagg

112201 tctcggaaca gggctgcagg tgtcctgatt cccaggcaaa agtcttgggc gtctggctgt

112261 tcctttgcca gctgcgaacc tttatcttgc agatgacctt gtgcgctgca cccttgacat

112321 tgaattaact ctggctccgt tgcaatcatg taacttctct tgtggcagct ctggcctcat

112381 ctacccatgt taactgtcat gaccaactga agcaacttat tcccctgaga agagaaaaat

112441 tactctgatt tgcaattaca agagatctaa acaggagagt gaaagaataa taacttacat

112501 gtatctagaa cttactatgt attaggactc ttctaaatac tctgcctgtg gttaatttag

112561 caacgctcat actgacccta ccagatactt attatcatcc ccactttaca gacgggaaca

112621 tggagataca caaaagtaga gggagcttga cgcaactcac gcaacaggtg catggtaggg

112681 gcaggatccc aagccaggaa actgggcttc agagtccgtg ctattaacca ctgggaaacc

112741 cttccccagt aagttcacca aaatctggag accacgggga cagaatgctt ggccaatgat

112801 aagacgattg aaatttgccg agtgctcaca gtgcatcaag caccgcgaac aggatctcca

112861 tacatcggtt catccagtcc tcaccagcag cttcatgact atgcctgtga tttttacctc

112921 ttccttggtg aggagggcga ggcttagaga ggttttgtgt ttttcggtga gttctatagc

112981 tacgaagggg tcaccacgac gttcaaacta gagcagcctc gtattaaggt tcgcgtgttc

113041 cactgtgaga ccataaacac aagcaaagag aacagcctgc actctatgga gttcagggac

113101 gaccccaggg agcctataga attctgagaa acccaagaca tgggttccca gagcaggaat

113161 catcataata atgaggcaca gaaggggtac ccagaatacg tgactctcag tgagagcgga

113221 cccccgctcc tacatccgca gacacacccg cacggcctac cagggcgcaa cgactgcttc

113281 agaggtagca gatcatccct ccggaagcag ttcctgtttc ttctacattt gaaatcttgc

113341 tggtatgaag aggatttacc ggaagagttt gtcagcccta ttgacatttt gggccacata

113401 atttttgttg tggggaacag ccctgcacac tgtaccatat taaacatatg tatatatata

113461 tttaattctc acctgtggac atatttattg atttgagaga gagaggaagg ggggaaagaa

113521 atagagaaac attgactgat tgccttctgt tcgtgccctt actggggatc gaatctgcaa

113581 ccttttggta catagggtga cattccaacc aactgagcca cggggccagg gccatactgt

113641 ggtgtattaa tcagctgtcc tggccttggg ttataagtag cactggcccc tcctcccagt

113701 tgtgacaacc aaacctgtgt ccagacatat tgctaaatgt cccctggggg tcaaaatcat

113761 ccccagctga gagaaggtgg gcaagggagt ttctcaggcg ctacggggaa gtgtgtctag

113821 cttcgggaca ctacaggcac tcagtaactc ctgcaagagt ggttcacaag gcagcgagct

113881 gctttttgtg ggtccctaaa aatcaggaga tactaatgag taagaggagg gttgggtgca

113941 aaaagggagc aacttcttat tgtgtttggt tccctcccat tgctgacacc cccgcggtgc

114001 agtatgggga gttgttggat tctacatcac ccctgaaact gctcctgagc ctgccggaca

114061 ttttcaccga catgagcaac accacatctt ggagcagagt tttcccagtc gctggcccgt

114121 gtgcagctat gtcctcgaat gttctctccc gtgtgtgctg ggctttatca cttggagccc

114181 tgccccttcc tccagggagg gagagagtgg ggattgccag gtaaggggag tcagctagga

114241 tcgattgcaa gtccccaagg acacaccttg aagcaaaggc acgggggcct gcctggctgc

114301 agtgggagga ttcagtcacc taaagtggca gtgaggacgt gggggaagtg ccaagtgagg

114361 ggatccaggc tccagttctt cccatgctgc tgtcatgcac ggagcacgta agctgcttct

114421 cacggtgcct gggacatagt cacactctga tacttacaca gacttcctcc ctacccagcc

114481 acgcgtctct ccttaagcaa agacattgac gttttgcgtg ttggcgaatc tttatctgat

114541 gcacattaac gggaacatat gcaatttaat aaaatagctc tttaaataaa tctggtcttt

114601 tgtgcactgt gtaagatcat gttggccctg gccactgtgg ctcagtgggt tggagccatg

114661 gtcccgcaga ccaaaaggca gcaggtttga ttcccagtct gggcacatgc ctaagctgtg

114721 ggttcgatcc ccagtcgggg catgtatgag gggcaaccga ttgatgtttc tctccccatc

114781 tttctccccc ctttcccctc tctctaaaat aaataaataa aaataaaatc acactgaaga

114841 ctgttagtga tgtgcacacc acacattagg cagatggttg gacaaaacaa gagggggagg

114901 attaaagatc tctggcagtg ggggtggggt ggtggtggtg gccctgtgca cagggggatg

114961 gagcagagcc gatcagcgtg tcaggtgaaa gcagttagtg tgctaagtga aggtgtcagg

115021 tgaaagcagt tagtgttcta agtgaaggtg actgggatga agctccctgc cgaagaggcc

115081 aggcaaaact gggtccccgc gaggagtctc tgtgtgtaca tggtttgtca ctgatgtgaa

115141 tgggtgagca ggtctaaaga agctgcccct acatgattgt gaaggagccc agaccgacac

115201 gatgcctttt ccctctcaga tactgtgggt catcctgtga agtcggaccc tgcagaactg

115261 ggctgcaagc agctcccaga ctcaaaaact atgcttgcct gtgcttgatg tgagcaaaac

115321 ttctgatctg gtgacattga tggattcccg ttgaccacca acgcggaaca tgccccatgg

115381 tgtgtgggtc tgcttccgca ggcccctccc gcagacggag ccgtcagctg cgtgtgggga

115441 aagcctgcct tctgcctgtg cggagtgtgt tggtgcctgc tcttcccttc cccacgtcaa

115501 ccacgtgggg gcccagaggc gtgggtctgt gtttagttca tctgcacttg ggccctctct

115561 ttcccacgca tgtcactcag tggccttgga gaattgcact agctttttcc taagctgcgg

115621 tctgactggg cgccagccaa tgtactgact tttgtactgg gaggagcagc tcagcagcct

115681 ctgaaggcag tttcgtgccg ttccgctcac tggagccagt tctcctcttc cccgagcttc

115741 cccagccgtc tccctggttc accctccctg ttccctgagg ccgtgctggc tcctaggggc

115801 tcccctgtgc aaaatttaaa aaatgagaat tcctctcctt gaagaaacat gtaataagaa

115861 acttattttt taaaaaaccc tgatgtaaac agggcagcaa attccagggg ggaaccccca

115921 aggtataaag gatatgctca cagaataacc tcgatgctga cctgcgtgtc gaagctgaat

115981 gtgtgccctg tgccatgcgt gagaaatcag tgactagaat gttcttcccg ggtaaagtcg

116041 aggctggaag cagacctggg tttcctcgag aacccgctca agggtggccc atgtctggga

116101 gccgtttcta tgctggctgc atgtgacgtt ctgggggggt tggataggaa acagtttagg

116161 ctgtgcaggc agcctggccc ctgtcgcagt tactctgctg ttgaaacagg agagcgaccg

116221 cgtgtaaaac gtcatttgct gaaatgggcc gggggccaga cttgtctctg ggccccagct

116281 tggaggccct gctctggaag ctggagagga aactctttca gtgccccgta cccagacttg

116341 aagctgcagg gtggctccgt ttcaataacc gggcctggct tgaaatggaa ttactaatgt

116401 taaaacaaat atctccccgt tgcttttact tccaatcatg ttttggggga aaggaagacg

116461 agaggggggt tgggggtttg gggctggaac cctgaggtga tggagaacag ggcgggaaaa

116521 ggagagatga cggacagaac atggtaccga agtatgagca tcctgaaatg gaagtggaga

116581 catttgggac atgatttttt aaaaatatat tttatttatt tatttctgga gagaagggaa

116641 gggagggaga aagagaagga gagaaacatg agtgtatggt tgcctctctt gtgcccccta

116701 ctggggacta ggcctacaac ccaggcatgt gccctgactg ggatttgaac tagcgaccct

116761 ttgcttcaca gtcaggcgct ccatccacgg agccacccca gccagggctg ggacacgatt

116821 ttatgtgctg gtatgaacag aatgctaact aaggaattgc agggctcctg tctagctaca

116881 gcagtaagcc tcatcctgac agacagactt cattcggaga cgggaggggg aggggctaat

116941 tacaaccctc tgactgcatg tccttccctc taagtcccct acggccctcc tgtctccacg

117001 gagggagaac caggtcttga cactcggttt ccctttgacc acaaggagga gctgtgactc

117061 tactgggggg cagggagcag atgagaggac ccccccatgg aagagggtta aggaaaagtc

117121 tggttttgcc tgcaacctgg gagaatgtgg ttggcagagg ctgggagtca gcgccgcaat

117181 gcgaggggtt aaatgcagag tcttccgctt ggtgtggacc tgcaggtgtt cttgtcccag

117241 cacagagccc gcactgccag accagctgct gtgggcctgc atgggagcag ggagaagggg

117301 gggagtggag agtggagcct gcaggcaggt gcaggcaggg gctttcctga gacaacctga

117361 aggctgtggg gctgggaggg gagctcctgg agcttaggca gggagcctga caggccacac

117421 gtttgtgggg gaccttgcag agacgacact aactagggac atggaactac caggctaagg

117481 gaaaccgggg cccctctccc ctcactccac agtatcaggg agttggctag gaagggtttc

117541 attctcgtcc ctctgctggg tggtcttctg agctctcgga gcaggagggt cctgggcagt

117601 gacaggaaac ccttctgggt ttgataaaac ggttctgtgc aaataccagc tacagacgtc

117661 cttctgggtg ctgtctccca cacagtgact cacgatggct gcattgtcca ctcccggaaa

117721 cacttgatac cgaagcaccc agagacagca aagacagaca cccgaaaccc cgatctgaga

117781 ccaggcttct cactgttttc actccaggct cctgttttgt tttgttttgt tttaacctct

117841 ggaatttttc ttgcttcttt ttttaaaaag atttttaaaa attattttta gagagggaag

117901 ggaggcagag agagagagag agagaaacat caatgtgcgg ttgctggggg tcatggcctg

117961 caacccaggc atgtaccctg gctggggatt gaacctgtga cactttggtt tgcagcccac

118021 actcaatcca ctgagctatg ccagccaggg cttcgttgct tcttaaaaat cccagaagtc

118081 cacaaaatta cgtttgatgt caactctagc catacgcagc aggaggaagc ttcagcatac

118141 gtacgggttc cacccggctg cctgacgcga tgaagtctgg ctggccgggc acttccaccc

118201 tcagccctgg gaaaaccgct agcccttcgg cgaagggtaa ttttgcagtt caccttagaa

118261 tgcgacagaa gaacctgcga aaggcagcca actcctttgc ctacatgtaa atctaaattt

118321 cttagctgtc tgtgttatga gtcactttca aaatcggttg gaagttataa acgtcagaaa

118381 atgcagaaac acacacttca tacttctaaa ctcaatattg aatgttcttt gagactaact

118441 tgaacgtaag gcttgggtag tcagagtccc cctggatctt cctcacgcca caaaacgcct

118501 acagcttctt ccaaatttag aaaaaaaatc aggtgacatg taattgtgtt tgaaagttaa

118561 taaagcctat gcctcatagt attacattta atttttgtta attgttgctc atgctacatt

118621 caaataactt caagattaaa cactgacagt ccaatagaaa tcgtcagcat acatatttta

118681 agtggctaaa cgagacttca gtagctaaac agggcgacag cgtgaactga gggcaacagc

118741 gtgaactgag ggtttatgga ggtgaatgac ccggggcagc agcctgtccc atctggtttg

118801 gatctggatt ttctttggga aaaccaacgg ccacgggccc gcctgcaggc atgtgacttc

118861 aagagtcgat gtgtatgtgg acttttgtat ttttatcaga caaaaagggg atctctttca

118921 gggccagagc cagcaggaac cagccgccag ctgagcagga cggaagaggg gtcgtggtcc

118981 tagttttaga aactggttct gaccagcctc gagagagagt ctgatgaaag tggcacctcc

119041 ccgggactct gtgcccaggg agtaactcag tgtgacagaa ctgggcacag aggttccctc

119101 cccgtgcccg ccccccggag cacaccgcct ggcttccggc tgggttgtcg ggagctgggg

119161 ggaaaggggc ggcttcgccc agcggagccg tgcccttgtc gaagtcacac acggcagcag

119221 caaggatgcg ggtgttgctt ctagaacaga aagcagattt gacttttaaa caaaccaaca

119281 cacaaccctt ttcccgggaa cacccacacc tcggggaagt gtccgaacac gagcagcggt

119341 gtgactaccg gacagcccca agcaggaccg ccgtccaaat gtgcatcctg ctctcggcca

119401 gccccctgcc cctctgtccc agctcttcct gagcctcggc ctcgtcccac cggtccccac

119461 ctccccacct cctcgtggat ccagttatct tcacgctaat tttcctgcag ttcaccctct

119521 cctttacttt gttgctgtga ccgaaccccg gcaccgccca ggcaccccag tgccagcgtg

119581 cattccccct gtggaggctt ccccttctcc ggctcagcaa cgaggggtca gaggacccgt

119641 ttccttactc cgtcctatcc tttgggcaat tctcgacagg cgacaaaaaa gttgtccttt

119701 tctacctctc ttgcaacaaa cgtcaaacag ccggaactaa ccttacgggc atctcgttgg

119761 atgaggcgca gctgattttt caagagctct tgcaaaaaaa acgctgaagt ataattctcc

119821 cggaatctac ttgttaacca tggttacact tcataccttc acagtcttca catatcttca

119881 aagtattaca cctagcctcc tgtcagtttg tttgcttttt tttttttttt cactatttgc

119941 acctgattgg tttactcctg tatctccaga ccttgttctg tgttcagata aagaaaatgt

120001 gaaccactgc gggctctttg aaggagtgcc caccaggagt tgacagtgtt gccgaattgt

120061 gtgtgtaaga tgcctcccct gcaggtggca gacctggttc ccagcctcac ccttccttgg

120121 tccttgctga cttgggatta ttaagggttg gagcctggcc cagtgggccc tgagagtgag

120181 aactgtgctt tgctgaccac caggaggcgc tgtggcccca ccaaaacccc tgaggttggt

120241 caaggacaga aggaaggaat gaagagaatg ctctttctcc aggaaattgg tttggcagca

120301 tctggagaca gttttggttg gcataagtgg gggtgggcag tgagcgccac tggcatgtag

120361 tggacagaga ccggggatcc tactaaatat cccatttgtc caggacaaca gccccacaag

120421 gaggagttat cctgagccgc atgccagtac tgcggaggct gaaaaaccgc agggcttcgg

120481 ttcacccccc ctcgaggacc cgtgaacagg gtctgctcct aaccaccatc agttcaggcc

120541 atttcaaatg gtctctgcca gttctcaggg gacacctcgg ggccactctc tgcccacagc

120601 cgaaacaacg tgtgagcgta aatcagacgt tgcagggagc caagatgtga gtggggtcgc

120661 cctgcattac tctgaaggag accaggctgc atgcaggagg ggcggggagg gaggaggtgg

120721 ccaggtgccg gggcaaggac aggggaggga ggcagctgct ggcacaggga tgacgtcagg

120781 tgaacgtcct tgagacaacg tgtccgcccc cctcagctac ccctagaaat cccaaagtcc

120841 tttgaaagca gtacaggttg aagctccttt gaaaggagca agggcaggac gagaagaaag

120901 gagagaaccc acgtgaggag agcctgagga tggggaaatc gttctagact gtgacatcac

120961 agcgaagcct gagctgggac agtggggcgc tgcagccggg gcttcttacc accggaggca

121021 agacccttcg ctagggccct gcccgtgggc cctgcccgag ggccctgaac ccaccatggg

121081 ccccaggttc ctctgctgtg tggccctctg cctcctggga gcaggtgagt gtggagtcag

121141 gtgacaggac gcactcggag tctctgacac gctcacaaga acggcaacag gctgcatcct

121201 gggtcttgtc tgatttctgc ctcttttctt ctcaggcccc ttggacgcag cagtttttca

121261 ggctccaaaa taccttgttg cacgggtggg ggatacaaag tcactaagat gtgaacagaa

121321 gctgggccac gatgctatgt actggtataa gcaagactcc aagcagtcgc tgaaggttat

121381 gtttgcctac aataacaagg agccgtttct aaatgagacg gcttcaggtc gcttcttacc

121441 agaatctcct gacaaagctc atttgaaact tcacatcaag tccctggagc tcagcgactc

121501 tgccgtgtat ctctgtgcca gcagccgaga cacagccctg caaagtcatc gcctccctct

121561 acacaaaccc accggcccag ccaggaagcg gtggggacaa caggttcacc cagctagagg

121621 gaggctccct gcacccgtct gtgacgcccc tgcctggagt tgtatctacg gcacagccca

121681 taatacccaa cagggtggtt ctacagttca tgcacccaga agtgttacag aattccaccc

121741 accagctaca gcaggggttt ctaacaagtt cactgaagca ctcaggactc cactcacggg

121801 aaagggccag gcactgaaga tcttaactgg tggaaggagc cattcctttg ctttctctgc

121861 tgtacgcatc ggtgcgtgtt acacacaaca atattactga aaacacacta tgtgttaaac

121921 atgtttgatg agtgaaagcc aaaaggggac gtgcaggtaa acgtttctaa gaccaaaagg

121981 aaatacgggt tatcagtgag ttccctgctt gatagaaaaa tacttggaga agcatgaaag

122041 tccatggcaa tagatgagct agaaagaaag ggggagggtg atgcgggccc cggctcttgg

122101 ggtccaagac attgtggatg gggagagaca ttcacacgga ccaccgagtc ctgtggaaga

122161 aaagggcaag cagtttcact ctccagggga gcaaaaagcc atggccttgg ccatgacagg

122221 cctttatttc tttttctccg cacattacgg tgatggtcct cattcactgt gcacaggttg

122281 gctctaggtg gttacctttt ccagagaaca aaggagccaa tgacgctaat cacatcacag

122341 aagagggata tttgcaaatg aaaagggaaa cgtggttgaa ccggttacac tggtcctggg

122401 aaggtttagc atagatttta ggaagttaca ggaagcactt gccatctcaa ttcagggtga

122461 gggagttttt agcaaaaagc aagactcaaa gcggcctagg tatattgcag gcctgattcc

122521 ccacgggaga acctatccgt gggtgtgggt cctgtgcatc tccgagtggg agctgggccc

122581 acgccacgca gaatggtctc ctacaggagg gcgaggagcc gacagaggta gaggtagcct

122641 tgtgcagtgc agactctcac gctcaggagc tgagcaggga tccctcccta ttctgactct

122701 catctcagag cctcctgcaa agaaggcagc agcaacatga tggaggacat tatgagacgt

122761 ggaaaagaaa gactttggac acaagtctct agacagtagt agctagtcct tgatcctacc

122821 tgacgtgaga tgacatgatc agatttgtgt tgacctacca tcacagtggc agacgtatag

122881 atttgggatg gaatgaacaa tgaggagact actaaaatat tctgcatgaa ggattataaa

122941 gggatttatg gggtgcatat gtggataacc taatccagaa tagggactct ggattatgtt

123001 atgttcttct aaagagtgtt aagtgttatc ctggcaggtt tgaatgtgga tttatttcag

123061 tttgtccctt tctcttaggg agcctgagca tgtcctcatt ctaagtgcct ggacttgtgg

123121 ggtcttggag gaaagcacca catccatgtg agagtcttct gaaatctgtc tccagctctc

123181 cagccttcca ccacctgtct ctgctcagcc tcctagagcc ctcactgtcc ataccccata

123241 ggaactggtc acgaaaaagg gagttccttc catgcatcct tggggattcc tttttgttcc

123301 ctatactcca aaccctagct gtcttggaat tgaagcagga gacaccatag gaagaactct

123361 cagactgcaa tactgtcact ggccagcact tagcaccctg gtggccagga aatgcctttt

123421 accaattagg tagaataaag agaaaccgag gcagggagga ggaaggagac aagtctgaca

123481 tgctaactca gcagtcctaa gcctgttctg ataatattgc caggtgttcc aatatcacaa

123541 gaaacactat gttagcaaga aagaaagcct tgaaacggtg ctttcatccc gaggcctcaa

123601 tgtgggagag ggtcccggga cccaaagaaa aagcccacaa ataactttgg ttaattgcct

123661 acctctggtc actatctgtt ttccgaggga gtccctgatg cttacagaaa gagcccataa

123721 ataactttgg aaagtgcccc aattttaatt aactgcctcc tgtcatgtat ttgttttaca

123781 acaggatgac caaatggggt ctggagcaag ataaggattc gggcgggcag acccccattc

123841 atccctaaat ttgggtagtc acattccccc aggaaagctg gcgccacatt tacccgctaa

123901 tcaatgtgta acttttttcc ctcctctccc accaactatg caagtcctga gaacaaagga

123961 cttgctctct ctctgtcggg ggctcagggg gctcctggct ctggggccac agggcctctg

124021 gccacccggc ctcaggctgc ccaggggctt gccgccctgg tctccagccg ctcttgcttt

124081 tctcaccctg ttttgctctg aaactctgcc tttcaccccc tactctaccc agtcctgtgc

124141 tcttccatgg aagcggacca ctaataaact gattacacac tactagattt gctgatatgt

124201 aatcctttac atgcattagc aagaagaacc aggtctctcc cgtcaacaga atcagttacg

124261 tttttccaat ttatcttcaa acaacataat gccattgcta ttttatatca acatctctac

124321 ttgaatgtac ttgggtgttt actattcatt ttttcactct tccttcttgc aatttggacc

124381 accctccagt tattttcctt cttcaggctc attccctgat cggatgactt taactctcct

124441 ttcacccacc ccttgcttcg agagtcactc acgttggctc ttgtttcaga gccaccaagc

124501 ctttcagttt ccctcatcct tcaaggtgtc agttaagttc tgattgtttt gtaatccttg

124561 gaatttactt ccttatcggg agccaagcaa tgtgatgcaa cttcatgtgt tcagacttgt

124621 gctgtttcac atagaggggc catcagagtg tccccttgct acgggggatg cgagtatcat

124681 agcagtgttc atagagatta tgctggccct gcaaccaggg ctagcttcag gttcttacct

124741 gagaatgcag tatctcccac tccaggggac tgagggtcca ccatacctgc cacgtgttca

124801 gggagaatac ccaccctcct ctctaactgc ttacattcta accgtgtgcc ttaacattta

124861 gtgtctcttt tggaccatat ctaaaatctg aaaaaattta ttaccataga aaacactaat

124921 atctacctcc cgacccagca cgctgactta aagcatgggt atccatcttc tttgccccca

124981 acatggattg atataaatcc ttgttattcc aatttggcga taaatactaa ccagaataat

125041 aaatggtatg ttaaaagatc atttagcagt ttatagagcg tttcattttt gtatttgttc

125101 atccatttac attttctgaa atgcaaattc cattcgggcc aaatggcacc tgtgaacaaa

125161 tcggatggga ctccaggaac taggcagata aaaagcatca acaggccgtg aagtagcaat

125221 ggctcctggt aggtcatccc cactagtgcc agcctgcaga gccgtctgca gaaatgacgg

125281 gcctggctcc gtctctccac catgggctgc agttgctggc tgcttttgtt attccggatg

125341 cctctttcct cttcacactg ccctaggctt gcacacccat cacagctgct cccagttcct

125401 ggagtgaggg gtgggtctgc attgctgccc tgggctgggg tggaagaggc ctgagatttg

125461 aaaagcatgc tgctttagcc agaaggagga gctgtggctc cactgaaact tctgagctgg

125521 gggaggggaa ggggttggga gtgagggagg ggtgtgagag ggcggacact aggacctggg

125581 gctccatcct cctgtcagct ggggccatcc gttgttgaca tcccaaggat attaaactga

125641 aattgtcttc aaattgatct ccctttgtct tcaaccagtg ctttccattg tctttcaata

125701 acggtcagtg ctgagcccag ccaagccctg tctcctcaat gcaggtgtgg agaccggttg

125761 tccctccttc cctcagcacc cttccccgtg tctggctctg ggctgggact gcagtcatgc

125821 atttaaactg atgggcgcgg gtccatcgaa tctgcccccc tccactcagt cgggcaaggg

125881 ggctcccagg aggtagacac cgagggaaac gacattggag agacatccta cctgactaga

125941 gggtctgtgg ctgagagggg gacttggaag ggagtgtgtt agggagagaa tctatgtact

126001 ggggaaagca gctaaaagaa acagggcaaa catcagggga ataacccacg tcagtccatt

126061 gagatccacc ttgggtcaga gtctgtacaa agcatgagag ggggaggagt gtcccttggg

126121 cctggggaaa ctgtcaggga cagtgacatc acaggcagag ccgccaacca aggccaagga

126181 gaccagagcc caccccccac ccccatggca tcccagttag aagaggccat tgggcctgac

126241 tccaccatgg gctgctggct gctgggctgt gtggccctct gtctcctggg agcaggtgag

126301 ttggggctcc aagggctgcc ctcgggccct cagccattcc cctgtggctg cagcaccacc

126361 cttcctgctg ggcttggtct gaattctgtc cctttccccc aacagtctgc ccagacactg

126421 gaattactca ggtaccaaag ttcctggtga tgggaatgag agataagaag tctttgaagt

126481 gcgaacaaca gctgggacat aacgctatgt actggtacaa acagagccct cacaagccgc

126541 tgcagctcct gtttgtctgt agctacaagg aactcactga aaacaatact gtcccagaac

126601 gcttcacgcc caaacgccca gacaccgctc agttacacct gctcgtggat gccctggagc

126661 cagaagactc ggccctgtac ctctgtgcca gcagcaaaga cacagccctg cagggccacc

126721 gcctccctgt gcagaaacct cccggtccag ccaggaagct ggggggcaac caggcttggg

126781 ggcgcacttc ctgcgagccc cgagacagca accccagcgc agaatcctgc ccatcccttc

126841 actggggtgg tgatgctccg tgcccacaca ggcagcagcc tgtggcctgg cctcgtcttg

126901 aagaagactg ttcccggaca gaaaagttca agctcacgtt cttcagtttc ttcctatcag

126961 cttgtgaccc cgggaagtct ttcatgagac acgttacagt ctggccccag cagactcctt

127021 ctggtaggca ccttcccctg acttctcttt ccatccatgt gagacccggg gcgcagccct

127081 gatccacctc taaatatact gtttccagaa cccctcattc cctcacccct tggctttgtc

127141 agttcccaac cctgaggaca atagctctct cattcacctg ggttatttct ttccccttga

127201 ctagaactat gagtcacaga gtccatccct ctggaccctg gcgctgaact ggctgtgaga

127261 gggacatggt gtcccacctg tgtgcagatc tctaggcctg gccctgccgt cctgaaggtc

127321 ccatcctctg agggcccttt gtagccgcag tatttcactc agggctgtgg gcctatgcac

127381 tgcaggacac caggctgagc cctgggcatc acgtgtctac actgacacta aggctcaaag

127441 tcctggtcct taccctagaa cgccacagcg ctgagccggg tggacaccct ccctccctgc

127501 cctgccatgg gcaccagctt ctctgctttg tggccctggg agctggtgag tcagaaacaa

127561 gaggaaactt tttttttaaa gattctattt atttattttt agagagggaa gggagggaga

127621 aagagagaga gaaaaacatc aatgtgcggt tgctgggggc cgtggcctgc aacccaggca

127681 tgtgccctga ctgggaatcg aacctgcgac acttggttcg cagtccgcgc ttaatccact

127741 gagctacgcc agccaaggac aaggaaactt ttgaggtgag cttttcattg ccaggtcttc

127801 agtatttctt taatctggac aatggctggc ttccttatct ccagctgagc ttccatatca

127861 tcctaaacag aactcatgga tactcaggtc aaacaggggg caagatctct gttgtggaga

127921 taaaaaaaga aagttgatct tagaatattc tcagatagga accattgtgc aatgtagtgg

127981 catcaacaca acccagcccg aggactgagg ctgacccaca agccagtgct gaatttatgg

128041 tcagaggaca tgtacgggaa gggtacatgg ctcaaacaaa tacaaagtgg gcattagcga

128101 agcccctcta atggattctg agcaagctag acaggacccc tcaccctgga ctggggtgtg

128161 cgccctgcac tgtcctgcag tgacggaggt ttgggcaaca gtcaccctgt ccatgacagg

128221 ctgctctcag ggccaatgcc agctccaggg tcttactgca tgatccggtc cgaagtgagt

128281 cctcatcaga gtctgttttc ccaaatctct gtgttgtccc gtttattcat tcggactggc

128341 ctcttccccg cacttactag tgtggaggat tagaatatgt tatttcctac ttgggcatca

128401 gagaaacact gagagagact ttatcatgta cacgtgtgca ctcccatgca ggagactgga

128461 attgtactat tgccaatata tggaggggaa aataaaggat ttctaaaaat gagaggtggg

128521 aagagaaatg tttcagcagt gtggggaaaa atgagtttta ttattactta agctttgggg

128581 taagctgatc agtaggcatc ttgcaaagag gcctgtctaa attatctgcc ttgagtcaac

128641 tgtgaaggcc atcaaagtta aaaccaattt tgttaattat taatcctcaa aaagtctttg

128701 ggagtccatc cccagtccat ctccacaacc accatgcttt ttgttacaac acaatttcca

128761 aattcagcca gaactgagtt ttttgtacct ttagttttta aaagtacagt gcgatcagca

128821 aatggtaaag ttattcctaa tttaaacaca tctcaggagt cttgtcctct tgcatcaggg

128881 ccaaagctaa atcctggttt ctgccccaac acgccacacg ggggcgctgt ggaactgctg

128941 agctccagaa gttcctgggg cagagcaggg gtcagagcta gctgcctggg atggcaaggg

129001 ttaagggaca gccctgatgg tgcgtctatc ctctacaagg tcaaatagca caagcactaa

129061 cttgcaggtg ctgcctcagg gggagccagg aagttcccct tgcagcggtt aggggggccc

129121 tctgagccca caaggggagg tgtatggtgt ggcgctggga gggggcagcc tgcaggccct

129181 gggagcaggc acggtggagg aacaggtgtc tgagggagaa gtaaaggagc tgtgtggact

129241 ggcatgctgc ctgaaccaga cacagcccct tggtccccac ccagcccctc gggtcagaga

129301 tgagcttttc caggacaaag caggggcctg agtcagctga gccagagtgc tggcagggga

129361 cccaggcaca tggcaaccag cagaggtaat gacatcagag caagatgtca ctgcctaatg

129421 cccaatcgga gaaggtgcca gtacatcagc tccacaggcc cagagccctg agcagggagg

129481 cgtctgccca gtcctgcccg gtcctgccat gggctccagg ctgctctgcg gggtggccct

129541 tcttctcctg ggaccaggta tgccctcgac acagctggga cgtctctgtt ccaagcctga

129601 cagcacctga atccattcac agtttccatc agggcctggg ccctgttctt tctccatctc

129661 ccgtctccct ccctcccagg catggtggat tctggagtca cgcaaactcc aagatacctg

129721 gtgaaaccca gaggacagac ggtgacactg agatgttccc cgatctctgg acacaccgcc

129781 atctattggt atcaactggc cccggggcca gggtcccaag ttcttcattc agtattacag

129841 agggacagca ggagccaaag gagacatgcc taaccgattc tcggggaaac agttcgataa

129901 caacagctct gagctcaccg cgagctctct ggagctgaag gactcggccc tgtatctctg

129961 cgccagcggc tcagcacagc cctgcaaagt cccctgggtt ctgtgcacaa acctttccgg

130021 cacagcgtgg agccacctca gagctgacag gcctgggaga acctgggaac cgctgggggc

130081 tcggggggtg ggggggactc gggcactggg agtgctgcct caggctttct ggggcaacag

130141 aggacaagaa cttctgctca ggtgctacat caacacgctc acgtcaacct caccttcctt

130201 gcctggcact ctgcggttct cgggcagagg acaggttggt catttaatag ctgcccactc

130261 ttttttttcg ggggaaagtt tattggagga tcgaaattgt tggttaaacg tgcagtctag

130321 ccgatagaac ccctggaatt tggtttataa attcatggaa aagaatttca ggttctgcag

130381 aaagtctagg aaataaaata tggcaaaaat gctttgaaat aaagatagag ctaaaagatt

130441 gttttaaatc tgttatttgt atcatatcat tttctctacc caccccccca aaaccttcaa

130501 tgcccagaaa agaaagctgt gttcagtcac agacccttgg catccttgca gctcacagat

130561 tgtctatgca cgtgtgtgtg tgtgtgtgtg tgtgtgtcct tgtggagaca caccgagtgg

130621 cagtattttc tctccatggg catttgttta aggggtggca tcactgagca gggctcaggg

130681 acactctggg taaggacagc atgtgttggc cccactgggt ctcacctagc tagagctcac

130741 agggcggtgt atgcaaggta tatggtctca gaaatgaata gctaaaatat ttaaactgtg

130801 tttgtaaaaa cacactttgt gccctggctg gtgtggctca gtggattgag tgcccgcctg

130861 cgaagtaaag ggtcgctggt tcaattccca gtcaaggcac atgcctaggt tgcaggccag

130921 gtccccagta gggggcgtgc cagaggcaac cacatattga tgtttttctc cctctctttc

130981 tccctccctc ctcctctctc taaaaataaa tagtttaaaa agtgcacttt aatgggtgct

131041 aataattatt ctaaatacat atttggtaaa gggggaaaga taaatatggc ctaaggagaa

131101 accagagtat ctaactacat atttggaagt tcatggatgt gattactagg aggagcgatg

131161 gaatagagca tttccaggca agatccactt gggatccgca gactatttat tgttcgggcc

131221 cgtataattc tggctgtctc ttctgttatt ggacattgac tgccatggtg caaaataatt

131281 ttttcctaat tgaaatgttg gttcatacaa tcttggcttc aaaatgaaga gtgtatgtga

131341 ggatcaactt tgtgtcagaa aaggctgggg aaatagtaga aatggccagt gggaggggaa

131401 ggggggtgag ggctgtagtg acaagggccg gggcaaatgc tggctcttaa tgtagataga

131461 tgtgaatccc ggagatgtgg actgttgccc ctgggaggtg ctgtggaccc acagggctgc

131521 cagcaccact gggaggggct aagggagagt cccaggagag gacagttaag ggacctctga

131581 cccatgtgtc tgcaggggaa gccgcgtgag tcactgaggg gtcctgagac gcatggagct

131641 ggcacaggct cctcctggcc aggaagaagg ggcgtgcctg catctcagga ggcaccaagg

131701 aggtgacgcc ctgagtctgc aggggaaggc gggatgtggg ggttggggtg gcaaaggctg

131761 ggtagatagt gtcaggtggg cagggacaat actgtgggaa ggtggggaca gtgctgaagc

131821 tgcagccaac ccttagttgc ggggatgtac aggacaggga gtgatgtata gaatcccaac

131881 cctgggtcac gccaaaggtt cagagcacca gggggcagcc tccttggaga gtcccatgag

131941 agcagctacc acatcacacc aatgatgtca tagcgaatgc tcctatcagc acacagggag

132001 cacagaactg tagaccgctc accccagggg accccgccca gagctgggag aggtcctggg

132061 cagctgccat gggccccagg ctcctctgct gtgtggccct ttgtctcctg ggagcaggtg

132121 agccccgggc actgtgtgta agtctggaca cacagtcata tccctgacac acaagcctgg

132181 gggtgatggc tgcctcggga ggctccctgt gctctgcccc cagctcctct gtgtctcctc

132241 ccaacaggcc cagtggactc tggagtcacg cagaccccaa aacacctgat caaagcaaaa

132301 aaacagcaag cgacgctggg gtgttcctac atctctgggc accgctacat gttttggtac

132361 caacaggcct ggggccaggg acccgtgttc ctcgttgagt actacaatgg gcaagagcga

132421 gaaaaaggga aattgccaga tcgattctca gtgaagcatt tcagtgacta ccgctcggag

132481 ctgaccgcga gctcgctgga gctgagtgac tccggcctgt atctctgtgc cagctactca

132541 gcacagccct gcaggcgcgg cagcctcctg cacagaaaca ctccgcccca ctcaggaagc

132601 ggcagctgcc agccagacag gctagggtct ttcccaggca cggttctgct gcttacagat

132661 ggggtggttg tagtgctcac agacatcgtg cagcttcagt atccttgttc ctgtttgtat

132721 acatgatgag aaataatgtt cctgggtctc agatttcata acccgaggag caagcaatca

132781 ctcaagtctg ctcctcaaca cctgtggatt cttcccctcc acctctccca gccccatgcc

132841 cacactgact ccccttttct gggtttatat gagcgggcct ggggagatca cgcaccatct

132901 gggattgtcg accttttccc ctacagcttc aaggaacatt tcacttcaag gcagcgaata

132961 tccatccatc cctcatgatg acagcaaaga aaggcacgat ttcttttctt ttcttttttt

133021 taaagatttt atttatttat ttttagaggg gaggagagtg agaaagagaa ggagagaaac

133081 accattgtgt agttgcctct tgtgcgcccc ctactggaga cctcacctgc aacccaggca

133141 tgtgccctac actgggaatt gaaccagcga ctgagcaagg cacaatttcc tgcactgttg

133201 ctgtcacctg ggcctgcaca gagccctgaa ggaaattagg gttcctgcaa agcatgggac

133261 tgtgggtaga taagctacag gtgtttaaag actcgttata tttattataa ttaatgtcag

133321 ctgctcccgc ccagccaccc acagacccag aggggccctg agtcagcccg gctgctgcca

133381 tgagcggcgg cctcctgtgc tgcgtggcct tgtgcctcct ccgtgcaggt gggtcctggg

133441 ctgggcccct ctgtggggac agtgcccagg cccagcaccg agccgctccc tggaggctgc

133501 agcatctgtt cccctgtcct ccccgcaggt ctggggatgg ccggcgtcac tcagtccccg

133561 acattccagg ttgtgacaac aggacagacg gtgaccttgc ggtgttccca ggacttgaac

133621 cataacaaca tgtactggta ccgacaggac ctgggtcacg ggctgaggct gatccattac

133681 tcagtgggtg ttggggtcaa ggacaaagga gaggttttgg atgggtacag cgtctctaga

133741 ccaaaacgag aagacttccc cctcacgctg gaggcggcca cccccgccca gacgtccgag

133801 tccgtctgcg ccagcagcga ggcagtgagc gcacagcgct gcacagccgc ctcctctctg

133861 cacagaaagg ccgggggggg ggggggccca caccctgact caggggaccc ttgtgcaggt

133921 tcctacacct tagagccccc agaggccccc gcctgcaggg ggacccccgg agtgtgctgg

133981 ggtccacctc cagggcagtg tctgccaagc ctggtctcga tccatggcct gggtcacaca

134041 tgtctgcact gtggctgtct gtcagtctgc gctctgcagc ggctccgtta gctgggaagg

134101 tgcttccgta gctccagctt tcttgtcttc cgcctgagtc tgatgccccc aaggtgggaa

134161 gtcatcagcc aatcagatgc acctagaccg ccctgcatac cacacacagg tgtgtcatgg

134221 tcagggaaac tgaggcttct tccacgaagc agctgtacga acccagtttt taagtgcatg

134281 gtgacgtaca aggacgaaag gctttactgc gggctgtgtg gacacaatac tgcatggaag

134341 tcagtgagca cagacaggac cctcagtcat gacctgcggg aggagtagga gcttcctact

134401 cgggtgttcc cagggcatcg ctccgtctgt gtgtctctga gtgtgtcagt gtgtccaggg

134461 cacagccgag ttgaccggac ccccgccctc acctaagaaa tggggaggac tgagcatgtc

134521 tgactgggac agtggagggt aaggtgacag aacagtgtgc tccaaggaga tcccgaattg

134581 cagagaagac aatgaaatga cccttctctg tattatattg atgacaccgt tgacatgtcc

134641 tagtgaaaat gccggctgac cagggtgaca ggtgagtgag atgccatgtg agaaccctgc

134701 aactagacag cgggggaccc catagtgaca gtcgtgcatg gattttgttc aaaaaggagc

134761 ttattaatgt ttgagccagt caatcaagtg tgctctcata tacaccacgt gcccctgagg

134821 tcgagagaac tgacagagac agtgatgtca ctgtggcagc cgccctgtgg ggacaaggga

134881 tgtccctcct cctctgctcc tgctcacagg aacctgaccc ggggaacctc ccactcctga

134941 cctgaccctg ccatgggcac caggctcctc tgctgggcga ccctctgtct cctgggggcc

135001 ggtgagtcct cagaaaacca ggcagcttca gtgtgtgtga gtctgatgat tataatagtt

135061 ttcctccttc tgttgtcaac ttctgtgttc caggtcacac agaggctgga gtctcccagt

135121 cccccagtca catagtcacc aagaggggac agaatgtgac atttcggtgt gatcctattg

135181 ctggccacat tagcctatac tggtaccaac acgtgccagg gcagggcccg aagttcctgg

135241 tgtactttca aaacaaggaa cctctggact cctccgggat gtctaacgat cgcttctctg

135301 ctgtgagacc tgacagctcc tactccactc tgagcatcca gcctgcagag cctggggact

135361 cggccgtgta cctctgtgcc agcactccga ccacagtgtg tcaccgtcac cgcctccctg

135421 ctcacaaacc ctcctgtgct cccctctgct catggttccc aggcttcctt gcaagagagg

135481 tgtcaacagc gctgctgcac aagtggccac ttgagggcgc tgtggatcca tggagtgaga

135541 tgcagatggt gccctgtggg agagggaggg gggaggggct tggttcaccc cagagacttg

135601 gcttggattc tgctttaggg aaattatcgg atgaatcaaa ggtttgctga tcatatcttt

135661 tcacatcaat ggatatgcat ttgtctgtca cagcagggag ccatgacctt ggtcttgcat

135721 ggagttatgt cctctcttga agcccatgtt tgtcccagag tgtgatgggg ctccagcgag

135781 aagcctggaa aacacaagcc aggtgtaggg agaggagcac ctgctccgaa gccatggggc

135841 tgctcaccag ccctgtaccc tggggcaccc agctcagtac agccagctgc acctctcaga

135901 ggaaggtggg cagaatgggg gaatgctgtg ccccaagatt gagcttgggg gtgcagggtg

135961 tttgggacct acacagacag tgacataaga cagtgacgtg ctctgcaaac agagcaacgg

136021 gagggcaaaa cctctgctct ctcatcccaa agagcagatc cccgagaggg gcgtggccag

136081 tcttgtgctg ccggaatcct ccaggggctc ttggctcctc tgctgcgtgg acctgtgtct

136141 gctgggtgca ggtgagtctc agctcaggtg ggacattgct ccaacccaac atccttgact

136201 gttgctggag tgtccatctg cctcctgggc tggtctcagc tccatctcct tcctccacag

136261 ggtcagtggc tgctggagtc acccagtccc caagacacct catcaaaggc cgtggtgggg

136321 aggctgttct gaaatgccac cccatctctg gacacagccg tgtgtcctgg taccagcagg

136381 cttgggggca ggaacccggg tccttcattc agtattagga acagcaggag tccgggaaag

136441 gaaacatctc tgaacgcttc aaaggcaaaa tgttcagtga ctaccgctct gagctggcta

136501 tgtgcacctt gcagctggag gacttggcct tgacctctgg gcctgcagct tagacacagc

136561 cctgcagggt ccatggcttt ctgttctagc tcaggatgtt tcaagcatag ggaatggcct

136621 tggccgagtt tcacagatgc ttctagctgt ttcctatcac tcgttttgcc ctgctgagct

136681 cagcgagtgc cttcctgaat gttctctcag ctctgccagc tccgtgcaat gcccagcaca

136741 tggcaggaag ccctcctctg tgacagagtg gagtccacac tgtgtgttgt ggagcccggt

136801 cctggtgaac accgtgcgtt cactttacag aggcgggagg gctcagtgca ccatttccta

136861 gacagggagc tgcagctcct ggacgttcca agtgtcctat gcacgtccat gctccatctc

136921 cacgcctcca aagtcaacaa gcccctggat tccatggtcc tgcctgggcc ttggtccatg

136981 agatagaatg gagcacagtc tctcccgttt caagagcatc tgagctggga tgggaggccg

137041 caccttagat ttgggtacac ggctgattgt ctggcaactg ctgggacata gagcctcacc

137101 ctagaggatg tatgtctgtc ctgtcatagt tgcagacaga gcgaggccat gatatccccc

137161 taagagagga gctaagtgac tggggaattt ccaggccatc aggagattct aggagggaca

137221 cagaagccgt ctaggagtgt gaagcagcat tctgggagct catgcaggga atgggtgttt

137281 ctgaaccact gccaggggta gaggaggatg agaggtctca gcctgtcctc agctccagca

137341 cttctggtca ggaaatctga aactgctgca tctgcccagg ggaggagctg cggacaacag

137401 ctgagcatca gggccattga aggaaagatg gaaccataca cagagtgcgg ggaatctatg

137461 ggggacacag atgactttaa ttctactcac tttgtgcatt ttattctgtt acattattta

137521 ttaccatttt cccagcattt tattatttat gctcttacac ttcttagtgt tccccctttg

137581 ctccccttcc cctagccagc cacccactac cggagtcaat ccccacaaat gtgtccacat

137641 tattgcccct ttcctgtgag gcaggcacgg gacccacgtt ccaggtgaaa acgctgatgg

137701 ttgagaggtg agaggacgtg ccaaaggagg cgcaggcgat gcaggtctaa gctgggactc

137761 caccgcaggc cgtgctcact cccagccttg ctctacgtca ctgctctctt ctgcgaattg

137821 gtaggggagg cgccttcaaa gtggggacac tgaccccagg gtccctctcg gacgtgctgg

137881 ctctgacaca gttcactggc ttaacagctt ccacctgcac agtgacaggc tgaagaggga

137941 agagatgcag cttgggccgt ctgtcgtttc ccaggcggcc ctgccctggg ctgctgacga

138001 agacgtttgt ctgagtttgt gcatgtatac cctggggccg atagtgcatg tctgggacag

138061 atttcacaca ggcaaataac acaaattgct taatcaaaca atatgggctc aaatgtccgt

138121 aggtgcccat aaactctgtg acaaacccct cgtccagcac agccactgct gggtgcaggg

138181 caggggtcaa ggcaggcaca ggtgcgttgt ccctgcctca cctgcattcc cacacagagt

138241 cctctgaaat gcaagcagtg acctctggta atctgttagg ggagtgagct actgtacaga

138301 gttgcttctg ggggaaatgg aataggggac ccgtaacagc acttcctgtt tgatatgaag

138361 cctgcacaca agaaggtgtg atgcttccag aaggttgtgg ctccagggcg ataggcgatc

138421 acacaccaga gggagggcag aagggacggt ttcatttaga gagaggccac cgtggtgggg

138481 cctccagtgc ccccagcaca tcctggcccc gctctgcttt ccgcctccgc tgcagtgggc

138541 aattccctgc aagaccgggt ccacttacct gctactgagt gcaccccagc cccagtcgag

138601 cgggagtccc tctgctgtct gtgctggtct cccctctgtc ccaggagccc aggaggcctg

138661 ctcgctggag cagccagagc tgctgaagtg ctaacactcc tgaagtgtgg gcccatgggc

138721 agagcagggg acaaatgctc cagcctctgt gctcaggagg agaggttggg aggcatggtg

138781 catgcttctc agcaggtgct aacaagtcca tctcccgatg ccccagcaca gaggcaacac

138841 cccacctcct actggtctct ccttccctgt tgcttcctcc ctgctctctt actcctgctt

138901 tctggggtca tatcccaaat atgctgcctg ctcctaagac cttctctcat attgctcccc

138961 tgggtgcagc ctacactgtg acagggatgg agtgaagtga ccgtcagaag tcccgtctcc

139021 ctggaagaga ccgccatttg tatgtcattc attctgccat ccattcattc aacagtctca

139081 tgtgaacaca cagtcttaca acagcaccta tgaatgaatg tcttcccagg gccttttgct

139141 tcacgttgaa ctcccagcag actcacgtgg gtctctgctc ccttcacgct ctgaacctat

139201 ctgccactga ctagcctggt tatgttcaca gaagacacgt gtgcctgggc tcaggtgacc

139261 actcactcct ctcatctttc ctccaacgag ccctgctgga atccaggtac agagcaggcg

139321 gggctcggtg ggggtgaaag ctgataaaat ggtagggtgc cctttgagaa agagaataga

139381 gaaacctggt caaaatcttg aaaagacctt tgaaaatcag gaagcctgaa atcatccttc

139441 attcctgtgc cgtcaatctg gcgcttacgg ttccgaatgt tgaggacctt tttaaagata

139501 gtgcttctgg aatcttctgc ctgtacacca agcagacagt cccagcatcc cttgggccat

139561 ggcagaagac acggtaatga gatgtcagtt actggccacc agatggcgct gtgatgccat

139621 tgacctggaa ggggctcagg ggagagtctg ggacagcagg aaagggttaa gaagccctgg

139681 gcagggaccc aacacagggc agaaactgag gcaggtttca gttgtgggta gtctccctac

139741 agctgtggaa cgggtgggaa gccctgctaa acggtaaaag acccactgtt gatggatatg

139801 gtcaaagcag ccctgctggc aggggcaggg gcaggggcag gggcagagag agaagtaact

139861 gtcccctagc acctggggag acccagagtg gcccacagga ggaaagataa gggacccctg

139921 ccccacatgt ctgcaggggg aagcagctgg agtcactggg gggtcctgac atgtgagcag

139981 ctggcacagg ctcctcctga ccagcaagag gggacaggcc tgcaccccag gaaacaccac

140041 ggaggggtct ccctgagccc gcaggggaag aagggatgca gtggtcaaca acaagggctg

140101 gtacttagtt caggcagggc caggcaggga cgatattgta ggcaccaggg accgtgcaga

140161 agctgcaagc aacccttagc catggggctg tgcagcaccg ggagtggtga atagaatctg

140221 gcccctgggt cccaccaagg gttcagagca ccagggggca gcctccttgg agagtcccat

140281 cagagcagtg acaccatcag agcagtgaca tcatagcaaa tgctcctatc agcacacaga

140341 gaacacagaa ctgtagaccg ctcacccagg ggaccccgcc cagagctggg agaggtcccg

140401 ggaagctgcc atggacccca ggctcctctg ctgtgtggcc ctttgtctcc tgggagcagg

140461 tgagccctgg gcactgtgtg taagtctgga cacgcagtca catccctgac acacaagcct

140521 gggggtggtg gctgcctcgg gaggctccct gtgctctgcc cccagctcct ctgtgtctcc

140581 tcccaacagg cccagtggac tctggagtca cgcagacccc aaaacacgtg gtcaaagcaa

140641 gaaaacagca agtgacactg aagtgttcct acatctctgg acacctgtct gtgtattggt

140701 acaaacaggt tcacggacag ggtcccgatt tcctcattca gtactacaat gggcaagagc

140761 gagaaaaagg gaagctgcct gatcggttct ccgtgaggct gctggatggc gaccgctccg

140821 agctgaccgc gagctcgctg gagctgagcg actcggccct gtatctctgc gccagcggcc

140881 ctgacacagc cctgcaggcg cggcagcctc ctgcacagaa acactccgcc ccactcagga

140941 agcggcagcc gccagccaca caggctcagg cctgagagag cagctcagct tccccaggcc

141001 cggttctgct gctcgcagat ggggtgggat gtgatgccca cagacatcat ggcagcttat

141061 cattgtcctg catatacacc tggtgggaag tcctgttcct gggtctcaca ttccgtaacc

141121 ggaagaccaa aacatcactc aagtctgctc ctcaacgtct gtgtatctgt cccatccacc

141181 tctcccagaa gtgtgtccac atctggctcc ccttttctgg gtttagatgc accgccctgg

141241 ggagatctca caccatctgg gttgtcgtct tcttacccgg cagcttcaat aacatctcac

141301 ttcatggcaa caaatactca tcaatccttc ctaatgacaa caaagaaacg cacaactgca

141361 tgcactgtgg ccgtcacctt gacctacaca aagccctgaa gcaagttttg ggtcatgtca

141421 agtgtgggac tgtgagtaga tgcgctacag gtgtttaaag gctcattatt ttattagtag

141481 tgtcagcagt tcttcacggg ggatataaac tgtggaacaa ggaagaaatt taaaggcagc

141541 agtttcactg tctcacccgg gaggtggctc ctgtcctgtc cccccgtccc tcacacagct

141601 gtaactgccg gggccatcgg ggcacctgca gagccgagca ccacgcagaa acccaggtcc

141661 ccggcctggg ctgggtgtgc tggtgctgcc tcaccctgaa acggaaatca ggctctcagg

141721 tgactctgaa ggtcctctgg agcatatggt gggggcccca tcagtcaggg gatggagaga

141781 aatcagagcc agagggtctt ctagggttta tgaggctgcc cctgaggttt ggccaccaga

141841 gaccaggagg gcccctgtgg gaggagggac agtgagggga tgctggacga ccagcaaagt

141901 agaaaaggag gctgagggac gactgggccc cagagcccta aaggcatcag aatgccctca

141961 ggggttgccc ttagatgcaa gagcacaggg acggggacac acagcagacc agggaggcat

142021 gaggatctgc gggcaggatg aagcacacct gagtcccaca ctgtgaacac ggagggtgag

142081 gacgactctg ggactcccgt cagttcagcc acacgccaca ttcttctccc tctgagctgt

142141 gccctttccc gtctgcttgt gattggatgc tctggggaag gggcgtgtcc cttgtgaccc

142201 aagcagtgtg agtgacacct cagagagact ccctggacag ccctgctcct gcccagccac

142261 ccccagactg agagggaccc tcagtcagcc tggctgctga catgagcggc ggcctcctgt

142321 gctgcgtggc cttgtgtctc ctccgtgcag gtgggtcctg ggctgggccc ctctgtgggg

142381 acagtgccca ggcccagcac cgagcggctc cctggaggct gcagcatctg ttcccctgtc

142441 ctccctgcag gtctggggat ggccggcgtc actcagtccc cagcctccca ggttgtgaca

142501 acaggacaga cggtgacctt gcggtgttcc caggacttga aacacgacgg catgtactgg

142561 taccgacagg acctgggtca cgggctgagg ctgatccatt actcagtggg ccctgggatc

142621 atggacaaag gagaggtccc cgatgggtac agtgtctcta gatcaaaaca agaagacttc

142681 cccctcacgc tggagtcggc cacccccgcc cagacatctg tgtacttctg cgccagcagt

142741 gaacccacag tgctgcatgg ccgcctcctc tctgcacaga aagtcagggg gaggcccagc

142801 gcccggactc agggaagccc tgtgcagact cctccacctt ggagccccca gtggccccca

142861 cctgcagggt gaccctctgt gtgtgctggg gttgccctcc aggtcagtgt ctcccaggct

142921 gggaccaaag cctggacctg gaacacacag ttgtccacct gtgatggctg tctgtctgtg

142981 tgtcctctgc agctggttca ttagctggtt cattagcaag agcttcccaa gctcttgctt

143041 ttcttgtcac cagccggagt cagaggccat gagggtgggc ggtcatcagt cgccagacac

143101 atctgtgccc acttgtgtat cagacacaca tgtcttacag gccagtgatg gtgagtcttc

143161 tcctggaaag aagctacgag gaatctgttt tcaaatgcct ggtgactcag aaggaccaga

143221 ggcttttctg tgggcagtgg ggacatgaaa gctcatggaa gtcagggagc gcagagggga

143281 cacttggtcg tgacctgcag aaggagtagg agctccctac tgaggtgttc ccagggcatc

143341 gctccatctg tgtgtctctg agtgtgtcag tgtgtccagg acaaagtcga gtcaccccga

143401 tgcccaccat cacctaagaa agggacaagg tggagcacgt gtgactggga gagtggaggg

143461 gatggtgaca ggacagggtg ctcccaggaa atcccgaata gcagagagga ctgtgaattc

143521 accctttggt gtgttgtact gatgacactg ttggcatgtc ctagtgaaac tgccagctga

143581 gcagggtgac aggtgaatga gactcagtgt gagtcccctg catgcacaca gcacagatcc

143641 aaagtgacag ctgtgtttgc attttgttca gaaggagctt agagtactca cccacatgtg

143701 cactaatgca caccatgtgt tcctgagggc aggaggactg atagagacag tgatgtcact

143761 gtgggagctg ccttgtgggg acaggagacg tccctcctcc tctgctcctg ctcacaagga

143821 cctgacccag aaaacctccc actcctgacc tgaccctgcc atgagcacca ggctcctctg

143881 ctgggtgacc ctctgtctcc tgggggccgg tgagtcctca gaaaaccagg cagcctcagt

143941 gtgtgtgtgt gtgtgtgtgt gtgtgtgtgt gtgtgtgacg attacagatg cttttctcat

144001 tctattgaca atttctgttt tccaggtaac acagaggctg gagtctccca gtcccccagg

144061 cacaaggtca ccaagagggg acagaatgtg acatttcagt gtgatccagt ttctggacac

144121 actgttcttt actggtaccg acagacactg gggcagagtc cggagctatt ggtgtacttc

144181 caaggcaagg accctgtaga cacctccggg atgcctaagg atcggttctt cattgtgaga

144241 cctgacggca cctcctccac tctgagcatc cagcctgcag agcctgggga ctcggccatg

144301 tacctctgtg ccagcagtga aaccacagtg tggcaccgtc cccaactgcc tgctcacaaa

144361 ccctcctgtg cccccctctg ctcatgggtc tgagactcct cagcaaagga gttgccctgt

144421 tcttcctttc tcaaggagca taagtgcatt tggattttca atgctttttc tggcagtaat

144481 gactagaaca cagcatgctg gatttctggg gttccttcat gtacctcaac attctgtaat

144541 tgaggataag ccccagagct ctccctcagc tgtggcgaaa tcttctcttg tgaaatagaa

144601 agtttcagca gatgtgagac aacctctgag tcctgagtgt cactgatgat gacacatgat

144661 gcccacatgt gagcagccat gcagagctct gttaggccag atgcagccac cagccttaaa

144721 aatcaatagt tacactcctt agagaaaact gtcattggaa tcactcacag acaccggtca

144781 tgcacaataa tggacattga tgcatgccca tatgttgttt tattattttt cttgacaatc

144841 ctgcagtcat tctacatttt atttccttct tctctagcat attttgaata gaatttaaat

144901 ttatcttgga ttaataaaaa ccctacgtga gaaggtttta aagggaagtc attcaataag

144961 atccaagtaa tagttgtact gatctcccct tattttgtgg agatcctttc caaggcccag

145021 tggatgcctg aaacccctga tggtttaatg gaacctgatg tttactgttc tccccccaca

145081 cacacctttc attttgaagg aagcactttc tggcatctct ttggcagatc tggttgctag

145141 catcatggcc ctgcatgtca cggccgtctc taagtacaat aatggtgact ggacctcagg

145201 cactgcccgc ccacaacagt cagcctgatg acagagacgg cctagggagt gatgggcagg

145261 gacagagcac agtgtggaga cgctgggcaa ggggctgatt cacatcccgg gtgggacaga

145321 agtgggacag tgtgagatgt caccacacta ctcagaatga cacatattta aaactgttaa

145381 attctttttt tctggaattt atcatttaat attttgggac tacagttgac ctcaggaaac

145441 tgcatctgcg gaaagcgaaa gtgcagctaa ggggggacta ctgtgggtaa aagtaaaatg

145501 tgcgacaata atatcaagac agggagcagg taatgcgtag gtgtcttacc gttgtgagag

145561 tctcctgatc tacgtggagg ggtgtaggct gtgataaagc cgagaggtcc attgcaaact

145621 ctagagccac catgaaaata gcagagttgt ataactcata ggccaacaaa cacagaaatg

145681 gaactcttat aaaaatgcct aattagtaca caagaaagca tgacacaaaa aacaagggaa

145741 caaagaacag atatgctaat agaaaacaaa cagcaaaatg acagacatga aggtaaccag

145801 aacaatatgt acatcaactc caaataatct aaatgcctca attcaaaacc attgattgtg

145861 atcatggata aaacacaaaa aagcagatgt gtgccacctg ccaccgcggc ctgcaccagc

145921 agcagaaggg ctcacaggtg cactggagca gacacaccac gctgacactg gtcacgtgaa

145981 agctggcgca gcaccgtgag tatcacacaa agcaaatctc aaagcaaaga ctgtgatctg

146041 agacaacaaa ggtcatattc taatgataaa aaggtcaact gatcaagaag acaaggcaat

146101 cagatacact tatgcatcta ataacagggt tttgaaatgc tgaagccaaa aaggcaaggg

146161 acaaattcag aattattgtc agatatttca tcacctgtca gtaattgatt gaacaacttg

146221 accaaaaaag aaaaatagtg gggatactga agccatagag aacattatcg atcaccttga

146281 cctcacacac atttacggga tcgttcaccc cacagcagca gaatgcacct tcttagcaaa

146341 ggcacacgga ctatgcaaca ggataaactg tggactttga ccataaaata agtcacaata

146401 cattgaaaag aattgaagtc gtacaaaccg tgttctctga acacagaggg gctgaattag

146461 aaatcaataa ccaaaagagg tccagaaaaa tccctaatat tagaaactaa ataacaatat

146521 tctaaataac ccatagggca aagaacaaat caaaagtaaa tgataaggga ttctaactac

146581 tgaaagtgag aatataacat atgaaaatga gtgaatgccg cactaggaat acttagggag

146641 aaacctgaaa acaagggaat aattcccttc tcatcctgag ctaccactat tttacccaaa

146701 tcaaaaccag gtatcaccca aacaaaacaa aacaaaaaac aaaaaaattt taaaaacccg

146761 agagggaata tcgctgatga gcacagatta ataaaccttt ggaattccag tgcatgaaac

146821 acgacactga aaaggataac acagcacgac caagtggggt ctgtctcagc attacatcct

146881 catgactgtt aatatttaaa aggacgtcca ggaaggttaa catctcaaat gctaaaatgt

146941 gaaaaatcat gtgattatct ctatagatgc ttaaaaatac cttgatgtat tccaacatct

147001 gtttggataa aaactcttct taaattagaa gtaattggga acaccctccc cctaatccag

147061 ggcatctgtg agaaatctag agttagtaac agttctggag gcagaccgcg gtgaggtgga

147121 ctgtggtaaa gcaaagatgc atgcacactg ccatgcacag gcccaacact aagagtagca

147181 gggaaggggg ggatagtcat ttcaacagta gtgcctaaca acccggatcc atagagaaaa

147241 ataatgaatc tcaatgctta cctcactcca taaagcagaa ggggggcaaa cttggcctct

147301 cctggttctt ctacggccca gaagataact attggggttt atataagtgg ttggaaaaca

147361 ttatcttttg tgacatacaa aactacgatt tcaatgtcca cacacaaagt tatgggaaca

147421 tgcccccact cagtaagcta cctagtgtct ctggctgacc tggcacagca gtggcagagc

147481 tgagtaggtg ggacagaaaa cagaacatat tcaccacctg cctctttaca cagtgatttt

147541 gctgactcgg ccacgcacag aatgaaattg aggtgagcca acatgacaga tctattaagc

147601 ttctgaaaga aagcgtagca cattcccttc atgaccctgg agtaggcaaa gattgcttag

147661 agaggacaaa aactattgga atccttacaa tcacatattt tgaattttgg ccaagtcgga

147721 aatgtaggaa gaaatgctcc acttcctcac ataatctgtc aaccaaggag ttaaagaaga

147781 actcttcatc cagagtggta ggaagggtgg agacgggtgc ccagaggaga ggctgtgtgg

147841 ccaggcgatg gggtgggcag ccccacattc gcacacagat gagctgggaa gaacatatgg

147901 ggagcgagac agacaccaaa accaagggtt tcttttatta ttattattat tattattatt

147961 attattatta tttcttttta aattatttta attttaatca ttgtttaagt acagttttct

148021 cccttttact cccaatccag cccattcacc caacccttcc cacttccttc ccatgccacc

148081 ctccccctag tttttgtcca tgtgtccttt atatttgttc ctgtaaaccc ttcccattct

148141 cccctgaaat tccctcctct ctcccctctg gtcactgtca gcctgtcctc tctttcagtg

148201 tctttggtta tattttgctt gtttctttgt tttgttgttt agattcctgt taaaggtgag

148261 atcatatggt atttgtcttt tactacctgg cttatttcgc ttagcataat gctttccagt

148321 tccatccaag ctgttgcaaa gggtaggagc tccttctttc tttctgctgc atagaattcc

148381 attgtgtaaa tgtaccatag ctttttgatc cactcattta ctgatgggca tctaggttgc

148441 ttccagcacc tagttattgt aagttgtgct gctatgaaca ttggggtgca taggttcttt

148501 tggattggtg ttttagtgtt cttaggatat agtcccagca gtgggattgc tgggttgaaa

148561 ggcagatcca ttttcagttt tctgagaaag ttccaaactg ctttccatag tggttgtacc

148621 agtctgcagt cccaccaaca gtgcactagg gtcccctttt ctccaaacct ctccaacact

148681 tgttgttgtt gctttgttta tgatggccat tctgaccggt gtgaagtggt atctcattgt

148741 ggttttaatt tgcatctctc tgatagctag cgatatggaa cattgtttca tgtgtctttg

148801 gattttctgt gtgtcctcct tggagaagtg tctgttgaag tcctttgccc attttttaat

148861 tgggttgctt gtcttcttag agtggagtca tgtgagttct ttatacactt tggagattaa

148921 acccttgtct gaggtatcac tggcaaatat gttttctcat acagttggtt atctttttat

148981 tttgatactg tcttctttag ccatgcagaa gctttttatt ttcatgagat cccatatgtt

149041 tattctctcc tttatgtccc ttgctctagg gtagatgtca gtaaaaaggt ttctgcatga

149101 aatgtctgag attttcctac ctacgagctt ctctaggacg ttaatggtgt cacggtgtac

149161 atttaagtct tttatccact ttgaatttat ttttctgtaa ggtgtaagtt ggtggccgag

149221 tttcattttt ttttgcacgt agctgtccag ttctcccaac accgtttgtt gaagaggcta

149281 tttttactcc tttttatatt gctgcctcct ttgtcaaata ttaactgacc gtgaagactt

149341 gggttatttc tgagctctct gttttgttcc actgatccat gtgcctgttt ttatgccaat

149401 accaggctgt tttgattata ctggccttgt aatatagttt agtgtcaggt attgtgatcc

149461 ctcctacttt actctttttt ctcaaaattg cagcagctat tcagggtcat ttatgattcc

149521 atataaattc tggaagtgct tgttctatgt ctgtgaaata agccattggt actttaataa

149581 gttttgcatt gaatgtgtaa attgctttgg gtagtatgga catttggatg atattaattc

149641 ttccaatcca tgaacacggt atatatttcc atttgtttgt gtcttccttg atttctctcc

149701 tcagtgttat gtaaaaaccg agggtttcag cacagaaaac tgaagactca aaacctctgg

149761 ctgtaaaaat ctgtgggggc tgcagtggtg ggagaaactc ccagtctcac aggagcattt

149821 gctggagggc ccagggtgtc ctagaatgta tacaaaccct cacacctggg aatcagcacc

149881 tacctaaaaa tgcacaatta gcttgtggga aatgagagag aagtgacaga aaggctgtga

149941 gagagccaag caagtggcat tgttccctca gggacgcctg cccctcatac agcctcacaa

150001 cacactgaag atggttgtcc caccctggtg aatacctaag tttctgctcc ttactacata

150061 acaggtgtgc cgaaacaaag aaatacggcc caaatgaaag aacagatcaa aacttcagaa

150121 aaacagtaag caacaaggag acagacaacc tttcagatgc agagttgaaa acactggtaa

150181 tcaggattct cacagaattg aagatctcag atgcaaaatg ggaaaagaaa tgaagttacc

150241 caaagggaaa tagagtaaaa tatacaggga accaatagtg aagggaagga aaccaggact

150301 caaatcaatg ctttgggaca aaaggaagaa ataaacatcc aactggaaca caatgaagaa

150361 ccaaaaaccc cccaaaatga ggagaggctt agggacctct ggaaccactt taaatgtacc

150421 aacatctcaa tcataggagt gccagaagga gaagaggaag agcaagaaat tgaaaaccta

150481 tttgaaaaaa tcatgaaggg aaactgccag tttggtgaat gaaatattct tccaagatgg

150541 ccaggaagct cagagagtct caaataaatt ggacccaagg agcacacacc aaggcacatc

150601 atgattaagt tacccaagag taaagataag tagagaatct taaaagcaac cagaggaaag

150661 aaaagagtta cctacaaagg agttctcatt acactatcag ctgatttatc aaaagaaacc

150721 ctgcaggcaa gaagggctgg aaagaaatag ttgaagtcat gcaaggcaaa gacctacatc

150781 caagatgact ctactcagca aagctatcat ttagaatgga aaggcagaga aagtgcttcc

150841 cagataagat gaagttaaag gagttcatca acaccaaacc cttactatat gaaaagttaa

150901 aagtaagaaa tagaggaaga tcaaaaactt ggaacagtaa aatgacaaga aactctgaac

150961 tatcaccaat tgaacctaaa aaaacaaaac caaaaactaa gcaaacaata agacaatagt

151021 atcacacaaa tggagatcac ctggagtgtt attagtgggg aggatgaggg aggggaatcg

151081 gggtagaggt acagggaatg agaagcataa ctggtaggca caaactagac agggggaggt

151141 taagaatagt gtaggaaaca gagaagccaa agaacttctc tgtacaaccc atggacatga

151201 agtaagggtg ggggaatgat ggagggtgag agggtgcaga gaagatgagg agaaagtgga

151261 ggaaaaaatc aggacaactg aaatagcata attaaccaaa tatatcttaa aataaataaa

151321 gatatcttaa gttaaaaaca cacacattat cctttatgaa aatttttaaa atcctgttca

151381 tcaaaagata ttaagaacat tcaactatat atctgaaagc gcatgtattc aaaatataaa

151441 caattcctag aaatcaataa ttaaggaaga gaaatcaatt tttttaaaaa tataagaagt

151501 tgaagatggt gctccaaaaa acaaaagtga tatgtaagta gaccacttct tacttccgtc

151561 ttcacagagg gtttctttca gtgctgagga gactttttgg ctgatgggag cccagcgtag

151621 accctgccca ggaatcactg ggtgaagaca gagctttgct gagggtcctg ccttcccttc

151681 cctgggcagc tgcgtgcatg gcgtcctctt tccatgatta ttcactgatc aggtggatga

151741 cgtttattcc cgtttggaaa aaggcatcac acctccctca ccaccacagg caaagggtga

151801 gcctgagaaa ggtaaacaag aaagcagcag gggctgtgca gagtccttgg ggatccctga

151861 taattaattt agacagaacc cagactgggg gtttctttgt gactgactta ggcagacccc

151921 agccagctcc agcagggtca aaagcgctgc tgcacaagtg accacttgag ggcgctgtgg

151981 ctccacggag taagatgctg atggcactct gtgggagaga gaggggtcat gggaagaatc

152041 ttggcttggt ttctgcaaga agaggattat ctgatgaatc tgaggttcac caatcttatc

152101 ttctcacatt aactgacaac caggtgggta tgtcttagca aggagccatg aagtaggcct

152161 tgcagggagt taagtttctc ctggagccta gtttggtgga gagtgtgatg gggcttcagg

152221 gaggagcctg ggtaacctga gccagggtag agcaaggagc acctgctcaa ggcagagaca

152281 acctgtgctg atggccacct gccctgtacc ctcggggccc aactcagcac agccagctgc

152341 acctgctcat aggtgatgga ccaactcagg gaacacagga ccagctgagc ctgtcccgga

152401 ggtgtgggtg cccacaagat gctacaaaga cagtgacatc agaacagtga cgtcacaggc

152461 aagccctcca aagagaacaa agggagggag ggacaagcct ctgttgtctc accccaaaga

152521 ccagagccct gagcagagac atggcccgtc tggtgctgcc tgaatcctcc aggggcaccc

152581 agctgctctg ttgcatggtc ctgtgtctgg tgggaccagg tgagtctcag ctctggtgca

152641 acttccctcc aacccagcgt ccttgaccat ttctggagtg tccatctgcc tcctgggctg

152701 gtctcagctc catctccttc ctccacaggg tcagtggctg ctggagtcac ccagtccccc

152761 agacacctca tcaaaggcct cggtggggag gctgttctga aatgccaccc catctctgga

152821 cacaaccgtg tgtactggta ccaacaggtt ctggggcagg aactcagctt tctcattcgg

152881 tattatgaac agcaggagta tgggaaagaa aatttcccca accgcttcaa aggaaagcag

152941 ttcagagact acagctccga gctgaccatg cacaccttgc agctggggga ctcggccgtg

153001 tacctctgtg ccagcagctt agacacagcc ctgcagggcc catgcctttc tgtacccaaa

153061 ccctcctgtc ccagctgatg tcacaggcag aaggaggggg cctcagccca gttccacaga

153121 tggttttcat tgttttctgc cacacccctt gccctgctga gctcagcaaa agccttcctg

153181 aatgttctcc cagctctgcc accagtgtgc aattcccagc acatggcagg aaacccttct

153241 ctgtgacaga gtggagttca caatgtgtgt tgtggtgccc ggtcctggtg aaccccgtgt

153301 gtccacatta cagagtaggg agcactcagt gcaccatgtc atccacaggg agctgaagct

153361 actggacctt ccaaacgtcc cttgcacgtc cattcttcat ctccacgcct ccgaagtcaa

153421 caagaccctg gattccatca tcgtacaagg gccttggtcc ataccgtaga acagagcaca

153481 gtctctcctg tttcaagagc atctgagctg ggatggggag gtcacctcta ggagtggggg

153541 acacggttga ttgcctccca ctttcaagga cgtagagctt gaagacagag cacatgtgtc

153601 tgtcctgtca tagttccaga cagagcaaga ccatgatatg cgcctctgag aggagctaag

153661 tcacctgggg aatttccagc ccatccagag atccttggag gaaagcagaa gccctctagg

153721 aatgaatgtc aaggagcact ctgggagctc atgtgggaaa tgcgtgtttc tgaactactg

153781 gagagggcgg gggatgggga tttctcagcc tgtccttagc tttacccctt gtggtcagga

153841 aatatgaact tgccccatct gcccaggtgc ggagctgcag acaacacctg agtgtcacat

153901 caccctggcc cctgatattt gagtccgtgt cccttctcac tgctgggtgg ttgttgttgc

153961 tcatcaaagt gacatcagga tacacagtca cagccagggt cttgtcacac tgaggtacga

154021 cctgtgtaag agggagcagc actgggagcg ctggctgaat atcccatcca cgtcactgac

154081 gaggctcccc tctttgtcac agaaagcaca gtcatggggc cacaggtgtc tagtccccct

154141 ggggcctcag ctggaaccag gggagactct tacccgtgag catgcagaga cctgagcttg

154201 ggtggctggg ctcccaccag tgggtgaatc gtggtaaaat ataacagaat tctcagactc

154261 caatggcctt gaccccaagg ctatgaaaac tggatatggg tttgttcctt taagggagag

154321 gattgctatg ggctgacatt gaaattatcc agagtttatg caaagagaaa aactaggaga

154381 atgtatttga agtgaatatt gatcagataa gtcaccacca aagcatgagg attgctatgg

154441 gaacatttcc tagtctacag taatgggagg aaaaactgta cagggaagga attgtatcac

154501 tgtgcataga aggaatttcc ttaccaaaag tcaggggaaa gcatgcgtac tgctaagatt

154561 tcttttaaaa agggacttac acattttccc atgctgcact gctccactgt ctctatgtca

154621 ctggtttgtt cctgtggggg aggggaggca ggtgagctct gtagctgaag catctgtgaa

154681 ggaccgtgat gtcaccgagg ccctgagagg ctcacttgtg tttccccaga cagaactgga

154741 agaggtgaca ctgtgcgctg gacctgaaat ggtcagcagg gtctgcttcc gtgtggctct

154801 ttgtctcctg tgggcaggtg ggtgcaggtg ggcttcctgc cctggaattc ccagccttca

154861 gtcccaggct tggtcttagg atgacaacat aagggtttgt cactttctcc acaggacact

154921 tggaagcagg aatcacccag agtccaaggt acaaagtcac agggacagga aaaaggtgac

154981 actgaggtgt caccagactg ataaccataa cgctatgtcc tggcatcgac aaaacctggg

155041 ccatggcctc aggcagatat attactcagg gggtgttggg gtcgccagca aaggagaggc

155101 tcccgatggg tacagtgcat ctagagtgaa catggaggac ttcctgctca cgctggagtc

155161 ggccaccccc tcccagacat ctgtgtactt ctgtgccagc agagacgtgc tgatccacat

155221 cgctgcacgg ccaccctctc tctgcacaga aaggactgtt gaggccctgc actccagatt

155281 catgcgaccc ctgagcaaac tcctacacct tagagccctg cagcaccctg cctccagggg

155341 aaccctgggt gtgtgatgag gtccccctct aggttagtgt gtaccaggcc tggtgtaggt

155401 ccagggcctc agacacaagt ctgcaatgtc gctgtctgtc agtctgtcct gccaggtgct

155461 ctgttagctg ggaaggtgtt tgctctttct agcttttctt gtcatcagcc tgagtgtgat

155521 gcctccaggg cggggagtca tgaacaccat atccacgtag atccctgcga gtgtcacatg

155581 caggtgtgta aaggtactgg acactgagtc ttccttcaga cagaagctac aagaaacatg

155641 ttttagtgct ggtgactaag aaggacacgg ggctttgctg tgggcagggg agacacctaa

155701 tagtacgtgg aggttaatga gcagagggag ggcactagga tgagccctgc agaaggagtt

155761 ggagcttcca actcagatat ttcccggggc atcactcaag gtgtctccaa gcaagcgggc

155821 ccagagagtg gccctcaggt gtgatcccat tcctggacac tcgacattta catgtgctga

155881 caggtcccgg gacagggccc agagtttctg atgtactttt acctcctgga tccccagact

155941 cctgcgggaa acctaggacc ggttctctgc tcagagacca ggggaatccc actgcactct

156001 gaggatccag cctgcagagc caggggactt ggccgtgccc ctctgtgcca gcagctccag

156061 cacagagtgg ccccgtcacc ccttcatgcg cacaccctca tccttctccc tccatgcagc

156121 tcccccactc taaagcagcc cttctttgct catcattccc cagtgactat gcagttctgg

156181 gcatcagctg tgctttgggc agacagaaca aagccctgcc cggtgagcct cagttggctg

156241 ggtgttgtac tgccaaccaa agtctttctg gttggattcc cggtcagggc acctgtctgg

156301 gttataggtt tggatctcat tggggcacat aggagaggca actgattgat atttctctca

156361 cattgatggt tgtctacccc tttctttctc attccctctc attgctatgg aagtaaataa

156421 acaaaataat taagtaatga ggatttgcag agccagctca gtgctctaag gcacagactg

156481 aacgtcatgg acacgaacta cctttggtag gactgtgagg ctctgtcccc aaacacagct

156541 ccgctgtggg tgagaagtgt gcctgttcct cagggcttgt ccgcatctgg aatgttggcg

156601 acaccctctt cctgcacatc cttctctgag ccgccctgtc accttggggg ctctgcagca

156661 tcttctccaa cgtgccttcc tgttgggagt gaaggtcctc accgcttctg tgtgtattgt

156721 ctgaagaaac ccaggtgata ccagaaaggc gtcatgccct ctgccaggct gcaggagccc

156781 acactctatt gtgttttcat tttcccgcca ctccatcctg agtgtcaggc tgcagcgtgg

156841 gtccacgacc tctcggtttc atcttgaccc catgggtaaa tccttctaga gatgagggtc

156901 ttcttggtgt cctctcattt tgacttgaag taaattccct gaattcttgc actcaaaggg

156961 atgtgacatt ctctagataa tatgttttat ttctttttat ttattttctg ttatggtggg

157021 atatattatt tgttataaat atagataatc gagtccctgt gggggggcag ctgcagacat

157081 ttggcaaagc cccagccatg ggcagagggg acaggccttc agatcatggg tttctgggag

157141 tcctgagagg taaccctcgg ctctgacgct gggactggat gtcacagacc cagtgccaat

157201 caaggtgcct gagccttggg tagcaggtaa tgacagtggc tgttcctcaa cacccaccca

157261 aacactgtag tagccatgac accagatctg ttcacccagg aacgagagtc catgggaggg

157321 acatttcctg gtcgtgtgtg gactgtgctc gggtccctgg gcctcctggg agggagggag

157381 gcctgggctc ccagggcagc tgcctgtcct tgggaagcca tcagggaccc cttcccggac

157441 tgtgtcccca tttatgccac tgcccagcac ctgaccctgg ccactctgct tccttgggac

157501 actgttttgt acccgagttt gcttgtctcc cacaacgctg ccgcttcacc cagaaccagc

157561 tccgtgctca cagagcaggt gcccactgtg ggggctgccc ctcccgtgtg ggctgtgcgt

157621 gggggtctca gtcccagcgt ccccagctcc ccattgggtg tcttttctcc atggctgctc

157681 acaaaccgta tgggttacat ttcacttgtg tttcaatttt aactcttatt cacgcacatt

157741 tgcctccaat cttgaaggta atattatctt cccagtcttt gctgatttat attttggaga

157801 taacatgaaa cacctatttt ccttttgcat tatgtttatt ttgagcaatt gagataaaaa

157861 atgcctcaca cttccacttg tgtgttttcc ttcctacttg gactagatat tatttcctcc

157921 cattcccctt ctctccctct gcctttcaga ggacaatgtg aaggaagccg gtcccagact

157981 gcagctgcag atgacgtgag agtgctccag gggaacgggg tagggtgtta agcatggaaa

158041 ctgaaagaaa tagggtcacg gtgggtaata aagttgggat tagggttcag aatgttgaga

158101 tagcttataa aggatagtgc ttctggaatc ctctgcctgt agaccaggca gacatgccca

158161 ccatcccttg ggctgtggca gaagacatgg taatgagatg tcagttactg gccacgagat

158221 ggcgctgtga caccactgac ctggaagggg ctctggggag agtctgggag aacaggaaag

158281 ggttaagaag ccctgggcag ggacccaaca cagggcagaa gctgaggcag gtttcagctg

158341 tgggcagcct ccctgcagct gtggagtggg tgggaggtgc agctgaaagt tgaaagaccc

158401 atggttgaag gacttgggca aggcagccct gctggcaggg gcaggtgcag agagagaagt

158461 aactgtcccc tagcactggg ggagaccagg agtggcccag gttaggacag tgaagggacc

158521 cctgccccac atgtcactga ggggtactag gacgcctggg gctggcacag gttcctccac

158581 ccagggatag gggacctgcc ttcacctcag gaggcaccaa ggaggggtct ccttgagcac

158641 aaagacaaga agggatgtag ggccagcagc aagagctggt gcttagttca ggcaggggca

158701 ggcagggaca atgttgtagg cacttggggg cggtggggaa gctgcagcca cccttagcca

158761 tggggctgtg caagacatgg aatgatgcat agaagctaga agcggggtcc caccaagggt

158821 acagagcacc agggggcagc ctccttggag agtcccatca gagcagttac aacatcacac

158881 caatgacgtc atagcaaatg ttcctatcag cacacggagc acagaactgt agaccgctca

158941 ccccagggga ctccaccctg agctgggaga ggccctgggc agctgccatg ggccccaggc

159001 tcctctgctg tgtggccctt tgtctcctgg gagcaggtga gccctgggca ctgtgtgtaa

159061 gtctggacac gcagtcacat ccctgacaca caagcctggg ggtggtggct gcctcgggag

159121 gctccctgtg ctctgccccc agctcctctg tgtcttctcc caacaggccc agtggactct

159181 ggagtcacgc agaccccaaa acacgtggtc acagcaagaa aacagcaagt gacgctgggg

159241 tgttcctaca tctctggaca cctgtctgtg tattggtaca aacaggttca gggtcagggc

159301 cctgatttac tggttgagta ctacaatggg gaagagcgac aaaaagggaa cctgcctaat

159361 agattctccg tgaggccgct ggatggctac cgctccgagc tgaccgcgag ctcgctggag

159421 ctgagcgact cggccctgta tctctgtgcc agcagccctg acacagccct gcaggcgcgg

159481 cagcctcctg cacagaaaca ctccgcccca ctcaggaagc ggcagctgcc agccacacag

159541 gctcaggcct gagagagcag ctcagcttcc ccaggcccgg ttctgccgct cgcagatggg

159601 gtgggatgtg atgctcacag acatcatggc agcttaggtg tccttgtcct gtttatacac

159661 ctggtggaaa gtcctgttcc tgggtgtcag atttcataac ccaaggagcc agaaatcgct

159721 cgtctgcccc tccacacaca tggatgtgca cgactgcccc tcccccctcc cagccccatg

159781 cccacactga ctcccctttt cctggtttaa gtgagcgggc ctggggggat cacacaccat

159841 cggggattgt caacctcttc cctggcagct tcaaggagca tttcacttca aggtagtgaa

159901 tatccatcca tccctcatga tgacagcaaa gaaaggtatg attattttcc ttttcttttt

159961 aaaaacattt tatttactta tttttagaag ggaggagagg gagaaagaag gagagaagca

160021 tcgttgtgtg gttgcctctt gtgggccccc tactggggac ctggcctgca acccaggcat

160081 gtgccctgag tgggaattga accagccaat gagcaaggca caattttccg cactgttgcc

160141 ctcacctggg cctgcacaga gccccgaagg aagttagggt tcctgcaaag catggactgt

160201 ggggagatta gctgcaagtg tttaaagact cattatattt ctcataatta atgtctgcaa

160261 tccttcacag ggagacattc cctggggaac aatgcctcaa cttaaaaaga gcatttttca

160321 ctctctccct gttggcagcc gcctctcccc acacccctct gtaacccgag gggccagcag

160381 ggcacctgca gagctgagca ccccacagag agggagtgtc cccctggact ggatgtgctg

160441 gccgctgcct caccccaaca tgggaagcac attctcaggt gactctggaa gtcctgggga

160501 gcacgtggca ggggtcccat ctgtcggggg atggagataa aacagagtca aaggatcctc

160561 aagtgttgac gagcctgacc ctgaggtttg gccaccagag accaggaggg cccctgtggg

160621 aggagggaca gtgagtggat gctggacgac cagcaaagta aacaggaggc tgagggacga

160681 cttgggcccc agagccctca aggcatcaga gcgccctcag gggctgccct cagacgcaag

160741 agcacaggga cggggacaca cagcagacca gggcggcatg aggatctgcg ggcaggacga

160801 agcacacctg agtcacacac tgtgaacacg gagggtgagg ctgactctgg gactcccgtc

160861 agttcagcca cacgcgttct cctccctctg agctgtgccc ctgcccttct gcttctgatc

160921 tgacgctcag ggaaggggcg tgtccccgtg tgacaagctg gccctggggc ccaggcagtg

160981 ggagtgacac ctcagagaga ctccctggac agccctgctc ctgcccagac acccacagac

161041 ccagagggac cctgagtcag cctgcctgct gccatgaacg gcagcctctt gtgctgcatg

161101 accttgtgtc tcctccgtgc aggtgggtcc tgggctgggc ccctctgtgg ggacactgcc

161161 caggcccagc accgagccgc tccctggagg ctgtagcatc tgttcccctg tcttccctgc

161221 aggtctgggg atggccgggg tcactcagtc cccaacattc caggttgtga caaaaggaca

161281 gatggtgacc ttgaggtgtt cccaggactt gaaccataac aacatgtact ggtaccgaca

161341 ggacctgggt cacgggctga ggttgatcca ttactcagtg ggtgttgggg tcaaggacaa

161401 aggagaggtc ccggatgggt acatcgtctc tagatcaaaa caagaagact tccccctcac

161461 gctggagtcg gccacccccg cccagacatc tgtgtacttc tgtgccagca gtgaacccac

161521 agtgctgcac ggccgcctcc tctctgcaca gaaagtcagg gggaggccca gcgcccagac

161581 tcaggggaac cctttgcaga ctcctccacc ttggagcccc cagtggctcc tgcctgcagg

161641 gtgaccctct gtatatgtag ggtgaccttc tgtacatgtg gggtcatcct ctaggccagt

161701 gtctgccagg atgcaaccaa accctgggcc ttggacacaa aggtgtccac ttggctggct

161761 gtctgtctgt cctctgcatc tgctccattg gctggggtga tgcttcccca gctcttgctt

161821 ttcttgtcag caacctcagt cacaggccat cagggtgggc agtcatcggt aaccagacac

161881 atctgtgccc actggtgtat cacacgcagg cgtgtcacag gtcagtcacg gtgagtctcc

161941 ttctgaaaag cggccatgag aaacccgtgt ttacatgcag ggcaacttag aagcacaaga

162001 ggcttctctg tgggctgtgc ggacatggta ttacatggga gtgtgagcac gcacagacag

162061 ggacctgggt tgagacctgt agaaggaaga ggggcttccc actccaatat atcccagggc

162121 gtcactccat ctgtgcgtct ccgagtgtgt cagtgcatcc agggcagagt tgggtcaacc

162181 cctatgccca ccctctcctt agcaagggac caggttgaac atgtgcgact aggagagtgg

162241 aggggatggt gacagaacag ggtgctccca ggaggtccca aatgacaaag agtcaatgaa

162301 ttcacccttc agtgtgttgt actgatgaca gtgttgacat gtcagagtga aaatgccagc

162361 tgacccggaa gacaggggaa tgaggtgtca tgtgggaacc ctgggtctag acagcaggga

162421 accccaaagg gacagccgtg cacgggtttt gtttaagaag gagtgtagaa ctatttacac

162481 cagtcgccca agtgcgctct cacatacatc acgcatccct gagttcaagg ggcctgagag

162541 aggcagtgat gtcactgtgg gaacggcccc gaggtgacaa gacatgttcc ctcctcctct

162601 gctcctgctc acaaggacct gacctagggt acctcccact cctgacctga ccctgccatg

162661 ggcaccaggc tcctctgctg ggtgaccctc tgtctcctgg gggccggtga gtcctcagaa

162721 accaggcagc ctcagtgtgt gtgtgtgtga caattacagt tgttttcctc cttctgttgt

162781 caacttctgt gttccaggtc acacagaggc tggagtctct cagttcccca ggcacaaggt

162841 caccaagagg ggacagaatg tgacatttca gtgtgatcca atttctggac acactgttct

162901 ttactggtac cgacagacac tggggcaggg tccggagttt ttggtgtact tccaaggcaa

162961 ggaccctgtg gacacctccg tgatgcctaa ggatcggttc ttcattgtga gacctgacgg

163021 ctcctcctcc actctgagca tccagcctgc agagcctggg gactcggctg tgtacctctg

163081 tgccagcagt gcaaccacag tgtggcaccg tccccaactg cctgctcaca aaccctcctg

163141 tgctcccctc tgctcatggt tctgagactc ctcagcaaag gagttgccct gttcttcctt

163201 ttccaagaag cataagtgca ttgggatttt caatgctttt tctggcagta atgactagaa

163261 cacggcatgc tggatttcta gggttccttc atgtacctca agattctgta attgaggata

163321 agccccggag ctctgcctca ggcatggtga aatcttctct tgtgaaatag aaagtttcag

163381 cagatgtgtg acaacctccg agtcctgagt gtcactgatg atgacacgtg atgcccacat

163441 gtgagcagcc atgcaagctc tgttaggcca caggcagcca ccagccttaa aaatcaatag

163501 ttatactcct tagtgaaaac tgtcattgga atcattcaca gacaccggtc atgcacaata

163561 atgcacatta atatatatcc atatgttgtt ttattatttt tcttcccaat cctgtagtca

163621 ttccatattt aatttccttc ttctctagcc tatttttaat agaattttaa atttagcttg

163681 gattaataaa aaccacattt gagaagtttt aaagggaagt cattcaaata aaatcaaaat

163741 aatagttgta ctgatctccc cttattttgg agggatgctt tccaagactc agtggatgcc

163801 tgaaaccgct gatggtttga tggaacccga cgcttactgg tctccacccc ccacccgaaa

163861 cacgcacctt tcactttgaa ggaagcactt tatggtatct ctttggcaga tctggttgct

163921 agcatcatgg ccctgcattt cacggccatc actaagtaca ataaggggga ctggacctca

163981 ggcactgccc gctcacagca gtcagtctga tgacagagac ggtccaggga gtgacaggca

164041 gggacagagc acagtgtgga gacgctgggc aaggggctga ttcacatcct gggtgggaca

164101 gagggggaca gtgtgagatg tcaccacact actgagaatg acgcatgttt aaaactgtta

164161 aatacttttt ctctggaatt tatcatttaa tattttggga ctacagttga acgtaggaaa

164221 ctgaatctgt ggaaagcaaa actacagcta aggggggact tctgtggata aaagtaaaat

164281 gtgagacaac aatagtgaca cagggagcag gtaatgcata catgtcttac cgttgtgaga

164341 tcttcctgat cgatgtggag gggtgtaggc tgtgataaag ctgagaggtc cattgcaaac

164401 tctagagcca ccactaaaat agcaaacaga gttatagctc ataggccaac aaacccggaa

164461 atgaaactat tataaaaatg cctaattaat acaaaagaaa gcatgaaaga aaggacaaag

164521 gaacaaagaa cagatatact aatagaaaac aaacagcaaa atgacagaca tgaaggtaac

164581 cagaacaata tgtatatcaa ctctagtcta aatgccccaa ttcaaaagca tcgattgtga

164641 tcatggataa gagagaaagg aagcagatgt gtgccacctg ccacagcggc ctgcaccagc

164701 agcagaagca ctcagaggtg caacggagca gacacaccac gctgacactg atcacatgaa

164761 agctggagca gcaacgtttg tatcagacaa agcaaatctc aaagcaggga ctatcatctg

164821 agacaacaga ggtcatattc taatgatgaa agggccaatt gatcaagaag aaaaaagcat

164881 tctgaaatat ttatgcatct gaaagcagag ttttaaaatg ctgaagcaaa aaaggcaagg

164941 agaaagagac aaattcacaa ttatagtcag acatttcatc acctgtcagt aattgcctga

165001 acaacttgac aaaagggaaa aatagtgagg gtactgaagt catagagaac atgatcaatc

165061 accttgacgt catgcacatt tacgggattg ttcacccaac agcagcagaa tgcaccttct

165121 taccaaaggc acacggacta tgcaacagga taaactgtgg actttgacca taaaataagt

165181 cacaatacat tgaaaacaat tgaagtctta caaaccatgt tctctgaaca cagaggagct

165241 gattagaaat caataaccga aagaggtcta gaaaaatccc aaatattaga aactaaataa

165301 caatattcta aataacccat agggcaaaga acaaatcgaa agtaaatgat aaagggattc

165361 taactactga aaatgagaat gtaacatata aaaatgagta caatgccaca ctaggaatac

165421 ttagggagaa acctgaaaac aagggaataa ttcccttctc atcccgagcc agcactattt

165481 tacccaaatc aaaaccaggt atcacccaaa caaaacaaaa caagaaacca aaaaattaaa

165541 aagccgacag ggaatatccc tcatgagtac aaactaataa acctttggaa tgccagtgca

165601 tccaacatga cattgaaaag gataacacag cacgaccaag tggggtctgt ctcagcatta

165661 catcctcatg actgttaaca tttaaaagga tgtccaggaa ggttaacatc tcaaatgcta

165721 aaatgtgaga aatcatatga ttatctctat agatgcttaa aaataccttg atgtattcca

165781 acatctgttt ggataaaaac tcttcttaaa ttagaagtaa ttgggaacac ccttccccta

165841 atccagggca tctgtgaaaa atctggggtt agtaacagtt ctggagggag accgtggtga

165901 ggtggactgt ggtaaagcaa agatgcatgc acactgccat ccacagaccc aacactaaga

165961 gtagcaggga agggggggat agtcatttca acagtagtgc cgaacaaccc ggatccatag

166021 agaaaaataa tgaatctcaa tgcttacctc actccataaa gcagaagggg gccaaacttg

166081 gcccctccag gttcttctag ggcccagaag ataactattg gggtttatat aagtggttgg

166141 aaaacattat attttgtgac atacaaaact acgatttcaa tgtccacaca cgaagttaag

166201 ggaacatgac cccactcagt aaggtaccta gtgtgtctgg ctgacctggc acagcagtgg

166261 cagagctgag taggtgggac agaaaacaga acatattcac cacctgcctc tttacacagt

166321 gattttgctg actcagccat gcacagaatg aaattgaggt gagctaacat gacagatcta

166381 ttaagcttct gaaagaaagc atagcacatt ccctttatga ccctggagta ggcaaagatt

166441 gcttagagag gacaaaaact attgtaatgc ttacaatcac atattttgaa ttttggccaa

166501 gtcggaaatg taggaagaaa tgctccactt cctcacataa tctgtcaacc aaggagttaa

166561 agaagaactc tttatccaga gtggtaggaa gggtggagac gggctgccca gaggagaggc

166621 tgtggggcca ggcgatggtg tgggcagtcc cacattccca cccagatgag ctgggaagaa

166681 cgtatgggga gcgagacaga caccaaaacc aagggtttct tttattatta ttattattat

166741 tattatttct tttttacttt taatcattgt ttaagtacag ttttctccct tttactccca

166801 atccagccca ttcacccaac ccttcccact tccttcccat accaccctcc ccctagtttt

166861 tgtccatgtg tcctttatat ttgttcctgt aaacccttcc cattctcccc tgaaattccc

166921 tcctctctcc cctctggtca ctgtcagcct gtcctctctt tcagtgtctt tggttatatt

166981 ttgcttgttt ctttgttttg ttgtttaggt tcctgttaaa ggtgagatca tatggtattt

167041 gtctttcact acctggctta tttcgcttag cataatgctc tccagttgca tccacgctgt

167101 tgcaaagggt aggagctcct tctttctttc tgctgcatag aattccattg tgtaaatgta

167161 ccatagcttt ttgatccact catttactga tgggcatcta ggttgcttcc agcacctagc

167221 tcttgtaaat tgtgctgcca tgaacattgg ggtgcatatg ttcttttgta ttggtgtttt

167281 agtgttctta ggatatagtc ccagcagtgg gattgctggg ttgaaaggca gatccatttt

167341 cagttttctg aggaagttcc aaactgcttt ccatagtggt tgtaccagtc tgcagtccca

167401 ccaacagtgc acgagggtcc ccttttctcc aaacctctcc aacacttgtt gttgttgctt

167461 tgtttatgat ggccattctg actggtgtga agtggtatct cattgtggtt ttaatttgca

167521 tctctctgat agctagcgat atggaacatt gtctcatgtg tctttggatt ttctgtgtgt

167581 cctccttgga gaagtgtctg ttcaagtcct ttgcccattt ttaattgggt tgcttgtctt

167641 cttagagtgg agtcatgtga gttctttata cactttggag attaaaccct tgtctgaggt

167701 atcattggca agtatgtttt ctcatacagt tggttctctt tttattttga tactgtcttc

167761 tttagccatg cagaagcttt ttattttcat gagatcccat atgtttattc tttcctttac

167821 gtcccttgct ctagggtaga tgtcagtaaa aagtttctgc atgaaatgtc tgagattttc

167881 ctacctacga gcttctctag gacgttaatg gtgtcacggt gtacatttaa gtcttttatc

167941 cactttgaat ttatttttgt gtaaggtgta agttggtggc cgagtttcat tttttttttt

168001 gcacgtagct gtccagttct cccaacacca tttgttgaag aggctatttt tactcctttt

168061 tatattgctg cctcctttgt caaatattaa ctgaccgtga agacttgggt ttatttctga

168121 gctctctgtt ctgttccact gatccatgtg cctgttttta tgccagtacc aggctgtttt

168181 gattatactg gccttgtaat atagtttaga gtcaggtatt gtgatccctc ctactttact

168241 cttttttctc aaaattgcag cagctattcg gggtcattta tgattccata taaattctgg

168301 aagtgtttgt tctatgtctg agaaataagc cattggtact ttaataagtt ttgcattgaa

168361 tgtgtaaatg ctttgggtag tatggacatt ttgatgatat taattcttcc aatccatgaa

168421 cacggtatat gtttccattt gtttgtgtct tccttgattt ctttcctcag tgttatgtaa

168481 aaaccgagcg tttcatcaca gaaagctgaa gactcaaaac ctctggctgt aaaaatctgt

168541 gggggatgca gtggtgggag aaactcccag tctcacagga gagtttgttg gagggcccag

168601 ggggtcctag aatgtataca aacccacaca cctgggaatc agcacctacc taaaaatgca

168661 caattagctt gtgggaaatg agagagaagt gacagaaagg ctgtgagaac cgagcaagtg

168721 acattgttcc ctcagggacc cctgccccac atacagcctc acaacacact gaagatggtt

168781 gcctcacctt ggtgaatacc taaggttctg ctccttacta cagaacaggt gtgcagaaac

168841 taagaaatat tgcccaaatg aaagaacaga ccaaaactcc agaaaaaaga ctaagcgaca

168901 atgagataga caacttttca gatgcagagt ggaaaacact ggtaatcagg atgctcatag

168961 aattgaagag ctcagttgca aaatggggga agaagtgagg gctacccaga ataaaataaa

169021 gcaaaatata cagggaacca acagtgaaaa gtaggaaacc aggactcaaa tcaatgcttt

169081 gggaaaacaa aaagatataa acacctgact ggaacagaat gaagaaagag cttcagaaaa

169141 atgaggagag gcttaggcac ctctggcacc actttaaatc ttccaacatc caaatcatag

169201 ggctgccaga aggagaagag gaagagcaag gaattgaaaa cttatttgaa aatatcatga

169261 agtgaaaccc ccagtctggt gaaggaaata gacttccaag tagtccagga agctcagaga

169321 gtctcaaaga aattggaccc aaggagcaca caccaaggca cattataatt atgttaccca

169381 agagtaaagg taaggagaga atcttaaaag cagcaagagg aaaggaaaga gtgacctaca

169441 aaggatttcc ctttagacta tcagctgatt tatcacaaga aaccttgcag gcaagaaggg

169501 ctggaaagaa gtatttgaag tcatgcaagg aaaggaccta aatccaagat gactctaccc

169561 agcaaagcta tcatttacaa tgaaatggca gagaaagctc ttcccagata aggtcaagtt

169621 aaaggagttc atcatcacca aacccttatt atatgaaaag ttaaaagtac atatgtaaga

169681 aatagaggaa gatcaaaaac taggaacagt aaaatgacaa gaaacacaca actatcaaca

169741 aatgaaccta aaaaaaaaaa aaaaactaag caaacaataa gaaaggaaca ggatcacaca

169801 aatggagatc acctggagtg tgctcagtgg ggaagatgag ggaggagaat gggggtagag

169861 gtacagggaa tgagaagcat aactggtagg cacaaagtag acagggggag gttaagaata

169921 gtgtaggaaa cagagaagcc aaagaacttc tatgtacaac ccatggacat gaactaaggg

169981 tgggggaatg atggagggtg cgaggataca gggaagatga ggagaaagtg gagaagatgg

170041 ggacacctgc aatggcataa ttaataaaat ataccttaaa aataaataag atatctaagg

170101 ttaaaaacat acacattatc ctttatgaaa attttaaaaa tcctgttcat caaaagatat

170161 taagaacata taggtatata tctgaaaaga gcatgtattc aaagtataaa caattcctag

170221 aaatcaataa ttaaagaaga caaatcaatt tttttaaaaa tatgacaagt tgaagatggt

170281 gctctcaaaa accaaaagtg acatgtaagt agaccatttc ttagttccgt cttcacagag

170341 ggtttctttc agcgctgagg agactttttg gcttatggga gcccagcgta caccctgccc

170401 aggaattgct gggtgaagac agagctttgc tgagggtcct gccttccctt ccctgggcag

170461 ctgcatgcat ggcgtcctct ttccatgatt attcactgat caggttgata acttttattc

170521 ccgtttggaa aaggcatcac acctccctca ccaccacagg caaatagtga gcctgagaaa

170581 ggtaaataag cagcaggagc tgtgcagagt ccttggggac ccctgatgac taatttagac

170641 agaacccaga ctgggggttc cctgatgact gaggcagacc ccagccagct ccagcagggt

170701 ccatagcgct gctgcacaag tggccacttg agggcgctgt ggttccacgg agtaagatgc

170761 tgatggcacc ctgtgggaga tggagggaga ttcatgggaa gaatcttggc ttggttcctg

170821 caagaagcag attatctgat gaatctgagg ttcactgatc ttatcttctc acattaactg

170881 acaaccacgt gggtatgtta tagcagggag ccatgaagta ggccttacat ggagttaagt

170941 ttctcctgga gcctgtgttt ggtggagagt gtgatggggc ttcagggagg agcctgggta

171001 acctgagcca gtgtagagca aggagcacct gctcaaggca gagagaacct gtgctgatgg

171061 ccacctggcc tgtaccctcg gggcccaact cagcacagcc agctgcacct gctcgtaggt

171121 gatggacaaa ctcagggaca caggaccagc tgagcctgtc caggaggtgt gggtgcccat

171181 gagatgctac aaagacagtg acatcagaac agtgacgtca caggcaagcc ttccaaagag

171241 agcaaaggga gggagggaca agcctctgct gtctcacccc aaagaccaga gccctgagca

171301 gagacatggc ccgtctggtc ctgcctgaat catccagggg cacccagctt ctctgttgca

171361 tggtcctgtg tctggtggga ccaggtgagt ctcagctgtg gtgggacgtc cctccaaccc

171421 agcaccctgg actcttgctg gagtgtccat ctgcctcctg ggatggtctc agctccattt

171481 ccttcctcca cagggtcagt ggctgctgga gtcacccagt ccccaagaca cctcatcaaa

171541 cgccgtggtg gggaggctgt tctgaaatgc caccccatct ctggacacaa ccgtgtgttc

171601 tggtaccagc aggattgggg gcaggaaccc aggtttctca ttgagtatta tgaaaagcgc

171661 gaggtcagta aaggaaactt cccagaccgc ttcaaaggaa agcagctcag agactacagc

171721 tccgagctgg ccatgcacac cttgcagctg ggggactcgg ccgtgtacct ctgtgccagc

171781 agcttagaca cagccctgca gggcccatgc ctttctgtac ccaaaccctc ctgtcccagc

171841 tgatgtcaca ggcagaggga gggggcctca gcccagttcc acagatggtt ttcattgttt

171901 tctgccacac ccctgccctg ctgagctcag ctagagcctt cctgaatgtt ctcccagctc

171961 tgccaccagt gtgcaattcc cagcacatgg caggaagccc ttctctgtga cagagtggag

172021 tccacactgt gtgttgtggt gcccggtcct ggtgaacacc gtgtgtccac ttgcagagta

172081 gggagcactc agtgcaccat gtcatccaca gggagctgta gcttgtcaat gttccaaacg

172141 tcccatgtac gtccatcctc tatctccacg cctctgaggt caacaagacc ctcaattcca

172201 tcggccctcc agagccttgg cccataggat tgaaaacagc acaacctctc ccctttcaag

172261 agcatctgag ctgggatggg gaggtcactc ctcaggagtg gggtacatgg ctgtttgcct

172321 gccattttct aggacggaga gcttcaagac agaggacatg tgtctgtcct gtcatagttc

172381 cagacagagc gaggccatga tatgctccta acagaggagc tcagtgaccc agggaatgtc

172441 cagtccatca ggagatccta ggagggaagc agaagccctc tgggagtgtc aaggagcatt

172501 ctgggagctc atgcggggaa tgcgagtttc tgaactactg gacagggcgg ggatagggtt

172561 tctcagcctg tcctcagctc caccacttct gatcaggaaa tccgaatttg ctacatctgc

172621 ccaggtgagg agctgcacac aacagctgag tgtcacatcc ccctggcctc tgatttttga

172681 ctccatgtcc tttctcactg ctgggtggtt gttgttgccc accaaggtga catcaagata

172741 cacagtcaca gacaaatcgc gtcacactga ggtatgacct gtgtaagagg tagcagcact

172801 gggaccgctg gctgaatatc ccatccacgt cactgacgag gctcctctct ttgtcacaga

172861 aagcacagtc atgggccaca ggtgtgtagt cccccgcggg gcctcagctg gaaccagggg

172921 agactcttac ccgtgagcat gcagagacct gagctcgggt ggctgggctc ccaccggtgg

172981 gagaatcatg agacaatgta aagtagttct cagattccaa tggccttgac cccaaggctg

173041 tgaaaactct ctggatatgg gtttgttccc ttaagggaga gggtccctgt gggctgacat

173101 tgaaattatc tagagtttat gcaaagagaa aagctaggag aatgtatttg aagtgaactt

173161 tgatcagaga agtcaccacc agcatgagca ttgctatggg aacatttcct agtctacagt

173221 aatgggagga aaacctgtac aggtaaggaa ttgtatcact gtgcatagaa gaaggaattt

173281 ccttaccaga ctcaggggaa agcatgcgta ctgctaagat ttcttttaaa aagtgactta

173341 cacatttttc ccatgctgca ctgctcagct gtctctatgt cactggtttg ttcctgtggg

173401 ggaggggagg caggtgagct ctgtagctga agcatctgtg acggaccatg atgtcaccga

173461 ggccctgaga ggctcacttg tgtttcccca gacagaactg gaagacatga cactgtgcgc

173521 tggacctgaa atggtcagca gggtctgctt ccgtgtggct ctttgtctcc tgtgggcagg

173581 tgggtgcagg tgggcttcct gccctggaat tcccagcctt cagtcccagg cttggtggta

173641 ggatgacaac ataaaggtct gtcactttct ccacaggaca cttggatgca ggaatcaccc

173701 agagtccaag gtacaaggtc acagggacag gacaaaaggt gacactgagg tgtcaccaga

173761 ctgataacca taacgctatg tcctggcatc gacaaaacct gggccatggg ctgaggcaga

173821 tatattactc cgggggtgtt gggttctcca gcaaaggaga ggatgctgat gggtacagtg

173881 cgtccagagt gaacatggag gacttcctgc tcacgctgga gttggccacc ccctcccaga

173941 catctgtgta cttctgtgcc agcagaaaag tggtgatcca cagcgctgca cggccacctt

174001 ctctctgcac aaaaatgatt gttgcggccc tgcactcagg attcatggga tccctgagca

174061 aactcctgca cctgagagcc ctgaggcacc tgcctacagg gggaccctgg gtgtgtgatg

174121 aggtccccct ctaggttagt gtctaccagg cctggtctag gcccagggcc tcacacacag

174181 tctgcactgt cactgtctgt cagtctgtcc tgtcaggtgc tctgttagct gggaaggtgt

174241 ttgctctttc tagcttttct tgtcatcagc ctgagtgtga tgcctccagg gcagggagtc

174301 atgaggagca tatgcacaga gatccccgtg agtgtcacgt gcaggtgtgt tgaaggtgca

174361 ggacactggg tcttcctcca gacggaagct ataagaaaca tggtttttag tgctggtgac

174421 taaggaggac acggggcttt gctgtgggtc ggggagacac cttatactac atggaggtta

174481 atgagcagag agagggcact aggtcgagcc ctgcagaagg agttggagct tccaactcag

174541 atatttcctg gggcatcatt ccgggtgtct ccaagcaagt gggcccaggg agtggtcctc

174601 aggtgtgatc cggtttatgg atacttgaca ttgacatgtg ctgacaggtc ctgggacagg

174661 gcccagagtt tctgatggac tttcacctcc tggatcccca gactcctgcg ggacccctaa

174721 ggactggttc tctgctgaga gaccaggggg atcccactgc actctgagga tccagcctgc

174781 agagccgggg gactcggccg tgtccctctg tgccagcagc tccagcacag ggtgaccccg

174841 tcaccccttc atgcgcacac accctcatcc ttctccctcc atgcagcttc cgcactctaa

174901 gcagcccttc tttgctcatc gttccccagt gacgatgcag ttctgggcat cagctgtgct

174961 ttgggcagac agaacaaagc cctgcccggt gagcctcagt tggctgggcg tagtactgcc

175021 aaccaaaggc ttgctggttg gattcccggt cagggcacat atctggattg cagaactggt

175081 accctgttga ggcacataca agaggtgtcc tatgcatatc tctctcccac atcaatgttt

175141 gtctccttct ctttcacatt ccctcccatc tctctggaag taaataaata acataattaa

175201 ataatgaggg ttcccagacc cagctcagtg ctccaaggca cagactgaac atcgtggcca

175261 caaatagtca tcggtaggac tgtgaggctc ggtccccaac acagctccgc tttgcagggc

175321 agggggagtg tgcccgctcc tcagggcttg tccgcatctg gaatgctgac aacaccctct

175381 tcctgcgcac ccttctctga gccgccctgt caccttgggg gctccgtagc aacttctcta

175441 acgtgccttc ctgttgggag tgaaggtctt cacagcttct gtgtctttga ctgagaatca

175501 ccatagtgat accagaaagg cgtcatgtcc tctgccaggc taaaggagcc cacactctat

175561 tgtgttttct ttttccctcc gctccatcct gagtgtcagg ctgcagcgtg tgtccacgac

175621 ctcttggttt catcttgacc ctatgggtca tcctttctag agatgagggt ttttcttggt

175681 gtcctctcat tttgacttga agtaatttcc ctgaattctt ttaactcaat gatgtgacat

175741 tttctagata atatgcctta cttcttttta tttattttat gtgttatttt gggatatatt

175801 atctgctaca aatataaata atcgagcccc tgtgtgtggg ggcagcggca gacatctggc

175861 aaatccccgc ccatgggcag aggggacagg cctttggatc atgggcttct gggagtcctc

175921 agttctgagg tgggactgga tgtcacagac tcagtcctgg tccaggagcc tgaccttgca

175981 cagcaggtaa ggacggtcac agtctctgaa aacccaccta aacactgtgg tagccatgac

176041 accagacctg ctcacccagg aactagagtc cttgggggga cgtttcctgg tcgtgtgtgg

176101 actgtgctca ggtccctggg cctccttgga gggaggaagg cctgggttcc cagggcagct

176161 gcctgtcctt gggaagccat cagggacccc ttcccagact gtgtccccat ttctgtcact

176221 gcccagcacc tcaccccgga cgactctgct tctttaggac actgttttgt acccgtgttt

176281 gcttgtctcc cacaacccgc cactttaccc cgaatcatct ccgtgctcac agagcaggtg

176341 cccactgtgc gggctgctcc tcccgtgtgg gctgtgtgtg ggggcctcag tcccagcgtc

176401 ctcagctccc cattggatgt ctattctcca caactgctca ccaaccgtat gggttacatt

176461 tcacttgtgc ttcaatttta acatttattc acacacattt gactccaatc ttgaaggtaa

176521 tatattatct tcgcagtctt ttgcttatct acattttgga ggtaactcaa tacacctttt

176581 ttcttttttt gcattatgtt tattttgagc aatggagata aaagaaatgc tttaaccttc

176641 aacttgtgtg ttttccttcc tacttggact agatattact tccccccatt cccctcctct

176701 ccctctgcct ttcagaggac aatgtgaggg aagccggtcc cagactgtag ctgcagatga

176761 tgtgagagtg ctccagggga acggggtggg gtgttaagca tggaagctga aagaaatagg

176821 gtcatggtgg gtaataaagt tgggattagg gttaagggtt ttgagatacc atataaagga

176881 tagtgcttct ggaatcctct gcctgtagac caggcagaca agcccaccat cccttgggct

176941 gtggcagaag acaggataat gaaatgtcag ttactggcca ccagatggcg ctgtgacacc

177001 actgacctgg aaggggctct ggggagagtc tgggagagca ggaaagggtt aagaagccct

177061 gggcagggac ccaacacagg gcagaagctg aggcaggttt cagctgtggg cagcctccct

177121 gcagctgtgg agtgggtggg aggtcccgct aaaccttaaa atatccatgg ttgaaggact

177181 tgggcaaggc agccctgctg gcaggggcag gtgcagagag acaattaact gtcccctagc

177241 actgtggggg accagaagtg gcccaggtta ggacagtgaa gggacctcag ggctcatgtg

177301 tgcaggggga agcagctgga gtcactgagg ggtcctagga cgccccgagc tggcacaggc

177361 tcctcctgcc caaggagagg ggacctgcct gcacctcagg aggcaccaag gaggcaactc

177421 cttcagcccg aagagcaaga agggatgtag ggccagcagc aagagctggt acttagttca

177481 ggcaggggca ggcagggaca atgttgaggt acttagggat acagggaagt tgcagtgaac

177541 ccttagccac ggcgctgggc agcacaggga gacgtgaata gaatctggac cctgggtcct

177601 accgaggata cagggcacca tgggcagcct cctcggagag tcccatcaga gcagtcacag

177661 catcagacca atgacgtcat agcaagtgct cctatcagca cacagggagc acagaactgt

177721 agaccactca ccccagggga ccccgcccag agctggaaga ggtcccgggc agctgccatg

177781 ggccccaggc tcctctgctg tgtggccctt tgtctcctgg gagcaggtga gccctgggca

177841 ctgtgtgtaa gtctggacac gcagtcacat ccctgacaca caagcctggg ggtggtggct

177901 gcctcgggag gctccctgtg ctctgccccc agctcctctg tgtctcctcc caacaggccc

177961 agtggactct ggagtcacac agacccccaa acacatggtc acagcaagaa aacagcaagt

178021 gacgctgggg tgttcctaca tctctggaca cctctctgtg tattggtaca aacaggttca

178081 gggtcagggc cctgagttcc tggttgagta ctacaatggg gaagagggag acaaagggaa

178141 cctgcctgat cggctctccg tgaagccgct ggatggctac cgctccaagc tgaccgcgag

178201 ctcgctggag ctgagcgact cggccctgta tctctgtgcc agcagccctg acacagccct

178261 gcaggcgcgg cagcctcctg cacagaaaca ctccgccccg ctcaggaagc ggcagctgcc

178321 agccacacag gctcaggcct gagagagcag ctcagcttcc ccaggcccgg ttctgctgct

178381 cgcagatggg gtgggattgt tgcccacaga catcatggca gcctaggtgt cattgtcctg

178441 catatacacc tggtgggaag tcctgttcct gggtgtcaga tttcataacc caaagagcca

178501 gaaatccctc aagtctgccc ctccacacac atggatgtgc acgactgccc ctcccccgct

178561 cccagcgctg ggcccacatt ggctgccctt ttccgggttt agatgaggga gcctggggag

178621 atcacacacc atcggggatt gttgatctct tccctgacag cttcaagaac atttcacttc

178681 aagggaacga atatccatgc ttccctcatg atgacagcaa acacaggaac aatttctttt

178741 attttttaaa gattttattt atttatttta gaacggagga gagggagaaa ggagagaaac

178801 atcattgtgt gtttgcctct tgtgcgcccc ctactgggga cctggtctgc aacccaggca

178861 tgtgccctag tctgggaatt gaaccagcaa atgagcaagg cacaatttcc tgcactgtcc

178921 ccgtcacctg aacctgcaca gagccctgaa ggaagttggg gatcctgcaa agcatgggac

178981 tgtggggaga ttagctacag gtgtttaaag acccgttgta ttcattataa ttaatgtcag

179041 cattccttca catggagaca ttccctgggg aacaatgcct caacttaaaa agagcatttt

179101 tcactctctc cctgttggca gccgcctctc cccacaccac tctgtaaccc gaggggccag

179161 cagggcacct gcagagctga gcaccccaca gagagggagt gtccccctgg actggatgtg

179221 ctggccgctg cctcaccacg aacatgggaa gcacattctc aggtgactct ggaagtcctg

179281 gggagcacgt ggcaggggtc ccatcggtcg ggggatggag ataaaacaga gtcaaaggat

179341 cctcaagtgt tgacaagcgt gtccctgagg tttggccacc agagaccagg agggccctgt

179401 gggaggaggc acagtgagcg gatgctggac gaccagcaaa gtagaaaagg aggcggaggg

179461 acgacttggg ccccagagcc ctaaaggcat cagaacgccc ccaggggttg ccctcagacg

179521 caagagcaca gggacgggga cacacagcag accagggcgg catgaggatc tgcgggcagg

179581 acgaagcaca cctgagtcac acactgtgaa cacggagggt gaggccgact ctgggactcc

179641 cgtcagttca gccacacgca ttcttctccc tctgagctgt gcccctgccc gtctgcttct

179701 gatctgacgc tctggggaag gggtgtgtcc ccgtgtgaca agctggctct ggggtccagg

179761 aagtgggagt gacacctcag agagactccc tggacagccc tgctcctgtg cagccaccca

179821 cagacccaga gggaccctga gtcagcccgg ctgctgccat gaaccgcagt ctcttgtgct

179881 gcgtgacctt gtgtctcctc cgtgcaggtg ggtcctgggc tgggcccctc tgtggggaca

179941 gtgccaaggc ccagcaccga gccgctccct ggaggctgca gcatctgctc ccctgtcctc

180001 cccgcaggtc tggggatggc cggggtcact cagaccccca cattccaggt tgtgacaaca

180061 ggacagacgg tgaccttgag gtgttcccag gacttgaacc acaacgctat gtactggtac

180121 cgacaggacc tcggtcacgg gctgaagctg atccattatt cagcgggtgt tgggttcaag

180181 gacaaaggag aggtccccga tgggtacagc gtctctagat caaaaaaaga agacttcccc

180241 ctcacgctgg agtcggccac ccccgcccag acatctgtgt acttctgtgc cagcagtgaa

180301 cccacagtgc tgcacggccg cctcctctct gcacagaaag tcagggggag ggccagtgcc

180361 cagactcagg ggagccctgt gcagactcct ccaccttgga gcccccagtg gcccctgcct

180421 acagggtgac cctctgtata tgtagggtga ccttctgtac ctgtggggtc atcctctagg

180481 ccagtgtctg ccagcctgca accaaaccca gggccttgga cacaaaagtg tccacttggc

180541 tggctgcctg tctgtcctct gcagctgctc cattggctga gatgatgctt ccccagctct

180601 tgcttttctt ttcagcaacc tcagtcacag gccatcaggg tgggctgtca tcagtaacca

180661 gacacgtctg tgcccactag tgtatcacac gcaggcgtgt cacaggtcag tcacggtgag

180721 tctccttctg gaaagcagcc atgagaaacc cgtgtttaca tgcagggcgg cttagaagca

180781 caagaggctt ctctgtgggc cgtgcggaca tggtattaca tgggagtgtg agcacagaca

180841 gacagggacc tgggttgaga cctgtagaag gaagaggggc ttcccactcc aatacatccc

180901 agggcgtcgc tccacctgtg cgtctctgag tgtgtcagtg catccagggc agagccgggt

180961 caacccctgt gcccaccctc tccttagcaa gggaccaggt tgagcatgtg caactgggag

181021 agtggagagg atggtgacag aacagggtgc tgccaggagg tcccaaatga caaagagtca

181081 atgaattgac ccttcggtgt gttgtactga tgacactatt gacatgtcag aatgaaaatg

181141 ccagctgacc tggaagacag gggaatgagg tgtcacgtgg gaaccctggg tctagacagc

181201 agggacccta aaaaggacag ccgtgcatgg gttatgttgt aaaaggagtg tagaactatt

181261 tacaccagtc acccaagtgt gctctcacat acatcacaca ttcctcagtt caaggggcct

181321 gagagaggca gtgatgtcac tgcggcagcc gccctgtggg gacaagggat gtccctcctc

181381 ctctgctcct gctcacaagg acctgacccg gggaacctcc cactcctgac ctgaccctgc

181441 catggacacc aggctcctct gctgggtgac cctctgtctc ctggaggccg gtgagtcctc

181501 agaaaaccag gcagctccag tgtgtgtgtg tgtgacgatt ccatttgttt tcctccttct

181561 gttgtcaact tctgtgttcc aggtcacaca gaggctggag tctcccagtc ccccaggcac

181621 aaggtcacca agaggggaca gaatgtgaca tttcagtgtg atccaatttc tggacacact

181681 gttctttact ggtaccgaca gacactgggg cagggcccgg agctgttggt gtacttccaa

181741 ggcaaggaac ctgtggacac ctcggtgatg cctaaggatc ggttcttcat tgtgagacct

181801 gacggctcct cctccactct gagcatccag cctgcagagc ctggggactc ggccgtgtac

181861 ctctgtgcca gcagtgcaac cacagtgtgg caccgtaccc aactgcctgc tcacacaccc

181921 tcctgtgccc ccctctgctc atggttcgga ctgcccagca aaggagttgc cctgttcttc

181981 ctttctcaag gagcatacgt gcatttggat gttcaatgct ttttctggca gtaatgacta

182041 gaacatggca tgctggattt ctggggttcc ttcatgtacc tcaacattct gtaattgagg

182101 ataagcccca gagctctgcc tcagctgtgg caaaatcttc tcttgtgaaa tagaaagttt

182161 cagcagatgt gtgacaacct ccgagtcctg agtgtcactg atgatgacac gtgatgccca

182221 catgtgagca gccatgcaga gctctgttag gccacacgca gccaccagcc ttaaaaatca

182281 atagttacac tccttagtga caactgtcat tggaatcatt cacagacacc ggtcatgcac

182341 attaatggac attgatgcat gcccatatgt tgttttatta tttttcttga caatcctgca

182401 gtcattctac attttatttc cttcttctct agcatatttt gaatagaatt taaatttatc

182461 ttggattaat aaaaacccta cttgagaacg ttttaaaggg aagtcattca aataagaccc

182521 aaataatagt tgtaatgatc tcctcttatt ttggggggat gctttccaag acccagtgga

182581 tgcctgaaac tcctgattgg ttgatggaac ccgacgttga ctgttttccc cccacacacg

182641 cacctttcac tttgaaggaa gcactttctg acatctcttt ggcagatctg gttgctcgca

182701 tcacggccct gcatgtcatg gccatcacta agtacaataa gggtgactgg acctcgggca

182761 ctgcccgccc acagcagtca gtctgatgac agagacggcc cagggagtga tgggcaggga

182821 cagagcatag ggtggagacg ctgggcaagg ggctgatcca catcctgggt ggcacagagc

182881 aggacactat gagatgtcac cacactactc agaatgacac acggttaaaa ctgttaaatt

182941 ctttttttcc tggaatttat catttaatat tttgggacta cagttgacct taggaaactg

183001 catctgtgga aagtgaaact gcacctaagg ggggactact gtgggtaaaa gtaaaatttg

183061 cgacaacaat agcaagacag ggagccagta atgcataggt gtcttaccgt tgtgagagtc

183121 tactgatcta cgtggagggg tgtaggctgt gataaagccg aggggtccat tgcaaactct

183181 agagccacca tgaaaatagc agagttgtat aactcatagg ccaacaaaca cagaaatgga

183241 actcttataa aaatgcctaa ttagtacaca agaaagcatg acacaaaaaa caagggaaca

183301 aagaacagat atgctaatag aaaacaaaca gcaaaatgac agacatgaag ggaaccagaa

183361 caatatgtac atcaattcca aatagtctaa atgcctcaat tccaaagcat cgattgtgat

183421 catggataaa acacaaaaaa gcagatgtgt gccacctgcc acagcagcct gcaccagcag

183481 cagaagggct cacaggtgca ctggagcaga cacaccacac tgacactgat cacgtgaaag

183541 ctggagcagc aatgttagta tcagacaaag caaatctcaa agcaaagact atcatctgag

183601 acaacaaagg tcatattcta atgataagaa ggtcaactga tcaagaagac aaggcaatca

183661 gatacactta tgcatctaat aacagagttt tgaaatgctg aagccaaaaa ggcaagtaga

183721 aagagacaaa ttcacaatta tagtcagaca tttcatcacc tgtcagtaat tgattgaaca

183781 acttgaccaa aaaagaaaaa tagtggggtt actgaagcca tagaaaacat tatcgatcac

183841 cttgacctca tgcacattta cgggatagtt cacccaacag cagcagaagg caccttcttt

183901 ccaaaggaac acggactata caacaggata aactgtggac tttgaccata aaataagtaa

183961 caatacattg aaaagaattg aagtcatata caccatgttc tctgaacaca gaggaggtga

184021 attagaaatc aataaccaaa agaagtctag aaaaatccca aattttagaa actaaataac

184081 aatattctaa ataacccata gggcaaagaa caaatcaaaa gtaaatcata aaggggtttt

184141 cactactaaa tatgaaaaca taacatatga aattgagcag aatggcacac taggaatact

184201 tagggagaaa cctgaaaaca agggaataat tccctactca tcccgagcca ccactatttt

184261 acccaaatca aaaccaggta tcacccaaac aaaacaaaac aaaacaaaaa caaaaaaaat

184321 tagaaaacct acagggaata tagctcatga acacagatta ataaaccttt ggaattccag

184381 tgcatccaac acgacattga aaaggataac acagcacgac caagtggggt ctgtcttagc

184441 attacatcct catgactgtt aacatttaaa aggacgtcca ggaaggttaa cctctcaaat

184501 gctaaaatta aaaaatcata tgattatctc tatagatgct taaaaatacc ttgatgtatt

184561 ccaacatctg tttggataaa aagtcttctt aaattagaag taattgggaa cacggttccc

184621 ctaatccagg gcatctctga aaaatcaagg gttagtaaca tttctggagg cagactacag

184681 tgaggtggac tgtggtaaat caaagatcca tgcacactgc catccataga cgcaaaacta

184741 agagtagcac gaaagtgggg aatagtcatt tcaacagtag tgctggacaa tccgtatcca

184801 tagggaaaaa taatgaatct caatgctgaa ctctctccat aaagcagcag gggccaaacc

184861 tggcctctcc tggttcttct agggcccagg agctaactat gggggtttat ataagtgggt

184921 ggaaaaaata atcttttgtg acatacaaaa ctacgatttc aatgtccatg cacgaagtta

184981 tgggaacatg accccgtcag caagttacac agtgtcgccg gctgacctgg cacagtagtg

185041 gcagagctga gtaggtggga cagaaaacag aacatattca ccacctgcct ctttacacag

185101 tagttttgct gactcagcca cgcacacaat gaaactgagg tgagccaaca ctgacagatc

185161 tattaagctt ctgaaagaaa gcatagcaca ttcccttcat ggccctggag taggcaaaga

185221 ttgcttagag aggacacaat tatcataatc ctacaaacta catttttcga aatcttgcca

185281 ggatggaggc ataggaagaa attcttcact tcctcacaca atttcccaaa taaggaagaa

185341 tccttctttt caaccaattt aaaaacaaaa acagccagat atgccagaca atcaaactga

185401 gtggaaatct gacaaccaag gagttacaga aaaactcttc atccagagtg ataggaaggg

185461 tggagaaggg ctgcccagag gagagcatgc gtggccaggc gatggggtgg gtagtggcac

185521 attcccacac agataagccc ggaagaacaa gtggagagtg agacagacca caaaacccag

185581 ggtttcagca cagaaaacta aagacacaaa acctctggtt gtaaaaatct atgggggctg

185641 cactggtggg agaaactccc agtctcacag gagagtttgt tggagggacc atggggtgct

185701 agaacgtaca caaatctgca cacctgggaa tcagcaccta cctgaaaatg cacagttagc

185761 ttgtgggaaa tgagagagaa gtgacagaaa ggctgtgaga tccaagcaag gggcattgtt

185821 ccctcaggga cccctgcccc acatacagcc tcacaacaca ctgaagatgg ttgtcccacc

185881 ctggtgaata cctaaggttc tgctccttac tacataacag gtgtgccgaa actaatatgg

185941 gccaaatgaa agaacagatc aaaaccccag aaaaaggcta accaacaagg agatagacaa

186001 cctttcagat gcagagctca aaacactggt aatcaggatt ctcacaaaat tgaagagctc

186061 ggatgcaaaa tgggaaaaga aatgaaggct acccaaagtg gaatagagta aaatatacag

186121 ggaaccaaca gtgaagggaa ggaaaccagg actcaaatca atgctttggg acaaaaggaa

186181 gaaataaaca tccgactgga acagaatgaa ggaacaaaaa ttgaaaaaaa tgaggagagc

186241 cttaggcacc tctggcacca ctttaaatgc tcccacatcc caatcatagg ggtgtcagaa

186301 ggagaagagg aagagcaaga aattgaaaac ttatttgaga aaaatcatga agggaaacct

186361 gcaatctagc gaaggaaata gacttccaag atgtccagga agctcagaga ggctcaaaga

186421 aattggaccc aaggagcaca caccaaggca catcataatt aagttaccca agagtaaaga

186481 taagcagaga atattaaaag caagaagagg aaaggaaaga gttgcttaca aaggagttcc

186541 ctttagaata tcagctgatt tatcaaaaga aaccttgcag gcaagaaggg ctggaaagaa

186601 gtagttgaag tcatgcaagg caaagaccta catccaagat gattctactc agcagaacta

186661 tcatttacaa tggaaaggca gagaaagtgc ttcccaaata aggtcaaatt caaggagttc

186721 atcaacacca cacccttatt atatgaaaag ttaaaagtaa gaaatagagg aagatcaaaa

186781 actaggaaca gtaaaatgac aagaaactca caagtatcac caattgaacc taaaaaaaaa

186841 caaaaccaaa gctaagcaaa gaataagacg ggaagagaat cacacaaatg gagatcacct

186901 ggagtgttat cagtggggag gatgagggag gggaatgggg gtaaaggtac agggaatgag

186961 aaacatattt ggtaggcaca aaatagacag ggggcggtaa aaaatcgtat aggaaacaga

187021 gaagccaaag aacttctgtg tacaacccat ggacatgaac taagggtagg gggtatgaag

187081 gagggtgaga gggtgcaggg aagatgaggg gaaagtggag gaaaacaatt gggacaactg

187141 taatagcata attagtaaaa tatattttta aaataaacaa agatctaaac ttaaaaacac

187201 acacattctc ctttatgaaa atttaaaaaa tccttttcat caaaagatat taagaacata

187261 gaagtatata actgaaaaga gcatgtattc aaaatataaa taattcctag aaatcaataa

187321 ttaaagaaga caaatcaatt ttttaaaaaa tatgacaagt tgacgatggt gctctaaaaa

187381 attgagaatg acatgtaagt agaccatttc ttagttccgt cttcacagag ggtttctttc

187441 agcgctgagg agggattttg gctgatggga gcccagcgta gaccctgccc aggaatcgct

187501 gggtgaagac agagctttgc tgagggtcct gccttccctt ccctgggcag ctgcatgcat

187561 ggcgtcctct ttccatgatt attcactgat caggtggatg acttttactc ccatttggaa

187621 aaaggcatca cacctccatc accaccacag gcaaagggtg agcctgagaa aggtaaataa

187681 gcagcaggag ctgtgcagag tccttgagga tccctgataa ctaatttaga cagaacccag

187741 cctggggggt tctatgtgac tgactgaggc agagcccagc cagctccagc agggtcaaca

187801 gcgctgctgc acaagtggcc acctgagggc gctgtggctc cacggagcaa tctgctgagg

187861 gcaccctgtg ggagagggag ggttcatggg aagaatcttg acttggttcc tgcaagaagg

187921 agattaatcg atgcatctga ggttcactga tcttatcttc tcacattaac tgacaaccat

187981 gtgggtaagt tatagcaggg agccatgaag taggccttgc agggagttaa gtttctcctg

188041 gagcctgtgt ttggtggaga gtgtgatggg gcatcaggga ggagcctggg taacctgagc

188101 cagtgtagag caaggagcac ctgctcaagg cagagacaac ctgtgctgat ggccacccgc

188161 cctggaccct ccgggcccaa ctcagcacag ccagctgcac ctgctgtgag gtgatggaca

188221 acctcaggga cacaggacca gctgagcctg tcccggaggt gtgagtgccc acgagattct

188281 acaaagacag tgacatcaga acagtgacgt cacaggcaag ccctccaaag agagcaaagg

188341 gagggaggga caagcctctg ctgtctcacc ccaaagacca gagccctgag cagagacatg

188401 gcccgcctgg tcctgcctga atcctccagg ggcacccggc tcctctgctg catggtcctg

188461 tgtctgctgg gaccaggtga gtctcagctg tggtgggtcg tccctccaac ctagcgtcct

188521 tgactgttgc tggagtgtcc atctgcctcc tgggctggtc tcagctccat tttcttcctc

188581 cacagggtca gtggctgctg gagtcaccca gtccccaaga cacctcatca aacgccatgg

188641 tagggaggct gttctgaaat gccaccccat ctctggacac aagagtgttt actggtaccg

188701 acaggctcgg gggcaggaac ccaggtttct cattgagtat tatgaacagc aggagtcagg

188761 gaaaggaaaa ttccccgacc acttcaaagg aaagcagttc agagactaca gctccgagct

188821 gaccatgcac accttgcagc tggaggactc ggccgtgtac ctctgtgcca gcagcttaga

188881 cacagccctg cagggcccat gcctttctgt acccaaaccc tcctgtccca gctgatgtca

188941 caggtagagg gagggggcct cagcccagtt ccacagatgg ttttcattgt tttctgccac

189001 accccttgcc ctgcggagct cacctaaacc ttcctgaatg ttctcccagc tctgccagca

189061 gtgtgggatt tccagcacat ggcaggaagc cctcttctgt gacagactgg agtccacact

189121 gtgtgttgtg gtgcccggtc ctggtgaaca ccgtgtgtcc actttgcaga gtagggagca

189181 ctcagtgcac catttcatac accgggagct tcagctcctg gaccttccca atgtcccatg

189241 tacgtccatc ctccatctcc acgcctccga agtcaacaag cccctggatt ccatcggcct

189301 tccagagcct tggtccatgg gatagaacag atcgcagtct ctcctgtttc aagagcatct

189361 gagctgggat ggggaggtca catctctgga gtgggggaca cggctgatgg cctcccatct

189421 tcgaggatgt agagcttcaa ggcagagcac atgtgtgtcc tcttatagtt ccagacagag

189481 cgagaccatg atatgctcct aagagaggag ctaaatgacc cggggaattt ccagtccatc

189541 aggggaaaac ggaagccctc tgggagtgtc aaggagcatt ctgggagctc atgcggggaa

189601 tgagtgtttc tgaactaccg gacagggtgg gggtgggggt ttctcagcct gtcctcagct

189661 ccaccacttc tggtccggaa atctcagctt gctccatctg cccaggtgtg gagctgcaga

189721 caacagctga gtgtcacatt gccctggcct ctgatatgtc agtccgtgcc cttgtcactg

189781 ctcggtggtt gttgttgccc atcagcatga cgtcaagata cacaggcaca ggcagggtca

189841 cgtcacactg aggtatgacc tgtgtaagag gtagcagcag tgggaccgct ggctgaatat

189901 ctcacccccg tcactgacaa ggctcctccc tttgtcacag aaagcacagt catggggcca

189961 aaggtgtgta gtcccccggg gcctcagctg gaaccagggg agactcttac ccgtgagcat

190021 gcagagacct gagctcgggt ggcagggatc ccactagtag gtgaatcgtg agacaatgta

190081 aagtagttct ctgattccaa tggccttgac cccaaggcta tgaaaactgg atatgggttt

190141 gttcctttac gggagaggat tgctatgggc tgacattgaa attatccaga gtttatgcaa

190201 agagaaaaac taggagaatg tatctgaagt gaaaattgat catataagtc accaccaaag

190261 catgagcatt gctatgagaa catttcctag tctacagtaa tgggaggaaa aactgtacag

190321 gtaaggaatt gtatcactgt gcatagaagg aatttcctta ccaaaagtca ggggaaagca

190381 tgcgtactgc taagatttct ttaaaaaagt gactttcaca ttttcccatg ctgcactgct

190441 caactatctc tatgtcactg gtttgttcct gtgggggagg gggaggcagg tgagctctgt

190501 agctgaagca tctgtgaagg accgtgatgt caccgaggcc ctgagaggct cacttgtatt

190561 tccccagaca gaactggaag acgtgacatt gtgcgctgga cctgaaatgg tcagcagggt

190621 ctgcttccgt gtggctcttt gtctcctgtg ggcaggtggg tgcaggtggg cttcctgccc

190681 tggaattctc agccttcagt cccaggcttg gtggtaggat gacaacataa tggtgtgtca

190741 ctttctccac aggacacttg gaagcaggaa tcacccagag tccaaggtac aaggtcacag

190801 ggacaggaaa aatggtgaca ctgaggtgtc accagactga taaccataac gctatgtcct

190861 ggcatcgaaa agtcctgggc catgggctca ggcagatata tcactcattg ggtgttgggg

190921 tcgccagcaa aggagaggat gccgacgggc acagtgcgtc tagagtgaac atggaggact

190981 tcctgctcac gctggagtcg gccaccccct cccagacatc tgtgtacttc tgcaccagca

191041 gagacgtgct gatccacagc gctgcacggc caccttctct ctgcacagaa aggaaggttg

191101 aggccctgcg ctccagattc atgtgacccc tgagcaaact cttacacttt agagccctga

191161 ggcaccctgc ctccatgggg acccttggtg tgtgatgagg tccccctcta gattagtgtc

191221 taccaggcct ggtgtaggtc cagggcctca gacacacagt ctgcactgtt gttgtctgtc

191281 agtctgtcct gccaggtgct ctgttagctg ggaaggtgtt tgctctttcc agcttttctt

191341 gtcatcagcc tgagtgtgat gcctccaggg tggggagtca tgagcagcat atcctggtag

191401 atccccgtga gtgtcacata caggtgtgtt gaaggtgcag gacatcgagt cttcctccag

191461 acagaagcta caagacacgt gtttcagtgc tggtgactaa ggaggacacg aggctttgct

191521 gtgggcgggg gagacaccta atactacgtg gaggttaatg agcagagaga gggcactagg

191581 tcgagtcctg cagaaggagt tggagcttcc aacacagata tttccatgca catcactcca

191641 cgtgtcatca agtgggccca ggaagtggcc ctccagtgtg atcccatttc tgtacccttg

191701 tcgtttacat gtgctgacag gtcctgggac agggcccaga gtttctgata tactttcacc

191761 tcctggatcc ccagactcct gtgggccacc taggaccggt tctctgctca gaggccagct

191821 ggctccactg cactctgagg atccagcctg cagagctggg ggactgggcc gtgtccctct

191881 gtgccagcag ctccagcaca gggtgacccc gtcacccctt catgtgaaca tgccctcatc

191941 ctcctccctc catgcagctc ccgcactcta agcagctctt ctttgctcat tgttccccag

192001 tgacgatgca gttctgggca tcagctgtgc tttgggcaga cagaacacag ccctgcctgg

192061 tgagcctcag ttggctgggc atggtactgc caaccaaagg cttgctggtt ggattcccgg

192121 tcagggcacg tgcctgggtt aaagatttgg acgcttgttt gggcgcatgc aagatgcaac

192181 cgatggatgt ttatctctca catcaatgtt tttctctttc tctctcattt ttttctcatt

192241 cctaaaagta aataaataaa tctttaaaaa actaggattc ctagagccaa ctcagtgctc

192301 caaggcatgg acccaatgtc atggacagga acagtctttg gtaaaactgt gggtctgggc

192361 ccccaacaca gctgcgctgt gggtctgggc gtgtgcccac tccccagagc ttgtccacat

192421 ctggaattcg ggtgacaccc tcttcctgtc catccttctc tgagccaccc tgtcaccttg

192481 ggggctttct gtagcaactt gtcaaaggtt ccttcctgct ggaagtgcag gtcttcaccg

192541 cttttgtgcc cgttgactga gaatcaccat agtgatacca gaatggcgtc atgtcctctg

192601 cgaggctgca ggagcacaca ctctattgtg ttttcatttt cccgccactc catcctgagt

192661 gtcaggctgc agcgtacctc cacgacctct ccacttcatc ttgaccccgt gggtaaatcc

192721 ttctagacag gcggggtgct gttggcaccc tcccattttg acttcaagta atttccctgg

192781 attctgtaac tcaatgtgat acggcatttt ctcgacaaca tgctttattt ctttttattt

192841 atatgctgtg ttatgttggg acatatcatt tgctacaaat ataaataatc gagcccctgt

192901 gggggggggc agtggcagac atctggcaaa tccgcggcca tgggcagagg ggacaggcct

192961 tcggatcatg ggtttccggg aacctgagag gtaatgctac agaacacaac taggggggcc

193021 tgggacaatg tactattatt gaaagaaggc cctgggtccg ttatgtccgc cgccctagga

193081 aagacgtctc tcaatgccag agcttcgtga aatggaaagg aaatgtttat ttaatgctat

193141 actaacttaa agtagtgacc taatgtcttt atcaaaaaaa tcctaaagtc cctaaaaaca

193201 cccacaaaca cacagttctt ccttccttcc ccctttgccc agtccagcgt accgtatctc

193261 aggaaaggaa atagaagtcc atggctcagg cagtcctctg gttgttccca gttagtcctc

193321 catctcaact gggagacctc cctggattcc cggcaccctt ggctgagttg ccgggatctc

193381 tgctaaaacc aggtggtggt tcccccttct aaagctgtgg gggtccccac tctgccaggc

193441 cgcgtggttc tctcctcagg gctgcgcatg gttccctctc tctgggatgc gggagtctcc

193501 actctgctaa agacgcgtgg ttctctctcc aattgcggtg atcttctctg cacctccaca

193561 gccacgtggt tctctcctct cagggctgtg cacagctctc cttcctaaag cagcatggtt

193621 ctccctctca atggccgcca agtctgggtt ttaaatcccc gcggccaatc ttcctctgca

193681 gccccatttc caactcctcc cacactcggc ttcacatgcc agaactcgta tccttccagc

193741 tttactgggc tgccatcctg agtctgggca ggcgtggccc catgccctgg agccaatcct

193801 ctctgagctc ccacgcaggc gctgtaactc aggggacccg ccccccccag ttacatctgg

193861 gtggggaagt tacttccatt cccccggctt agagctgatc acagctactt aacatatcta

193921 tgcaaccagt caaaggttat agatatgcca aacgaccaca ccagagctta gctgcaaggc

193981 tgttgctatg ctaaacagct ctcaatggcc ctgctccatt tgtctcttcc cctaacccac

194041 accctgggcg ggggggagac atcccaaaat ctcctggaca ccttaacttc tggaccccat

194101 ttcaaatgcc tatttggggc cccccctctt ggctgcaccc tgtaacagta acccttggct

194161 ctgagtctgg gactgcatgt cacagacgca gtcccaatca aggtgcctga ggcttgggta

194221 gcaggtaatg acagtggctg ttcctcaaca cccacctaaa cactgtggta gccatgacac

194281 cggatctgct cacccaggaa cgagaggcct tggggggaca tttcctggtc gtatggggac

194341 tgtgctcggg tccctgggcc tcctgggagg gagggaggcc tgggctccag ggcagctccc

194401 tgtccttggg aagccatcag ggaccccttc ccggaccctg tccccatttg tgccactgcc

194461 agcacctgac cctggccact ctgcttcctt aggacactgt tttgtacccg tgtttgcttg

194521 tctcccacaa cgccgccgct tcacccagaa ccagctccgt gctcacagag caggtgccca

194581 ctgtgcgggc tgctgctgcc gtgtgggctc tgcgtggggg gtcagcccca gtgtcctcag

194641 ctccccattg ggtgtctttt ctccagggct gctcaccaac cctatgggtt acatttcact

194701 tgtgtttcaa ttttactttc gtgcacacac atttgcctcc atcttgaagg taatatcatt

194761 atcttcgcag tctttgctta tttatgtttt ggagataaca tgaaacacct gttttctttt

194821 tgcattatgt ttgttttagc aacagagata aaaatgcctc acacttccac ttgtgtgttt

194881 tccttcctac cttggactag atattatttc ctcccattcc ccttagggtt caggatgttg

194941 agatagctta taaaggatag tgcttctgga atcctatgtc tatagaccag gcagacaagc

195001 ccaccatccc ttggactgtg gcagaagata cagcaatgag atgtcagtta ctggccacca

195061 gatggcactg tgatgccact gacctggaag tgggtctggg gagagtctgg gagagcagga

195121 aagggttaag aagacttggg cagggaccaa cactgggcag atgctgaggc aagttttagt

195181 tgtgggcagc ctccctacag ctgtggagtg ggtgggaggt cctgctcaac cttaaaagac

195241 ccactgtgga tggatttggt taaggcagcc ctgctggcag gggcaggtgc agagagagaa

195301 ttaactgtcc cctagcactg ggggagacca ggagtggccc aggctaggac agtgacggga

195361 cccctgccca catctctgca gggggaagca gccagagtca ctgaggggtc ctaggacgcc

195421 tggagctggc acaggctcct cctgcccagg gagaggggac ctgtctgcac ctcaggaaac

195481 accaaggagg ggtctccttg agtagaaagg tcaaaaaggg atgtagggcc agcagcaagg

195541 gctggtactt agttcaggca gggtcaggca gggacaatgt agtaggcact gtggacggtg

195601 gggaagctgt tgggaaccgc cctgactggt atcagaagct gtaaccccca cctaggctca

195661 ggccagggga acgcccttgg aaccgtaagc cagcaaggag acaaaagctt atctccctgg

195721 caggaatgct gcttctgctg ctccattcat aactgaaccc caaagcttgg tcggttagcc

195781 aaagatgggt aagattccct aagaggggaa tgacctgaga caggcatgat cacgtgggtg

195841 gctccccaag aggactttgg gggctgcagc aaaaggggat gatggaccct cgttccttgg

195901 ctttgacata gactgagttc tcatcctctg ggagaaaatc tccttatctc ttggttgcct

195961 tagttcccgt gctccaccta agcctgaaac aatgacaggg tggtccagct ctgtgctgaa

196021 aagggcggat tccccaggtg accaggccta agaaagaaca cgtaaattac tgtgaaacct

196081 gctttgttta gaatgctctc agttgaatga gaggggtcca aagaggaagt tagtttgttc

196141 ctcaaagttt tacaggtctt tgatcctgac tcaaacccac agagttcctt gttttctttg

196201 gttccttact tcctcgtaat gagtactgta cttgaattat tatggaaaac gagcccaatg

196261 aaagcaggtt tggacgataa atcggagcgc tccccactag agggggtggc cattccctcc

196321 ctacttctcc atagaaactg gtatgtctct gtgcatgttt tctctcgcat atctacgagc

196381 catcggcagc gttccatgat cactgctggc tggtgaccca cgcatcagaa agctacagcc

196441 aaccttagcc atggggttgt gcaagacatg gaatgatgca tagaagttag aataggggtc

196501 ccatcaaggg cacagagcac caggggacag cctccttgga gggttcatca gagcaactac

196561 aacatcagac caatgatgtc atagcaaatg ctcctatcag cacacggagc acagaactgt

196621 agaccgctca ccccagggga ccccgccctg agctgggaga ggtcccgggc agctgccatg

196681 ggccccaggc tcctctgctg tgtggccctt tgtctcctgg gagcaggtga gccccggaca

196741 ctgtgtgtaa gtctggacac gcagtcacat tcctgacaca caagcctggg ggtggtggct

196801 gcctcgggag gctccctgtg ctctgccccc agctcctctg tgtctcctcc caacaggccc

196861 agtagactct ggagtcacgc agaccccaaa acacgtggtc aaggcaagaa aacagcaagt

196921 gacactgaag tgttcctata tctctggaca ccaatctatg ttttggtaca aacaggttca

196981 gggtcagggc cctgatttac tggttgagta ctacattggg ctagctcgag gaaaagggaa

197041 cctgcctgat cggttctcag tgcagcagtt tgatggctac cgctccgagc tgaccgcgag

197101 ctcgctggag ctgagcgact cggccctgta tctctgcgcc agcagccctg acacagccct

197161 gcaggcgcgg cagcctcctg cacagaaaca ctccgccccg ctcaggaagc ggcagctgcc

197221 agccacacag gctcaggcct gagggagctg ctcagcttcc ccaggcccgg ttctgctgct

197281 cccagatggg gtgggatgtg atgcccacag acatcatgca gctcaagtat cattgtcctg

197341 cacatacacc tggtgggaat cctgttcctg ggtctcacat tccgtaaccg gaagaccaaa

197401 acatcactca agtctgctcc tgaacgtctg tgtatctgtc ccatccacct ctcccagaag

197461 cgtgtccaca tctggctccc cttttctggg tttagattca ccgccctggg gagatctgac

197521 accattgggt tgtcattttc ttacccggca gcttcaataa catctcactt catggcaaca

197581 catattcatc aatccttcct aatgacaaca aagaaaggca caactgcatg cactgtggcc

197641 atcacctaga cctacacaaa gccctgaagc aagttgtggg tcatgtcaag tgtgggactg

197701 tgagtagatg cgctacaggt gtttaaaggc tcattatttt attatcagtg tcagcaattc

197761 ttcacggggg atagtccctg tggaacaagg aagaaattta aaggcagcag tttcactgtc

197821 tcacctggga ggtggctcct gtccccatgt ccctcacaca gctgtaactg ccggggccat

197881 cggggcacct gcagagccga acaccatgca gaaacccagg tcccagtcct gggctggatg

197941 tgctggtgct gcctcaccct gaaacggaaa tcaggttctc aggtgactct gaaggtcctc

198001 aggagcacat ggtggggccc catcggtcag gggatggaga gaaatcagag ccagagggtc

198061 ttctagggtt tatgaggctg cccctgaggt ttggccacca gacaccagga gggcccctgt

198121 gggaggagcg acagtgagcg gatgctggac gaccagcaaa gtaaacagga ggctgaggga

198181 cgactgggcc ccagagccct aaaggcatca gaatgccccc aggcgttgcc ctcaggtaca

198241 agaacacagg gccggggaca cacagcagac cagggcggca tgaggatctg cgggcaggac

198301 gaagcacacc tgagccacac actgtgaaca cagagggtga ggacgcctct gggactcccg

198361 tcagttcagc cacacgcgtt cttctccctc tgagctgtcc cctgcccgtc tgcttctgat

198421 ttgatgctct ggggaagggg gcgtgtccct tgtgacaagc tggctctggg gcccaggaag

198481 tgggagtgac acctcagaga gactccctgg aatcgccctg ctcctgtgca gacacccaca

198541 gacccagagg gaccctgagt cagcctgcct gctgccatga acggcagcct cttgtgctgc

198601 gtgaccttgt gtctcctccg agcaggtggg tcctgggctg ggcccctctg tggggacagt

198661 gcccaggccc agcaccgagc cgctccctgg aggcttcagc atctgttccc ctgtcctccc

198721 cgcaggtctg gggatggccg gcgtcactca gtccccaacc tcccaggttg tgacaacagg

198781 acagacggtg accttgcagt gttcccagga cttgaaccat aactacatgt actggtaccg

198841 acaggacctg ggtcacgggc tgaggctgat ccattactca gtgggtgttg gggtcaagga

198901 caaaggagag gtcccggatg ggtacagcgt ctctagatca aaacaagaag acttccccct

198961 cacgctggag tcggccaccc cctcccagac atctgtgtac ttctgcgcca gcagtgaacc

199021 cacagtgctg cacggccgcc tcctctctgc acagaaagtc agggggaggc ccagcgccca

199081 gactcagggg aaccctgtgc agactcctac accttggagc ccactgtggc ccctgcctgc

199141 agggtgaccc tctgtatatg tagggtgacc ttctgtactt gtggggtcat cctctaggcc

199201 agtgtctgcc aggctgcaac caaacccagg gccttgaaca caaatgtgtc cacttggctt

199261 gctgtctgtc tgtcctctgc atctgctcca ttggctggga tgatgcttcc ccagctcttg

199321 cttttcttgt cagcaacctc agtcataggc catcagggtg ggctgtcatc agtaaccaga

199381 cacatctgtg cccactggtg tatcacacgc aggcgtgtca caggtcagtc acggtgagtc

199441 tccttctgga aagcggccat gagaaacccg tgtttacatg cagggcgact tagaagcaca

199501 agaggcttct ctgtggcctg tgcggacatg gtattacatg gtagtgtgag cacgcacaga

199561 cagggacctg ggttgagacc tgtagaagga agaggggctt cccactccaa tatatcccag

199621 ggcgtcactc catctgtgcg tctctgagtg tgtcagtgca tccagggcag agttgggtca

199681 acccctatgc ccaccctctc cttagcaagg gaccaggttt agcatgtgcg actaggagag

199741 tggaggggat ggtgacagaa cagggtgctc ccaggaggtc ccaaatgaca aagagtcaat

199801 gaattcaccc ttcggtgtgt tgcactgatg acactgttga catgtcagag tgaaaatgcc

199861 agctgacccg gaagacaggg gaatgaggtg tcacgtggga accctgtgcc tagacagcag

199921 gaacccgaaa gggacagccg tgcacagttt tgtttaagaa ggagtgtaga actatttaca

199981 ccagtcaccc aagtgcgctc tcacatacat cacgcatccc tgagttcaag gggcctgaga

200041 gaggcagtga tgtcactatg ggaacggccc ccaaggtgac aagacatgtc cctcctcctc

200101 tgctcctgct cacaaggacc tgacccaggg aacctcccac tcctgacctg accctgccat

200161 gggcaccagg ctcctctgct gggcgaccct ctgtctcctg ggggccggtg agtcctcaga

200221 aaaccaggca gcttcagtgt gtgtgtgtgt gtgtgtgtgt gtgtgtgtga tgattccatt

200281 tgttttcctc cttctgttgt caacttctgt gtcccaggtc acacagaggc tggagtctcc

200341 cagtccccta ggcgaaaggt caccaagagg ggacagaacg tgacatttca atgtgatcca

200401 atttctggac acactgttct tctttactgg tactgacaga ctccgggcca gggcccggag

200461 ttcttggtgt actttcagta caagggagct ctagacacct ctgggatgcc taaggatcgg

200521 ttgcccgttg tgagacctgc tggctcctcc tccactctga gcatctagcc tgcagagcct

200581 ggggacttgg ccgtgtacct ctgtgccagc agtgtaacca cagtgtggca ctgtccccaa

200641 ctccctgctc acacaccctc ctgtgccccc ctctgctcat ggttcttaga ctgcccagca

200701 aaggagttgc cctgttcttc ctttctcaag gagcataagt gcatttggat tttcaatgct

200761 ttttctggca ggaatgacta gaacacagca tgctggattt ctggggttcc ttcatgtacc

200821 taaagattct gtaattgagg ataagccctg gagctgtgcc ttgggtgtgg tgaaatcttc

200881 tcttgtgaaa tagaaagttt cagcagacgt gcgacaacct ccgagtcctg agtgtcactg

200941 atgatgacac gtgatgccca catgtgagca gccatgcagc attctcttag gctacatgca

201001 gccaccagcc ttaaaaatca atagttacac tccttagtgc aaactgccat tcaaatcatt

201061 cacagacacg agtcatgtac aataatggac attgatcaat atccatacgt ggttttacta

201121 tttttcttga caatgctgaa gtcattccat atttaatttc cttcttctct agcctatttt

201181 tagtacaatt taaatttatc ttggattaat aaaatcccta cttgagaagg ttttaaaggg

201241 aagtcactca aataagatcc aaataatagt tgtactgatc tccccttatt ttgtggagat

201301 cctttccaag acccagtgga tgcctgaaac ccctgatggt ttggtggaat ccaacgttta

201361 ctgttccccc ccacacacac acctttcatt ttgaaggaag cactttctgg catctcttcg

201421 gcagatctgg ttgctagcat catggccctg catgtcacgg ccgtctctaa gtacaataat

201481 ggtgactgga cctcaggcac tgcccgccca cagcagtcag cctgatgaca gagctggccc

201541 agggagtgat gggcagggac agagcacagt gtggagacgc tgggcaaggg gctgattcac

201601 atcccgggtg ggacagaagg gacagtgtga gatgtcacca cactactcag aatgacacat

201661 gtttaaaact gttaaattct ttttttcttg aatttatcat ttaatatttt gggactacag

201721 ttgaccttag gaaactgaat ctgtggaaag cgaaagtgca gctaaggggg gacttctctg

201781 ggtaaaagta aaatgtgcga caacaatagc aagacaggga gcaggtaatg cataggtgtc

201841 ttaccattgt gagagcctcc tgatctacgt ggaggggtat aggctgtgat gaagctgaga

201901 ggcccttgca aactctagag ccaccacgga aatagcaaac agagttgtat ctcataggcc

201961 aacaaacagg gaaatggaac tattataaaa atgcctaatt aatacacaag aagggatgaa

202021 acaaatgaca agggaaacaa agaacagatt tgctaataga aaacacacag caaaatgaca

202081 gacatgaagg taaccagaac aatatgtaca tcaactccaa atagtctaaa tgcccaaatt

202141 caaaaccatt gattgtgatc atggataaaa cacaaaaaag cagatgtgtg ccacctgcca

202201 cagcggcctg caccagcagc agaagggctc acaggtgcac tggagcagac ccaccgcgct

202261 gacactgatc acacgaaagc tggcgcagca acgtgagtat cacacaaagc aaatctcaaa

202321 gcaaagacta tcatctgaga caagaaaggt catattctaa tgataaaaag gtcaactgat

202381 caagaagaca aggcaatcag aaacacttat gcatctaaaa acagagtttt gaaatgctga

202441 agacaaaaag gcaaggagag agagacaaat tcacaattat agtcagacat atcatcacct

202501 gtcagtaatt gattgaacaa cttgaccaaa aaggaaaaat agtgagggaa ctgaagccat

202561 agagaacatt atcgatcacc ttgacctcat gcacattttc gggatagttc acccaacagc

202621 agcagaaggc accttcttac caaaggcaca cagactatgc aacaggataa tctgtggact

202681 tttactataa aataagtaac aatacattga aaagaattca agtcatacaa accgtgttct

202741 ctgaacacag aggagctgaa ttagaaatca ataaccgaaa gaggtctaca aaaatcccaa

202801 atattagaaa ctaaataaca atattctaaa taacccatag gacaaagaac aaatcgaaag

202861 taaatcataa agggattttc actactaaaa atgaaaatgt aacatatgaa aatgagtgaa

202921 tgccacacta ggaatactta gggagaaacc tgaaaacaag ggaataattc cctactcatc

202981 ccgagccagc actattttac ccaaatcaaa accaggtatc acccaaacaa aacaaaacaa

203041 aacaaaacaa aacaaaaaac caaaaatatt aaaaacccta cagggaatac ccctcatgag

203101 cacagattaa taaagctttg gaattccagt gcatccaacc cgacactgaa aaggataaca

203161 cagcacgacc aagtggggtc tgtctcagca ttacgtcctc atgactgtta acatttaaat

203221 ggacgtccag gaagggtaac ctctcaaatg ctaaagtgta aaaaatcata tgattatctc

203281 tatagatgct taaaaatacc ctgatgtatt ccaacatctg tttggataaa aactcttcct

203341 aaattagaag taattgggaa cactcttccc ctaatccagg gcatctgtga aaaatctggg

203401 gttagtaaca tttctggagg tagaccgcgg tgaggtagac tgtggtaaag caaagatgca

203461 tgcacactgc catccacagg cccaacacta agagtagcac gaaaggggag aatagtcatt

203521 tcaacagtag tgctggacaa cccggaccca tagagaaaaa taatgaatct caatgcttac

203581 ctcactccat aaagcagaag gggccaaacc tggcctctcc tggttctcct agggcccagg

203641 agctaactat tggggtttat ataagtgggt ggaaaaaatt atcttttgtg acatacaaaa

203701 ctcagatttc aatgtccaca cacgaagtta tgcgaacatg ccccactcag taaggtacac

203761 agtgtctctg gctgacctgg cacagcagtg gcagagctga gtaggtggga ctgaaaacag

203821 aacatattca ccacctgcct ctttacacag tgattttgct gactcggcca tgcacacaat

203881 gaaattaggt gagccaacat gacagatcta ttaagcttct gaaagaaagc atagcacatt

203941 cccttcatga ccctggagta ggcaaagatt gcttagagag gacaaaaact attgtaatcc

204001 ttacaatcac attttttgaa ttttggccaa gatggaaatg taggaagaaa aacttcactt

204061 cctcccataa tctgacaacc aaggagttaa agaagaactc ttcatcagga gtggtaggaa

204121 gggtggagac gggctgccca gaggagaggc tgtgtggtca ggcgatgggg tgggcagtcc

204181 cacattccca cacagatgag ctgggaagaa catatgggga gcgagacaga cagcaaaccc

204241 aagggtttct tttatgatga tgatgatgat gatgatgatg atgatgatct tattatattt

204301 cttttttatt ttttaatttt aatcattgtt taagtacagt tttctccctt ttactcccaa

204361 tccagcccac ccacccaacc ctccccactt ctctcccatt accaccctcc ccctagtttt

204421 tgtccatgtg tcctttacat ttgtgcctgt aaacccttcc cattctcccc tgaaattccc

204481 tcctctctcc cctctggtca ttgtcagcct gtcctctatt tcagtgtttt tggttatatt

204541 ttgcttgttt ctttgttttg ttgtttatgt tcctgttaaa ggtgagatca tatggtattt

204601 gtctttcact gcctggctta tttcgcttag cataatgctc tccagctcca tccatgctgt

204661 tgcaaagggt aggagctcct tctttctttc tgctgcatag aattccattg tgtaaattac

204721 catagttttt tgatccattc atttactgat gggcacctag gttgcttcca ccacctagct

204781 cttgtaaatt gtgctgctat gaacatcggg atacataggt tcttttggct tggtgtttta

204841 gtattcttag gatagagtcc cagcagtgga attgctgggt tgaaaggcag atccattttt

204901 agttttctga gaaagttcca tactgttttc cacagtggtt gtacccgtct gcagtcccac

204961 caacagtgca cgagggtccc cttttctcca caacctctcc aacacttgtt gtttgttgct

205021 ttgtttatga tggccattct gactggtgtg aagtggtatc tcattgtggt tttaatttgc

205081 atctctctga tagctagcaa tatggaacat tgtttcatgt gtctatggat tttctgtatg

205141 tcctccttgg agaagtttct gttcaagtcc tttgcccatt ttttaattgg gttccttgtc

205201 ttcttagagt ggagtcgtgt aaattcttta tatattttgg agattaaacc cttgtctgag

205261 gtatcattgg caaatatgtt tccccataca tttgtttctc tttttatttt gatgaggtcc

205321 catgtgttta ttctctcctt tatgtccctt gctctagggg acatgtcagt aaaaaagttt

205381 ctgtgtcaaa tgtctgagat tttcctacct acgttctcct ctaggacttt aatggtgtca

205441 cgttttatat ttaagtcttt tatccacctt gaatttattt ttgtgtaagg tgtaagttgg

205501 tgttcaagtt tcattttttt gcacgtagct gtctagttct cccaacacca tttgttgaag

205561 aggctatttt taccccattt tatgttgctg cctcctttgt caaatattaa ttgactgtac

205621 agacttgggt ttatttctgg gctctctgtt ctgttccatt ggtccatgtg cctgttttta

205681 tgccagtacc aggccatttt gattacagtg gccttgtagt atagtttagt gtcaagtatt

205741 gtgacccctc ctactttatt ctcctccctc cttctcaaaa ttgcagcagc aattcggggt

205801 catttacgat tccatataaa ttttggaagt gtttgttcta tgtctgtgaa atatgccatt

205861 ggtactttag taggtattgc attgaatgtg taaattgctt tgggtagtat ggacatttgg

205921 atgatattaa ttcttcccat ccatgaacac ggtatatgtt tcaatttgtt tgtgtcttcc

205981 ttgatttctt tcctcagtgt tatgtaaaaa ccaagggttt cagcacagaa aactaaagac

206041 tcaaaacctc ttgctgtaaa aatctgtggg ggctgcagtg gtgggagaaa ctcccagtct

206101 cacaggagag tttgctggag ggaccatggg gtcatagaat gtatacaaac ccacacacct

206161 gagaaccatc atctgaaaag gcacaattag cttgtgggaa gggagagaga agtgacagaa

206221 aggctgtgag aaccgagcaa ctggcattgt tccctcaggg acccctgccc cacataaagc

206281 ttcacaacac actgaagatg gtgcctcacc ctggtgaata cctaatgttc tgctccttac

206341 tacataacag gtgtgcagaa actgagaaat atggcccaaa tgaaagaaca gatcaaaact

206401 ccagaaaaaa gactaaacaa caaggagata gacaaccttt cagatgcaga gtggaaaaca

206461 ccggtaatca ggatgctcat agaattgaag agctcggttg caaaatgggg gaagaaatga

206521 aggctaccca aagtgaaata tagtaaaata cataggaacc aacagtgaag ggaaggaaac

206581 caggactcaa atcaatgctt tggaacaaaa ggaagaaata aacatccgac tggaacagaa

206641 tgaagaaaca aaaattcaaa aaaaaatgag gagaagctta ggaacctctg gcaccacttt

206701 aaatgttgca acatcccaat cataggggtg ccagaaggag aagaggaaga gcaagaaatt

206761 gaaaacttat ttgaaaaaaa tcatgaaggg aaacccccaa tctggtgaag gaaatagact

206821 tccaagatgt ccaggaagct gacacatctc aaaaaaattg gacccgagga gtacacatca

206881 aggcacataa taaataagtt acccaagagt aaagacaagc agagaatgtt aaaagatgca

206941 agaggaaatg aaaggaaaga gttgcctaca aaggagttcc cattagacta tcagctgatt

207001 tatcataaga aaccttgcag gcaagaagag ctggaaagaa gtatttgaag tcatgcaagg

207061 caaagaccta catccaagat gactctaccc agcaaagcta tcatttacaa tggaaaggca

207121 gagaaagtgc ttcccagata aggtcaagtt acaggagttc atcaacacca aacccttact

207181 atatgaaaag ttaaaagtaa gaaatagagg aaggtcaaaa actaagaaca gtaaaatgac

207241 aagaaactca caactatcga caactgaacc taaaaaaaca aacccagaaa ctaaggaaac

207301 agtaagacag gaacaggatc acagaaatgg agatcacctg gagtgttatc agtggggacg

207361 atgagggaga agaatggggt aaaggtacag ggaatgagaa gcataattgg taggcacaaa

207421 gtagacaggg ggaggttaag aatagtatag gaaacagaga agccaaagaa cttctatgtg

207481 caacctgtgg acatgaagta aggggtaggc ggaatgatgg agggtgagag gctccaggtt

207541 acatgacgag aaagtggagg aaacaatcag gacaactgta atggcataat taacaaaata

207601 tatcttaaaa taaataaaga tatctaaact taaaaacaca cacattatcc tttatgaaaa

207661 ttttaaaaat cttgctcatc aaaagatatc aagaacatat aagtatatat ctgaaaagag

207721 catgtattca aaatataaat aattcctaga aaccaataat taaggaagac aaatcaattt

207781 tttttaaaaa tatgacaagt tgaagatggt gctctaaaaa acaaaagtga tatataagta

207841 gaccatttct tagttccttc ttcacagagg gtttcttcta gagctgagga gacgttttgg

207901 ctgatgggag cccagcgaag accctctcct ggaatcgctg ggtgaagaca gagctttgct

207961 gaaggtcctg ccttcccttc cctgggcagc tgcatgcatg gcgtcctctt tccatgatca

208021 ctcactgatc aggttgataa cttttattcc catttggaaa aggcatcaca cctccctcac

208081 caccacaggc aaagggtgag cctgagaaag gtaaataggc agcaggagct gtgcagagtc

208141 cttgaggatc cctgataact aatgtagaca gaacccagac tgggggtttc tttgtgactg

208201 acttaggcag aacccagcca gctccagctg ggtcaaaagc gctgctgcac aagtggccac

208261 ttgagggcgc tgtggctcca cggagtaaga tgctgatggc acgctgtggg agagagaggg

208321 gtcatgggaa gaatcttggc ttggtttctg caagaagagg attatctgat gaatctgagg

208381 ttcaccaatc ttatcttctc acattaactg acaaccaggt gggtatgtct tagcaaggag

208441 ccatgaagta ggccttgcag ggagttaagt ttctcctgga gcctagtttg gtggagagtg

208501 tgatggggct tcagggagga gcctgtgtaa cctgagccag ggtagagcaa ggagcacctg

208561 ctcaaggcag agagaacctg tgctggtggc cacctggcct gtaccctcgg ggcccaactc

208621 agcacagcca gctgcacctg ctcataggtg atggaccaac tcagggaaca cagggccagc

208681 tgagcctgtc ccggaggtgt gagtgcccac gagattctac aaagacagtg acatcagaac

208741 agtgacgtca caggcaagcc ctccaaagag aacaaaggga gggagggaca agcctctgct

208801 gtttcacccc aaaggccaga gccctgagca gagacatggc ccgtctggtc ctgcctgaat

208861 cctccagggg cacccggctc ctctgctgca tggtcctgtg tctgctggga ccaggtgagt

208921 ctcagctctg gtgcgacgtc cctccaaccc agcgtccttg accgtttctg gagtgtccat

208981 ctgcctcctg ggatggtctc agctctgtct ccttcctcca cagggtcagt ggctgctgga

209041 gtcacccagt ccccaagaca cctcatcaaa ggccttggtg gggaggctgt tctgaaatgc

209101 caccccatct caggacacaa ccgtgtgtac tggtaccaac aggctcgggg gcaggaaccc

209161 agctttctca ttcggtatta tgaacagcag gagtacggga aagaaaattt ccccgaccac

209221 ttcaaaggaa agcagttcag agactacagc tccgagctga ccatgcacac cttgcagctg

209281 ggggactcgg ccgtgtacct ctgtgccagc agcgtagaca cagccctgca gggcccatgc

209341 ctttctgtac ccaaaccctc ctgtcccagc tgatgtcaca ggcagaggga gggggcctca

209401 gcccagttcc acagatggtt ttcattgttt tctgccccac cccttgccct gctgagctca

209461 gctagagcct tcctgaatgt tctcccagct ctgccaccag tgtgcaattc ccagcacatg

209521 gcaggaagcc cttctctgtg acagagtgga gtccacactg tgtgttgtgg tgcccggtcc

209581 tggtggacac tttgtgttca cattacagag tagggagcac tcagtgcacc atgtcatcca

209641 cagggagctg cagctgctgg accttccaca cgtcccgtgc acgtccatcg tccatctcca

209701 tgcctctgaa gtcaacaagc ccctggattc catcatcgta ccaggtcctt ggtccatagg

209761 gtagaacaga gcacagtctc tcctgtttca agagcatctg agctgggctg gggaggtcac

209821 ctctaggagt gggggacacg gctgattttc tcccaccttc gaggatgtag agcttcaaga

209881 cagagcacat gtatctgttc tgtcatagtt ccagacagag caagaccatg atatgcacct

209941 atgagtgcag ctaagtcacc tggggaattt ccagcccctc aggagatcct aggagggaag

210001 cagaagccct ctgggagtgt caaggagcac tctgggagct catgcgggta tgcgagtttc

210061 tgaactactg gatagggcgg gggatggggt ttctccgcct gtcctcagct ccaccacttc

210121 tggtcaggaa atctgaattt gctccatctg cccaggtgcg gagctgcaga caacagctga

210181 gtgtcacatc gccctggcct ctgatatttg agtccgtgtc ctttctcact gctgggtggt

210241 tgttgttgcc catcaaggtg acatcaggat acacagtcac agacagggtc acgtcatact

210301 gaggtacgac ctgtggaaga ggtcgcagca ctgggagcgc tggctgaata tcccatccac

210361 gtcactgacg aggctcccct ctttgtcaca gaaagcacag tcatggggcc acaggtgtct

210421 agtccctctg gggcctcagc tggaagcagg ggagactctt acccgtgagc ctgcacagac

210481 ctgagctcgg gtggctgggc tcccactagt gggtgaatcg tggtaaaata taacagaatt

210541 ctcagactcc aatggccttg accccaaggc tatgaaaact ggatatgggt ttgttccttt

210601 aagggagagg attgctatgg gctgacattg aaattatcta gagtttatgc aaagagaaaa

210661 gctaggagaa tgtatttgaa gagaaatttg atcagagaag tcaccaccag agcatgagca

210721 ttgctatggg aacatttcct agtctacagt aataggagga aaacctgtac agggaaggaa

210781 ttgtatcatt gtgcatagaa gaaggaattt ccttaccaga ctcaggggaa agcatacata

210841 cttctaagat atctttaaaa aagtgacttt cacatttttc ccatgctgca ctgctcaact

210901 gtctctatgt cactggtttg ttcctgtggg ggagggggag gcaggtgagc tctgtagctg

210961 aagcatctgt gacggaccat gatgtcaccg aggccctaag aggctcactt gtgtttcccc

211021 agacagaact ggaagacgtg acattgtacg ctggttctga aatggtcagc agggtctgct

211081 tccgtgtggc tctttgtctc ctgtgggcag gtgggtgcgg gtgggcttcc tgccctggaa

211141 ttcccagcct tcagtcccag gcttggtggt aggatgacaa cataaaggtc tgtcactttc

211201 tccacaggac acttggatgc aggaatcacc cagagtccaa agtacaaggt cacagggaca

211261 ggacaaaagg tgacactgag ctgtcaccag actgataacc acaacgttat gtcctggcat

211321 cgacaaaacc taggccatgg gctgaggcag atgtattact cctggggtgt tgggttttcc

211381 agcaaaggag aggatgccga tgggtacagt gcgtctaggg agaacatgga ggacttcctg

211441 ctcacactgg agtcggccac cccctcccag acatctgtgt acttctgtgc cagcagaaaa

211501 gtggtgatcc acagtgctgc atgaccatct tctctctgca caaaaacaat tgttgcagcc

211561 ctgcattcgg gattcatggg acccctgagc aaactcctac acctgagaga actgaggcac

211621 cctgcctgct aggggaccct gggtgtgtga tgaggtcccc tctaggttag tgtctaccag

211681 gcctggtgta ggtccagggc ctcagacaca agtctgcaat gtcgctgtct gtcagtctgt

211741 cctgccaggt gctctgttag ctgggaagat gtttgctctt tctagctttt cttgtcatca

211801 gcctgagtgt gatgactgca gggcagggag tcatgaacag catatccact gagatccccg

211861 tgagtataac atgcaggtgt gttaaaggtg cagaacactg agtcttcctc cagacagagg

211921 ctgagaaacg tggtttttag tggtggtgac taagaaggac acggggcttt gctgtgggcg

211981 ggggagacac cttatactac atggaggtta atgagcagag agagggcact aggtcaagcc

212041 ctgcagaagg agttggagct tccaactcag atatttcctg gggcatcact ctgggtgtct

212101 ccaagcaagc ggggccaggg agtggtcctc aggtgtgatc ccatatctgg acacttgaca

212161 tttacatgtg ctgacaggtc ctgggacagg gcccagagtt tgtgatgtac tttcacctcc

212221 tggatcccca gactcctgcg ggaagcctag gaccggttct ctgctcagag gccaggggga

212281 tcccactgca ctctgaggag ccagcctgca gagctggggg actcggccgt gcccctctgt

212341 gccagcagct ccagcacagg gtggtcctgt caccccttca tgcgcacaca ccctcatcct

212401 tctccctcca tgcagctccc gcactctaag cagcccttct ttgctcatca ttcaccaggg

212461 aacacagagt tcttgacatc agctgtgctt tgggaagaca aaacaaagcc ctgcctggtg

212521 acactcagtt tcctgagcat tgtactgcta accaaaggct tgctggttcg aatcccagtc

212581 agggcacatg tctggattgc agaattggta ccctcttgag gcacatacaa gaggtgtcct

212641 atgcatatct ctctcccaca tcaatgtttg tctccttctc tttcgcattc cctctcatct

212701 ctctggaagt aaataaataa cataattaaa taatgagggt tcccagaccc agctcagtgc

212761 tccaaggcac atactgaaca tcgtggtcac aaatagtcat cggtaggact gtgaggctcg

212821 gtccccaaca cagctccgct ttgcggggga gggggagtgt gcccgctcct caggaatgca

212881 cacaacaccc tcttcctgcg cacccttctc tgagccgccc tgccaccttg ggggctccgt

212941 agcaacttct ctaacgtgcc ttcctgttgg gagtgaaggt cttcacagct tctgtgtctt

213001 tgactgagaa tcaccatagt gataccagaa cggcgtcatg ccctctgcca ggctgcagga

213061 gcccacactc tattgtgttt tctttttccc tccactccat cctgagtgtc aggctgcagc

213121 gtgtgtccac gacctcttgg tttcatcttg accccatggg tcatcctttc tagagatgag

213181 ggtttttctt ggtgtcctct cattttgact tgaagtaatt tccctgaatt cttttaactc

213241 aatgatgtga cattttctag ataatatgcc ttacttcttc ttatttattt tatgtgttat

213301 tttgggatat attatctgct acaaatatga ataatcgagg ccctgtgtgt gggggcagcg

213361 gcagacatct ggcaaatccc cgcccatggg cagaggggac aggcctttgg atcatgggct

213421 tctgggagtc ctcagttctg aggtgggact ggatgtcaca gactcagtcc tggtccagga

213481 gcctgacctt gcacagcagg taaggacagt cacagtctct gaaaacccac ctaaacactg

213541 tggtagccat gacaccagac ctgctcaccc aggaactaga gtccttggtg ggggacgttt

213601 cctggtcgtg tgtggactgt gctcgggtcc ctgggcctcc tgggaggggg gaaggcctgg

213661 gttcccaggg cagctgcctg tccttgggaa gccatcaggg accccttccc agactgtgtc

213721 cccatttatg ccactgccca gcacctgacc ctggccgctc tgcttcttta ggacactgtt

213781 ttgtaccagt gtttgcttgt ctcccacaac ccgccgcttt accgcgaatc atctccgtgc

213841 tcacagagca ggtgccccct gtgcgggctg ctcctcccgt gtgggctgtg cgtgggggcc

213901 tcagtcccag cgtcctcagc tccccattgg atgtctattc tccacgactg ctcaccaacc

213961 gtatgggtta catttcactt gggtatcaat tttaacattt attcacacac atttgactcc

214021 aatcttgaag gtaatatcat tatcttttca gtcttttgct tatctacatt ttggaggtaa

214081 ctcaatatac cttttttctt tttttgcatt atgtttattt tgagcaatgg agataaaaga

214141 aatgctttaa ccttcacctt gtgtgttttc cttcctactt ggactagata ttatgtcccc

214201 ccattcccct tctctccctc tgcctttcag aggacaatgt gagggaagcc ggtcccagac

214261 tgcagctgca gatgatgtga gagtgctcca ggggaacggg gtggggtgtt aagcatggaa

214321 cctgaaagaa atagggtcac ggtgggtaat aaagttggga ttagggttaa gggttttgag

214381 ataccatata aaggatagtg cttctggaat cctctgcctg tagaccaggc agacaagccc

214441 accatccctt gggctgtggc agaagacagg ataatgaaat gtcagttact ggccaccaga

214501 tggcgctgtg acaccactga cctggaaggg gctgtgggga gcgtctggga gagcaggaaa

214561 gggttaagaa gccctgggca gggaccaaca ctgggcagat gctgaggcag gtttcagttg

214621 tgggcagcct ccctacagct gtggagtggg tgggaggtcc cgctaaacct taacagatcc

214681 atggttgaag gacttgggca aggcagccct gctggcaggg gcaggtgcag agagacaatt

214741 aactgtcccc tagcactgtg ggagaccagg agtggcccag gttaggacag tgaagggacc

214801 tcagggctca tgtgtgcagg gggaagcagc tggagtcact gaggggtcct aggatgcccc

214861 gagctggcac aggctcctcc tgcccaggga gaggggacct gcctgcacct caggaggcac

214921 caaggaggca actccttcag cccgaagagc aagaagggat gtagggccag cagcaagagc

214981 tggtacttag ttcaggcagg ggcaggcagg gacaatgttg aggtacttgg ggatacaggg

215041 aagttgcagt gaacccttag ccatggcgct gggcagcaca gggagacgtg tatagaatct

215101 ggaccctggg tcccaccaag ggttcagagc accagggggc accctccttg gggagttcca

215161 tcacagcagc gacagcatca gaccaatgac gtcatagcaa atgctcctat cagcacacgg

215221 agcacagaac tgtagaccgc tcaccccagg gggccctgcc cagagctggg agaggccctg

215281 ggcagctgcc atgggcccca ggctcctctg ctgtgtggcc ctttgtctcc tgggagcagg

215341 tgagccccgg gcactgtgtg taagtctgga cacgcagtca catccctgac acacaagcct

215401 gggggtggtg gctgcctctg gaggctccct gtgctctgcc cccagctcct ctgtgtctcc

215461 tcccaacagg cccagtggac tctggagtca cgcagacccc aaaacacgtg gtcacagcaa

215521 gaaaactgca agtgacgctg gggtgttcct acatctctgg acaccgctct gtgtattggt

215581 acaaacaggt tcagggtcag ggccctgagt tcctcattca gtactacaat gggcaagagc

215641 aagaaaaagg gaacatcccc gatagattct ccgtgaggcc gctggatggc gaccgctccg

215701 agctgaccgc gagctcgctg gagctgagcg actcggccct gtatctctgt gccagcagcc

215761 ctgacacagc cctgcaggcg cggcagcctc ctgcacagaa acactccgcc ccactcagga

215821 agcggcagct gccagccaga caggctcagg ccctccccag gcaccgttct gctgcttaca

215881 gacggggtgg ttgtagtgct tacagacatc gtgcagcttc agtatccttg ttcctgttta

215941 tatacgtggt gggaagtaac gtttctggga ctcacatttc ataagcagag gagcgagaaa

216001 acactgaagt ctgttcctca acacctgtga cttgccccac tgcccgctgc tcccagtgct

216061 gtgcccacgt ggcttttttc ttccaggttt aggtgaacag gcctggggag atgaagtgcc

216121 atcagggatt gtcacctctt ccctgacagc ttataagtgt ttaaacaagt cacccaagtg

216181 tgttccatgt gcatcacatg tccctgagtt caaggggcct gacagaggca gtgatgtcac

216241 tgtgggagct gccctgtggg gacaggggac gtccctcctc ctctgctcct gctcacaggg

216301 acctgacccg gggaacctcc cgttcctgtc ctgaccctgc catgggcacc aggcccctct

216361 gctgggcgac cctctgtctc ctgggggccg gtgagtcctc agaaacccag gcagcctcag

216421 tgtgtgtgtg tgtgtgtgtg tgtgtgtgag agagagagag agagagagag agagagagag

216481 agatgattac atttgttttc ctctttctgt tttcaacttc tatgtcccca ggtcatacag

216541 aagctggagt ctcccagtcc cccagttaca aggtcaccaa gaggggacag aacgtgacat

216601 ttcggtgtga tcctatttct ggccacatta gcctatactg gtaccaacac gtgccagggc

216661 agggcccgaa gttcttggtg tcctttcaaa acaaggaacc tgtggactcc tccgggatgt

216721 ctaacgatcg cttctctgct gtgagacctg ctggctccta ctccgatctg agcatccacc

216781 ctgcagagcc tggggactcg gccgtgtacc tctgtgccag cactgccacc acagtgtgtc

216841 accgtcaccc tctccctgtt cacataccct cctgtgctcc cctctgctca tggttcccag

216901 gcttccttgc aagggaggtg tcaacagcgc tgctgcataa gtggccacat gagggcgctg

216961 tggatccatg gagtgagatg cagatggtgc cctgtgggag agggagggtt catgggaaga

217021 atcttgggtt gattcctgca tcaaggagat tatctggtga atcagaggct cactgatctt

217081 atcttctcac attaactgac aaccaggtgg gtatgttata gcaaggagcc atgaagtagg

217141 ccttgcaggg agttaagttt ctcctggagc ctgtgtttgc tggagagtgt gatggggctt

217201 cagggaggag cctggggaac ctgagccagt gtagagcaag gagcatctgc tcaaggcaga

217261 gagaaccttt gctgatggcc acccgccctg taccctcggg gcccaactca gcacagccag

217321 ctgcacctgc tcgtaggaaa atggtcagat tcagggagag caggactagc tgagcttgtc

217381 ctggggtgtg agtacccacg agtgcctaca aagacagtga catcagaaca gtgacgtcac

217441 aagcaagtcc tccaaaggga gcaaagagag ggacaaaact gcattctcaa ccaaaggacc

217501 agagccctga gcagagacat ggccagttgg gtcctgcctg aatcctccag gggcacccgg

217561 ctcctctctt gcatggtcct gtgtctgctg ggtgcaggtg agtcttagcc caggtgggac

217621 atccctccaa cccagcattc ttgactgttc ctggagtgtc catctgcctc ctgggctggt

217681 ctcagctccg tctccttcct ccacagggtc agtggctgct ggagtcaccc agtccccaag

217741 acacctcatc aaaggccctg gtgggggggc cgttctgaaa tgccacccca tctctggaca

217801 caagagtgtt tactggtacc agcaggctca agggcaggga cctaggtttc tcattcagta

217861 ttatgaaaag caggagtacg cgaaaggaga catcccagat cacttctcag tacaagtgtt

217921 cagtgactac agctccgagc tgaccatgca caccttgcag ctgggagact cggccgtgta

217981 cctctgtgcc agcagcctag acacagccct gcagggccca tgcctttctg tacccaaacc

218041 ctcctgtccc agctgatgtc acaggcagag ggagggggcc tcagctgagt ttcactgttg

218101 gtttttgttt cctttctaac tctctccctg ccctgctaaa ctcagttaga gccttcttga

218161 atgttctccc agctctgcca gcagtgtgaa atgcccagca catggcagga agccctcttc

218221 tgtgacagag tggagttctg tgactgtgtg ttgtggtgcc cggtcctggt ggacactgtg

218281 tgtccacttt gcagaggggg gagggctcag tacaccattt cctagacagg gagctgcagc

218341 tgctggacct tccaaacgtc acatgcacgt gcatcctcca ttctcatgcc tctgaagtca

218401 acaagcccct ggattccatg gtcctgcctg ggccttggtc catgggatag aacagagcac

218461 agtctcttcc atttcaggac cctctgatct gggatgggga ggtcacccct caggtgtagg

218521 gtacatggct gattgcctgc caactgctag gacctagagc ttcaagccag aggacatgtg

218581 tctgtcctgt catgcttgca gacagagcga ggtcatgata tccccctaag agaggagcta

218641 agtcacccgg ggaatgtcca gcccatcagg agatcctagg agggaagcag aagccctctg

218701 ggactgtcaa ggagcactct gggagctcat gcgggggaat gcgtgtttct gaactactgg

218761 acagggtagg ggatgggggt ttctcagcct gtcctcagct ccaccacttc tggtcaggaa

218821 atctcaactt gctccatctg cccaggtgcg gagctgcaga caacagctga gtgtcacatc

218881 cccctggcct ctgatttttg actccgtgtc ctttctcact gctgggtggt tgttgttgcc

218941 catcagcatg acatcaaaag agcagtcaca gaaacagggt cacgtcacac tgaggtatga

219001 cctgtgtaag aggtagcagg acggggatct ctggctgaat atctaatcta tgtcactaac

219061 tctctttttc acagaaagcg cagtcacagg gccataggta tctagtcccc ccgggcctca

219121 gctggaacca ggggagacac tcacccgtga gcatgcagag acctgagctt gggtggctgg

219181 gctcccacca gtgggtgaat cgtgagacaa cgtaaatcaa gtctcagatt ccaatgaccc

219241 tgaggctgac actgtgaaaa ctttctgggt atcagttagt tcctttaacc aagaggatcc

219301 ctgtgggctg acatcaaaat tatccagaat atatgcaaag agaatcacta gcagacagaa

219361 tgtgaaggtg tattatcagc agagaaatct gccaaagcac caaaaatggt gttaaaaatt

219421 atagcagtat tcaagtaagg aattgtatca ttatgcatag aggcatgaat ttcatacctg

219481 aaggtgggaa aaaagaatgt aggacttatt agatttcttt aaaaacagac ttacacattt

219541 ttcccatgct gcactgctca gctttctctg tgtcactgat tcgttcctgt gggaggggtg

219601 tggcaggtga gctctgtagc tgaagcatct gtgaaggtca gtgatgtcac tgagtccctg

219661 agaggccgag ttctatgtgc ccaaatggaa caggaagacg tgagattctg tcctggaccc

219721 aaaatggtca ccaggctctt cttctgcgca gctgtttgtc tcctgtgggc aggtgagtgc

219781 aagcgggctt cctgccctgg aattcccagc cttcagtcca agccttcgtc ttaggatgac

219841 aacctcacac tatgtcactc tctctcacag gacacctgga tgccaaagtt acccagagtc

219901 caagaaacaa ggtcacagtg acaggaaaga aggtgatact gagttgcaac caggcttatg

219961 accacaacta tatgtactgg tatcgacaag atccaggtca cgggctgagg ctgatctatt

220021 attcatatgg tattggcagc attgacaaag gagaggtccc tgatgggtac agcgtctcta

220081 gatcagatat aaagaatttc ctcctcacgc tggagtcggc cacccccgcc cagacatctg

220141 tgtacttctg cgccagcagt gaatccacag tgctgcatgg ccacctcctc tctgcacaga

220201 aagtgtcagt agaaagcctg aggttgcact ctgattcctg gcccaaaccc aaagaagtcc

220261 ctgccagtgt gggggcctta cggcactggg cactatgacc ctcagggccg aagtcctcct

220321 ctgtaccagt ctcagcctga ctggagacca gccacccgaa cacacttaac tagctgattc

220381 tgccttctgc agcgctcgcg ctggcgctct ctctctctct ctctcttccc gtttgcagaa

220441 aggaaaatgc atcacaagtt gagctgcatg aggagtcccc tgataatctt aaagtcccca

220501 cactctccta ggggcttcag tgacttcttc gtttacgctg gtccccacgc tctattccct

220561 tcaacttata gtcataaact ttccttcaga tggatctgcc gctggctgcc tttctcctat

220621 gggacaatag ccctacaagg tcctatagtg tttgcccttc accttgccct tgacctccac

220681 ggtcttcttg tacaagtctc ctctgctctc cctgtgctag attcattcat ctgcattcag

220741 tttctccctc aggtgtgcca cctcagggat ctgttttaca gattatttct tcagcatgca

220801 acctacgtcc cgccctctta tctctaggtt tccccttcat tccttatgtg acaatatatt

220861 ttaggcatac ttactctcaa atgtgaaaag gaaaacttag attttactag gagaagggaa

220921 tgaaatatat ttaggcattc atttaggcga gggtgttttt agcaaagaca caaaagagca

220981 caaacattta taaagcatat aaatacttat aaaataatgt aatgtacaaa tattgacaaa

221041 catatttata atatcagtgg tattaaatta tacccttgta tatcaaaaga caccacaaca

221101 caccccatga gacacagaag ggtaagttgc aggctgggaa gggacatgag gaagggacac

221161 catcctaatg tgacagaccc cttgtggtca ccacctcccc aagttgctct agatccccag

221221 ccttgcttgt aggtgtaggg accacacaca cacacacaca cacacacaca catacagaga

221281 gagagagaga gagagagaga gagagagaga gagagagaga gagagatgtg agagaaaaac

221341 atccatccat tacctcccat tcacacccca accatggatc aaacctgcaa tctaggtatg

221401 ggtcctgatt gggaattgaa cccacaacct tttggttcat agaatggcac ttgaaccaac

221461 tgagccatgt ggccaaggcc attgctctct ttttaaaaaa ccatgaaatt cacttgtaag

221521 tcatgttaga ggtgtaggtt ttttcagatt ccagaaggtc tgttcaattc atattgccaa

221581 tactgtgtcc accctggtat tttcagggat cagagagctc tccacctttg caggtaagat

221641 atacgacacc cttcccctgg aagccagcac ctcggaaatg aaggctccag ttcattgtgc

221701 atcatggagg ttggcgaaag ccaagaggaa agtggtctct gacctctgga cagaacaaaa

221761 gcgcccgact tcagtctgcg ccccgggctt catctcatct ctccaccttc aagcgtgccc

221821 gttggttccc tgtgttccta ggctacagtg gtgggaatta ccgcagcttc cactttgtct

221881 aagctcctgt ggctggggga atggagaccg ccttaaaaga gaaacatccc aggagacggg

221941 ccatcacaga taacgcagag gtcattaacc gatgggcctt ggccatgtcc cagcagaaac

222001 gtccagcgag ggtcagagga cggtgatgga gatgtagagg tggggcgggg ggggggggtg

222061 cgccccatca ctcgggccct ggaacaatga gcggaggccc cttggatgag cttacacaca

222121 gcctgagaat atgctgaaca aacttgtagg tatgttttag tttacaacca ttggttttta

222181 aggcggagca tttgtggaga caatgatgtc actgtgggga ctgccctgcg gggacaaggg

222241 acgtcccacc tcctctgctc ctgcttacaa ggacctgacc tggggaacct cccactcctg

222301 acctgaccct gccatgggca ccaggctcct ctgctgggcg accctctgtc tgctgggggc

222361 cggtgagtcc tcagaacagc aagatctctg cattttatct ttccagggat catttcaatc

222421 atttccaact tcctttgtct tttccactca cagaactcac tgaaggtggc gtcacccaga

222481 cccccagaca taagatccta ggaaagagtc aggcggccac tttgtggtgt aatcccattt

222541 cgggccacca aaccctttac tggtaccagc agttccaggg acagggccca cggcttttgg

222601 tccgctttga gaatgaagaa gcagtggatg actctcagct gtctaaggat cgattttttg

222661 cactgaggcc caaaggagca gactcaactc tgaagatcct gcgtgcagag cggggggact

222721 cggccgtgta tttctgcgcc agcagcttag ccacagcgtc acagagacgc gtccttcctg

222781 tgcacaaacc accaggcttc ccctctctct gctccgctcc cggcagctct gaacacaggc

222841 ctcccctggt tcctcacgca cgggaaagaa gcaatctaga attcatgaga aaggaggatg

222901 tgaattattt cttgaaacac cagggctgaa aataattatg actcagggat ctgaagcact

222961 ttttggtgac aattcaagga tgcaaaactg aaagtgactt atgtctcacg gagtcagccc

223021 tcaggcgcta atgaggaatc ttctcttata aaaattatgc agtcaccaaa aatgtacaat

223081 ctactgtaat aataatatag atggtgattc tgggagagtg gcaaacagca ggctctccct

223141 ttgtggggct tttaccttct aaatgatttt aaatgatatc ctttgctttc taggttcctc

223201 gctgactttc ttatcaactt ttctgcatca gagtctcagt acatggtatg tgtttggaaa

223261 acattttttt ccattttgtg gaaatgtgtt gataataatg atgttgttga tgatggcgat

223321 gatttcagct attttattca gtccttttcc tacttcgaag gaatacacac aaatggatat

223381 tgtgacaacc agtctgaaat ctcagctcag aacgaatcct cctttctccg gggtggtctc

223441 actcttcaac ataagcagga ccgctcaaca ttacatccag gccagagcca accagtttgg

223501 aggctgttga ggatagttcc agttgtctgc cctggtacat tgaaatagct ccttagtgta

223561 ttaaaatatt tcttagtgaa aacttctgaa acaatccatt gcccttgatc caatgatccc

223621 atctgataat actgtgcata cagggtgtgg gtctacaccc cgtactctgc tgtactctaa

223681 cttctgtact ctcacttctg cctggattct ctaagacgag aaggcagttt gaaacactgg

223741 ttggaactgt tgggaaagca gcacacgcag attttcctga aggtgttatc attgctgctc

223801 agcccctgct ggcctcactt cctccttgtc caaccaggac tgccagtcta atgtctgtcc

223861 atccacacct tgcctacccc tcatctcctc tgcccatggc cccctccctc cctccaatgg

223921 ccgtgtccga caaacctccg tcatcggtga gcgcagctac ccaccgtctc cttgccagag

223981 gttttatgtg cttgttttcc aatctgctac gttacatttt gtaattacta accccctaat

224041 aacattctcc tagatcatct ttgatctcct taaacgcagg aagcagtggt tttaggggct

224101 gatcattttc acagctgaga tctctgtggg gctgttttaa ttgattttct gctgcttctc

224161 agtcgtgctg tttgtgtttt catggtgttt gtatgttttg tttatgaatc agattatctt

224221 tgacttggtg ccagacacgg gctttctaga tatatatata tttttttatc tcggatgggt

224281 gagtctttct ccaaagagaa ttttattttg cttttgccaa atgcaagggg tatcagcaag

224341 gcggggcaac ctagggcgag gtagcattca aggcgtgagg tgtcccagat cagccagaga

224401 acacgtttac ctctaggtca tctctgcatt gcgtttgtct gttttcccag tttgatacat

224461 ttggtctttg acctttgtcc ccctgtcttg tgagcaccga gactgggtgg gccaatgccc

224521 tcggggcaaa gggacagctg tgcccctccc tcagtactca ccctctccag gatcccagcc

224581 tactgtccct ccctgtattt ttagagcatt aatttaagtt ctttatttta ttttatccaa

224641 ctatttagac aatcaggaag ggttgctttg agtgctctcg tctgtactta ctaagattaa

224701 ggtcaaaatc atttcatgta aaattttaaa taaaaaataa tgtcagattc tgagtctatc

224761 atttctttaa ataacttaaa tgacatagag atgtatgtca ccctgcatga gatttatcaa

224821 ctttaatttc aaccaaaatt taaaaaaata aacttaaatg gtatttatta tcagattgag

224881 atagctgttt gtttttgtca ttctattatt ggaatgttta cttttttttg acttttattg

224941 tattccaata aaagtcacat tctgttgacc tctgacatgc cattgaacca tgtgacaaga

225001 caggatataa agcagggact gaatatttag actcagtcag aaagcataga agagacttac

225061 aatttgtgta attgccagtg cagcacatgt gctttttaaa tgaaggtgcc cgggacaagt

225121 ggttggaact gggaagaatg gctgatgcac ggccacctgc acttccccac gggaacccag

225181 tctcgtgggg gaaaccatgc ttcccacttg gggggcttcc ctaaagaaat ccccctgttc

225241 tgattttcct gttcctcgtg gaatcgctct ctgggaaggc acgtaaagtg ttttgtttaa

225301 tctcttcacc ttactttagc ctctatcttc ttgctttctt actggccacc tagagaagag

225361 ataggctgtt tggtgttttc tcaacatgca ttaaaattag cggggaactt tctttcctta

225421 tggggtccac ctgtggtttg gaagggaggg aagtgactta aattctgatt ctgcgtagtg

225481 tcagccagcc ccctcgggtt cacaacaggg ccttggtagt caccaatatt aaaattgata

225541 ttatcttcac atatctgact ccaatgtcca gttctcacgt ggttccatct gaggctacaa

225601 tattacagtt ttatttattg atcctctaac accagtaaat cataatatca gtgcctcata

225661 tcccctgtat tcaatgaaac aaaatacccc ttgctttatg tcaagagagc ctgtttgtgc

225721 tttctgctca ctggacacta ggaggcgctg tggctccgtt gtaatctaaa atctgcaggg

225781 aggttcttcc ctggaggggg aggatggaga gaaaccctgg ggcggggctt agattaaagg

225841 agttgcagat ccgttgatgg tgagaatgaa gaacagagac caacacttct tgctctctgt

225901 gcagatgggt tttgctctgc atggagctct gcaaggcagg tgtgcttgca gtgggcattc

225961 ctccaagctg gcttagtttg tgcggctcag gacgctggtg gtgaagctgg cacatgctgg

226021 gagaagcaga caagcgggag tgtcagagga gctttagcga gcccagccca aaccagccct

226081 ggccagccca ccacctgctc tgctgttcag cagaaagtag cagggagaaa tgccagtgct

226141 cccctggacc acaccaatgg ggacttggcc ccaggagctg acaatcacag tgacatcagc

226201 agcaaatcca cttgtcaggc atggacaggg gtgcaccaga acaacagaat ttctccccag

226261 gagcttttac tgtgagcagg aggatgccac aatgtagtcc tgaatttgac cgtcacccca

226321 tgcttctgtg catcccttcg aagcttatga ggtctggaca gagagaagtt ttttcgtggg

226381 cttctcttga ccagattcca agttctttcc acgcaactgg gtgcctatca tgtactgtat

226441 ctagtttctg ccttttccac ggacactgag tcccatggat cctgtaaagt attagattcc

226501 tgttgctgct ctaaaaagtt atcacacatt ctgtagctta aagcaacacg aattgattcc

226561 ggagatcaga ggtccaacat ggccctaacc gggttaacac aaatgtgttg gcattgcctc

226621 gttccttctg gaagctctaa tggatgatcg gttttcttac ctttttcagc ttctccaggc

226681 caccgacagt ccttggctcg tggccccgcc atcttcaaag gctacaatga ccgtccaatc

226741 tgtcctcatg ctacatcgct ctgactctcc ctccctctcc tacgttttat tttagggccc

226801 ttgtgattac atggaaccca cctgggaagt cccggataac cttcctacat taaggtcagc

226861 tgattagcct ccttggttcc atttggagcc caactcccct ttgctatgtg agataacaca

226921 tcgcaatgcc agggattaga cacggacatg cttggggagc cagcagtcgg ccttccgcac

226981 ctaggtgcct ggtaacaggg aaggaacgat ggtcgttttg aaaacagaat aatggcccaa

227041 gttttatgtt ctggatccag cagaaaccga agaggcaggc ccaacaaggg aaacgggtcc

227101 caagggtcac accatttagc ctgcaaacta tctgctcctc agtccaggag tctcggtgta

227161 tgtctctgga ccgaccgtaa aatacggcct tacactgggc cttcttagtt aacatgcagc

227221 ctaaccaatt aggaattttt tgagcaaccc aagaaactga ggctcctctc agcccttcgt

227281 aacctctacc acgagaggaa gtgggtttgt ggagtccttt cctttgacgg ggaaaaggcc

227341 acgaagaaca ctgagtttct ctaggagtaa gaacgaggtt taaatcaact ctgcagtatt

227401 ttgtgtgcaa atcttccaga acaagaaaac cctctgctta ttgcatgggg gaggggtgga

227461 aaatgtagat tacaggcagt gtgcgttggg aatttaacag tgctctagga taaacaaact

227521 cagagaggtc tttcacggca aacaagagct tttattttat tttttatttt attttatttt

227581 ttaattttat tttaattgtt gctcaagtac agttttctgt cctttactcc cagcccacgc

227641 acccatccct ccccacctcc ctcccatttc caccccccct agttgttgtc catgtgtcct

227701 ttatacttgt ttataccttt cccattttcc cctgaaattc cctcctctgt cccctttggt

227761 cactgtcagc ttgtcctctc tttcagtgtc ttagggtata ttttgcttat ttgtttgttt

227821 tgctgattag gttcctgtta aaggtgaaat catatggtat ttgtctttca ccgcctggct

227881 tgtttcgctt agcataatgc tttccagctc catccatgct gttgcaaagg gtgggagctc

227941 cttctttctt tctgctgcac agaattccat tgtgtaaatg taccatagct ttttgatcta

228001 ctcatttact gatgggcact tggctattgt aaattgtgct gctaatttag ttctcaggac

228061 atcacatcag tccctgaagc cctaacaatg caggggatag gcttgttttt catggcgagc

228121 tctgaaccca gctcctagct aactactggg ggatggtgag tgtgggtata tgaccagcag

228181 catcaagcca ccagtgagag ggatgtgagt ttcagagcag ctggcttgca atgggatgat

228241 gaatttgtct ttaagtactt gaagttcaaa tggcaatagt tggaaggctc tataaaaggt

228301 agtatggaga aggcattagg agctgataac attgataccc acaggggacc tctggatgcc

228361 caaatctgta ggccaggtca gttaaaatgg acttcagtcc tgccaaagat atagccttct

228421 ttcctgaatg gagcccctga cttgggggta ggaggtcatg cagagtgatg tcagcatctt

228481 gtagagataa aatgtcccag aatgcacttc tctgctcatg ctcacaagga ccctgggccg

228541 ggctgttcca catctaccac actttgtcct gggaacctgg gacctctgct gagtgggcct

228601 ctggctgctg ggagtaggtg agtccacaaa atatttcgca tcctcatgct atgtctccct

228661 actgtgactc caatgttttt cttattttgt cttcaaagta tgtcctcttc catagacccc

228721 acagagactg gagtcaccaa gacaccaggc acaaggtgac aacaatgaga caaaacgtaa

228781 ctgggagctg tgagccaatt tcggaccacc atgcacttct ttggtgcaga ccgaccgcac

228841 ctcagggact ggcaattctg acttatttca ggagtcaaac ttttgcagat gagacagggc

228901 tgcccaagga gcgattctca gctcagatgc ctgatcgatc attgtctaat ctgaagatcc

228961 agcccacgga acccagagac tcagccgtgt acctctgtgc cagcagcgta gccacagcgc

229021 tgcacaggca ccccctccct gggcagaagc cccctgcttc ccctgctcgc atcagctgcc

229081 ccagtcacat aaaggtctcc ccagctcctc tctcaccgag agaaatgagt gaatttgggg

229141 catggcaaga acagaaataa tgaggtatca acagaaaaac ttgagtatga taaaatgtag

229201 gaaatgggct tcagggattt gctggcacat tttctgtggt ctcaaaactc accacacctt

229261 gagataaacc tcagttaccc acacagtgcg tgtcccggcc ttcctgcacc agcctctcca

229321 tctgaaccgt aggagctcct ggtaagggct cctcagccct ccattctcct ctccctctaa

229381 gctgctaaca gaatctaccc tccacacaca cacataagtt tgattgtatt aaaacctctt

229441 tactttgatt gtggacattc ctttataatg ttaatattcc tttatattta tatgggactc

229501 atttcaatca cagtatctca aatgctggta aagcagatac tttatataca ttagatggaa

229561 gatatgactc ttgaatacca gactttgcct ctgtgccgcc tacgggcagc ccaggccaga

229621 ctgcagaggc tgccaccgcc gagggctggt gtaggactca cagatgttca tggggcctct

229681 tcagtggcca gatctggaac gacaaacatg gacttcacat tgggatgtct aaaggcctgc

229741 aggcctgtcc ggtcttatgt agctctcttc cctacctgat cctatgtcct caacacccaa

229801 acctacattt tatattgatt tctgccagaa atgtccacat tatccctgca gtcccttctg

229861 aacagttatg attactctct tctgtggtct tatttacatc ccttctcaga ccctttgaga

229921 gcatctttgg caactggtgc aaaagacccc tgtgttttga gtgcccgaga caattaggtt

229981 tacctgagtt ctgccatata gccaattcca ctgtcacgtt ctacttgttg ccgttcagag

230041 accagggctt tcaagatgga agcagggaag aatgaaattt ctgatgtgtt gacgctgtta

230101 ccaggcaggg gacctggccc tgagcgggtc tgcctagggc agctggtagt gtgtgggttc

230161 tcgacttcgt gcaggagaga tgtcacaaca ccagtccatg tgactatgag ggtacattta

230221 ttgaagatgg ggagagtgaa acagggaagg gcttagcata gaagaagcaa cagaaaagcc

230281 cgggctgatc tgccttaact cactgggaag tcacagaaag gacggtttgg ggggcaggtt

230341 caaggggtta gcctagggcc agctgcctct gcccactgct cccctggttg caagtctcat

230401 ggagaccctt agagtttagg agatgtgtgc ctgtggggga agcagagaag gaggaacagg

230461 gggaagggaa tggcacgggc tactccccag tgggagaaca cgtgctgagt ccttagactt

230521 gagtttttat ctcgcttttg caagcaagaa cctaggggag gtctcagggg agggtgtcag

230581 cagaacactc atcacctttc caggtgccct ctttcaagat catggtctct gcttactggg

230641 cagtgccagg acaggggtcg tcagtccttg cagctggtcc tggcgtcctc ggccacctgg

230701 tttttctgct tttctgggcc tggagctgaa atacagccga ggcctggatg ttatctccag

230761 ggaggtgacc ttctgtacct ggctgcatgt tgctcagcca gtttgctcag ctctctgtct

230821 ccgtttccat ccatattcca cttgttgtgg cttactggcc tgcctcatta agacataatc

230881 tgcttctctg cctgatttca gtgcattctt tgtcctcctg ggtcctccct gcagctttcg

230941 gaccagtccc actccccagc tttaacaccc agctgttcac acatatgcca tcagaatatt

231001 gccttttgtt gctgaactgc tttaccatca ggcccctcct cattcatgtt ccctcccact

231061 actactccca tcaccgctgg tgacatcatc atcttgcaga caatttatat acgggtttgg

231121 gtcctccatc ccttgacctc ctcacatttg atgatttttt tttgcctcca atccttttca

231181 ggtgcacttt gtcattctgg accttggaat gaacaccaaa caagctcctc cccaaatttc

231241 tatttcaatc attccctgct cccaggcgtt ccttcttcct tcccatctca ccaacccagc

231301 atcgtcctct tatgatggtt ccagccactg tccccagcag cttgtaaatc cccttgttct

231361 taattctcac accatctaag tcaggtttaa agtttaaaat cataacctca gccctggata

231421 aatccactcc atctgcacaa ggcatctggg tgtgttttta aagattttat ttatttattt

231481 atttttagag agagggaagg gagggagaaa gagagggaga gaaacatcaa catgtgaaga

231541 aaacattgat cagttgcctc tcacacactc cctatttggg gccaagacct gcaactcagg

231601 catgtgccct gaccgggaat caaactggtg acccttcact ttacaagata aagttcaaac

231661 aactgatcaa ctccggtagg ggccagggca tctgtttata taccttcaga tgaccatggt

231721 tgtcaatcaa aacctttcca agaatcttgg taaaaccccc aattcaataa ggaattattt

231781 tgattttgtg tgtctgggag aaatggattc tcttgaaaga agtatgaaga acttgagatt

231841 tagaaagtac gttactgcta cacaggacta gcactgccga tggcacaccc atgcagaaaa

231901 gctcagtagg tggtagatgt gtgatatagc gggagacaaa gagtggttaa agcacagggt

231961 tgtgtgaggt gacagctgat gaaagtcttg gagactttaa atggtccaaa caaccctaaa

232021 acacatgatt tatcccaatc aattcttctg agaagattaa tatttacctt tgggaggcag

232081 acctctgaga aatgacagcg tgatgtcact ctgcacctta tggagcccag aggaacaaga

232141 agccctgccc ctctgtcaca ctcacaaggg ccttatctgg gtgaagtttc ctcctgcccc

232201 gccctgccat ggccaccagg ctcctctgct gggtggccct ttgtgtcctg ggagtaggtg

232261 agtccccaga gattaagaat ctgcattttg ttgatattcc cagttctatt tccatttatg

232321 ttccttatcc tgttcctaaa ctctgtcctc ttccacagag ccctcacatg ctggagtcac

232381 ccagacaccc aggcacaagg tgacaaagag gggacaagga gtgactctga gttgtgagcc

232441 aatctcaggc catgacgaac tttactggta cagacagacc tcggtggaag gaataaagtt

232501 tctgatttac ttcctcaatc aaagccctct ggacaagaca ggaatgccca aggagcgatt

232561 ctcagctcag atgcctaata agtccctgtc cactctgacg atccagggca cagaacccag

232621 ggactccgcc gtgtacctct gtgccagcag cttacccaca gcgctgcaca gtcgccccct

232681 ccctgtgcag aaacctcctg cttccccttc tctcaccagc tcccaggtgt cctgagtaaa

232741 agtctctctg ctcttctcac accaggagac acacgtgggt gtgggacatg acaaggccag

232801 aaaggataag gtataaagct agaaatccca gcctagaaaa caatgtagga aatggctttg

232861 ggggttcccc gcaagctgtc tttggactca gctcttacca cacctgggag atacacctta

232921 gtcacccaca ctgtgtcttt tcctgccttc ctgcaccagc cacttcatct gaatggtagg

232981 agctcctggt aagggctcct cagccctcca ttctcttttc cctctaagct gctaatagaa

233041 tttccctcca gcacacaaac acgtgctttt ttttttttta aagattttat tcatccattt

233101 ttagggaggg aagtaggggg agagagaggg agagagagaa agagagagag aaagaaacat

233161 caatgtgcgg ttgctggggg ttatggcctg caacccagga atgtaccctg gctgggaatc

233221 gaacctggga cactttaatt cccagcccgc gctcaatcca ctgagctatg ccagccagga

233281 aacacgtgct tttttaaaaa aaggctttat tgatttgtag agaggggaaa gggagggaga

233341 aagagaggga gagaaacatc agtgtgtggt tacctctcat atacacaacc caggcacgtg

233401 ccacaactgg gaaacaaatt ggccaccttt gctttgcagg ataatgccca atacacggga

233461 gccacactgg tcagggctca tataagcttt ttaagttttc tcatgtaacc aaattccact

233521 cgtcgttgtc ttccatgttg acacttaaat gctctgacct ctaagatgga aatgcaataa

233581 aatttctacc gtgtttatgc caagagataa aatcaaacat atggtgtcct gttcagtgcc

233641 ttgtttatcc tcctgggtct tccctgctgc tcccacacca gtcccctctc tccagcttca

233701 ccacccgctc ctcacacacg tgcctcagaa caatgccttt tgttgctgaa cttactgcta

233761 cctccctacc atcagatccg tcctcattca cttcttctcc ccaccagtga agctgtcacc

233821 actggagacg tcgccatcac tcagacactt tctgcagggg cttggtgtct cagcctcctg

233881 acctcctgac ttgcaataat tttcacctcc aacacttgtc agatgcactt cagtaattcc

233941 cgtctgtggg atgagacacc aatgaccccc ttccagaatt tccatttctg tcatatcatt

234001 ctcttacctt atcatattac tccttagcgg tactaaatca gcaccatcct cctaagattc

234061 ctttcgccac tgaccctcag ttcatgtact taacaacttt taaactcata tgttcttaat

234121 tctctcattg tctaaaccaa gttcaaggtt taagactcat ggggtcaacc ctgacaaatc

234181 cagctgcctc taggaagtta tggatgacaa gtcgggagtt cctgagttga cagagagcgt

234241 tcggcagtga ccacacacag cctggtcacg ggcagaaatt ctctgatcct cgctgaagag

234301 ccttccattg gttcaccgct gtgcacagga agtgcatagg tcaataagac tttttgctta

234361 catttaaaga atctatcttt tcacctgatg tgttagtgtt attaaataac agaaatgtta

234421 taaccataca gcaattaaat aatgaagctc aaaatatagg acatgatccc atttatgtga

234481 aaatgtatct ataagaataa agcaagcagg caaatagagc tagatttaca tctactgatg

234541 tatatgactg cacattttct caaattttag aaattacaaa acaatataca aaagataagc

234601 ttctattttg ttaagaacat gtttttcata tatgccataa atttttacta taaatttatg

234661 agccaggaca aatctacata atcaagttct tcttcctgta gctctacaag gtgggattgg

234721 gggaaggaca gaaggtgtta cctagccttg tattacatat aactcttact cacgtattgc

234781 taaactaaca tgtgaaatat tactttgaaa tctcttccag ggcataggga acccttttta

234841 aaaaaggaga attctagcca tgaaaaaaga tttgtgagaa ctcagcacag tgaggggtgg

234901 taaaggctag agagacaact ggtgattctc cccagaaaga gcccaggggc agggccaggt

234961 cccaggtcac atgtgactgt cccagtgatc tgctgcacag aattgatgct cccctttggg

235021 aggaggtccc aggggcagtg acagagagca aagtcactct ccaccttgtg gagtccagag

235081 gaaccagaag ccctgcccct gtgttcaggc tcacaaggtc ctcgtctggg tgaagcctcc

235141 tcctgcccca ccctgccatg gccaccaggc tcctgggctg ggtggccctt tgtgtcctgg

235201 gcataggtga gtccccagag attaagaatc tgcactttca aaaatatttt cagttctatt

235261 ttcatttatg ttctttatcc tgtcccaaac tctgtcctct tccacagagc ccacatatgc

235321 tggagtcacc cagacaccca gacacaaggt gacaaagagg ggacaaggag tgactctggg

235381 ttgtgagcca atttcaagcc atacctacct ttactggtac agacagacct cagtgagggg

235441 actggagttt atggtttatt tcagcagtga aactattgta gacgacactg ggatgtccaa

235501 ggaccgattc ttagctcaca tgcctaatgg gtcatactcc actctgacga tccagcccac

235561 agaacccggg gactcagccg tgtacctctg tgccagcagc ttagccacag cgctgcacag

235621 ttgccccctc cctgtgcaga aacctcctgc ttccccttct ctcatcagcc cccaggtgtc

235681 ctgagtaaaa gtcttctcca ctctgaagtc atcaagagac atgtgtgggt ttgagacatg

235741 acaaggacag aaacaattag gtgtcagctt agtaacccca gtaaggaaac aatatgggaa

235801 atgggcttta ggttttcctc acactgtctg tgggctcagc accctgcaca cctggatgac

235861 agaccttcgt ttcccaaact gtgcacgttc ctgtcttcct gccctgccgc tccatatgaa

235921 tcgtaggagc tcctggtaag ggctcctcag ccttccattc tctgctcctt gtcagttgct

235981 aacagaccct catgaccaac aaacacacgc atgttttcta gagtatgtct tataaccaaa

236041 ttctacttgt tgttgtcctt cttgtcatcc aggggcccgg atcctcaagt tggaaatata

236101 ataaaattcc tgctttgaag ccaaaagacg aaatctgctt ctctgtccaa tttcactatg

236161 tactgggtcc ttcaaatgct acctacttat tctagaacat tcccctctct ctggctttaa

236221 tacccagctc ctcacacagg tggattagaa cactgccttt catgttgccc cgacttcttt

236281 accctcagac ctgtcctcac tcccttcctc cccaccactg accctgccac ccactgggga

236341 cgtcatcagc ctgtggacaa tttatacaca ggtttgcctc tcagtttcct gatctcttca

236401 cttgcagtaa tattttgtct ccaatcctat ccaagagcac ttccaagtct agaccatgga

236461 gtgaacaata aatagtctgc tttcaaaatt tccatttcat cattctgtgc tcctatctct

236521 tttcctttct ttcttcaatg caaaatattt cctacgattc ttatagtccc tgaacatcag

236581 cccatgtacc caactgcttt gaaactctca ggtcttattt ctcacaaaat ctacaccagg

236641 tacaaggact agtacgataa agtcaaccct ggttaagtcc aaccctcctt tcccagggca

236701 tctgtttatg tgccttcaca atcacccata cgtccaatct aaactctcct cggaggggct

236761 ggggaaaagc cgcaggtcag cctgaaatga ttcagatttg tgtgtctggg agtcagggag

236821 tgcactgaga ggagtgtgaa ggacctgaga ctggaagtgc tgttttggtg cccagatgct

236881 gcagtgccga tggaacacgc acgcccccat gccagggaga ctgcagacaa tggatacagc

236941 agggaacaag gaatggtggc agcacagggt gaatattgat gaaagtctta gagactttga

237001 atgttccaaa caaaccccaa aagagacatg acccattcca gtcaattcaa ctgaagagga

237061 ttaatattcc cctttgggag gaggacctat gagaaatgac agagagcgat gtcactacca

237121 agatacactc caccttgtgg agcccagagg aattagaagc cttgtccctc tatgcacacc

237181 cacagggtcc tcgtctgggt gaagcctcct cctgccccac cctgccatgg ccaccaggct

237241 cctgtgctgg gcagcccttt gtctcctggg agtaggtgag tcctcagaaa ttaagaatct

237301 gcattttgtt gatattctca gttatgttcc atggatgtcc catatcctgt tcccaaactc

237361 tgttgtcttc cacagagccc agagatgctg gtgtcaccca gacacccagg cacaaggtga

237421 caaagagggg acaagaagta actctgagtt gtgaaccaat ttcaggccat gctggtctct

237481 tctggtacag ccagacacca gggcagggca taaagttcct tatttacttc aacaatcaaa

237541 gtcctgtgga tgacacaggg atgtccaagg agcgattttc agctcagatg cctaataagt

237601 cattctccac tctgacgatc cagcgcacag aacccgggga ctcagccgtg tacctctgtg

237661 ccagcagctt agccacagcg ctgcacagtc accccgtccc tgtgcagaaa cctcctgctt

237721 cctcttctgt cattggcccc caggggtcgt gagtataagt tttctctgct cagctgtcac

237781 caagacacat acttggcttt ggggcatgac aagggcagaa aaaataaggt atcagcagag

237841 aaaactctac atagaaaaca gtgtagataa tgtgctttag gttttcctca cacactgtct

237901 gtgggctcag cactctgcac acctcaaaga cagaccttag ttacctacac tgtgcgtttt

237961 cctgccttcc tgcactgacc tccacagcta aaccataggt gctcctggta ctggctcctc

238021 agccctgcat ttttcctttc tctctaagat gctaacagat ttacccccag cacacatatg

238081 ttttttagca ttcaatcatg tagtcaaatt ccatttatca ttgtcttaat ttatgccatt

238141 aaggtgctct tccctttaag atggatatgc aataaaattt ctactgtgtt tatttcaaaa

238201 cacagaattg aatatatgat gtgcaatttc agtgcattct ttatcttctt gggtcctccc

238261 tgctccgtcc agaccagtgc cctctctctg gattcaatgc tcacccctca cacacctggc

238321 atcagaacat cgccctttgt tgctgaactt actgctcttt ctctaccgtc agatccgtcc

238381 tcactcacgt cttctcccca ccagtgaagc tgtcatcatg ggtgatgtca ttatcacata

238441 gacactttcc acaggggttt ggtgtctcag tctcctggcc tcctcatttt caataatttt

238501 tacctccaac acttgtcaga tgcacttcag taattcccgt ctgtgggatg aaaccccaat

238561 gagctccttc taaaattccc atttcaaata tctcattctc ctacctcatc atatgagttt

238621 tcttctcact aattcagcac catcctccta agattgcttt agcctctgac cctcagtcat

238681 gttcctaaca gcttttaaac tcatatgttc tcaattctct cattgtctag actgggttta

238741 gtggtgaaga ctttgatgtg aactctgggt aagcccatct tcatctgggg atccctggat

238801 ggcaaagcag gagtccctga gccactgaga gcattcggca gtgatcacac agtctggtca

238861 ctggcaggaa ttctctgatc ctcactggag agccttccat tggttcgctg ctgtgcacag

238921 gaaatgctta ggtcaatagg atgctttgct tacacttaaa gaatctgctc acctggggtg

238981 ttagtgtaat taaataacag acatgctata accatacaat aactaaagaa taaagctcaa

239041 aatataggat atgatcccat ttatgtgaaa atatatgaca tgcataaaga gagcaggcaa

239101 ataaacagag ctagatttac acttactgat gtatatgact gcatattttc tcaaatttta

239161 gaaattacaa aataatatat agaagatact tctcctttga tgaaaaaaga atatgtcttt

239221 tatatatgcc attaattttt actataagtt tatagaagcc acgacaagtg tatgtaaggt

239281 tataaatcaa ttccttttcc agtagctcta cgaggtggga ttggaggaag gacagaaagg

239341 tgtcacctag ccttgtgtta catacatctc tttccccctc gttgctgaag taacatctaa

239401 aatattactt ctgaaatttc ttgcagggca tagggaattt ttttaaagaa tttatttatt

239461 tgtttttaga gagagggaaa gggaaggaga gaaacatcaa tgtggggttg cctctcacac

239521 tccccatact ggggacctgg cctgcaagcc aggcatgtgc cctgactggg aatggaactg

239581 gcgacccttt ggtttgcagc cagcactcaa tccactgagc tataccaggc agggcaggaa

239641 tgtttttttt ttttttaaaa aggagaattc tcagccatga agaaaagatt tgtgtgaagt

239701 cagcacagtg aggggtggta aaggccagag agatgaccag tgatactcaa cacaaagaac

239761 ccacatgcag ggccacatcc cagtgatccg ctgcctacag ttaatgttca tctgaggagg

239821 aggtcccatg agcagtgaca gacagtgata ccactctcca ccttgtggag accaaaagaa

239881 ctagaagcct tgtccctcta tgcacaccca cagggtcctc gtctgggtga agcctcctcc

239941 tgccccacat tgccatggcc accaggctcc tctgctgggt ggccctttgt ctcctgggac

240001 taggtgagtc ctcagagatt aagaatctgc acttttaaaa atattttgag ttctattttc

240061 atttatgttc cttatcctgt tcccaaattc tgtcttcttc cacagagctc acacatgctg

240121 gagtcaccca gacacgaagg aacaaggtga caacaaaagg acaaagagtg actctgggct

240181 gtaagccaat ttcaggccat gatggtcttt actggtacag acagagctca gggcaaggaa

240241 tggagtttct gatttccttc agctatgcaa aacctctgga tgacacagga ctgcccaagg

240301 agcgattctc agctcacatg cctaataggt ccttctccac tctgacgatc cagcccacag

240361 aacccaggga ctcagccgtg tacctctgtg ccagcagctt agccacagcg ctgcacagtc

240421 gccccctccc tgtgcagaaa cctcctgctt ccccttctct caccagcccc caggtgtcct

240481 gagtaaaagt cttctctgct ctgctgtcac taagagacat gtgtgggttt ggggcatgaa

240541 agggcagaaa ggaaaaggta tcaacagaca aaccccagca cagggaacaa tgtagtaaat

240601 aggcttcagg ttttcctcac ctactgtctg tgggcttagc actccccaaa cctgagagac

240661 agacctcagt taccctcact gtgtgtgctc ctgccttcct gccctggcct ctccatctga

240721 actgtgggag cttctggaaa gggctcctca gacctccatt ctgctctcct tctaatctcc

240781 taacagaatg atccaatgac cacacaggca cacacacaca cacacacaca cacacacaca

240841 tgcatgtttt ctagagtctg tcttatacca aattccactt accattgtct tttttgttat

240901 tttgggacgc tgatcttcaa gttggaaata gaataaaatt cctactgtgg atgccaacgg

240961 acacgttctg ctcctctgtt caatttcagt gcattcttca tcctcctaat cctacctact

241021 gcttctagaa cattctgctc tccctggctt taactcagct cctgagaaca gggattagaa

241081 tattgccttt catgttgccc tgaattctct acgtgacatc tgtcctcatt cactcccctc

241141 cccaccactg atcctgccac cactagtgat gtggtgatca tgtggacaat gtatacatgg

241201 gtttggcgtc tctgttttct gacctcatgt ctttcccaaa agttttgtct ttaatctttt

241261 caaaaaccaa ctttgactct agaccatgga gcgaacaaca aataatctgt ttccaaaatg

241321 tccatttcat cattccatac tcctatctct ttctactttc tttctttaat gcaaaatctg

241381 ctttctatga ttcttatagt cactgaacat cagcccatat acccaactgc tttgaaactc

241441 ttatgtccca tttctcacat tatctaaacc aggtacaagg attagtgcta taatgtcaac

241501 actggttaaa tccaaccctt ctttcccagg gcatctgttt atgtaccttc ataatcaccc

241561 atacgtccaa tctaaattct ccttggaggg attgggtaaa agcctcaggt catccgagat

241621 tatttacatt tgtgtgtctg ggagtcaggg agtgccttca gaggagcctg aaggatgtga

241681 acttcgaagt agtgtcctgc tgtccagaat gtgcagtgtc aatggcacac ccacttggga

241741 atgcccagca gacagcaggt atgtgataca gcagagaaca aggaatggtg gaagcacagg

241801 gtcgtgggaa gtgaaaatag ataaaagtct tgggtcactg aatggtccaa atggccccaa

241861 gagagacatg acatattccc gtcaattcac ctgaagaggg ttaatgttca ccattgggaa

241921 gaggtcctct gagacaacac agaaagtgat gtcactacca agccacactc ccccttgtgg

241981 agtccagggg agcaagaagc cctgcccctg tgctcaggct cacaacagtc ttgcctgagt

242041 agagcttcct cctctgtcac ccttccatgg ccaccatgct cctgtgctgg gtagcccttt

242101 gtctcctggg agtaggtgag tccctagaaa ttaagagtct gcatttctgg atattcccca

242161 ttctgtgtcc atttatgctc cttatcgtgt tcccaaactc tgtcctcttc cacagagccc

242221 acacatgctg gagtcaccca gacacccagg cgcaaagtga caacaagggg acaaggagtg

242281 actctgagtt gtgagccagt ctcaggccat aatgtcattt attggtacag gcagacctca

242341 gtgcagggac tggagcttat gatttacttt cgtagtcaaa ctactgtagg tgacacaggg

242401 ctgcccaacg agcgattctc agctcagatg cctaatgagt catgctctac tctgaagatc

242461 cagcccacag aacccgagga ctcagccgtg tacctctgtg ccagcagctt agtgccagca

242521 atttagccac agtgcagcac agtcaccccc tccctgtaca gaaacctcct gcttcccctt

242581 ctctcactag ccccaggtgt cctgtgtaag ataattctct cctctgcttg caccaaacga

242641 catgtgtggg ctagggacat gacaaggaca gaaaagaaaa ggtatcaaca gagaaacccc

242701 tgggaaggga tcagtgtaag acatgggctt taggttttcc tcacacactg tctgtgggct

242761 caacactctg cacacctgga gacagacctt agttactcac actgtgtgtg ttcctgcctt

242821 cctgcaccac cctctccatc taaactgcag gaggtcctac taagggctcc tcagccctcc

242881 attctctcct ccctctaagg ctctaagaga atttcccccc aacacacacg tgctttctaa

242941 ggttccatca agaagccaaa ttctagctgt gattgtctta atttacacca aataggtgct

243001 ctgcccttta agatgcatat gcaatgaaat ttctacactg tttctttcaa aacatagaat

243061 cgaacacatg atgtccaatt tcagtgcatc ctttatcctc ctgggccctc cttgctcctt

243121 ccagaccagt ccgctctctc tggcttcaat gcccactcct cccacacatg cctcagaaca

243181 tcgccctttg ttgctgatct tactgctact tccctaccat cacatcagtc ctcattcact

243241 tcctctcccc atcagtgaag ctgtcaccac tggagacatc accatcactc agacactttc

243301 cacaggggtt tggtgtctca ctctcctggc ctcctcactt tgaataattt ttacctccca

243361 cacttttcag atgcacttca gtcattcccg tctgtgggat gagacaccaa tgaccccctt

243421 ccaaaactgc catttcaatc aactcattct cctccctcat catatttttc ttctcactaa

243481 ttcagcacca tcctcctaag attgctttag ccactgaccc tcagttcatg ttcctaacag

243541 tttttaaact cacatgtttt taattctcac actgtctaaa ctgggtttac tggtggatac

243601 tttaatttga accctgggta aatccagggt gcttatgggt ggcagggtgg gagttcctgt

243661 gtcactgaga gcattcggca gtgatcgtga cacagcctgg tcgctgacag gaactctctg

243721 atcctcactg aagagccttc cattggttcc ccgctatgca caggaaatgc ttaggtcaat

243781 aggatgcttt gctgacatta taagaatcta ttttcactga cctggtgtgt tggtgtcctt

243841 ttattagaat caggaacttc atccctggct ggtgtggctc agtggattga gtgctggcct

243901 gggaaccaaa gggtcagagg ctcacttccc agtcaggtca catgcctgtg tcatggccag

243961 atccccagga gggggcgcat gagaggcaac cacacactga tgtttctctt tctttcccct

244021 ttccttcccc tgtctctaaa aataaataaa ttaaaatttt aatttacaaa ccaaactttg

244081 taacagtaca ttcactaaat aatgaagctc atccaataac ttagtaatga agctcaaatt

244141 aggatatgat cccattcatg tgaaaacatt tatatgaata aagaaagcag gaaaatgcat

244201 agatatagat tgatatctac tgattcatag gactgttcgt atattttctc aaattttaga

244261 aattacataa tgatgcttac aagctctttc tactttgcta aaaacagaag cacatgtctt

244321 tctggtatgc catgttttac tataaattta tatgagccag gacaaatcta tgaaacctac

244381 agatcaagtt tttccccgta attcaacaaa gtgagactgg aggtggaaga ggggtgatgc

244441 ttactgctgt gttccatatc aatttgtctc agttactagc atgttcattg tgaagtactg

244501 ctccccagta ttccttcagc gcatatgaga tcttaaatag aaaagaagaa ttcgctataa

244561 acaaaagtct gtgagtcaga gcagtgaggg gtggtaaaga ccagagagat gagcgctgtt

244621 tctcaccaga aagagccctg gtgcggggcc acatacccgg acacttgtga ctgtcatatg

244681 tgacagttat atctggagtt cacctttggg aggccatccc atgagcagtg acaaggatcc

244741 atgtcactct ccaccttgtg gaacccagaa gaaaagaagc ccggcccctt gatttatgtt

244801 cacaggggcc tcacctggga gaagcctcct cctgccccac cctgccatgg ccaccaggct

244861 cctctgctgg gtggcccttt gtctcctggg cataggtgag tccccagaaa ttaagaatct

244921 gcatttggtg gatattccca gctttatttc ctttcatgtt ccttatcgtg atcccaaact

244981 ctgtcctctt ccacagagcc cacacatgct ggagtcaccc agacacccag gcacaaggtg

245041 acaatgagag gacaaggagt gactctgagc tgtgagccaa tttcaggcca taatgacctt

245101 tactggtaca gacaggcatc agtggaagac ataaccttgc tgatttcctt cggtcgtaca

245161 atactcctgg agaagacaag tctgctcaat gagcgattct cagctcagat gcctaatgag

245221 tcatgctcca ctctgaagat ccagcccaca gaacccgggg actcagccgt gtacctctgt

245281 gccagcagat tagccacagc gctacacagt caccccctcc ctgtgcagaa acctcctgct

245341 tccccttctc atcagcccct aggtgtcctg tgtaaaagtc ttctctgctc agctgtcacc

245401 aagagacatg tgtgggtttg gggcatgaca atggcagaaa agaaaacgta tcaacagaga

245461 aaccccagca caaggaacaa tgtaggaagt gggctttagg ttttcctcac acactgtcgg

245521 tgggatcagc actctgcata cctgagagac agaccttagt taccctcact gtgtgtgctc

245581 ctgcctcctg ccctggcctc tccatctgaa ctgtgggagc ttctggtaag ggctcctcag

245641 acctccattt tcttctcctt ctaatctcct aacagaatga cccaatgacc acacacacac

245701 acgttttcta gaatctgtct tatggccaaa ttgcacctgt cattgtcctt tctgttattc

245761 agggatgctg atcttgaagt tggaaataga ataaaattcc tactgtgaat tccaagagac

245821 atattctgct cctctcttca atttcagtgt attctacatc ctcccaatcc tgcctatggc

245881 ttctagaaca ttctgctctc cctggtttta atacccagct cctgagaaca atctatgttg

245941 ccctgaattc tctatatgac atctgtcctc attcacttcc ctccccacca ctgatcctgc

246001 cacaactggt gatgcggtta acatgtggac aatttataca caggtctgga ttaacagttt

246061 cctgacctct tcattttcaa taatattttg cctccattgt cttgtaagac taccatccat

246121 tctagaccat ggagtgaaca acaaatagtc tgcttctaaa atgtccattt cattattcca

246181 tactcctttc tctttctcct ttctttcttt aatgcaaaat ctactttcga tgattcttat

246241 agttactgga cttcagccca tatacacaac tgctttgaaa ctcttatgcc ctatttctca

246301 cattatctac accaggtaca aggattagta ccataaagtc aaccctggtt aaatccaact

246361 ctcctttccc agggcatctg tttatgtacc ttcataatca ccattgagtg taatctaaat

246421 tttcctcgga gggattgggg taaagcctca actcagccag aatttattta gatttgtgtg

246481 cctgggagtc agggggtgtc ttgagaggag gatggaggac gtgagactgg aagtgctgtc

246541 ttggtgccca gaatctgcag tgccattggc acacccacac tgacgtgcca ggagaggtgg

246601 acacgtgctg cagcacagaa caagggagga cggaagccca ggttgtgtga ggtgagtgtt

246661 gttaacagtc ttggagacat taaaagactc cactgcattc caggtgagac ttcacctatt

246721 gcagtcatct tctgcagagg agcactgttc agctttagga ggaggcactg tgacagatga

246781 tgaagaccta tgtcactacc aagccactct ccaccttgtg gagcttagga gaaccagaag

246841 ccctgcccct gtgttcaggc tcaccagggc ctcatctggg tgaagcctcc tcctgcccca

246901 ccctgccatg gccaccaggc tcctctgctg ggtggccttt tgtctcctgg gcataggtga

246961 gtccccagaa atgaagattt tacatatttt taatatcccc agttcttttt tcattcatgt

247021 cccttatcct gttcccaaac tctgtctctt ccacagaacc cacacatgct aaagtcaccc

247081 agtcaccctg gtgcaaggta acaaagcggg gacaaggagt tactctgagt tgtgagccaa

247141 tttctgccca taaacgcgtt tctggtacag gcagacccca gtgaagggac tggaattact

247201 gttttacttc caaaaccaat ttcctgtgga caacacaggg atgcccaagg agcgattctc

247261 agctcagatg cctaatgagt ccttctccac tctgaagatc cagcccacag aacccaggga

247321 ctcagccgtg tacctctgtg ccagcagctt agccacagcg ctgcacagtc accccctccc

247381 tgtgcagaaa cctcctgctt ccccttctct caccagctcc caggtgcccc gtgttaaaca

247441 cttctctgtt ccacttacat ccagagacat gtgtgtgttt ggggcatgac aagagcagca

247501 aggaaaatgt gtcaacagac aaaccccagc acagggaaca atgtaggaag tgggcttagg

247561 ttttccttac acattgactg tgggctcagc actccccaca catcagagac agacttcagt

247621 tacccacact gtgtgtatcc ctgccttcct gccctggcct ctccatctaa actgtgggag

247681 cttcttgtaa gggctcctca gctacccatt ctcctctcct tctaatctcc taacagaatg

247741 atccagtgac cacacacaca cacacacacg ttttctggag tctgtcttat atccaaattc

247801 cattcagcat tgttcattct gtcatccagg ggtcctgatc ttcaatttag caatacaata

247861 aaattcctac tttgatatca agacttggaa tctgcttctc tgtccaattt tgtgcattct

247921 tggtcgtaca aatactaact actcattcta gaacattccc ctctccatgg ctttcataca

247981 cagttcctca cacaggtgga ttagaacacg gcctttcatg ttgccctgac ttctctaccc

248041 tcagatctgt cctcactccc ttcctcccca ccacggatcc tgccacccac tggggatgtc

248101 atcagcctgt ggacaattta tacacgggtt tgcttcccag cttcctcctc tcttcacttt

248161 cagtaatgtt ttatctccaa tcctatccaa gagcacttcc aagtctaggc catgcagtga

248221 gcaaagatat tctccttcca aaatgtccat ttcacattcc atgctccttt gtctttctct

248281 tttctttaat gcaaaatcca ctccttgtga ttctcatagt ctctaacctt cagcccacac

248341 acgcaactgc tctgaaactc tcaggtctta ttcctcacgt tatctaatcc aggtacaagg

248401 attagctcta taaaatcaat gctgcttgac tccaaccctc ctttcccagg gcatctgttt

248461 ccctcccttc acaattaccc ttgggtgcaa tctaaatgat tctgggacag attatgaaaa

248521 agccccagct cagccagaaa ttattcagat ttgtgtgtct ggaagtcagg gagtgccttg

248581 acaggactat gaaggatgca aagatcacag tattgtcttc ttggccacaa tctgcagtgg

248641 ctatggcaca cacacgccgc catggcaggc agatggtaga ctatggagac atcagggaac

248701 aagaaatggt ggaagcacag ggttgtgaga agtgacactt gatgaaagtc ctggagactt

248761 tgaatgctcc atccaaaccc tgaaagagac atgacccatt ctagtcaatt caactgacaa

248821 ttaatgtcca ctgttggtag gaggtcctat gacaaatgac agacagcgat gtcactacca

248881 agacacactc caccttgtgg agcccagagg aaccagaagc cctgctcctg tgttcagggt

248941 cacaagggcg tcatctgggt gtagcctcct cctgtcccac cctgccatgg ccaccaggct

249001 cctgtgctgg gtggcccttt gtctcttgag cagaggtgag tctccagaaa tgaaaactct

249061 aattttttga tattaccagt tgtgacttca cttatgttcc taatcctata ccgaaactct

249121 gtcttcttcc acagggtcca cagatgctgg tgtcacccag acacccagac acaaggtgac

249181 agacagggga caagaagtgg ctctgaattg tgagccaatt gcaggccatg actaccttta

249241 ctggtacaga caggcctcag cagaaggcat aaagtttctg atttccttca gctttgccaa

249301 acctctggat gagtcaggaa tgcccagtga gcgattctca gctcacatgc ctaataagtc

249361 cttgtcgatt ctgaagatcc aggccacaga acccggggac tcagccgtgt acctctgtgc

249421 cagcagttta gccacagcac tacacagtcg tcccctccct gtgcagaaac ctcctgcttc

249481 cccttctctc accagcccgc aggtgtcatg aataaaagtc ttctctgctc tgatgtctcc

249541 aagagacttg tgtgggtttg agacaggaga aggacaaaaa ggattaggtt tcaacttaga

249601 aacccctgca gaggaaacaa cgtaggacat gagcttcagg gaaccctcac accttgactg

249661 tgggctcaac actcaccaca cctgagagat agactttagt caccctgacg tttattttct

249721 tgcttcctgc atttgcctcc acctgcactg tcagacctcc tggtcatggc tcctcagcct

249781 tcccttgtcc tctccctgaa aactgccaac agaattctga atcccccgac acacacctgt

249841 cctagggttc tatcatacag acaaattcaa cctgctgttg tccttgtttt tgttattcag

249901 tggcccttaa catcaatatg aaagaataaa tttctactgt atttatactg aaatgcataa

249961 gctgcttcta tgttcagttc aatacattgt ttgtcctccc catcctacct tctgcttcta

250021 gaacattctc ctcccatagt ttttcatacc cagtccttca tacgcatgga ttagagtgtt

250081 ggcctttctt cctaagctca cttctccacc agaggacaag ttcttggtca ctgcccctct

250141 cactgcagat cctgccatca ctggtgccat caccaccatc acatggacaa gttatacacg

250201 ggtttggggt ctcagtgtcc tgacctcctc actgtcaata attttgtgcc tccaatgttt

250261 tccaagtgca ctcccacgtc ttgaccatgc agtgaagaac aaaaaagctc cttccataat

250321 ttccatttta tcattccatg tgcctatctc tttctccttc cttgctttct ttaacacaaa

250381 atcatctgcc tgtgttactt agagtcactg agctgccccc catccaacaa ctctgaaatg

250441 ctcatgtcgt atttctcatg ctgtctaaac ccccttaaag agttaacact ataatgtcaa

250501 ccctgggtga atcgaaccct ttcttcccag ggcatttgta tagttccctt cataatgacc

250561 atggtggcca atcagaatgc ttctaggaga ccttgggtaa aaggtgcagc tctgccagga

250621 agtattatat ttgtctgtct ggagtcgggg actgtcttga gaggagtacg aaggatgtga

250681 gactggaagt actgtcttgg tgcccaggtt ctgcagtgcc attgacacac ccacactgaa

250741 atcccaggag atggtaaacg tgtgtactgg cataaaataa agaatgattg aaacacaggc

250801 tgtatgcgct gagcattgtt aaaagtcttg gacactctga atgatccaaa tgaatctcaa

250861 aagggacatg acctagtcca gtcaatgcct ctgaagagga ttaatattca catttgggag

250921 gaggggttgt gagaaatcac agagaagcat gaaactagtc agccagtctc caccttgtgg

250981 agcccagagg aaccagaagg cctgccccta tgttcaggat cacaacggcc tcttccaagt

251041 gaagcctcct cctgccccac ctgccatggc caccaggttc ctgagcttcc tggccctttg

251101 tctcctggta ataggtaagt ccccagaaat gaagagtctg catttctgga tattccccat

251161 tctgtgtcca tttatgctcc ttatcctgtt cccaaactct gtcctcttcc acagatccca

251221 tacatgctgg agtcacccag acacccaggc acaaagtgac aacgaaggga caaggagtga

251281 ctctgagttg tgaggcaatt ttaggccata atgtgcttta ttggtaccga gagacctcag

251341 tgcacggact ggagcttatg atttactttc atagtcaaac tattgtaggc gacacagggc

251401 tgcccaagga gcgattctca gctcacatgc ctaaggagtc atgctccact ctgaagatcc

251461 agcccacaga acccggggac tcagccgtgt acctctgtgc cagcagctta gccacagtgc

251521 tgcacagtcg ccccttccct gtgcagaaac ctcctgcttc cccttctctc accagcccca

251581 ggtgtcctgt gtaagatagg tctctcctct gcttgcacca agagacatgt gtaggtttgg

251641 agcatgacaa ggagagagga aaatgtatca acagagatac ccagcacagg gaacaaagta

251701 ggaagtgggc tttaggtttt cctcacacac tgtctgtggg ctcagcactc tgcacacctg

251761 agagacagac ctgagttacc cacactgtgc atttttctgc ctgcctgcac caccctctcc

251821 atctaaactg tagcaggtcc tactaagggc tcctcagccc tccaatctcc tctccctcta

251881 atgcggtaac gtaatttccg cccaagacac gcgtgttttc tagggttcta tcatgcagcc

251941 aaattctagc tttgattgtc ttaatttata ccattaaggc actctgccct ttaaggtgga

252001 tatgcaatga aatttctctg tgtttctttc aaaacataga atcgaacaca tgatgtccaa

252061 tttcagtgca ttcttcatcc tcctgggtcc tcccacctcc ttccagacca gtctgctctc

252121 tctggcttca ccacccactc ctcacacaca tgtctcagaa catggccctt tgttgctgaa

252181 ctcactgcta cttctctact gtcagatctg tcctcattca cttcttctcc caccagtgaa

252241 gctgtcacca ttgggaatgt catcatcact cagacacttt ccacaggggt ttcttgtctg

252301 tgtctcctga ccccctgact tgcaatgatt tttacctccc acacttttca gatgcacttc

252361 agtcattcct gtctgtggga tgagacacca atgagctccg tccaaaattg ccactccaat

252421 catctcattc tcctccctca tcatgttagt tttcttctca ctaattcagc accaccctcc

252481 taagattcct ttaaccattg accctcagtt catgttccta acagctttta aactaacaca

252541 tatgttttta attctcacac tgtctaaact gggtttgttg ttggatactt taatttgaac

252601 cctgggtaaa tccagggtgc ttatggatag cagggtggga gttcctgcga cactcagagc

252661 attgggcagt gatcacacac agcctggtca ctgacaggaa ctcttgatcc tcactgaaga

252721 gccttgcatt ggttccctgc tgtgcacagg aaatgcttag atcaatagga cactttgcag

252781 acatttaaag aatctatttt cacggacctg gtgtgttggt gtcattttat taaaatcaga

252841 aacttcagcc ctggctggtg tggctcagtg gattgagtgc tggcctggga accaaagggt

252901 cagaggctca attccctgtc aggtcacatg cctgtgtcat ggccagatcc ccaggagggg

252961 gcgcctgaga ggcaaccaca cactgatgtt tctctctctt tccccttccc ttcccctgtc

253021 tctaaaaata aatcaattaa aattttaatt taaaacggaa actttgtaac agtacaatca

253081 atcaataatg aagctcatac agttacttaa taatggagct caaattagga tatgatccca

253141 ttcatgtgaa aacacacata tgaataaaga aagcaggcaa atgcatagat aaagatttat

253201 atctactgac ccataggact gtttgtatct tttctcaaat tttagaaatt acatgatgat

253261 gcatagaagc cctttctact ttgctaaaaa caaaagcaca tgtctttctg gtatatcatg

253321 ttttactata aatttatatg agccaggaca aatctatgaa acctacagat caagtttttc

253381 cccgtaattc aacaaagtga gactggaggt ggaagagggg tgatgcttac tgctgtgttc

253441 catataaatt tgtctcagtt actaccatgt tcattgtcaa gtattgctcc ccaggattcc

253501 ttcagtgcat atgagatctt aaaaaataga aaagaagaat ttgctacaaa caaaagtctg

253561 tgagtcagag cagtgagggg tggtaaagac cagagagatg accagtgatt ctcaccagaa

253621 agagccctgg tgcggggcca catccccgga cacttgtgac tgtcatatgt gacagttata

253681 tctggacaga tttggtgttg acctttggga ggccacccca tgagcagtgg cagggatcga

253741 tgtcactctc caccttgtgg aggcaagaag aaaagaagcc cggccccttg gttcatgttc

253801 acaggggcct cacgtgggag aagcctcctc ctgccccacc ctgccatggc caccaggctc

253861 ctctgctggg tggccctttg tctcctggga gtaggtgagt acctacaaat gaggaatctg

253921 catttttgga tattcccagt tcagtttctt ttcttgttcc ttatcctgat cccaaattct

253981 gtcctcttcc acagagccca cacatgctgg agtcacccag acacccagga acatggtgac

254041 aaagagggga caaaatgtga ctatgagttg tgagccaatt tcaggccata actaccttta

254101 ctggtacaga cagacctcag tgaagggact ggaatttatg atttacttca cctatgcaaa

254161 acctgtggac aaggcaggaa tgcccaatga gcgattctca gctcacatgc ctaatgcgtt

254221 actctccact ctgacgatcc agcgcacaga acccggggac tcagccgtgt acctctgtgg

254281 cagcagctta gccacagcgc tgcacagtcg ccccctccct gtgcagaaac ctcctgcttc

254341 ccctcctctc atcagccccc aggtgtcctg agtaaaagtg ttctctgctt cgctgtcacc

254401 atgagacatg tgtggtttgg gggcacaaca aggacagaaa ggaaaagtta acaatgaaga

254461 gaaccctgca agggaaacgg tatagaaaat gccctttagg ttttcctcac acattttctt

254521 taggctaaac actcaaccca tcaaagagac agactgactt acccacactg ggcatgctcc

254581 tgccttcctg caccagcctc tccatctgaa atgttggacc ttgtggtaat gcctcctcag

254641 actcccattc tgctctcctt ctgaggtcct accagaatcc accctcccac cacgcacaaa

254701 tggtttccaa gtctgtctta tagccaaact ctacttcgta ttgtcctttc tgtcattcag

254761 ggatgctgat cttgaagatg aaaattgaat aaaattccta ctgtagatgc caagggacat

254821 gttctgcttc tctgtccaat ttcagagcat actttgtcct ctcacttcga cctactgctt

254881 ctagaacatt ctcctctctc gggctttagt acccagctcc tcacacacat ggattagaat

254941 attgccttcc atgtttccct gacttctcat tctgtgctcc ttcacttccc tccccaccac

255001 tgatcctgcc atccctgggg acatcaccct cctgtggaca atgtatacgc gggcttggct

255061 tctcagtgtc ctgacctcct cactttaagt aatgttttgt ctctgatctt ttccaacagc

255121 acttccaagt ctggaccagg aggtcttgac tttaacaaca aagagtctcc ttccaaattt

255181 tccacttcgt gattctgtgc tcctatcttt ctcctttctt tccttaacac catgtctatg

255241 tcctaagatt cttatagtct ctgaacacca gcccatttac ccaacttctt tgaaactctc

255301 atgtcttatt tctcacaata tctacatcag gtacaagaat gagtatcata aagccaaccc

255361 tggataaatc caacccacct ttcccagggc atctgtttat gtaccttcat aatcacccat

255421 ttgtccaacc taaattctcc ttggaggaat ggggcaaaag cctcagctca gccagaatta

255481 tttagatgtg tgtgtctggg agtcagggag caccgagaga ggagcgtgaa gcacatgaag

255541 ttcgactcac tgtcctggtg cccagaatat gcacacgccc ttggaaatgc ccggcagaca

255601 gtagacaatg gatacagcag agagcaagga gcgggaagca caggactgtg agaagtgaca

255661 attgatgaga gtcttggaga ctttgagtgg tccaaacaaa cctcaaaaga gacatgacct

255721 atcccagtca attcaacagg ggaggattaa tattcgctct tggagggggt cccctgagaa

255781 atggtgggga gtgatgtcac taccaccaca ctcctccttg agaagccgag aggaacaaga

255841 agccctgccc ctgtgctcag gttcacaagg tccccatgtc agggagcctc ctcctgcccc

255901 accggccatg gccaccaggc tcctctgctg ggtggccctt tgtctcctgg gggtaggtga

255961 gtccctagaa atgaggaatc tgtatttgtt gatattccca gttctgtttc catttatgtt

256021 ccttatcctg atcccaaact ctgtcctctt ccacagagcc ctcacatgct ggagtcaccc

256081 agacacccag gcagaaggtg acaaagaggg gacaagatgt gactatgagc tgtgagccaa

256141 tttcaggcca ccctgacctt ttctggtaca ggcagacatc ggggcagggc ataaagttac

256201 ttatttactt cagcaatcaa agtccgctgg acaacacagg gatgcccaag gagcgattct

256261 cagctcagat gcctaatgag tcatgctcca ctctgaagat ccagcccaca gaacccgggg

256321 actcagccgt gtacctctgt gccagcagct tagccacagc gctacacagt caccccctcc

256381 ctgtgcagaa acctcctgct tccccttctc tcgtcaggtc ccagaagttg tgagcaaaag

256441 acttgtctgt tgtgctgtca tgaagataag tgttctgttt tggggcatga caagtgcaga

256501 aaggaaacgg tatcaacaga gaaatctctg catgggagac agtgtaggaa atgggcttta

256561 ggtttttctc acaccctgtc tgtggactca aaggtccgca cacctggaga cagacctctg

256621 ttacccacac tatgcccgtt gctgccttcc tgcactgccc actctgtctg aatggtagga

256681 gcctctgcta atggcgtttt gccctccatt ctcctctggt tccaagatgc taacagaatt

256741 ctcccccaca acacactcat gttttctggg gttccttcat gtagaccaat tccaggtatc

256801 attttcttaa ttacaccatt aaggtgctct accctttaag atagaaatgc aataaaatat

256861 ctactgtgtt tatatcacaa cataaaactg aacatttaac atcttgtttc agcccattct

256921 ttatcttcct gggacctccc tgcttctccc agaccagttg cctctccagc ttcaccaccc

256981 agtcctcaca cacatgcctc agaacatcgc cctttgttgc tgaacttatt gctacttctc
[truncated: 1,147,852 more chars]
