# Supplementary figures and images for: New insights into the germline genes and CDR3 repertoire of the TCRβ chain in Chiroptera
Source: Front Immunol. 2023 Mar 27;14:1147859. doi: 10.3389/fimmu.2023.1147859 (PMC10083501; doi:10.3389/fimmu.2023.1147859)

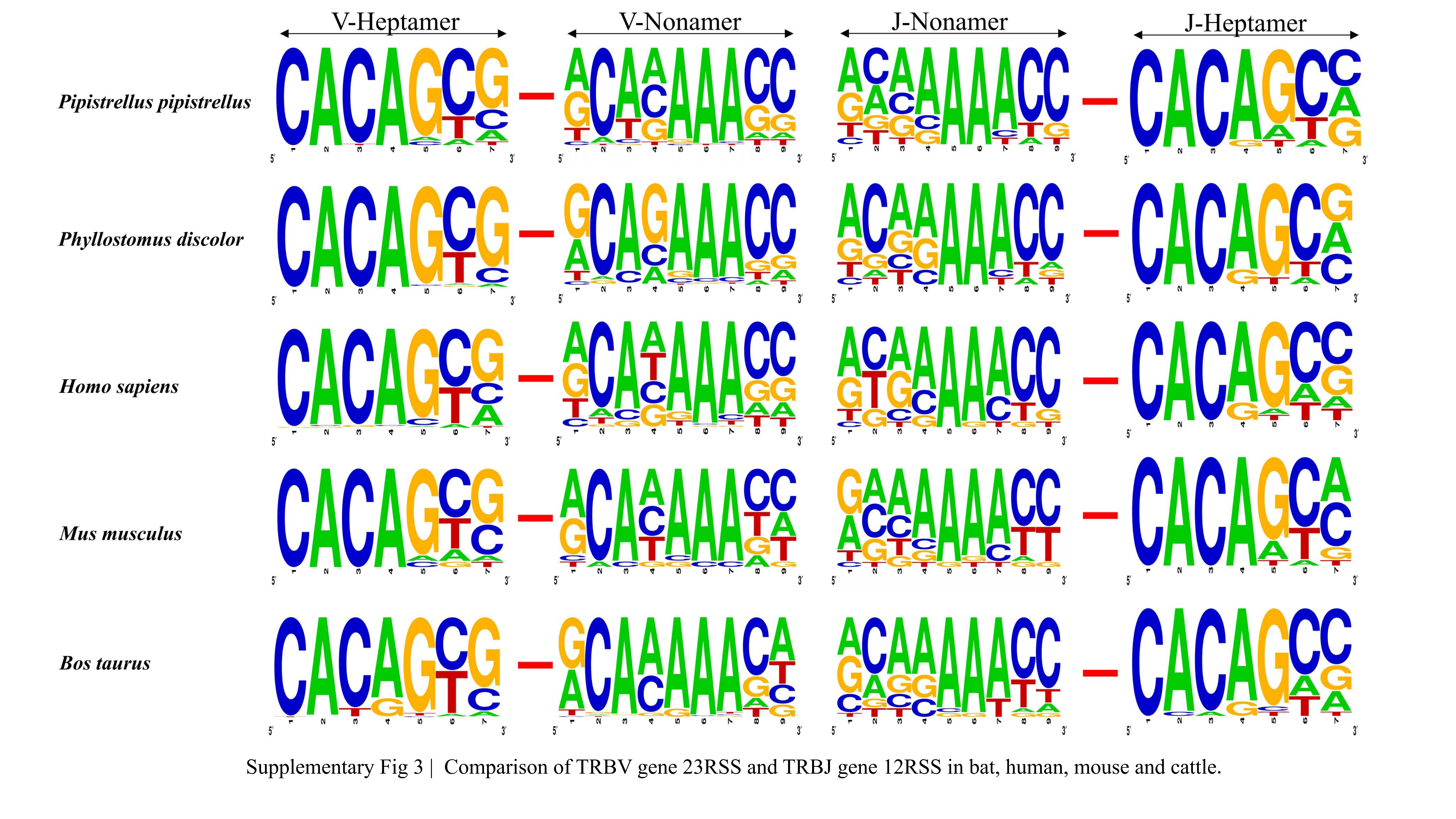

Supplement: Supplementary file 4 [file Image_3.png]

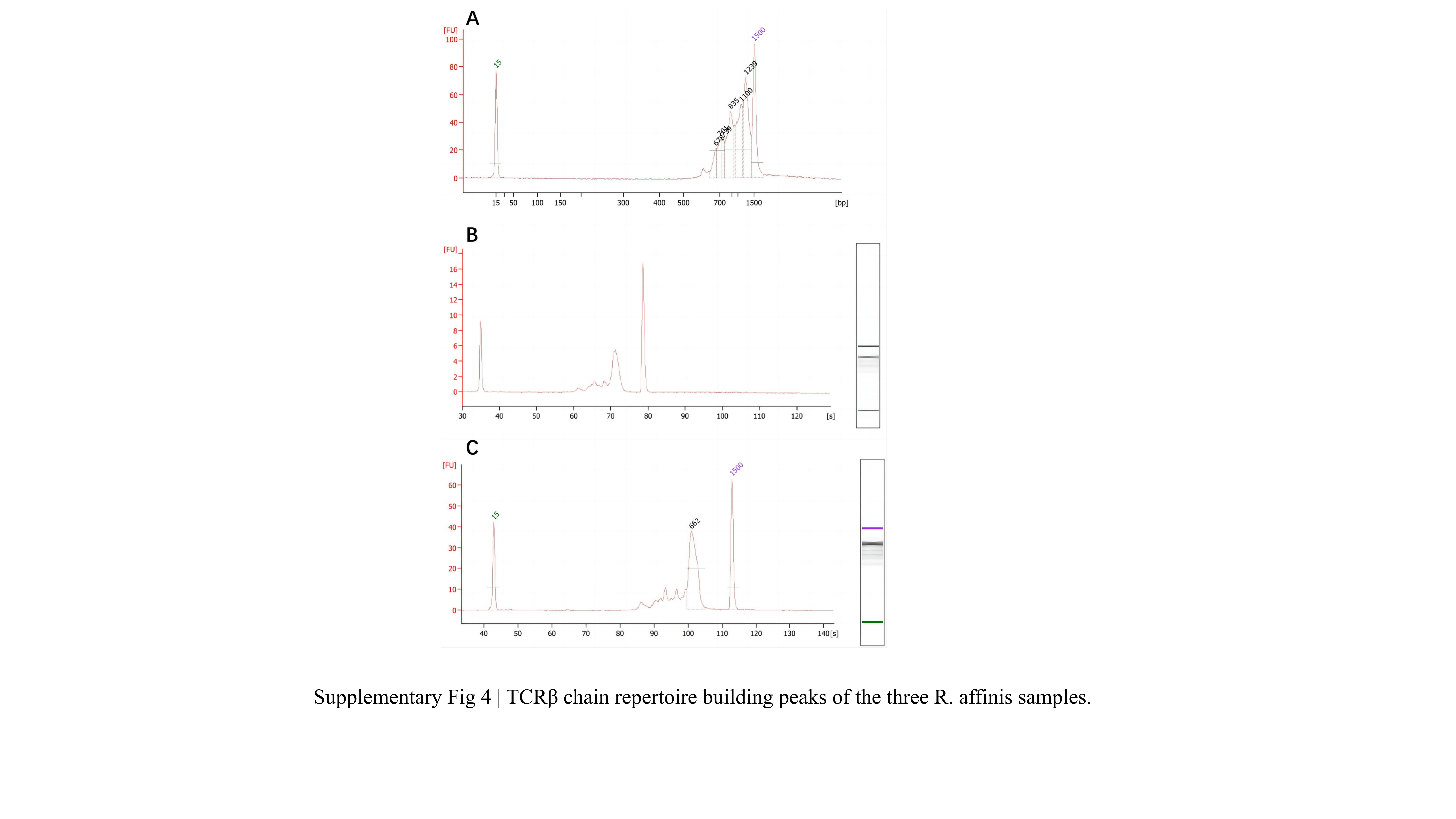

Supplement: Supplementary file 5 [file Image_4.png]

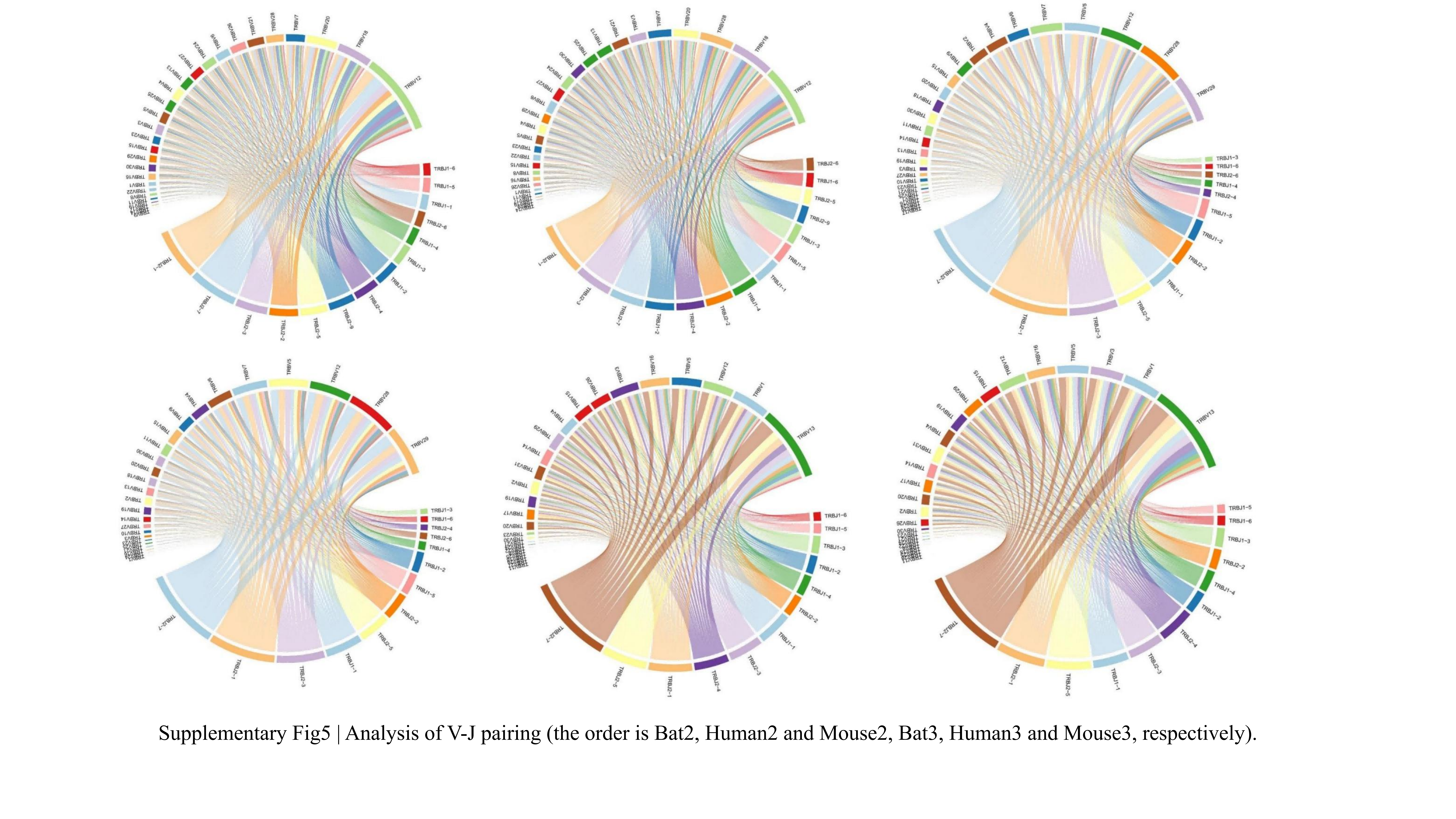

Supplement: Supplementary file 6 [file Image_5.png]
